# Supplementary material for: HMBA ameliorates obesity by MYH9‐ and ACTG1‐dependent regulation of hypothalamic neuropeptides
Source: EMBO Mol Med. 2023 Nov 20;15(12):e18024. doi: 10.15252/emmm.202318024 (PMC10701615; doi:10.15252/emmm.202318024)
Supplement: Supplementary file 1 — Appendix S1 [file EMMM-15-e18024-s004.pdf]

Appendix

HMBA ameliorates obesity by MYH9 and ACTG1-dependent regulation of hypothalamic neuropeptides

Appendix Figures 3

Appendix Figure S1. 3

Appendix Figure S2. 5

Appendix Figure S3. 6

Appendix Figure S4. 7

Appendix Figure S5. 8

Appendix Figure S6. 10

Appendix Figure S7. 12

Appendix Figure S8. 14

Appendix Figure S9. 15

Appendix Figure S10. 16

Appendix Figure S11. 17

Appendix Figure S12. 18

Appendix Tables **20**

Appendix Table S1. 20

Appendix Table S2. 21

Appendix Table S3. 22

Appendix Table S4. 23

Appendix Table S5. 24

Appendix Table S6. 25

References **29**

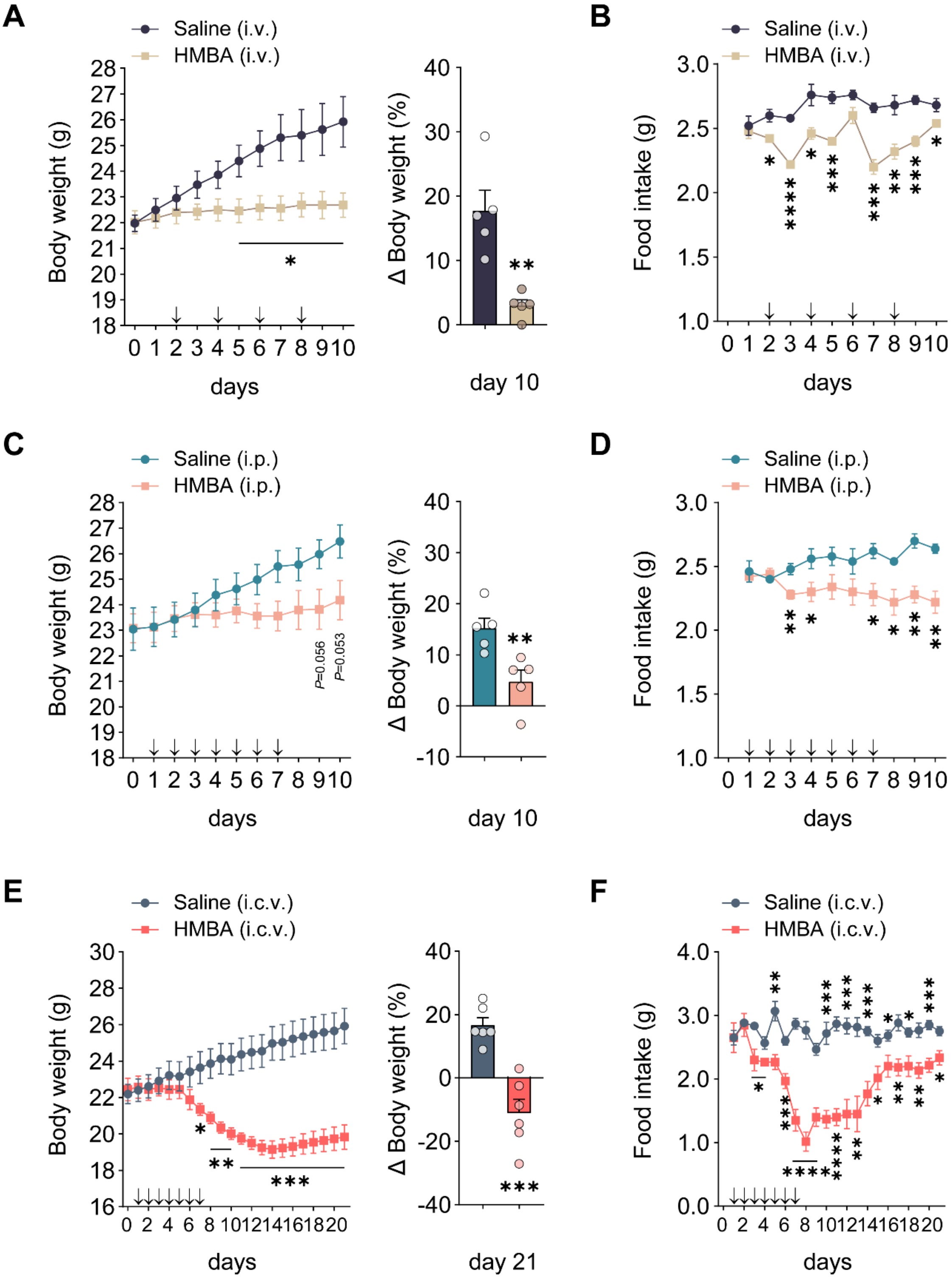
A
Saline (i.v.)
HMBA (i.v.)
Body weight (g)
days
Δ Body weight (%)
day 10
B
Saline (i.v.)
HMBA (i.v.)
Food intake (g)
days
C
Saline (i.p.)
HMBA (i.p.)
Body weight (g)
P=0.056
P=0.053
days
Δ Body weight (%)
day 10
D
Saline (i.p.)
HMBA (i.p.)
Food intake (g)
days
E
Saline (i.c.v.)
HMBA (i.c.v.)
Body weight (g)
days
Δ Body weight (%)
day 21
F
Saline (i.c.v.)
HMBA (i.c.v.)
Food intake (g)
days

Appendix Figure S1. Peripheral and central administration of HMBA has anti-obesity effects in female DIO mice

A–B. Changes in (A) daily body weight (grams) (left panel) and body weight (percentages) on day 10 relative to day 0 (right panel), and (B) food intake in female DIO mice injected i.v. with HMBA. Mice were injected with 500 mg/kg of HMBA on day 1, followed by three injections of 1,000 mg/kg bi-daily. n = 5 per group. Arrows represent the days of each injection.

C–D. Changes in (C) daily body weight (grams) (left panel) and body weight (percentages) on day 10 relative to day 0 (right panel), and (D) food intake in female DIO mice injected i.p. with HMBA. Mice were injected with 1,000 mg/kg of HMBA daily for 7 days. n = 5 per group. Arrows represent the days of each injection.

E–F. Changes in (E) daily body weight (grams) (left panel) and body weight (percentages) on day 21 relative to day 0 (right panel), and (F) food intake in female DIO mice injected i.c.v. with HMBA. Mice were injected with 400 nmoles of HMBA (2 µl per injection) daily for 7 days. n = 6 per group. Arrows represent the days of each injection.

Data information: Data represent different numbers (n) of biological replicates. Statistical significance was determined by a two-tailed unpaired Student's t-test. *P<0.05, **P<0.01, ***P<0.001, ****P<0.001 vs. Saline. Data are mean ± SEM.

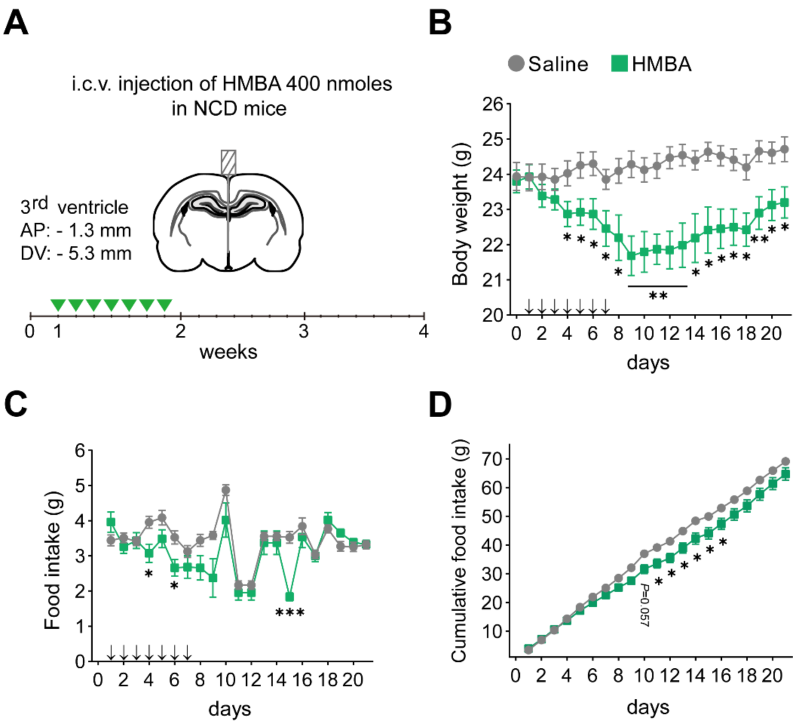

Appendix Figure S2. Central administration of HMBA has anti-obesity effect in mice NCD mice

A. Schematic of the experimental design. HMBA (400 nmoles per injection) was injected daily for 7 days. AP, anterior/posterior; DV, dorsal/ventral. Triangles represent the days of each injection.

B–D. Changes in **(B)** body weight, **(C)** food intake, and **(D)** cumulative food intake. $n = 7$, Saline; $n = 8$, HMBA. Arrows represent the days of each injection.

Data information: Data represent different numbers (n) of biological replicates. Statistical significance was determined by a two-tailed unpaired Student's t-test. *$P<0.05$, **$P<0.01$, ***$P<0.001$ vs. Saline. Data are mean ± SEM.

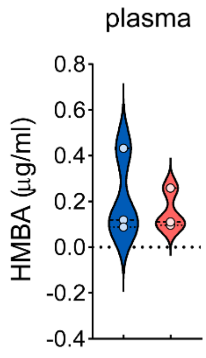

Appendix Figure S3. Central administration of HMBA does not affect plasma HMBA concentration

HMBA (400 nmoles per injection) was injected intracerebroventricularly daily for 7 days. Blue, saline injection; red, HMBA injection. n = 3 per group.

Data information: Data represent three biological replicates. Statistical significance was determined by a two-tailed unpaired Student's t-test. There was no significant difference between the groups. Data are mean ± SEM.

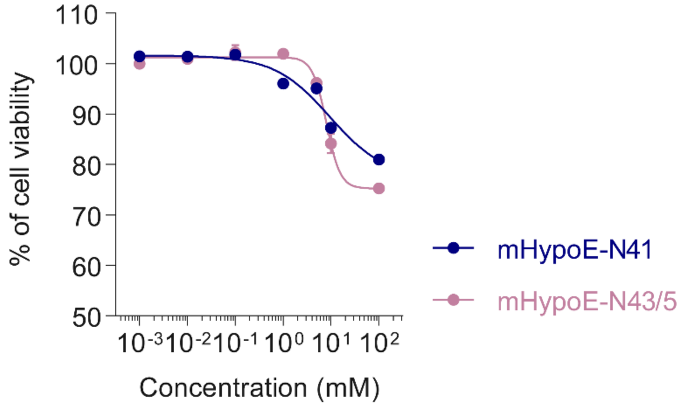

Appendix Figure S4. Cell viability in hypothalamic cell lines at different HMBA concentrations

Cells were treated with various doses (10^{-3}, 10^{-2}, 10^{-1}, 10^{0}, 10^{1}, 10^{2} mM) of HMBA for 24 h. Cell viability was measured using a CellTiter-Blue cell viability assay kit. Data are mean ± SEM. *n* = 5 per group; Data represent five technical replicates.

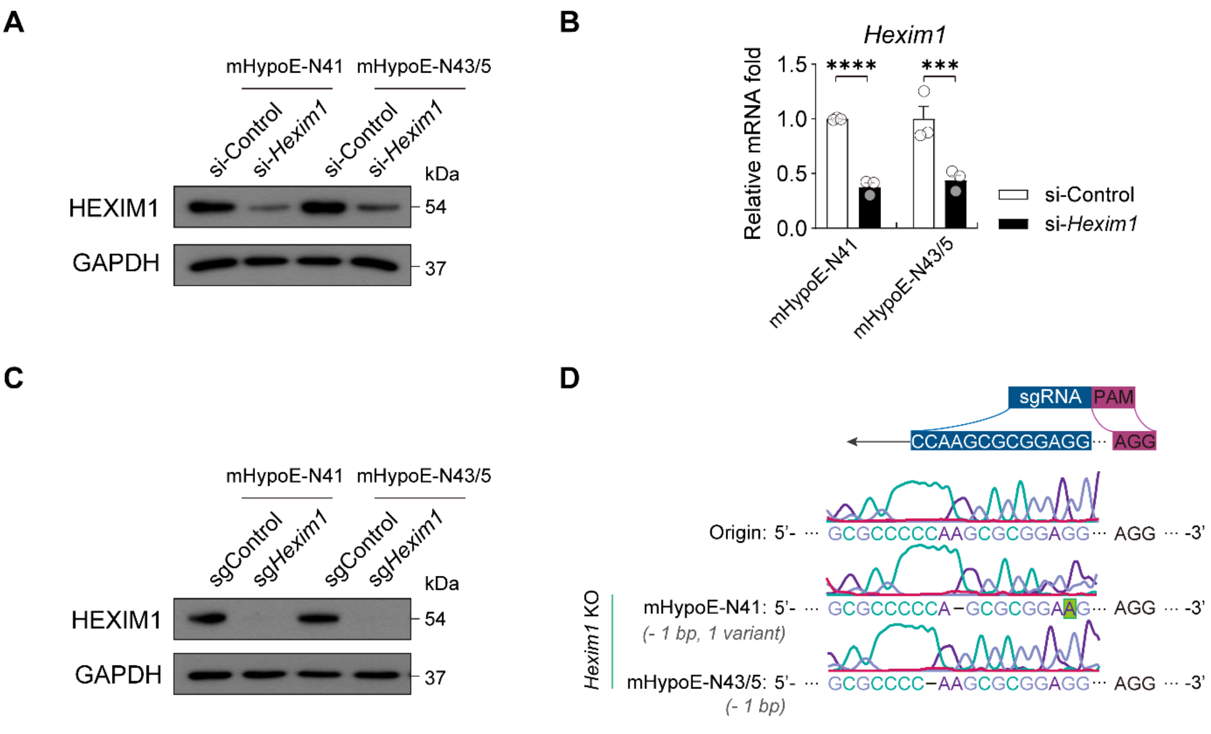

Appendix Figure S5. The efficiency of *Hexim1* knockdown and knockout

A. Western blot analysis of *Hexim1* knockdown (si-*Hexim1*) in mHypoE-N41 and mHypoE-N43/5.

B. Relative *Hexim1* mRNA levels after *Hexim1* knockdown in mHypoE-N41 and mHypoE-N43/5. $n = 3$ per group.

C. Western blot analysis of *Hexim1* knockout (sg*Hexim1*) in mHypoE-N41 and mHypoE-N43/5.

D. Confirmation of *Hexim1* knockout in mHypoE-N41 and mHypoE-N43/5 by sequencing. One base pair was deleted and substituted in mHypoE-N41, and one base pair was deleted in mHypoE-N43/5.

Data information: qPCR data represent three technical replicates. Statistical significance was

determined by a two-tailed unpaired Student's t-test. ***P<0.001, ****P<0.0001. Data are mean ± SEM.

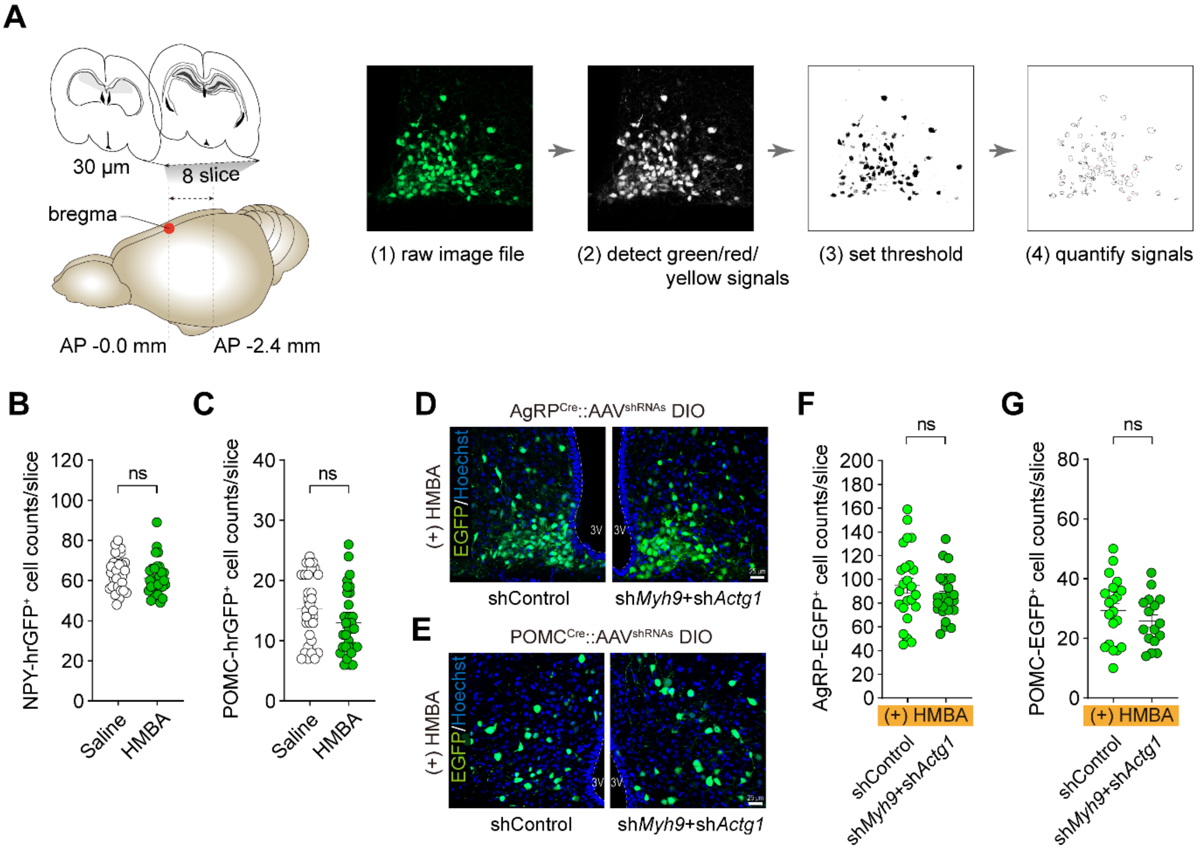

Appendix Figure S6. The number of hrGFP or EGFP-positive cells remains unchanged

A. Schematic diagram of experimental design for immunohistochemistry analysis. NPY-hrGFP and POMC-hrGFP DIO mice fed an HFD for 4 weeks were injected with HMBA (i.p., 1,000 mg/kg), and then brains were harvested 4 h after injection. AgRP^{Cre} and POMC^{Cre} DIO mice fed an HFD for 4 weeks were injected with AAV-FLEXon-shRNAs (shControl or sh*Myh9*+sh*Actg1*)-EGFP viruses into the ARC. After recovery, mice were injected with HMBA (i.p., 1,000 mg/kg), and then brains were harvested 4 h after injection. Brain slices were prepared posteriorly from the bregma, with a maximum of 8 slices per mouse and a thickness

of 30 μm. The number and intensity of positive signals (green, red, yellow) were analyzed via Fiji of ImageJ 2 software using the following sequence: (1) load raw image file, (2) signal detection by colors, (3) threshold setting of detectable cell size, (4) quantification of cell number and intensity.

B–C. The number of hrGFP-positive (hrGFP^{+}) cells per slice in (B) NPY-hrGFP DIO mice and (C) POMC-hrGFP DIO mice. *n* = 32 per group (eight brain slices were obtained from each of the four mice per group).

D–E. Immunohistochemistry analysis for the functional efficiency of AAV in the ARC of (**D**) AgRP^{Cre}::AAV$^{(\text{shControl or sh}\textit{Myh9}+\text{sh}\textit{Actg1})}$ DIO mice and **(E)** POMC^{Cre}::AAV$^{(\text{shControl or sh}\textit{Myh9}+\text{sh}\textit{Actg1})}$ DIO mice after HMBA treatment. Scale bars, 25 μm.

F–G. The number of EGFP-positive (EGFP^{+}) cells per slice in **(F)** AgRP^{Cre}::AAV$^{(\text{shControl or sh}\textit{Myh9}+\text{sh}\textit{Actg1})}$ DIO mice and **(G)** POMC^{Cre}::AAV$^{(\text{shControl or sh}\textit{Myh9}+\text{sh}\textit{Actg1})}$ DIO mice. *n* = 24 per group (eight brain slices were obtained from each of the three mice per group).

Data information: Statistical significance was determined by a two-tailed unpaired Student's t-test. ns, no significance. Data are mean ± SEM.

A

Plasmid DNA 1 : pAAV[FLEXon]-CMV>LL:rev(EGFP:Control[shRNA#1]:Control[shRNA#2]):rev(LL):WPRE
Vector size : 6055 bp
Viral genome size: 3436 bp
Virus titer (QC) : 4.20 x 10^{13} GC/mL

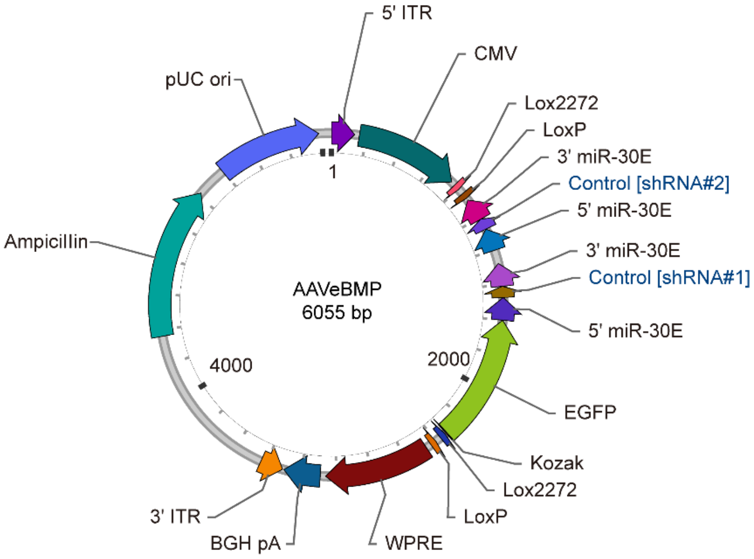

B

Plasmid DNA 2 : pAAV[FLEXon]-CMV>LL:rev(EGFP:m*Myh9*[shRNA#1]:m*Actg1*[shRNA#2]):rev(LL):WPRE
Vector size : 6055 bp
Viral genome size: 3436 bp
Virus titer (QC) : 1.06 x 10^{13} GC/mL

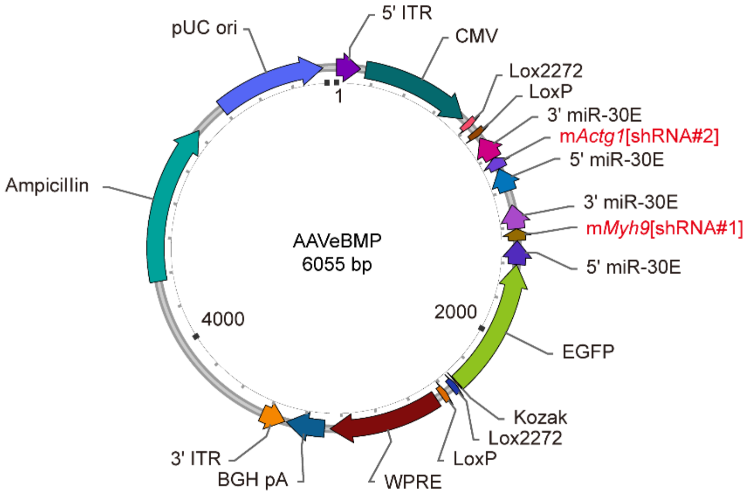

Appendix Figure S7. Schematic representation of plasmid DNA used for producing Cre-dependent adeno-associated virus (AAV) viruses

The AAV-PHP.eB serotype was used to package EGFP-expressing AAV. A miRNA (miR)-based shRNA system was inserted into the pAAV FLEX Cre-on vector to allow for multiple shRNAs to be expressed on one vector. Sites of shRNAs inserted were *Myh9* (shRNA #1), *Actg1* (shRNA #2), and Control (shRNA #1, 2). The titrations of ultra-purified AAV were validated using quantitative PCR.

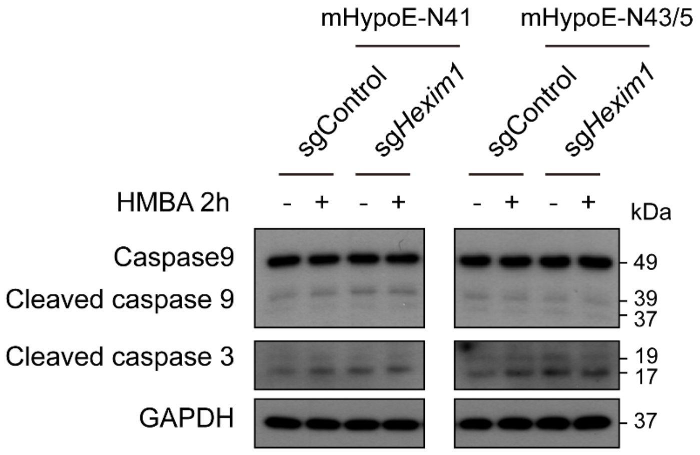

Appendix Figure S8. HMBA or *Hexim1* deletion does not cause apoptosis

Western blot analysis of caspase 9, cleaved caspase 9, and cleaved caspase 3 after HMBA treatment in sgControl or sg*Hexim1* mHypoE-N41 and mHypoE-N43/5 cells. Cells were treated or not with 0.1 mM HMBA for 2 h. The experiment was repeated twice independently with similar results.

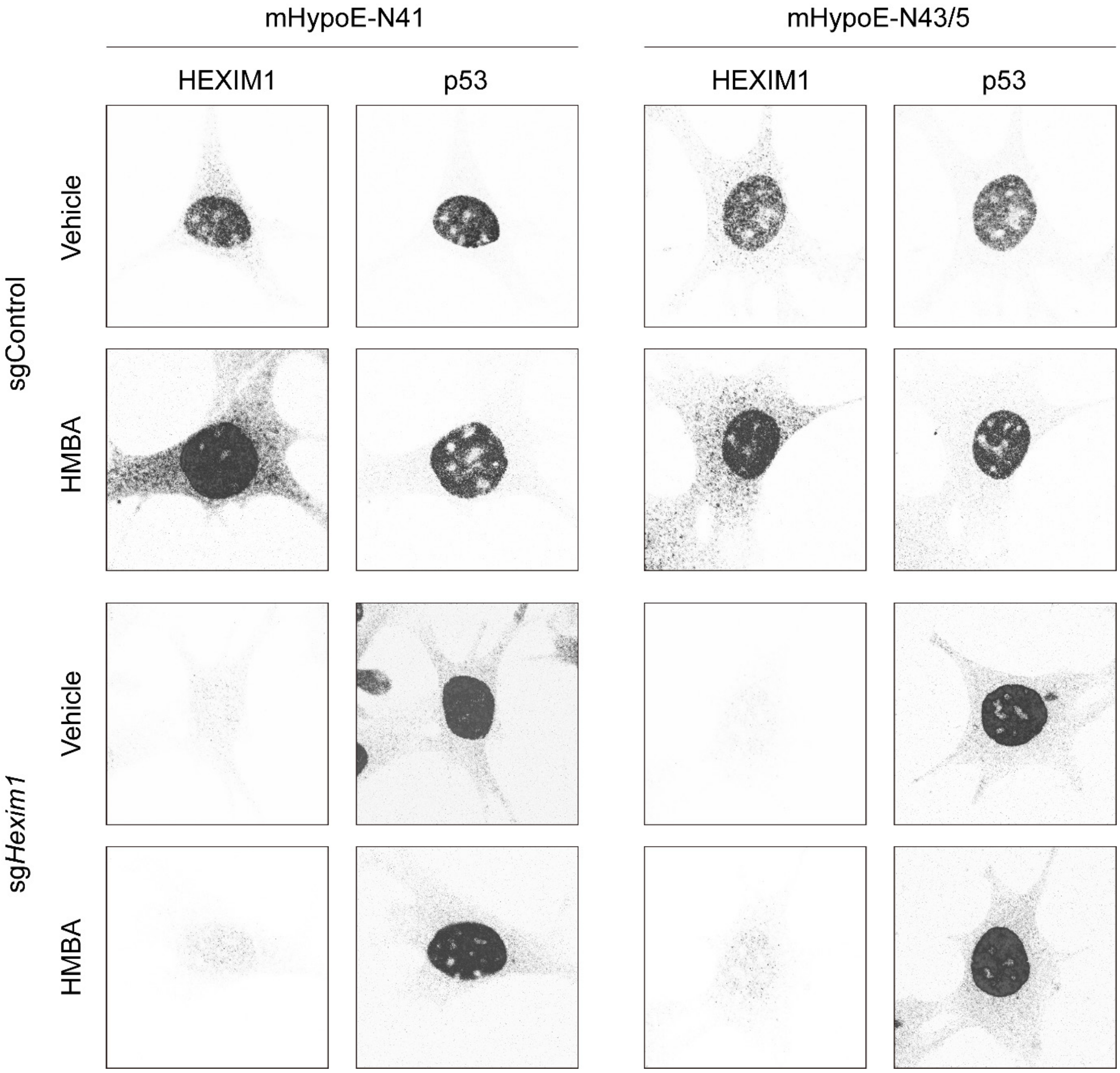

Appendix Figure S9. Localization of HEXIM1 and p53 after HMBA treatment

Immunofluorescence analysis of sgControl and sg*Hexim1* mHypoE-N41 and mHypoE-N43/5 cells after HMBA treatment. Cells were treated with 0.1 mM HMBA for 2 h. Images show the same cells as in Fig 7A and B; black-and-white inverted images are shown to better visualize the localization of HEXIM1 and p53.

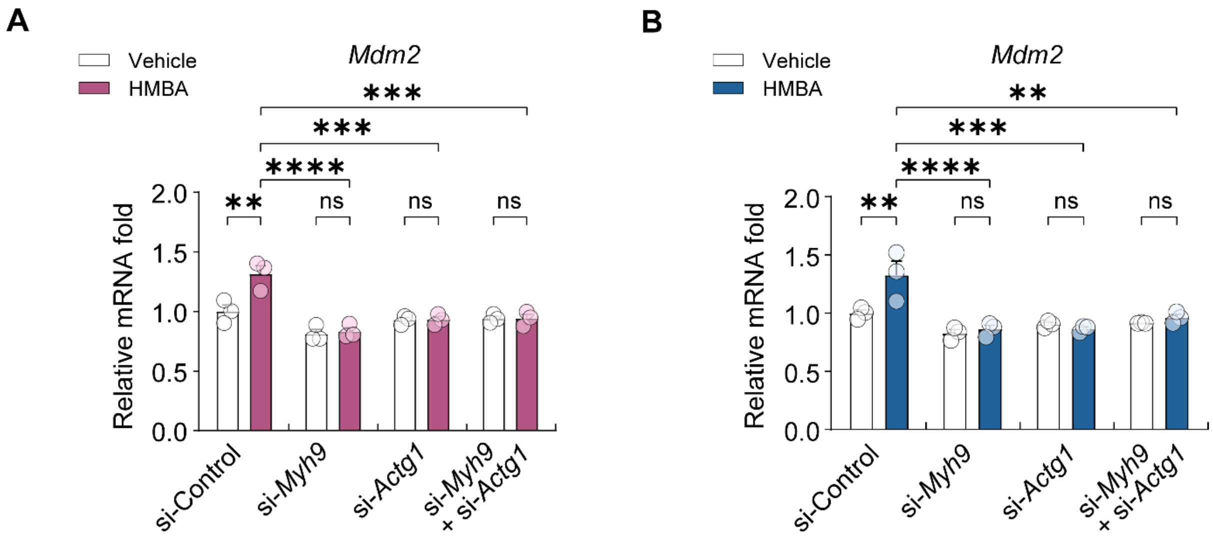

Appendix Figure S10. HMBA induces *Mdm2* expression in the presence of MYH9 and ACTG1

A, B. Relative mRNA levels of *Mdm2* in (**A**) mHypoE-N41 and (**B**) mHypoE-N43/5. $n = 3$ per group.

Data information: After the knockdown of *Myh9*, *Actg1*, or both, cells were treated with 0.1 mM HMBA for 2 h. The datasets in qPCR experiments were comprised of three biological replicates and each biological replicate was an average of three technical replicates. Statistical significance was determined by two-way ANOVA followed by a post hoc Bonferroni test. ***P*<0.01, ****P*<0.001, *****P*<0.0001; ns or unless otherwise stated, no significance. Data are mean ± SEM.

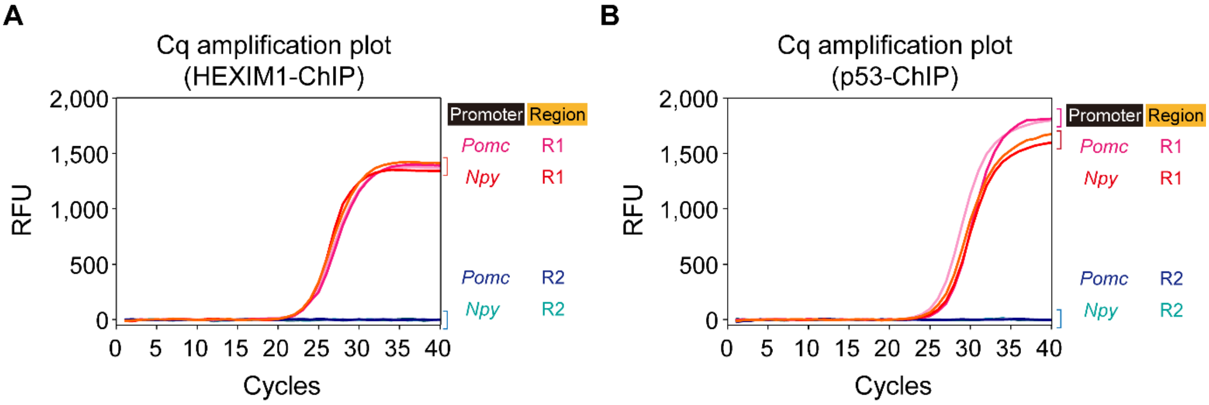

Appendix Figure S11. HEXIM1 and p53 bind to regions 1 but not regions 2 of the *Npy* and *Pomc* promoters

A, B. The quantification cycle (Cq) amplification plots from ChIP-qPCR analysis of the binding of **(A)** HEXIM1 or **(B)** p53 to the *Npy* and *Pomc* promoters. R1, promoter region 1; R2, promoter region 2. Relative fluorescence units (RFU) are used to indicate fluorescence intensity. Details of promoter regions are shown in Fig 7D and E and the primers used for each region are listed in Appendix Table 4.

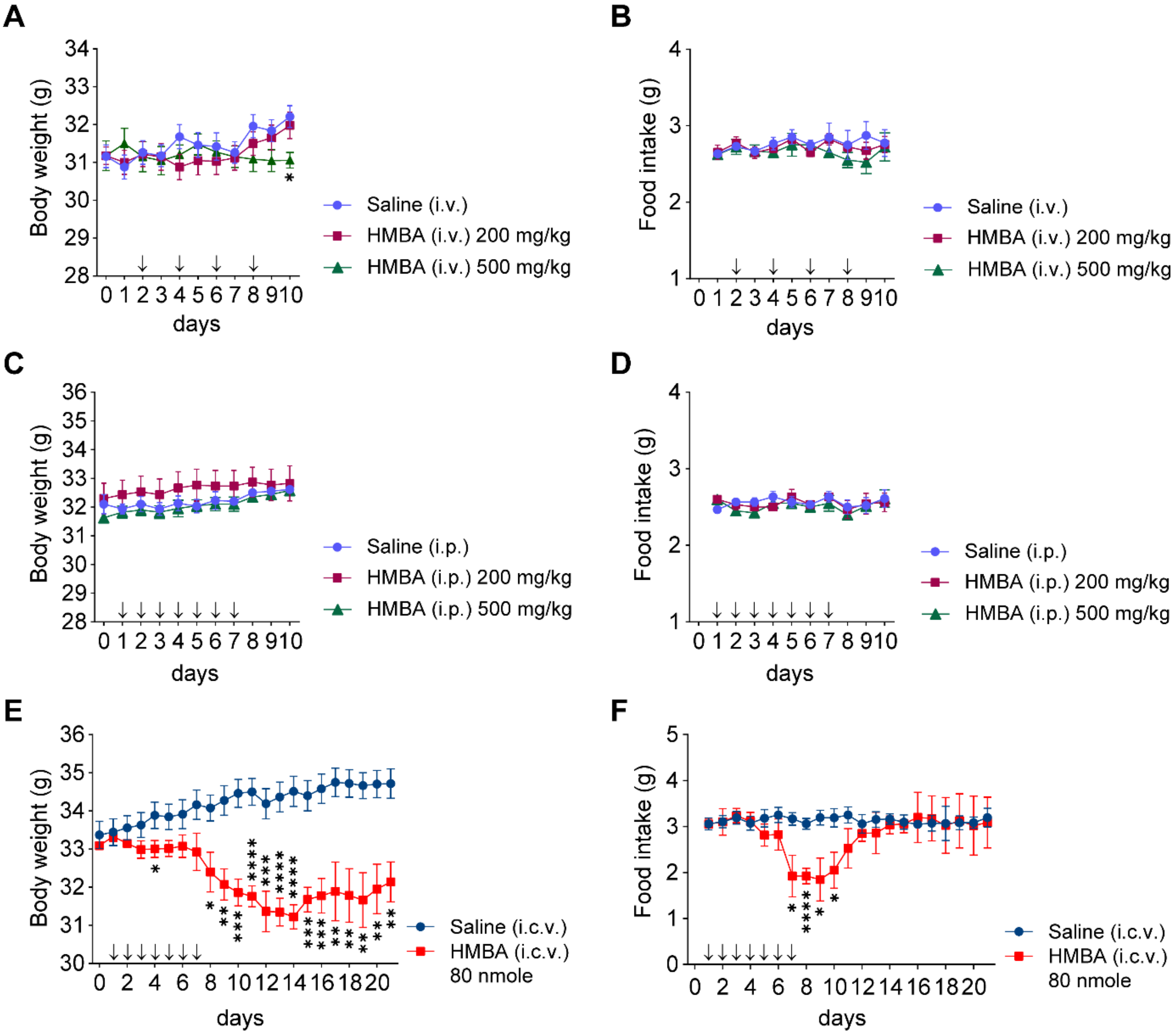

Appendix Figure S12. The effects of low-dose HMBA in male DIO mice

A–B. Changes in (A) daily body weight and (B) food intake in male DIO mice injected i.v. with HMBA. Mice were injected with 200 or 500 mg/kg of HMBA bi-daily. *n* = 5 per group. Arrows represent the days of each injection.

C–D. Changes in (C) daily body weight and (D) food intake in male DIO mice injected i.p.

with HMBA (n = 5). Mice were injected with 200 or 500 mg/kg of HMBA daily for 7 days. *n* = 3, saline and HMBA (i.p.) 200 mg/kg group; *n* = 4, HMBA (i.p.) 500 mg/kg group. Arrows represent the days of each injection.

E–F. Changes in (E) daily body weight and (F) food intake in male DIO mice injected i.c.v. with HMBA. Mice were injected with 80 nmoles of HMBA (2 μl per injection) daily for 7 days. *n* = 8 per group. Arrows represent the days of each injection.

Data information: Data represent different numbers (*n*) of biological replicates. Statistical significance was determined by a two-tailed unpaired Student's *t*-test. *P<0.05, **P<0.01, ***P<0.001, ****P<0.001 vs. Saline. Data are mean ± SEM.

Appendix Table S1. Top 10 small molecules in the Connectivity Map database

Rank	Name	References[1]	Function
1	Oleoylethanolamide	Fu *et al*, 2003	Anorexigenic effect
2	Lithocholic acid	Yousef *et al*, 1988	Induces cholestasis
3	Mestanolone	Harris, 1961	Anabolic steroid
4	Hydroxycholesterol	Asghari *et al*, 2019	Induces obesity
5	Otenzepad	None	None (anti-muscarinic)
6	Valaciclovir	None	None (anti-viral)
7	Artemether	Fu *et al*, 2020	Improves glycolipid metabolism
8	Hexamethylene bisacetamide	None	None (anti-cancer)
9	Guggulsterone	Yang *et al*, 2012	Hypolipidemic effect
10	Geranylgeraniol	Campia *et al*, 2009	Reduces cholesterol synthesis

[1] References and function refer to metabolism-related content only

Appendix Table S2. Precursor-to-fragment ion transitions in LC-MS/MS analysis

Analyte[2]	Precursor ion (m/z)	Fragment ion (m/z)	Collision energy (ev)
HMBA	201.1	159.2	10
NADAH	158.9	99.9	8
DAH	132.0	114.0	10
AmHA	117.1	100.1	20
HMBA–Biotin	385.2	326.1	16

[2] Metabolites were analyzed using positive electrospray ionization mode

Appendix Table S3. Sequences of primers used for qRT-PCR

Gene	Forward primer (5′ → 3′)	Reverse primer (5′ → 3′)
Agrp	CTGCAGACCGAGCAGAAGA	TGCGACTACAGAGGTTCGTG
Actg1	TACCCTATTGAGCACGGCAT	CGCAGCTCGTTGTAGAAGGT
Cart	CGAGAAGAAGTACGGCCAAGTCC	GGAATATGGGAACCGAAGGTGG
Gapdh	ATCACTGCCACCCAGAAGAC	ACACTTGGGGGTAGGAACA
Hexim1	TACAGGTGCTGCTGTTGTCC	CTCCAGGGAGCTCAATTCTG
Hexim2	AGGGTGAATGTCTCGTGCAG	TGCCCACCTACAGACACAAC
Npy	CAGAAAACGCCCCCAGAA	AAAAGTCGGGAGAACAAGTTTCATT
Mdm2	CCGAGTTTCTCTGTGAAGGAGC	GTCTGCTCTCACTCAGCGATGT
Myh9	GGCCCTGCTAGATGAGGAGT	CTTGGGCTTCTGGAACTTGG
Trp53 (*p53*)	CTCCGAAGACTGGATGACTGC	CAACAGATCGTCCATGCAGTG
Pomc	GAACAGCCCCTGACTGAAAA	ACGTTGGGGTACACCTTCAC

Appendix Table S4. Sequences of primers used for ChIP-qPCR

	Gene	Forward primer (5′ → 3′)	Reverse primer (5′ → 3′)
Promoter region 1	*Npy* −605/+3bp	GCGAGGAGCACAGTGGCGTT	AGCAGGGACCGCTTAGATTGCCT
	Pomc −414/−259bp	AATCTGCGACATAACAAATCCCC	AGAACTGGACAGAGGCTTAGCGT
Promoter region 2	*Npy* −118/+22bp	CCCAAGTACAGTGTCTGGTCCCT	TGGGCGCCTCTGCGAGGAAT
	Pomc −162/+82bp	CTTTCCAGGCAGATGTGCCTTG	CAGCTCTCTGGGGACAAAGATGTT

Appendix Table S5. List of antibodies

Antibody	Manufacturer	Catalog Number	Dilution or amount
ACTG1	Santa Cruz Biotechnology	SC-65638	1:1000 for WB<br>1:400 for IHC
Caspase 9/Cleaved Caspase 9	Cell Signaling Technology (CST)	9504	1:1000
Cleaved Caspase 3	CST	9661	1:1000
H3K9Ac	Invitrogen	MA5-33384	2 μg for ChIP
H3K4me3	Abcam	ab8580	2 μg for ChIP
H3K27me3	Abcam	ab6002	2 μg for ChIP
HEXIM1	Bethyl Laboratories	A303-112A	1:1000 for WB<br>1:150 for IF<br>1:400 for IHC<br>2 μg for IP/ChIP
IBA-1	Abcam	ab5076	1:400 for IHC
MDM2	CST	51541	1:1000 for WB
MYH9	CST	3403	1:1000 for WB<br>1:500 for IHC
NeuN	Millipore	MAB377	1:300 for IHC
p53	Santa Cruz Biotechnology	SC-99	1:1000 for WB
	Santa Cruz Biotechnology	SC-126	1:200 for IF
GAPDH	CST	2118	1:10000
GFAP	Dako	Z0334	1:600 for IHC
Goat anti-Rabbit IgG, HRP	Invitrogen	31460	1:10000
Mouse anti-Rabbit IgG, HRP	Abcam	99697	1:5000
Goat anti-Mouse IgG, HRP	CST	7076	1:10000
Donkey anti-mouse IgG, Alexa Fluor 488	Jackson ImmunoResearch Laboratories	715-545-150	1:500
Donkey anti-mouse IgG, Alexa Fluor 647	Jackson ImmunoResearch Laboratories	715-605-151	1:500
Donkey anti-rabbit IgG, Cy3	Jackson ImmunoResearch Laboratories	711-165-152	1:500
Donkey anti-goat IgG, Cy3	Jackson ImmunoResearch Laboratories	705-165-147	1:500

Appendix Table S6. The exact *P* values

Figure	Panel	Statistics analysis	*P* value	Note
1	C	Two-tailed t test	0.0470	Body weight (g), day 5
			0.0225	Body weight (g), day 7
			0.0150	Body weight (g), day 8
			0.0022	Body weight (g), day 9
			0.0018	Body weight (g), day 10
			<0.0001	Body weight (%) on day 10
	D	Two-tailed t test	0.0191	Food intake (g), day 3
			0.0054	Food intake (g), day 4
			<0.0001	Food intake (g), day 8
			0.0107	Food intake (g), day 9
	E	Two-tailed t test	0.0384	Body weight (g), day 7
			0.0221	Body weight (g), day 8
			0.0189	Body weight (g), day 9
			0.0199	Body weight (g), day 10
			0.0074	Body weight (%) on day 10
	F	Two-tailed t test	0.0049	Food intake (g), day 8
			0.0035	Food intake (g), day 9
			0.0203	Food intake (g), day 10
	G	One-way ANOVA	<0.0001	Saline (i.v.) vs HMBA (i.v.) in plasma
			0.0036	Saline (i.p.) vs HMBA (i.p.) in plasma
			0.0369	HMBA (i.v.) vs HMBA (I.p.) in plasma
			<0.0001	Saline (i.v.) vs HMBA (i.v.) in hypothalamus
			<0.0001	Saline (i.p.) vs HMBA (i.p.) in hypothalamus
			0.0001	HMBA (i.v.) vs HMBA (I.p.) in hypothalamus
	H	Two-tailed t test	0.0009	*Npy*
			0.0007	*Agrp*
			0.0235	*Pomc*
			0.0011	*Cart*
			0.0252	*Hexim1*
	I	Two-tailed t test	0.0001	*Npy*
			<0.0001	*Agrp*
			0.0326	*Pomc*
			0.0267	*Cart*
			<0.0001	*Hexim1*
	J	Two-tailed t test	0.0307	fat mass
	K	Two-tailed t test	0.0010	
	L	Two-tailed t test	<0.0001	
	M	ANCOVA	0.0139	Left panel
		Two-tailed t test	0.0161	Right panel
	N	Two-tailed t test	0.0483, 0.0485	Light, total
	O		0.0017, 0.0093, 0.0039	Dark, light, total
	P		0.0348, 0.0365	Dark, total
	Q		0.0027, 0.0128, 0.0057	Dark, light, total
2	B	Two-tailed t test	0.0141	Body weight (g), day 7
			0.0047	Body weight (g), day 8
			0.0002	Body weight (g), day 9
			<0.0001	Body weight (g), day 10 ~ 21
			<0.0001	Body weight (%) on day 21
	C	Two-tailed t test	0.0132	Food intake (g), day 2
			0.0123	Food intake (g), day 3
			0.0006	Food intake (g), day 6
			<0.0001	Food intake (g), day 8 ~ 17
			0.0053	Food intake (g), day 19
			0.0022	Food intake (g), day 20
			<0.0001	Food intake (g), day 21
	D	Two-tailed t test	0.0275	*Npy*
			0.0449	*Agrp*
			0.0004	*Pomc*
			0.0121	*Cart*
			0.0001	*Hexim1*
	E	Two-tailed t test	0.0002	fat mass
			0.0005	lean mass

	F	Two-tailed t test	0.0311	
	G	Two-tailed t test	0.0081	
	H	ANCOVA	0.0011	Left panel
		Two-tailed t test	<0.0001	Right panel
	J	Two-tailed t test	0.0153, 0.0025, 0.0205	Dark, light, total
	K		0.0087, 0.0043, 0.0178	Dark, light, total
	L		0.0001	All
	O	Two-way ANOVA	<0.0001, 0.0091	15 ~ 90 min, 120 min
		Two-tailed t test	0.0041	AUC
	P	Two-way ANOVA	0.0364, <0.0001, 0.0041, 0.0022, <0.0001	0, 15, 30, 60, 90 ~ 10 min
		Two-tailed t test	0.0110	AUC
	Q	Two-tailed t test	0.0052	
	R	Two-tailed t test	0.0125, 0.0097	Total, in SFA, in PUFA
3	F	Two-way ANOVA	<0.0001	2 h
			0.0005	4 h
			0.0003	8 h
			0.0004	24 h
	G	Two-way ANOVA	0.0018	0.5 h
			0.0065	1 h
			<0.0001	2 h
			<0.0001	4 h
			0.0049	8 h
			0.0003	24 h
	H	Two-way ANOVA	<0.0001	All
	I	Two-way ANOVA	0.0388, <0.0001	10^{-2}, 10^{-1} in 1 h
			0.0003, <0.0001	10^{-2}, 10^{-1} in 2 h
	K	Two-way ANOVA	0.0123	0.5 h
			<0.0001	1 h
			<0.0001	2 h
			<0.0001	4 h
			<0.0001	8 h
			<0.0001	24 h
	L	Two-way ANOVA	<0.0001	0.5 h
			<0.0001	1 h
			<0.0001	2 h
			0.0002	4 h
	M	Two-way ANOVA	0.0148	Vehicle vs HMBA in si-Control
			>0.9999	Vehicle vs HMBA in si-*Hexim1*
			0.0038	Vehicle (si-Control vs si-*Hexim1*)
			>0.9999	HMBA (si-Control vs si-*Hexim1*)
	N		<0.0001	Vehicle vs HMBA in si-Control
			0.9996	Vehicle vs HMBA in si-*Hexim1*
			0.1300	Vehicle (si-Control vs si-*Hexim1*)
			<0.0001	HMBA (si-Control vs si-*Hexim1*)
	O		0.0001	Vehicle vs HMBA in sgControl
			>0.9999	Vehicle vs HMBA in sg*Hexim1*
			<0.0001	Vehicle (sgControl vs sg*Hexim1*)
			<0.0001	HMBA (sgControl vs sg*Hexim1*)
	P		<0.0001	Vehicle vs HMBA in sgControl
			0.6734	Vehicle vs HMBA in sg*Hexim1*
			0.0361	Vehicle (sgControl vs sg*Hexim1*)
			<0.0001	HMBA (sgControl vs sg*Hexim1*)
4	I	Two-way ANOVA	<0.0001	Vehicle vs HMBA in si-Control
			0.0566	Vehicle vs HMBA in si-*Myh9*
			0.0009	HMBA (si-Control vs si-*Myh9*)
	J		0.0013	Vehicle vs HMBA in si-Control
			0.6083	Vehicle vs HMBA in si-*Myh9*
			0.0034	HMBA (si-Control vs si-*Myh9*)
	K		<0.0001	Vehicle vs HMBA in si-Control
			0.1362	Vehicle vs HMBA in si-*Actg1*
			0.0002	HMBA (si-Control vs si-*Actg1*)
	L		0.0005	Vehicle vs HMBA in si-Control
			0.9044	Vehicle vs HMBA in si-*Actg1*
			0.0011	HMBA (si-Control vs si-*Actg1*)
	M	Two-way ANOVA	<0.0001	Vehicle vs HMBA in si-Control
			0.0079	Vehicle vs HMBA in si-*Myh9*

			0.0003	HMBA (si-Control vs si-*Myh9*)
	N		0.0002	Vehicle vs HMBA in si-Control
			0.6627	Vehicle vs HMBA in si-*Myh9*
			0.0026	HMBA (si-Control vs si-*Myh9*)
	O		<0.0001	Vehicle vs HMBA in si-Control
			0.0033	Vehicle vs HMBA in si-*Actg1*
			<0.0001	HMBA (si-Control vs si-*Actg1*)
	P		<0.0001	Vehicle vs HMBA in si-Control
			0.0738	Vehicle vs HMBA in si-*Actg1*
			<0.0001	HMBA (si-Control vs si-*Actg1*)
5	A	Two-tailed t test	<0.0001	
	B		<0.0001	
	C		0.0157	
	D		<0.0001	
	F	Two-way ANOVA	<0.0001	All
	G		<0.0001	All
	H	Two-tailed t test	0.0092	
	I	Two-tailed t test	0.0004	
	J	One-way ANOVA	<0.0001	*Npy*
			<0.0001	*Pomc*
	K		<0.0001	*Npy*
			<0.0001	*Pomc*
	L	Two-way ANOVA	0.0358	shControl (Saline vs HMBA), day 7
			0.0193	shControl (Saline vs HMBA), day 8
			0.0059	shControl (Saline vs HMBA), day 9
			0.0017	shControl (Saline vs HMBA), day 10
			0.0242	HMBA (shControl vs sh*Myh9*+sh*Actg1*), day 6
			0.0239	HMBA (shControl vs sh*Myh9*+sh*Actg1*), day 7
			0.0200	HMBA (shControl vs sh*Myh9*+sh*Actg1*), day 8
			0.0138	HMBA (shControl vs sh*Myh9*+sh*Actg1*), day 9
			0.0087	HMBA (shControl vs sh*Myh9*+sh*Actg1*), day 10
			0.0038	(%), shControl (Saline vs HMBA)
			0.9430	(%), shMyh9+shActg1 (Saline vs HMBA)
			0.9856	(%), Saline (shControl vs sh*Myh9*+sh*Actg1*)
			0.0097	(%), HMBA (shControl vs sh*Myh9*+sh*Actg1*)
	M	Two-way ANOVA	0.0159	shControl (Saline vs HMBA), day 4
			0.0003	shControl (Saline vs HMBA), day 5
			0.0036	HMBA (shControl vs sh*Myh9*+sh*Actg1*), day 1
			0.0575	HMBA (shControl vs sh*Myh9*+sh*Actg1*), day 2
			0.0582	HMBA (shControl vs sh*Myh9*+sh*Actg1*), day 3
			0.0105	HMBA (shControl vs sh*Myh9*+sh*Actg1*), day 5
	N	Two-way ANOVA	0.0383	shControl (Saline vs HMBA), day 9
			0.0186	shControl (Saline vs HMBA), day 10
			0.0329	HMBA (shControl vs sh*Myh9*+sh*Actg1*), day 8
			0.0197	HMBA (shControl vs sh*Myh9*+sh*Actg1*), day 9
			0.0104	HMBA (shControl vs sh*Myh9*+sh*Actg1*), day 10
			0.0038	(%), shControl (Saline vs HMBA)
			0.9983	(%), shMyh9+shActg1 (Saline vs HMBA)
			0.9873	(%), Saline (shControl vs sh*Myh9*+sh*Actg1*)
			0.0046	(%), HMBA (shControl vs sh*Myh9*+sh*Actg1*)
	O	Two-way ANOVA	0.0015	shControl (Saline vs HMBA), day 1
			0.0009	shControl (Saline vs HMBA), day 2
			0.0003	shControl (Saline vs HMBA), day 3
			<0.0001	shControl (Saline vs HMBA), day 4 ~ day 7
			0.0031	shControl (Saline vs HMBA), day 8
			0.0440	HMBA (shControl vs sh*Myh9*+sh*Actg1*), day 3
			0.0033	HMBA (shControl vs sh*Myh9*+sh*Actg1*), day 4
			<0.0001	HMBA (shControl vs sh*Myh9*+sh*Actg1*), day 5
			0.0017	HMBA (shControl vs sh*Myh9*+sh*Actg1*), day 6
			0.0200	HMBA (shControl vs sh*Myh9*+sh*Actg1*), day 7
6	B	Two-way ANOVA	0.0007	Vehicle vs HMBA in si-Control
			0.1394	Vehicle vs HMBA in si-*p53*
			0.5516	Vehicle (si-Control vs si-*p53*)
			0.0003	HMBA (si-Control vs si-*p53*)
	D	Two-way ANOVA	<0.0001	Vehicle vs HMBA in si-Control
			0.7118	Vehicle vs HMBA in si-*p53*
			0.1406	Vehicle (si-Control vs si-*p53*)

			<0.0001	HMBA (si-Control vs si-*p53*)
	F	Two-way ANOVA	0.0433	0.5 h in *Hexim1*
			<0.0001	1 h in *Hexim1*
			<0.0001	2 h in *Hexim1*
	I	Two-way ANOVA	<0.0001	0.5 h in *Hexim1*
			<0.0001	1 h in *Hexim1*
			<0.0001	2 h in *Hexim1*
7	A	Two-way ANOVA	<0.0001, 0.0401	HEXIM1
			<0.0001	p53 (all)
	B	Two-way ANOVA	<0.0001, 0.0203	HEXIM1
			<0.0001	p53 (all)
	E	Two-way ANOVA	<0.0001	p53
			<0.0001	HEXIM1
			0.0428	H3K9Ac
			0.0339	H3K4me3
			>0.9999	H3K27me3
	F	Two-way ANOVA	<0.0001	p53
			<0.0001	HEXIM1
			<0.0001	H3K9Ac
			0.9876	H3K4me3
			0.8217	H3K27me3
EV 1	A	Two-tailed t test	0.0555	Eating
	C	One-way ANOVA	0.9095	Pre vs post in HMBA
			<0.0001	Pre vs post in LiCl
			<0.0001	Post (HMBA vs LiCl)
	D	Two-tailed t test	0.0464	Eating
	F	One-way ANOVA	0.9977	Pre vs post in HMBA
			<0.0001	Pre vs post in LiCl
			<0.0001	Post (HMBA vs LiCl)
	G	Two-tailed t test	0.0004	Eating
	I	One-way ANOVA	>0.9999	Pre vs post in HMBA
			<0.0001	Pre vs post in LiCl
			0.0003	Post (HMBA vs LiCl)
EV 3	A	Two-way ANOVA	<0.0001	All in *Myh9* panel
			0.0002	Vehicle (si-Control vs si-*Actg1*)
			0.0002	HMBA (si-Control vs si-*Actg1*)
	B	Two-way ANOVA	<0.0001	All
	C	Two-way ANOVA	<0.0001	All in *Myh9* and *Actg1* panel
			<0.0001	si-Control (Vehicle vs HMBA) in *Hexim1*
			0.3985	si-*Myh9*+si-*Actg1* (Vehicle vs HMBA) in *Hexim1*
			<0.0001	HMBA (si-control vs si-*Myh9*+si-*Actg1*) in *Hexim1*
	E	Two-way ANOVA	0.0017	si-Control (Vehicle vs HMBA)
			0.9776	si-*Myh9*+si-*Actg1* (Vehicle vs HMBA)
			0.0029	HMBA (si-control vs si-*Myh9*+si-*Actg1*)
	F	Two-way ANOVA	0.0001	Vehicle (si-control vs si-*Myh9*+si-*Actg1*) in *Myh9*
			<0.0001	HMBA (si-control vs si-*Myh9*+si-*Actg1*) in *Myh9*
			<0.0001	All in *Actg1* panel
			<0.0001	si-Control (Vehicle vs HMBA) in *Hexim1*
			0.2983	si-*Myh9*+si-*Actg1* (Vehicle vs HMBA) in *Hexim1*
			<0.0001	HMBA (si-control vs si-*Myh9*+si-*Actg1*) in *Hexim1*
	H	Two-way ANOVA	<0.0001	si-Control (Vehicle vs HMBA)
			0.8015	si-*Myh9*+si-*Actg1* (Vehicle vs HMBA)
			<0.0001	HMBA (si-control vs si-*Myh9*+si-*Actg1*)
EV 4	C	Two-tailed t test	0.3925	
	D	Two-tailed t test	0.4754	
EV 5	C	Two-way ANOVA	<0.0001	Vehicle vs HMBA
			<0.0001	Vehicle+LMB vs HMBA+LMB
			0.9701	Vehicle vs Vehicle+LMB
			<0.0001	HMBA vs HMBA+LMB
	D	Two-way ANOVA	<0.0001	Vehicle vs HMBA
			<0.0001	Vehicle+LMB vs HMBA+LMB
			>0.9999	Vehicle vs Vehicle+LMB
			0.0239	HMBA vs HMBA+LMB

References

Asghari A, Ishikawa T, Hiramitsu S, Lee WR, Umetani J, Bui L, Korach KS, Umetani M (2019) 27-Hydroxycholesterol promotes adiposity and mimics adipogenic diet-induced inflammatory signaling. *Endocrinology* 160: 2485-2494

Campia I, Lussiana C, Pescarmona G, Ghigo D, Bosia A, Riganti C (2009) Geranylgeraniol prevents the cytotoxic effects of mevastatin in THP-1 cells, without decreasing the beneficial effects on cholesterol synthesis. *Br J Pharmacol* 158: 1777-1786

Fu J, Gaetani S, Oveisi F, Lo Verme J, Serrano A, Rodriguez De Fonseca F, Rosengarth A, Luecke H, Di Giacomo B, Tarzia G, Piomelli D (2003) Oleylethanolamide regulates feeding and body weight through activation of the nuclear receptor PPAR-alpha. *Nature* 425: 90-93

Fu W, Ma Y, Li L, Liu J, Fu L, Guo Y, Zhang Z, Li J, Jiang H (2020) Artemether regulates metaflammation to improve glycolipid metabolism in db/db mice. *Diabetes Metab Syndr Obes* 13: 1703-1713

Harris LH (1961) The protein anabolic action of mestanolone. *J Clin Endocrinol Metab* 21: 1099-1105

Yang D, Yang J, Shi D, Xiao D, Chen YT, Black C, Deng R, Yan B (2012) Hypolipidemic agent Z-guggulsterone: metabolism interplays with induction of carboxylesterase and bile salt export pump. *J Lipid Res* 53: 529-539

Yousef IM, Tuchweber B, Morazain R, Kugelmass R, Gauvin M, Roy CC, Weber AM (1988) Cholesterol synthesis in the pathogenesis of lithocholic acid-induced cholestasis. *Lipids* 23: 230-233
