# Supplementary material for: HMBA ameliorates obesity by MYH9‐ and ACTG1‐dependent regulation of hypothalamic neuropeptides
Source: EMBO Mol Med. 2023 Nov 20;15(12):e18024. doi: 10.15252/emmm.202318024 (PMC10701615; doi:10.15252/emmm.202318024)
Supplement: Supplementary file 2 — Expanded View Figures PDF [file EMMM-15-e18024-s003.pdf]

Expanded View Figures

Figure EV1. HMBA does not induce sickness behaviors in DIO mice.

A Behaviors of male DIO mice injected i.v. with HMBA. *N* = 4 per group.
B Locomotion (position distribution, distance traveled, and time of immobility) of i.v. injected group. Position distribution images were taken by integrating four individual mouse cages. *n* = 4 per group.
C Conditioned taste aversion test of i.v. injected group. *n* = 5 per group.
D Behaviors of male DIO mice injected i.p. with HMBA. *n* = 4 per group.
E Locomotion (position distribution, distance traveled, and time of immobility) of i.p. injected group. Position distribution images were taken by integrating four individual mouse cages. *n* = 4 per group.
F Conditioned taste aversion test of i.p. injected group. *n* = 5 per group.
G Behaviors of male DIO mice injected intracerebroventricular (i.c.v.) with HMBA. *n* = 4 per group.
H Locomotion (position distribution, distance traveled, and time of immobility) of i.c.v. injected group. Position distribution images were taken by integrating four individual mouse cages. *n* = 4 per group.
I Conditioned taste aversion test of i.c.v. injected group. *n* = 5 per group.

Data information: Data represent different numbers (*n*) of biological replicates. Data are represented in box and whisker plots where the central band denotes the median value, box contains interquartile ranges, while whiskers mark minimum and maximum values in panels (C), (F), and (I). The behaviors of mice were monitored immediately after the last dosing of HMBA i.v., i.p., or i.c.v. in LABORAS cages for 24 h. Mice received lithium chloride (LiCl, 0.05 M, 2% body weight; i.p.) to induce malaise, or received HMBA (i.v., i.p., or i.c.v.). Statistical significance was determined by a two-tailed unpaired Student's *t*-test in panels (A), (B), (D), (E), (G), and (H), and one-way ANOVA followed by a *post hoc* Tukey test in panels (C), (F), and (I). *$P < 0.05$, ***$P < 0.001$, ****$P < 0.0001$. Data are mean ± SEM. The exact *P*-values are reported in Appendix Table S6.

 © 2023 The Authors

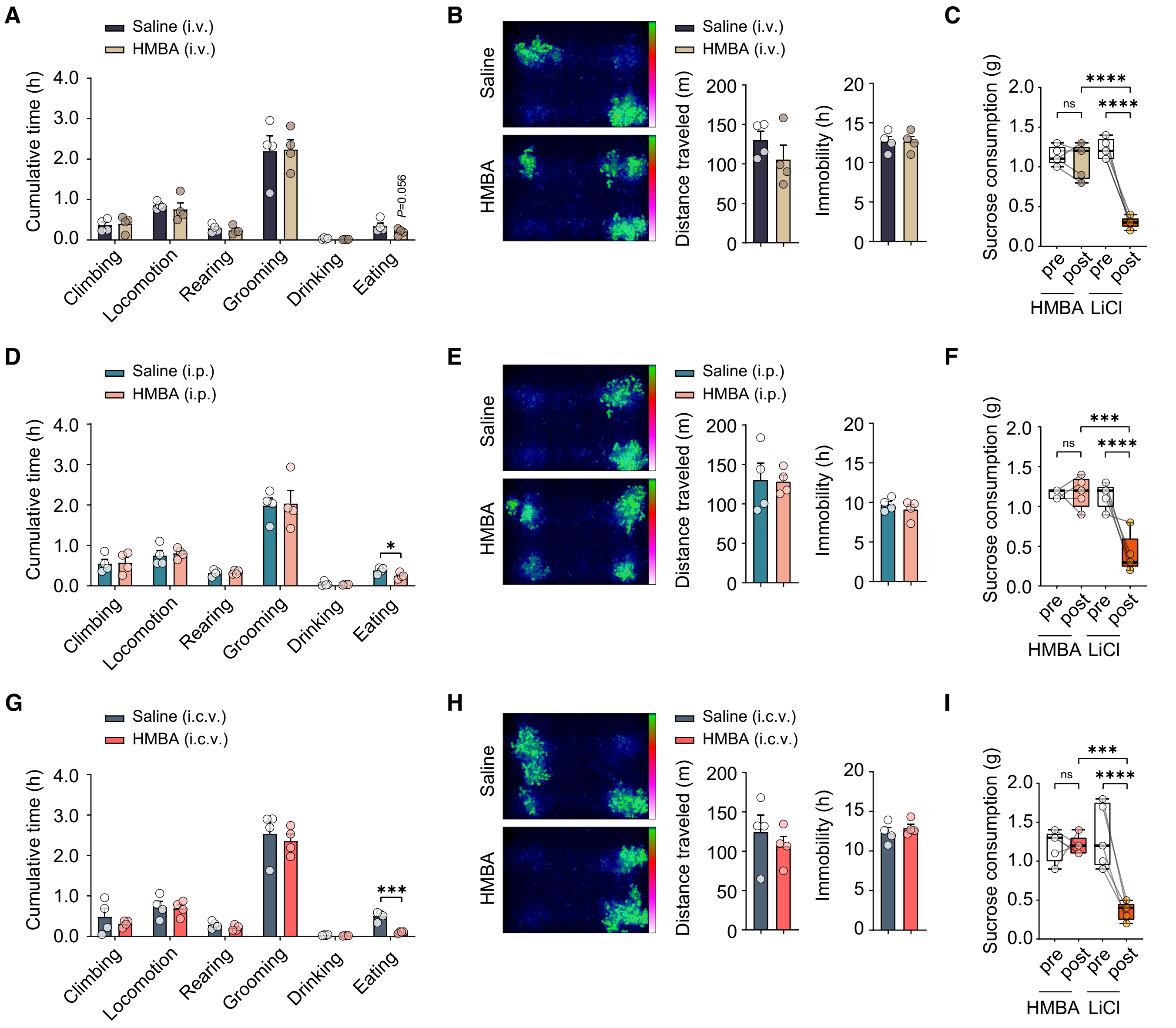

Figure EV1.

Figure EV2. HMBA complexes with MYH9 and ACTG1.

A, B The most energetically favored poses of HMBA in the potential binding pockets of (A) MYH9 and (B) ACTG1 were predicted by AutoDock v4.2.6 and were depicted in sphere view by Protein Imager. aa, amino acid sequence.

C, D Ligplot (two-dimensional docking models) analysis of (C) the MYH9–HMBA complex and (D) ACTG1–HMBA complex. HMBA is shown in yellow. Hydrogen bonds are indicated by dashed green lines with the distance (Å) from the donor atom to the acceptor atom indicated. Hydrophobic interactions are shown by red spokes radiating toward the ligand atoms they contact, and blue spokes indicate hydrophobic interactions with potential hydrogen bonds. C, N, and O atoms are shown in black, blue, and red, respectively.

© 2023 The Authors

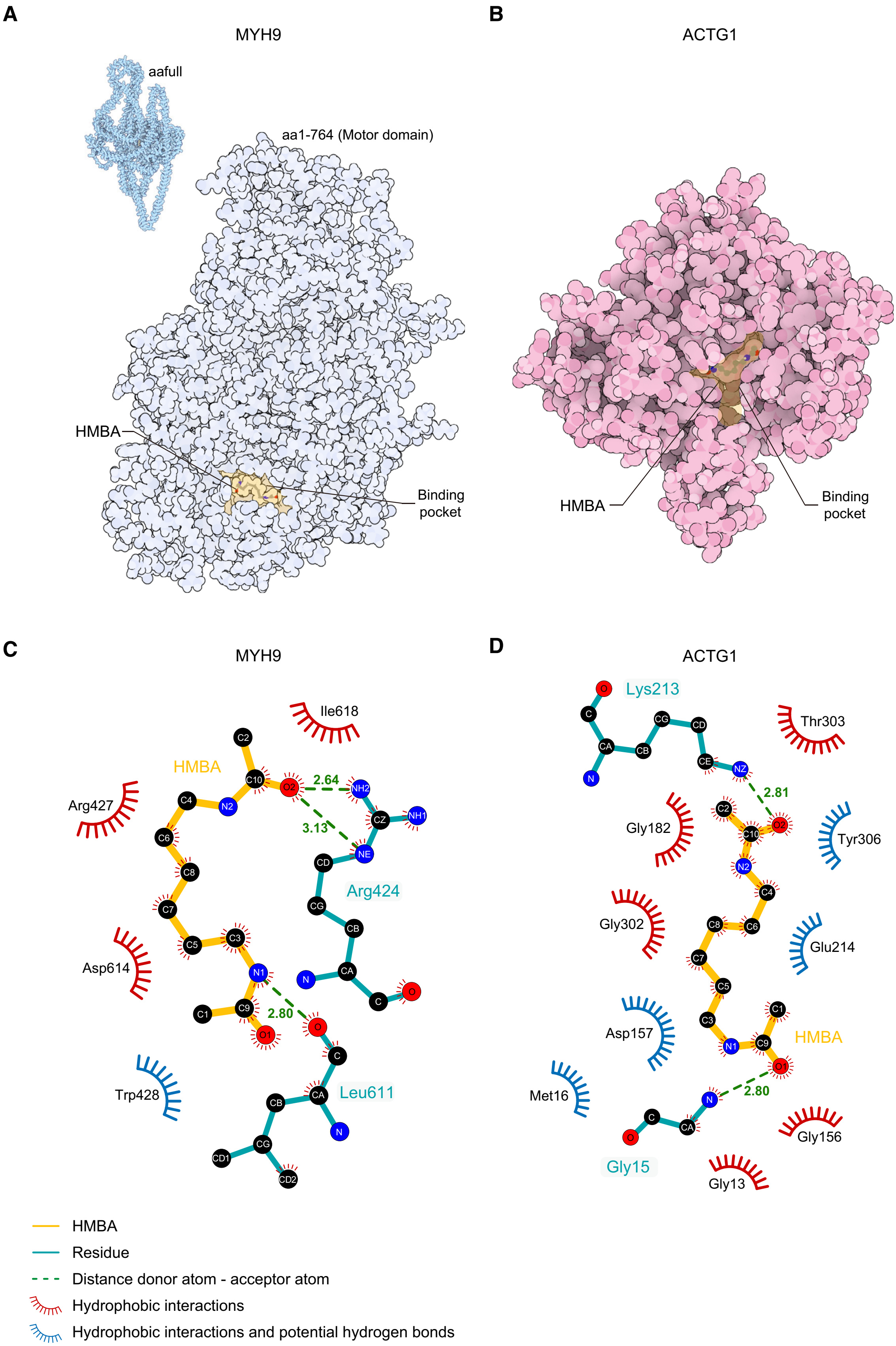

Figure EV2.

© 2023 The Authors

Figure EV3. MYH9 and ACTG1 are required for the regulation of neuropeptide expression by HMBA.

A, B Knockdown efficiency of *Myh9* (si-*Myh9*) and *Actg1* (si-*Actg1*) assessed from relative mRNA levels in (A) mHypoE-N41 (related to Fig 4I–L) and (B) mHypoE-N43/5 (related to Fig 4M–P). n = 3 per group.

C Relative mRNA levels of *Myh9*, *Actg1*, and *Hexim1* in *Myh9* and *Actg1* double-knockdown (si-*Myh9* + si-*Actg1*) mHypoE-N41. n = 3 per group.

D Western blot analysis of *Myh9* and *Actg1* double-knockdown mHypoE-N41.

E Relative mRNA levels of *Npy* in *Myh9* and *Actg1* double-knockdown mHypoE-N41. n = 3 per group.

F Relative mRNA levels of *Myh9*, *Actg1*, and *Hexim1* in *Myh9* and *Actg1* double-knockdown mHypoE-N43/5. n = 3 per group.

G Western blot analysis of *Myh9* and *Actg1* double-knockdown mHypoE-N43/5.

H Relative mRNA levels of *Pomc* in *Myh9* and *Actg1* double-knockdown mHypoE-N43/5. n = 3 per group.

Data information: After single or double knockdown, cells were treated or not with 0.1 mM HMBA for 2 h. The datasets in qPCR experiments were comprised of three biological replicates and each biological replicate was an average of three technical replicates. Western blots were repeated twice independently with similar results. Statistical significance was determined by two-way ANOVA followed by a *post hoc* Tukey test. $^{**}P < 0.01$, $^{***}P < 0.001$, $^{****}P < 0.0001$; ns or unless otherwise stated, no significance. Data are mean ± SEM. The exact *P*-values are reported in Appendix Table S6.

© 2023 The Authors

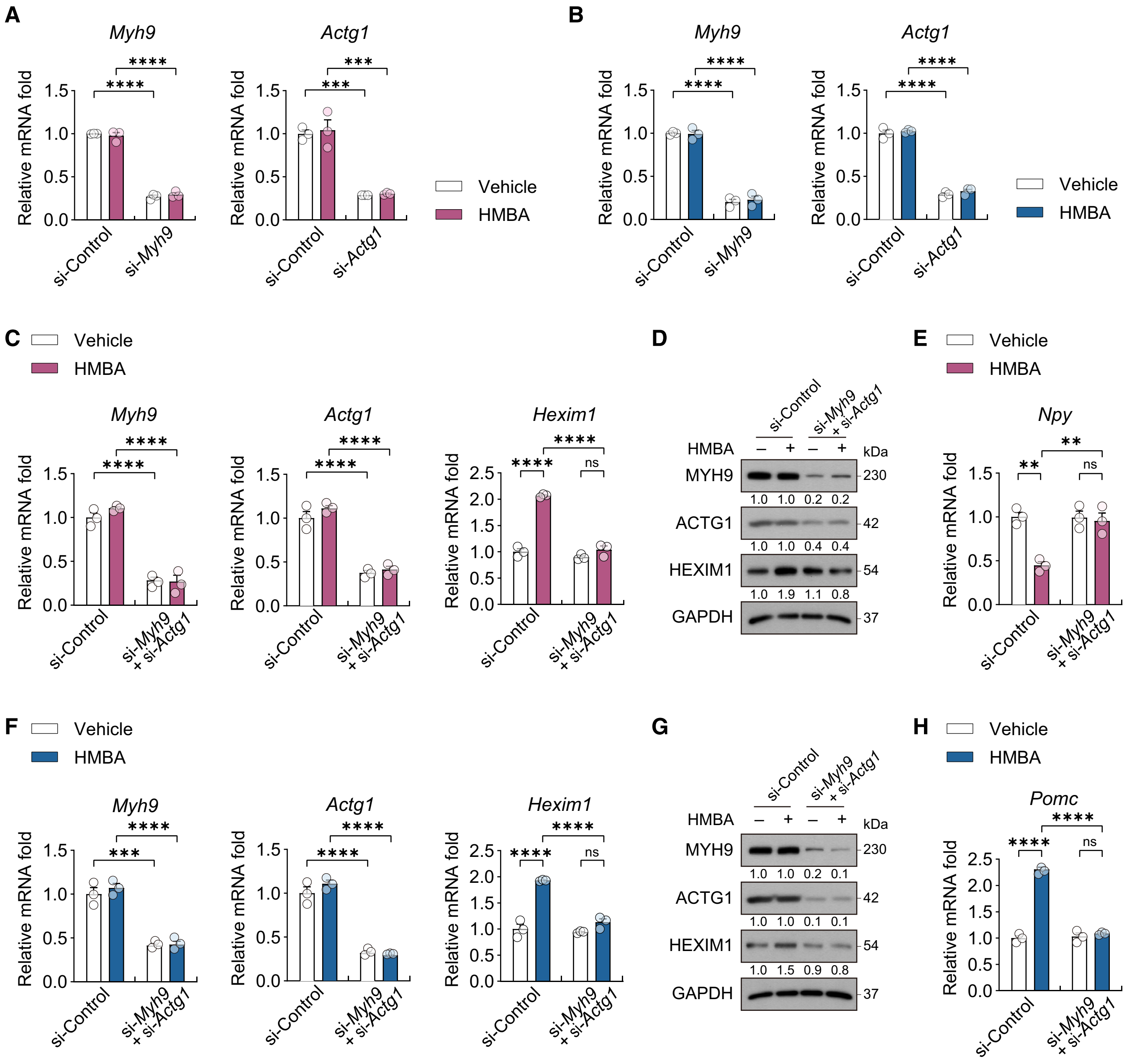

Figure EV3.

 © 2023 The Authors

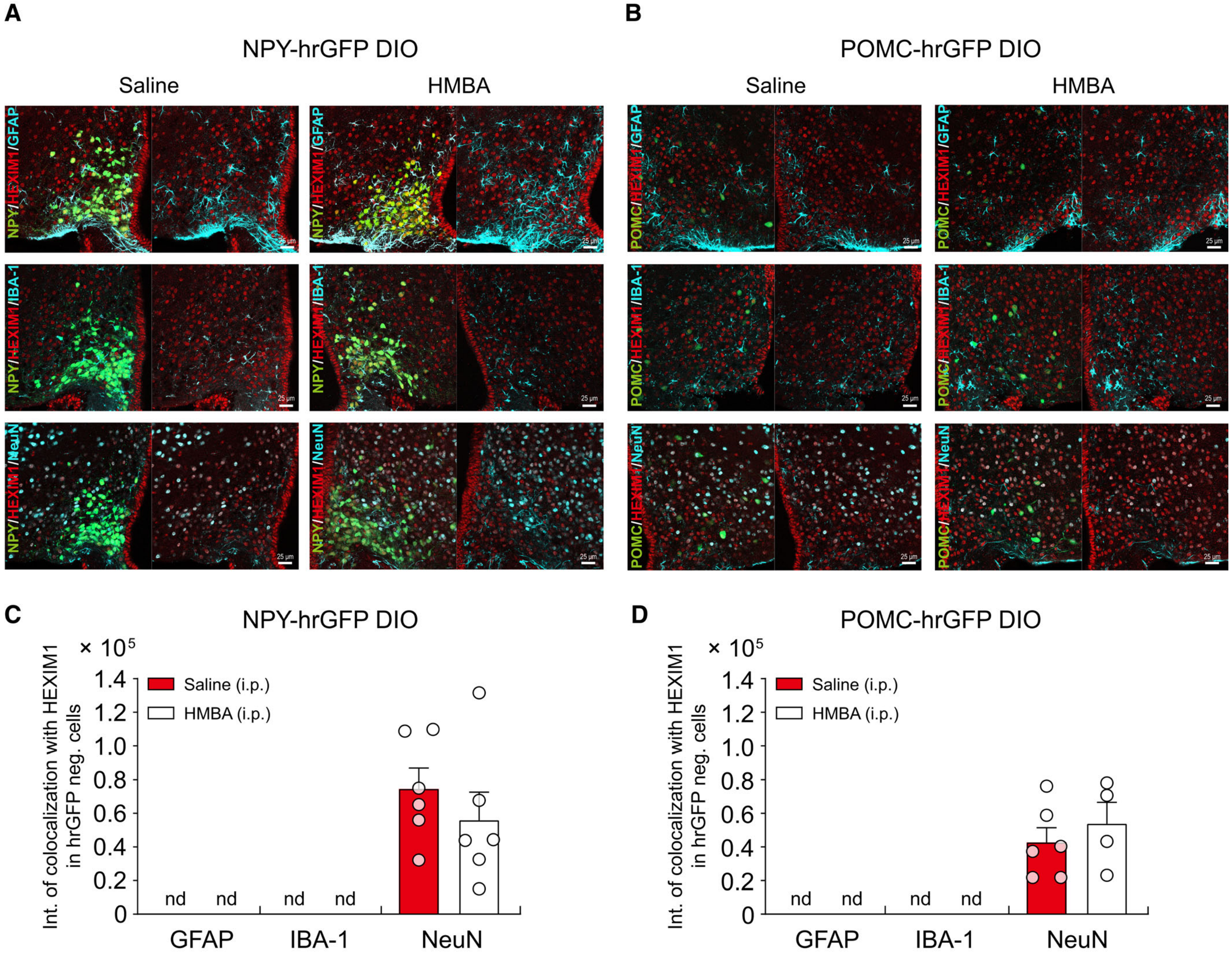

Figure EV4. HMBA specifically induces HEXIM1 expression in NPY and POMC neurons rather than other cells in the ARC.

A, B Immunohistochemistry analysis for the fluorescence intensity of GFAP (astrocyte), IBA-1 (microglia), NeuN (mature neuron), and HEXIM1 in the ARC of (A) NPY-hrGFP DIO mice and (B) POMC-hrGFP DIO mice after HMBA treatment. Scale bars, 25 μm.

C, D The fluorescence intensity of HEXIM1 (Cy3) which co-localized with hrGFP-negative cells in the ARC of (C) NPY-hrGFP DIO mice and (D) POMC-hrGFP DIO mice after HMBA treatment. n = 6 per group (saline- or HMBA-injected NPY-hrGFP DIO mice, and saline-injected POMC-hrGFP DIO mice): three brain slices were obtained from each of the two mice per group; n = 4: two brain slices were obtained from each of the two HMBA-injected POMC-hrGFP DIO mice.

Data information: NPY-hrGFP and POMC-hrGFP DIO mice fed an HFD for 4 weeks were injected with HMBA (i.p., 1,000 mg/kg), and then brains were harvested 4 h after injection. Statistical significance was determined by a two-tailed unpaired Student's t-test. nd, not detected. Data are mean ± SEM. The exact P-values are reported in Appendix Table S6.

© 2023 The Authors

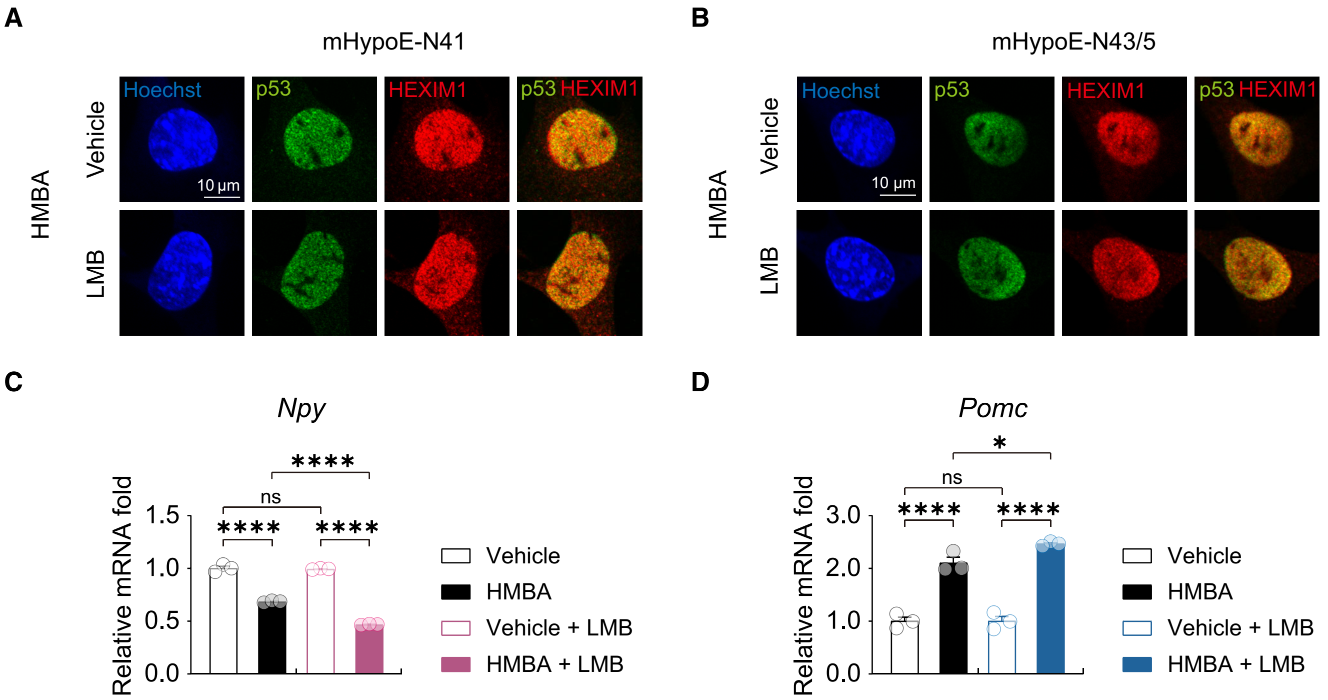

Figure EV5. Inhibition of nuclear export of both HEXIM1 and p53 by leptomycin B effectively modulates neuropeptide expression.

A, B Immunofluorescence analysis of HEXIM1 and p53 after HMBA and leptomycin B (LMB) treatment in (A) mHypoE-N41 and (B) mHypoE-N43/5.

C Relative mRNA levels of *Npy* in mHypoE-N41. $n = 3$ per group.

D Relative mRNA levels of *Pomc* in mHypoE-N43/5. $n = 3$ per group.

Data information: Cells were pre-treated with 0.1 mM HMBA for 1 h and then treated with 50 ng/ml LMB. Scale bars, 10 µm. The datasets in qPCR experiments were comprised of three biological replicates and each biological replicate was an average of three technical replicates. Statistical significance was determined by two-way ANOVA followed by a *post hoc* Tukey test. $*P < 0.05$, $**P < 0.01$, $***P < 0.001$, $****P < 0.0001$; ns or unless otherwise stated, no significance. Data are mean ± SEM. The exact *P*-values are reported in Appendix Table S6.

 © 2023 The Authors
