# Supplementary material for: HMBA ameliorates obesity by MYH9‐ and ACTG1‐dependent regulation of hypothalamic neuropeptides
Source: EMBO Mol Med. 2023 Nov 20;15(12):e18024. doi: 10.15252/emmm.202318024 (PMC10701615; doi:10.15252/emmm.202318024)
Supplement: Supplementary file 3 — PDF+ [file EMMM-15-e18024-s006.pdf]

Article

SOURCE DATA | TRANSPARENT PROCESS | OPEN ACCESS

EMBO Molecular Medicine

HMBA ameliorates obesity by MYH9- and ACTG1-dependent regulation of hypothalamic neuropeptides

Seokjae Park[1,2], Sungjoon Oh[1,2], Nayoun Kim[1] & Eun-Kyoung Kim[1,2,*]

Abstract

The global epidemic of obesity remains a daunting problem. Here, we report hexamethylene bisacetamide (HMBA) as a potent anti-obesity compound. Peripheral and central administration of HMBA to diet-induced obese mice regulated the expression of hypothalamic neuropeptides critical for energy balance, leading to beneficial metabolic effects such as anorexia and weight loss. We found that HMBA bound to MYH9 and ACTG1, which were required for the anti-obesity effects of HMBA in both NPY-expressing and POMC-expressing neurons. The binding of HMBA to MYH9 and ACTG1 elevated the expression of HEXIM1 and enhanced its interaction with MDM2, resulting in the dissociation of the HEXIM1–p53 complex in hypothalamic cells. Subsequently, the free HEXIM1 and p53 translocate to the nucleus, where they downregulated the transcription of orexigenic NPY, but p53 and acetylated histone 3 upregulated that of anorexigenic POMC. Our study points to a previously unappreciated efficacy of HMBA and reveals its mechanism of action in metabolic regulation, which may propose HMBA as a potential therapeutic strategy for obesity.

Keywords ACTG1; anti-obesity; hexamethylene bisacetamide; hypothalamic neuropeptides; MYH9

Subject Categories Digestive System; Metabolism; Neuroscience

DOI 10.15252/emmm.202318024 | Received 17 May 2023 | Revised 19 October 2023 | Accepted 27 October 2023 | Published online 20 November 2023

EMBO Mol Med (2023) 15: e18024

Introduction

Obesity is considered a pandemic worldwide (Swinburn *et al*, 2011; Bluher, 2019). The main etiological factor of obesity is an energy imbalance between excessive calorie intake and reduced energy expenditure, which is often associated with impairments in the hypothalamic integration of peripheral signals and/or extrahypothalamic structures (Schwartz *et al*, 2000; Myers *et al*, 2021). The hypothalamus plays a key role in maintaining whole-body energy homeostasis through multiple neuronal networks and circuits (Myers & Olson, 2012; Myers *et al*, 2021). In particular, the arcuate nucleus (ARC) is a crucial hypothalamic nucleus that regulates feeding, energy expenditure, and overall energy homeostasis by sensing and responding to metabolic signals such as nutrients, hormones, and metabolites from the peripheral circulation (Myers *et al*, 2021). ARC contains neural circuits composed of two functionally distinct neuronal populations. One population expresses orexigenic neuropeptide Y (NPY) and agouti-related peptide (AgRP) that promote appetite and decrease energy expenditure, and the other population expresses anorexigenic proopiomelanocortin (POMC) and cocaine- and amphetamine-regulated transcript (CART) that promote satiety and increase energy expenditure. These neuropeptides play critical roles in energy balance by regulating appetite, thermogenesis in brown adipose tissue, and glucose homeostasis in the liver and muscle directly or indirectly by controlling the neuroendocrine and autonomic nervous system (Stanley & Leibowitz, 1985; Poggioli *et al*, 1986; Brady *et al*, 1990; Garcia de Yebenes *et al*, 1995; Huszar *et al*, 1997; Myers *et al*, 2021; Vohra *et al*, 2022). Since the ARC neural system has been suggested to be impaired in the obese state, restoring or regulating the function of the ARC is important for the improvement of obesity and its associated pathologies. Pharmacological interventions including agonists, antagonists (Roseberry *et al*, 2004; Brothers & Wahlestedt, 2010), hormone analogs (Huang *et al*, 2021), and natural (Matias *et al*, 2008; Li *et al*, 2018) or synthetic compounds (Loftus *et al*, 2000; Shimokawa *et al*, 2002; Hu *et al*, 2003; Tavares *et al*, 2007, 2013; Anderson *et al*, 2008) that enable modulation of neuropeptide expression in the ARC are considered promising strategies, and understanding the mechanisms of action of these compounds will provide therapeutic directions for the development of obesity treatment.

Hexamethylene bisacetamide (HMBA) is a bipolar planar compound (Fig 1A) that induces cell differentiation (Reuben *et al*, 1976) and is classified as an exogenous chemical in the Human Metabolome Database (Wishart *et al*, 2018). HMBA has been investigated as an anti-cancer agent acting through the induction of cell differentiation in various types of tumors including leukemia (Reuben *et al*, 1976), breast cancer (Guilbaud *et al*, 1990; Ketchart *et al*, 2016), and neuroblastoma (Kondo *et al*, 1990; Turano *et al*, 2006). HMBA also exerts anti-cancer function through the induction of

1 Department of Brain Sciences, Daegu Gyeongbuk Institute of Science and Technology, Daegu, Korea
2 Neurometabolomics Research Center, Daegu Gyeongbuk Institute of Science and Technology, Daegu, Korea
*Corresponding author. Tel: +82 53 785 6111; Fax: +82 53 785 6109; E-mail: ekkim@dgist.ac.kr

© 2023 The Authors. Published under the terms of the CC BY 4.0 license

apoptosis by regulating transcription factors of pro-inflammatory cytokines and cell survival kinase-signaling pathways (Siegel *et al*, 1998; Dey *et al*, 2008). The anti-cancer effects of HMBA are mediated mostly by HMBA-inducible protein 1 (HEXIM1) (He *et al*, 2006; Turano *et al*, 2006; Yoshikawa *et al*, 2012; Ketchart *et al*, 2013, 2016; Lama *et al*, 2017).

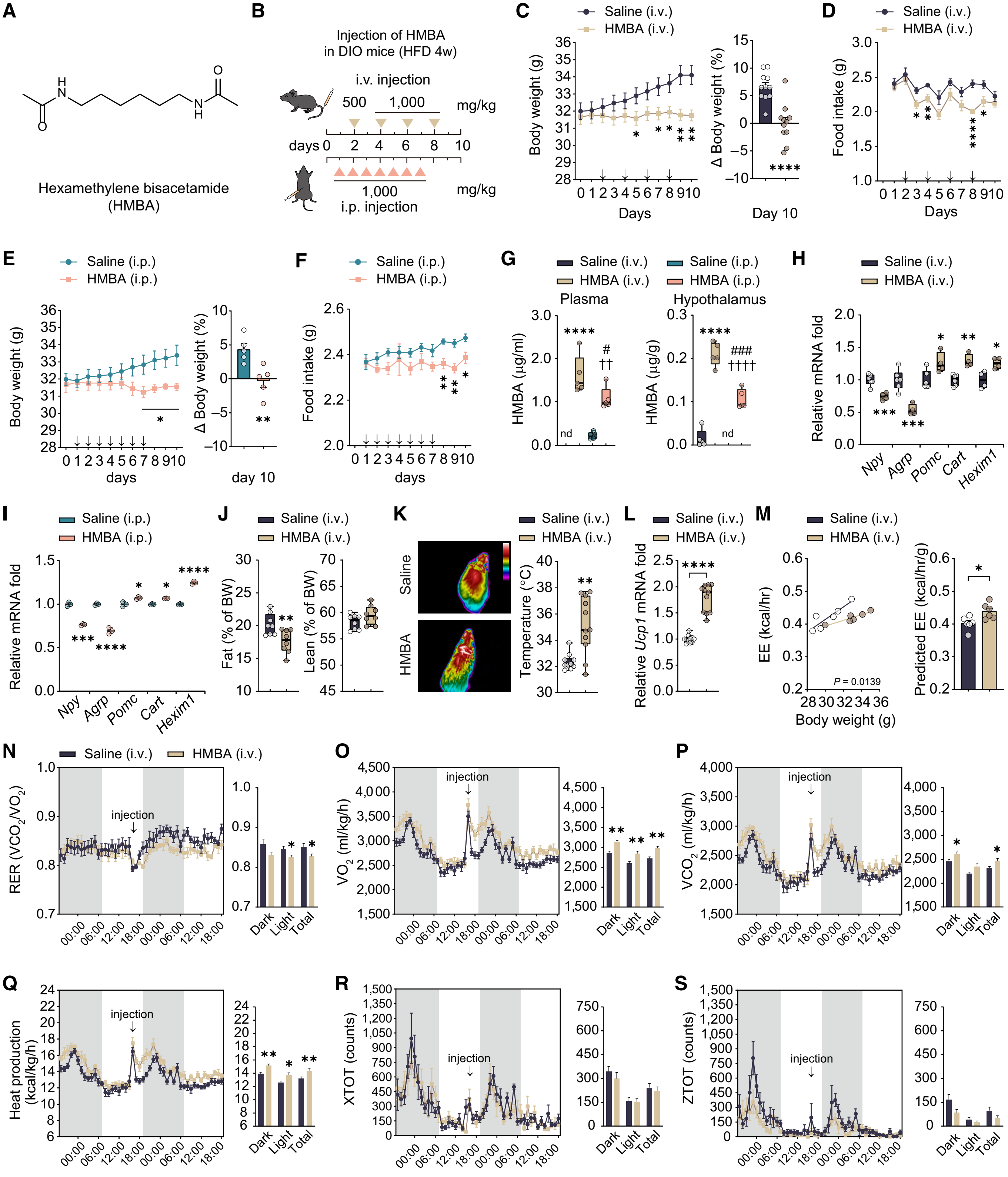

Figure 1.

 © 2023 The Authors

Figure 1. Peripheral administration of HMBA ameliorates obesity in DIO mice.

A Chemical structure of HMBA. HMBA has a symmetric structure with amides at both ends (CC(=O)NCCCCCCNC(=O)C).

B Design of intravenous and intraperitoneal injections in male DIO mice. Triangles represent the days of each injection.

C, D Changes in (C) daily body weight (grams) (left panel) and body weight (percentages) on day 10 relative to day 0 (right panel), and (D) food intake in male DIO mice injected intravenously (i.v.) with HMBA. $n = 10$ per group. Arrows represent the days of each injection.

E, F Changes in (E) daily body weight (grams) (left panel) and body weight (percentages) on day 10 relative to day 0 (right panel), and (F) food intake in male DIO mice injected intraperitoneally (i.p.) with HMBA. $n = 5$ per group. Arrows represent the days of each injection.

G The amounts of HMBA in plasma and hypothalamus. $n = 5$ per group.

H, I Relative mRNA levels of hypothalamic neuropeptides and *Hexim1* in (H) i.v. group and (I) i.p. group. RNA was isolated 4 h after injection. $n = 4$, saline (i.v.); $n = 6$, HMBA (i.v.); $n = 3$, saline (i.p.) or HMBA (i.p.).

J Fat and lean mass 2 days after the last dose of HMBA. $n = 8$ per group.

K Representative infrared thermography images and interscapular brown adipose tissue temperature. $n = 10$, Saline (i.v.); $n = 11$, HMBA (i.v.). The temperature of brown adipose tissue was determined as the average skin temperature of the same area in each thermography image (one image per mouse). In the color key, the temperature decreases from top to bottom.

L Relative mRNA levels of *Ucp1* of interscapular brown adipose tissue. $n = 10$, Saline (i.v.); $n = 11$, HMBA (i.v.).

M Regression-based analysis of energy expenditure (EE) against body weight (left panel). Bar graph indicates EE values adjusted for body weight using ANCOVA (right panel). $n = 6$ per group.

N–S Indirect calorimetry. (N) Respiratory exchange ratio. (O) VO_2 consumption. (P) VCO_2 production. (Q) Heat production. (R) Locomotor activity *X*-axis. (S) Locomotor activity *Z*-axis. $n = 6$ per group.

Data information: Data represent different numbers (*n*) of biological replicates. Data are represented in box and whisker plots where the central band denotes the median value and box contains interquartile ranges, while whiskers mark minimum and maximum values in panels (G–L). Statistical significance was determined by a two-tailed unpaired Student's *t*-test in panels (C–F), (H–S), and one-way ANOVA followed by a *post hoc* Tukey test in panels (G). $^{*}P < 0.05$, $^{**}P < 0.01$, $^{***}P < 0.001$, $^{****}P < 0.0001$ vs. Saline (i.v.); $^{\dagger}P < 0.05$, $^{\dagger\dagger\dagger}P < 0.001$, $^{\dagger\dagger\dagger\dagger}P < 0.0001$ vs. saline (i.p.); $^{\#}P < 0.05$, $^{\#\#\#}P < 0.001$ HMBA (i.v.) vs. HMBA (i.p.); nd, not detected. Data are mean ± SEM. The exact *P*-values are reported in Appendix Table S6.
Source data are available online for this figure.

The gene of HEXIM1 was initially cloned from human vascular smooth muscle cells and was predicted to encode a nuclear protein, which was upregulated after HMBA treatment (Kusuhara *et al*, 1999). According to the Human Protein Atlas (Uhlen *et al*, 2015), HEXIM1 is expressed at the highest level in the heart, kidney, and brain, whereas HEXIM2, a paralog of HEXIM1, is expressed mainly in the skeletal muscle and testis. HEXIM1 inhibits the activity of positive transcription elongation factor b (P-TEFb), preventing transcription elongation by RNA polymerase II (Pol II) (Yik *et al*, 2003; Dey *et al*, 2007). HEXIM1 also interacts with p53 in MCF7 breast cancer cells, and overexpression of HEXIM1 enhances the interaction and inhibits p53 ubiquitination, resulting in enhanced p53 stability and activation of p53 target gene expression required for DNA repair, apoptosis, and differentiation (Lew *et al*, 2012, 2013). Although p53 is a well-known potent tumor suppressor, its role in maintaining energy balance is also emerging (Yahagi *et al*, 2003; Quinones *et al*, 2018). Selective ablation of p53 in AgRP neurons (AgRPp53KO) of diet-induced obese (DIO) mice fed a high-fat diet (HFD) for a long period increased food intake, promoted weight gain, and reduced energy expenditure and thermogenic activity of brown adipose tissue in comparison with wild-type DIO mice (Quinones *et al*, 2018). In contrast, POMCp53KO DIO mice showed no changes in the overall metabolic phenotypes including feeding behavior, energy expenditure, and body weight (Quinones *et al*, 2018). Furthermore, obese *Hexim1* heterozygous mice fed an HFD showed increased adipogenesis in adipose tissue and enhanced glucose transporter 4 expression for glucose disposal in muscle as well as enhanced leptin signaling in the hypothalamus (Dhar-Mascareno *et al*, 2016). However, it has not been investigated yet whether HMBA has any metabolic effects or how HEXIM1 is involved in energy homeostasis.

Herein, we report that HMBA mitigates obesity by regulating the expression of the *Npy* and *Pomc* genes. HMBA-induced HEXIM1 downregulated *Npy* expression and upregulated *Pomc* expression in the hypothalamus of DIO mice, leading to reduced appetite, elevated energy expenditure, and weight loss. We propose that HMBA, which has long been investigated in oncology, can be repurposed for the control of appetite and body weight with the aim of extending its use to metabolic disorders. Understanding the action of HMBA and finding its biological targets in the regulation of energy homeostasis will provide not only mechanistic information but also therapeutic strategies relevant to metabolic diseases such as obesity.

Results

Peripheral administration of HMBA ameliorates obese phenotype in DIO mice

We searched the Broad Institute Connectivity Map database (Lamb *et al*, 2006) to select small-molecule candidates with potential efficacy in the regulation of metabolism. A comparison of gene expression signatures between oleoylethanolamide, a known anorexic endocannabinoid, that reduces food intake and body weight by regulating lipid and glucose metabolism (Fu *et al*, 2003; Guzman *et al*, 2004; Gonzalez-Yanes *et al*, 2005), and 2,429 compound profiles registered in the TouchStone v1.5 of dataset (Lamb *et al*, 2006) identified HMBA as one of the top 10 compounds with a high connectivity score (Appendix Table S1). We chose HMBA as a suitable candidate for this study since its effects on metabolic diseases such as obesity are not known yet at all among the listed compounds. Although the roles of otenzepad and valacyclovir on metabolism are unknown, these two drugs were excluded from the candidates due to their adverse effects on the central nervous system, nephrotoxicity, or tissue-specific response in previous studies (Mickala *et al*, 1996; Broadley & Kelly, 2001; Zhang *et al*, 2016; Brandariz-Nunez *et al*, 2021).

© 2023 The Authors

To investigate the effects of HMBA on obesity, male DIO mice fed an HFD for 4 weeks were injected with HMBA either intravenously (i.v.) or intraperitoneally (i.p.) as indicated in Fig 1B. Intravenous injections every other day (Fig 1C and D) or daily i.p. injections (Fig 1E and F) significantly reduced weight gain and food intake in comparison with those in saline-injected control groups.

To determine the effect of HMBA on food intake and body weight in females, we injected HMBA i.v. and i.p. to female DIO mice as the same experimental design in Fig 1B (Appendix Fig S1A–D). Our data showed that each injection of HMBA had very similar effects on the reduction in body weight and food intake in females as seen in HMBA-injected DIO males. It is likely that HMBA has the same effects on food intake and body weight regardless of gender. Hence, all subsequent experiments were consistently performed in male DIO mice.

To explore whether the peripherally injected HMBA is delivered to the hypothalamus and exerts a central effect, we analyzed and quantified HMBA in plasma and hypothalamus 4 h after the last injection using liquid chromatography with tandem mass spectrometry (LC–MS/MS). We detected 6.59 ± 0.71 μmoles of HMBA in plasma and 0.79 ± 0.09 μmoles in the hypothalamus per mouse. The concentrations of HMBA in plasma and hypothalamus were higher after i.v. injection than after i.p. injection (Fig 1G). Given these results, we tested whether peripherally delivered HMBA regulated whole-body metabolism by modulating orexigenic or anorexigenic neuropeptides. Both i.v. and i.p. administration of HMBA decreased the mRNA levels of *Npy* and *Agrp* and increased those of *Pomc* and *Cart* in the hypothalamus of DIO mice, whereas that of *Hexim1* was elevated in the hypothalamus of both HMBA-injected groups (Fig 1H and I).

To assess the effect of HMBA on body composition, we analyzed fat and lean masses using *in vivo* nuclear magnetic resonance 2 days after the last dose. Fat mass was significantly reduced, while lean mass was not changed (Fig 1J). We measured thermogenesis in brown adipose tissue. HMBA increased interscapular surface temperature adjacent to brown adipose tissues and the mRNA level of *Ucp1*, indicating greater thermogenesis (Fig 1K and L). HMBA led to marked induction in energy expenditure compared to saline-injected group, indicating that HMBA increased metabolic rates independently of the body mass (Fig 1M). Calorimetry analysis showed that HMBA increased metabolic consumption of oxygen and generation of carbon dioxide (Fig 1N–P) and increased heat production (Fig 1Q) without changes in locomotor activity (Fig 1R and S). Taken together, the data indicated that peripheral administration of HMBA ameliorated the obese phenotype in DIO mice.

Central administration of HMBA prevents obesity in DIO mice

To confirm the central effect of HMBA, we daily injected 400 nmoles of HMBA into the third ventricle (3 V) of male and female DIO mice, which resides near the hypothalamus (Fig 2A). This intracerebroventricular (i.c.v.) injection of HMBA significantly decreased body weight and food intake. After dosing was stopped, body weight remained low despite the recovery of food consumption in both sexes (Fig 2B and C, and Appendix Fig S1E and F). All subsequent experiments were conducted in male DIO mice.

Central administration of HMBA to mice fed a normal chow diet (NCD) also led to weight loss and reduced appetite (Appendix Fig S2).

Consistent with the effects of peripheral injection of HMBA, its central injection reduced the mRNA levels of *Npy* and *Agrp* and increased those of *Pomc*, *Cart*, and *Hexim1* in the hypothalamus (Fig 2D).

Metabolic parameters were measured on the last day of monitoring of body weight and food intake after i.c.v. injection of HMBA. Fat mass was dramatically decreased while lean mass was increased (Fig 2E). HMBA increased thermogenesis in brown adipose tissue

Figure 2. Central administration of HMBA has anti-obesity effects in DIO mice.

A Schematic of the experimental design. HMBA (400 nmoles) was injected intracerebroventricular (i.c.v.) daily for 7 days. Triangles represent the days of each injection.

B, C Changes in (B) daily body weight (grams) (left panel) and body weight (percentages) on day 21 relative to day 0 (right panel), and (C) food intake. $n = 15$, saline; $n = 16$, HMBA. Arrows represent the days of each injection.

D Relative mRNA levels of hypothalamic neuropeptides and *Hexim1*. RNA was isolated 4 h after injection. $n = 15$ per group. Arrows represent the days of each injection.

E Fat and lean mass. $n = 7$, saline; $n = 9$, HMBA.

F Representative infrared thermography images and interscapular brown adipose tissue temperature. $n = 7$ per group. The temperature of brown adipose tissue was determined as the average skin temperature of the same area in each thermography image (one image per mouse). In the color key, the temperature decreases from top to bottom.

G Relative mRNA levels of *Ucp1* of interscapular brown adipose tissue. $n = 7$ per group.

H Regression-based analysis of energy expenditure (EE) against body weight (left panel). Bar graph indicates EE values adjusted for body weight using ANCOVA (right panel). $n = 5$ per group.

I–N Indirect calorimetry. (I) Respiratory exchange ratio. (J) VO_2 consumption. (K) VCO_2 production. (L) Heat production. (M) Locomotor activity *X*-axis. (N) Locomotor activity *Z*-axis. $n = 5$ per group.

O Glucose tolerance test (GTT). $n = 9$ per group.

P Insulin tolerance test (ITT). $n = 9$ per group.

Q Serum triglycerides (TGs). $n = 9$ per group.

R Plasma free fatty acids (FFAs). $n = 6$ per group. MUFA, monounsaturated fatty acids; PUFA, polyunsaturated fatty acids; SFA, saturated fatty acids.

Data information: Data represent different numbers (*n*) of biological replicates. Data are represented in box and whisker plots where the central band denotes the median value, box contains interquartile ranges, while whiskers mark minimum and maximum values in panels (D–G). Statistical significance was determined by a two-tailed unpaired Student's *t*-test in panels (B–N) and (Q–R), and two-way ANOVA followed by a *post hoc* Tukey test in panels (O) and (P). $^*P < 0.05$, $^{**}P < 0.01$, $^{***}P < 0.001$, $^{****}P < 0.0001$ vs. saline. Data are mean ± SEM. The exact *P*-values are reported in Appendix Table S6.
Source data are available online for this figure.

 © 2023 The Authors

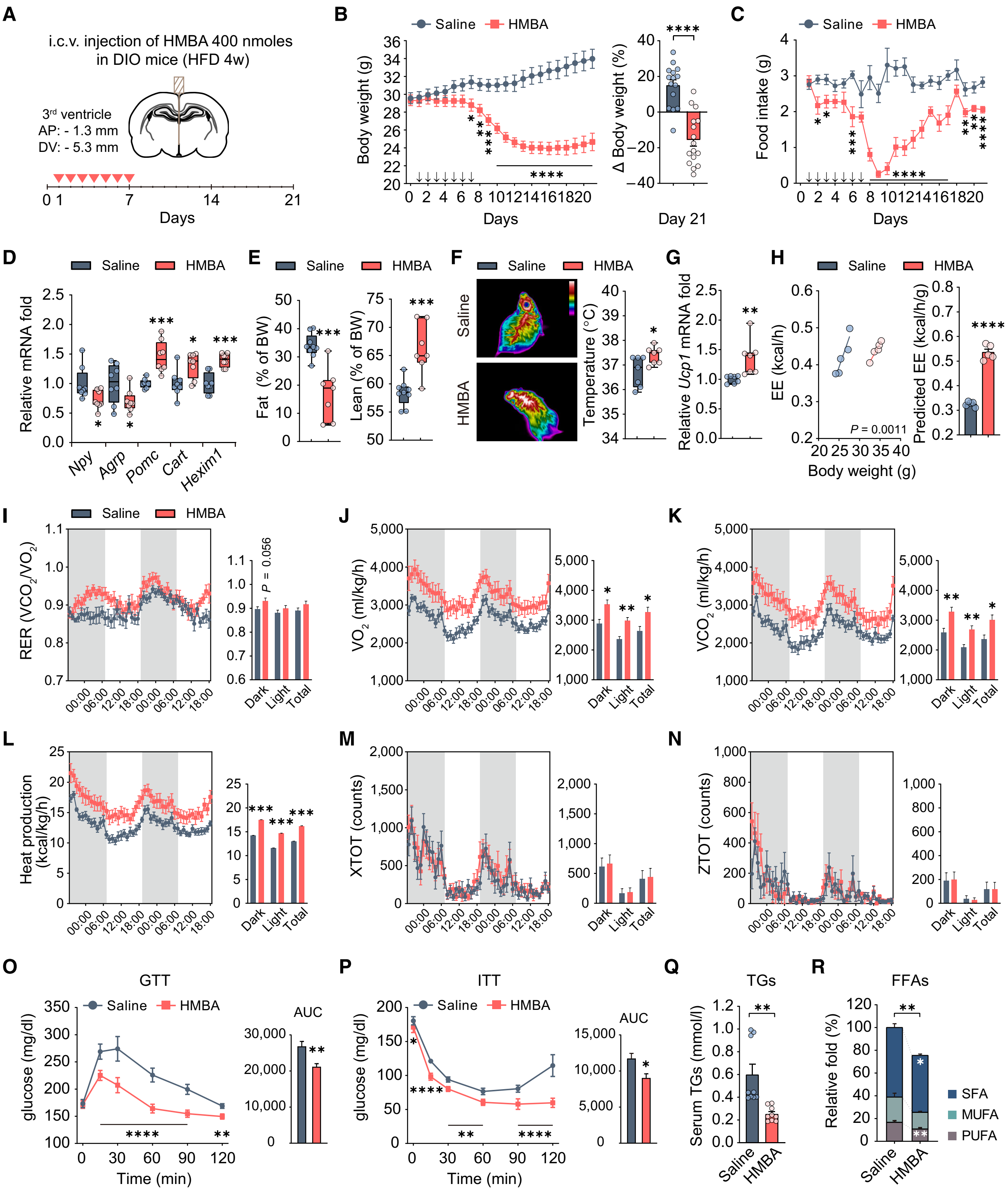

Figure 2.

(Fig 2F and G). Consistent with i.v. injection of HMBA, i.c.v. injection of HMBA significantly increased metabolic rates independently of the body mass (Fig 1H). Consumption of oxygen, generation of carbon dioxide (Fig 2I–K), and heat production (Fig 2L) were elevated by HMBA without changes in a day/nighttime locomotor activity (Fig 2M and N).

© 2023 The Authors

To investigate the effect of HMBA on glucose homeostasis and insulin sensitivity, we performed i.p. glucose tolerance test and insulin tolerance test in DIO mice. Glucose intolerance and insulin sensitivity were significantly improved by HMBA (Fig 2O and P). In addition, the concentrations of triglycerides and the relative amounts of free fatty acids (saturated and polyunsaturated) were significantly reduced by HMBA injection (Fig 2Q and R).

HMBA did not cause sickness behaviors in DIO mice

To investigate whether HMBA exhibited sickness behaviors, we monitored various behavioral tests in DIO mice. First, we observed that none of the mice displayed any noticeable signs of sickness such as vomiting, diarrhea, inactivity, and hair loss during HMBA treatment. Our metabolic cage data also showed no negative effect of HMBA on locomotor activity compared with saline-injected mice. Second, in order to demonstrate the health status of the mice more accurately, we employed the laboratory animal behavior observation registration and analysis system (LABORAS) (Fig EV1) after the last dosing of HMBA (i.v., i.p., or i.c.v.). The LABORAS data demonstrated that eating behavior was decreased by HMBA treatment, however, no significant differences in the other behaviors were observed compared to saline-injected groups (Fig EV1A, B, D, E, G, and H). Lastly, we performed a conditioned taste aversion (CTA) test to assess the effect of HMBA on a taste aversion associated with an illness over sucrose preference (Fig EV1). The CTA test showed that HMBA did not induce a taste aversion (Fig EV1C, F and I). Collectively, our data demonstrated that HMBA did not exhibit sickness-associated or aversive appetite suppression and weight loss under our experimental conditions.

HMBA is metabolized in hypothalamic tissue and cells

HMBA in the body is sequentially metabolized into N-acetyl-1,6-diaminohexane (NADAH), 1,6-diaminohexane (DAH), and 6-aminohexanoic acid (AmHA) (Fig 3A), and the metabolic rate varies depending on cell type or dosage (Callery *et al*, 1986; Egorin *et al*, 1988). We simultaneously analyzed these metabolites with LC–MS/MS by obtaining high-resolution peaks of each metabolite through multiple reaction monitoring (Fig 3B). Using this approach, we determined the metabolic flow of HMBA in the hypothalamus and plasma of DIO mice harvested 4 h after i.c.v. injection of HMBA. High levels of HMBA and NADAH were detected in the hypothalamus, and DAH and AmHA were also readily detectable. The molar concentrations of HMBA (47%) and NADAH (47.6%) were much higher than those of DAH and AmHA (Fig 3C). However, the i.c.v. injection of HMBA did not affect plasma HMBA concentration (Appendix Fig S3).

We also screened HMBA and its metabolites in hypothalamic cell lines expressing *Npy* (mHypoE-N41) or *Pomc* (mHypoE-N43/5) treated with 0.1 mM HMBA for 1–8 h (Fig 3D and E). The concentration of 0.1 mM HMBA did not reduce cell survival and was non-toxic (Appendix Fig S4). HMBA gradually decreased from 1 to 8 h in both cell lines. NADAH decreased by 4 h, probably due to its breakdown to DAH or AmHA. DAH and AmHA continuously increased from 1 to 8 h. The molar concentration of HMBA was much higher (80.7% in mHypoE-N41 and 82.2% in mHypoE-N43/5) than those of its metabolites. These data suggest that HMBA remains mainly intact, although it is metabolized to some extent in the hypothalamic cell lines tested.

HMBA regulates neuropeptide expression in a HEXIM1-dependent manner

To elucidate the mechanism by which HMBA regulates the neuropeptide expression *in vitro*, we treated hypothalamic cells with HMBA for up to 24 h and examined the changes in neuropeptide expression. In HMBA-treated cells, the mRNA level of *Npy* declined, with the lowest value at 2 h, and remained significantly lower than the control until 24 h (Fig 3F), whereas that of *Pomc* was significantly higher than in the control at 0.5 h and peaked at 2 h (Fig 3G). The time point (2 h) and the concentration of HMBA (0.1 mM) that showed the strongest anorexigenic effect (Fig 3H and I) were selected for the following experiments.

We hypothesized that HMBA-induced HEXIM1 regulates *Npy* and *Pomc* expression since *Hexim1* was the dominant isoform in

Figure 3. HMBA regulates neuropeptide expression in a HEXIM1-dependent manner.

- A Metabolic pathway of HMBA.
- B Chromatogram peaks of LC–MS/MS.
- C Metabolite concentrations in the hypothalamus and relative percentage of molar concentrations. Metabolomic analysis was performed 4 h after i.c.v. injection of 400 nmoles of HMBA. $n = 6$ per group.
- D Metabolic flow of HMBA in mHypoE-N41 and relative percentage of the area under the curve (AUC). $n = 3$ per group.
- E Metabolic flow of HMBA in mHypoE-N43/5 and relative percentage of the area under the curve (AUC). $n = 3$ per group.
- F Relative mRNA levels of *Npy* upon HMBA treatment up to 24 h. $n = 3$ per group.
- G Relative mRNA levels of *Pomc* upon HMBA treatment up to 24 h. $n = 3$ per group.
- H, I Relative mRNA levels of (H) *Npy* and (I) *Pomc*. Cells were treated with 10^{-3}, 10^{-2}, or 10^{-1} mM HMBA for 1 or 2 h. $n = 3$ per group.
- J Heatmap of HEXIM isoforms' quantification cycle (cq) values in the hypothalamus and cell lines. $n = 3$ per group.
- K, L Relative mRNA levels of *Hexim1* upon HMBA treatment up to 24 h in (K) mHypoE-N41 and (L) mHypoE-N43/5. $n = 3$ per group.
- M, N Relative basal mRNA levels of (M) *Npy* and (N) *Pomc* in *Hexim1* KD (si-*Hexim1*). $n = 3$ per group.
- O, P Relative mRNA levels of (O) *Npy* and (P) *Pomc* in *Hexim1* KO (sg*Hexim1*) after HMBA treatment for 2 h. $n = 3$ per group.

Data information: (D–G) and (K–P), Cells were treated with 0.1 mM HMBA. (A) Data represent six biological replicates. (D, E, J) Data represent three technical replicates. The datasets in qPCR experiments were comprised of three biological replicates and each biological replicate was an average of three technical replicates. (F–L) Statistical significance was determined by two-way ANOVA followed by a *post hoc* Bonferroni test. *$P < 0.05$, **$P < 0.01$, ***$P < 0.001$, ****$P < 0.0001$. (M–P) Statistical significance was determined by two-way ANOVA followed by a *post hoc* Tukey test. *$P < 0.05$, **$P < 0.01$, ***$P < 0.001$, ****$P < 0.0001$; ns or unless otherwise stated, no significance; nd, not detected. Data are mean ± SEM. The exact *P*-values are reported in Appendix Table S6.
Source data are available online for this figure.

© 2023 The Authors

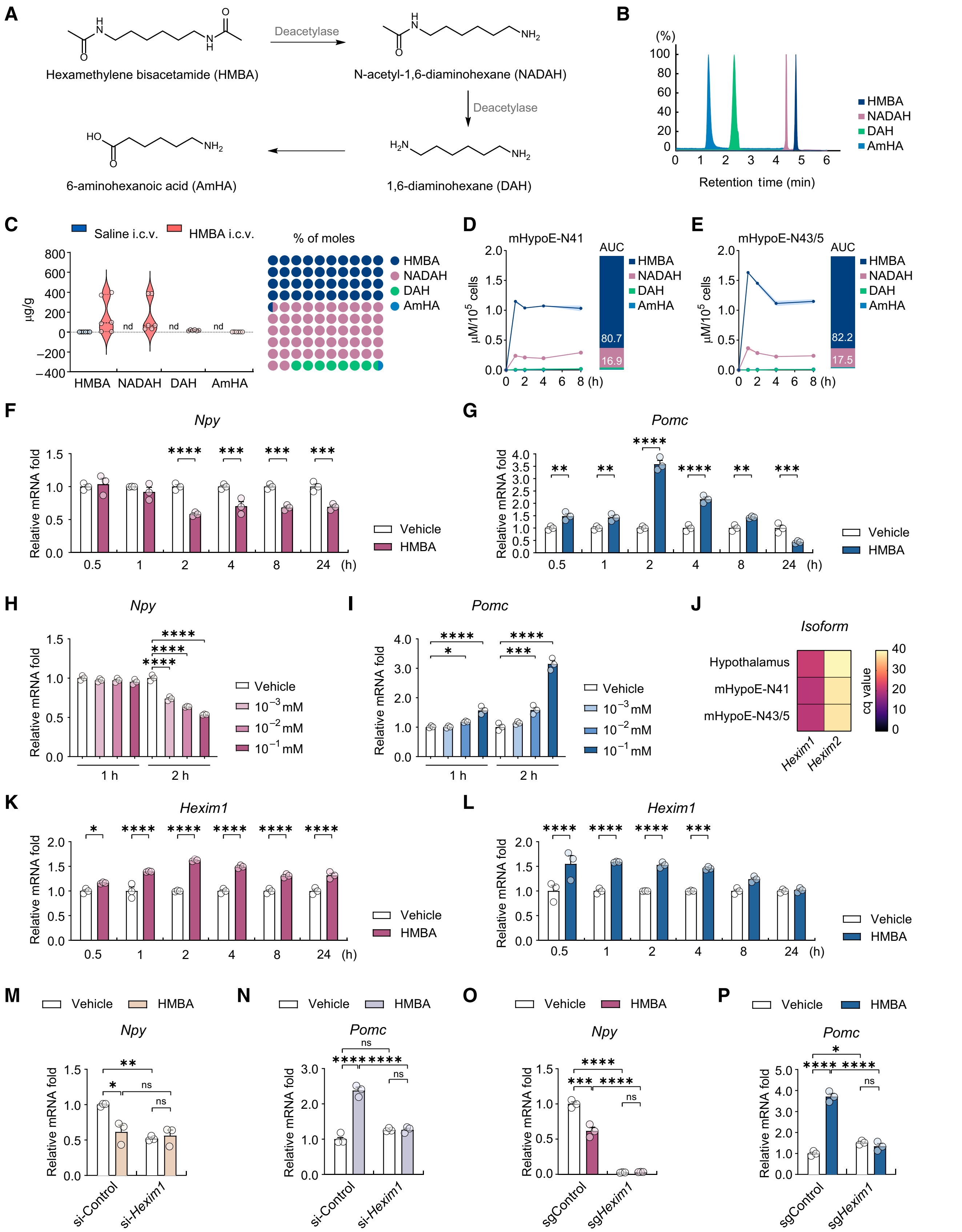
A
Hexamethylene bisacetamide (HMBA)
Deacetylase
N-acetyl-1,6-diaminohexane (NADAH)
Deacetylase
1,6-diaminohexane (DAH)
6-aminohexanoic acid (AmHA)
B
Retention time (min)
HMBA
NADAH
DAH
AmHA
C
Saline i.c.v.
HMBA i.c.v.
μg/g
% of moles
nd
D
mHypoE-N41
AUC
80.7
16.9
E
mHypoE-N43/5
82.2
17.5
F
Npy
Vehicle
HMBA
Relative mRNA fold
G
Pomc
H
Npy
I
Pomc
J
Isoform
Hypothalamus
mHypoE-N41
mHypoE-N43/5
cq value
Hexim1
Hexim2
K
Hexim1
L
Hexim1
M
N
O
P
si-Control
si-Hexim1
sgControl
sgHexim1
ns

Figure 3.

comparison with *Hexim2* in mHypoE-N41, mHypoE-N43/5, and hypothalamic tissue (Fig 3J). After HMBA treatment of hypothalamic cells, *Hexim1* induction was maintained up to 24 h in mHypoE-N41 (Fig 3K), and up to 4 h in mHypoE-N43/5 (Fig 3L). To study the role of HEXIM1 in neuropeptide regulation, we generated hypothalamic cell lines with *Hexim1* knockdown (*Hexim1* KD) using siRNA transfection or knockout (*Hexim1* KO) using CRISPR-Cas9 (Appendix Fig S5). In the absence of HMBA, *Hexim1* KD or KO decreased the mRNA levels of *Npy* and slightly increased those of *Pomc*, with greater effects in KO than in KD (Fig 3M–P), indicating that HEXIM1 also regulates the basal expression of those neuropeptides. The anorexigenic effects of HMBA, which reduced *Npy* and elevated *Pomc* gene expression, were abolished in *Hexim1* KD (Fig 3M and N) and *Hexim1* KO (Fig 3O and P), indicating that HEXIM1 is required for HMBA-dependent regulation of *Npy* and *Pomc*.

HMBA binds to MYH9 and ACTG1 in hypothalamic cells

To identify the proteins binding HMBA in hypothalamic cells, we applied a chemical proteomics approach wherein biotin-conjugated HMBA (hereafter referred to as HMBA–biotin) was used as a bait for HMBA targets. HMBA–biotin was synthesized by reacting the primary amine group of NADAH with the N-hydroxysulfosuccinimide group of biotins (Fig 4A). The conversion rate of NADAH to HMBA–biotin was 92.9% (Fig 4B). The structure and *m/z* value of the product were verified by ^{1}H NMR and LC–MS/MS, respectively (Fig 4C and Appendix Table S2). Treatment with HMBA–biotin for 2 h was sufficient to induce HEXIM1, confirming that HMBA–biotin retained the biological activity of HMBA (Fig 4D).

HMBA-binding proteins were identified by biotin–neutravidin pull-down assay and peptide analysis using LC–MS/MS (Fig 4E). Two protein bands from Coomassie blue gels were identified as MYH9 (non-muscle myosin heavy-chain IIA, 230 kDa) and ACTG1 (γ-actin, 42 kDa) based on the highest mascot scores (Fig 4F). We predicted the structures of the protein–HMBA complexes, including the HMBA-binding pockets using an autodocking approach (Forli *et al*, 2016). HMBA was successfully docked at the best poses of MYH9 (Fig EV2A) and ACTG1 (Fig EV2B). In the docking model of HMBA–MYH9, a hydrogen bond was formed between the hydroxyl group (acceptor O atom) at the amide of HMBA and the guanidinium side chain of Arg424 (donor H atom), and another one was formed between the amine group at the symmetrical amide of HMBA (donor H atom) and the carboxyl group of Leu611 (acceptor O atom). Trp428 was revealed as a residue in the hydrophobic pocket potentially capable of hydrogen bonding with HMBA (Figs 4G and EV2C). In the docking model of HMBA–ACTG1, a hydrogen bond was formed between the hydroxyl group (acceptor O atom) of HMBA and the amine group of the side chain of Lys213 (donor H atom), and another one between the symmetrical hydroxyl group (acceptor O atom) of HMBA and the amine group of Gly15 (donor H atom). HMBA potentially had hydrogen bonds and hydrophobic interactions with Met16, Asp157, Glu214, and Tyr306 of ACTG1 (Figs 4H and EV2D). These data suggest that MYH9 and ACTG1 directly bind HMBA.

MYH9 and ACTG1 are required for the HMBA-dependent regulation of neuropeptide expression via HEXIM1 induction

To determine the role of MYH9 and ACTG1 in the regulation of neuropeptide expression by HMBA, *Myh9* and *Actg1* were knocked down in hypothalamic cells, which were then treated with HMBA (Figs 4I–P and EV3A and B). Knockdown of either gene abolished or reduced not only the HMBA-induced decrease in *Npy* and increase in *Pomc* expression but also the HMBA-induced increase in *Hexim1* expression (Fig 4I–P). Double knockdown of *Myh9* and *Actg1* completely abolished HMBA-induced increase in *Hexim1* expression in mHypoE-N41 (Fig EV3C and D) and mHypoE-N43/5 (Fig EV3F and G). The effects of HMBA on *Npy* and *Pomc* expression were also abolished by double knockdown (Fig EV3E and H). Overall, these data indicate that MYH9 and ACTG1 are required for the HMBA-dependent regulation of neuropeptide expression via HEXIM1 induction.

Figure 4. HMBA regulates neuropeptide expression through binding to MYH9 and ACTG1.

- A Schematic diagram of NADAH biotinylation (HMBA–biotin, biotin-conjugated HMBA).
- B LC–MS/MS chromatogram peaks of NADAH (−biotin, pink) and HMBA–biotin (+biotin, green), and a bar graph of percentage peak integration values before and after biotinylation.
- C A 400-MHz 1D ^{1}H NMR spectrum of HMBA–biotin. Chemical shifts are described in the Materials and Methods section.
- D Western blot analysis of HEXIM1 after HMBA–biotin treatment. Blot quantifications are the mean of two biological replicates.
- E Schematic of the experimental design of chemical proteomics study including biotin pull-down using neutravidin and LC–MS/MS peptide analysis.
- F Coomassie blue-stained gel of samples after biotin pull-down (left panel) and Mascot score plots of MYH9 and ACTG1 (right panel). In the stained gel, blue ellipses indicate MYH9 (230 kDa) and pink ellipses indicate ACTG1 (42 kDa).
- G, H Docked conformation of (G) the MYH9–HMBA complex and (H) the ACTG1–HMBA complex. Raw data generated by AutoDock were verified and displayed through Protein Plus (left panels) and PLIP (right panels). HMBA is shown as gray (left panels) and yellow (right panels) sticks. Amino acid residues are shown as cyan sticks. Hydrogen bonds are indicated by solid gray lines with the distance (Å) from the donor atom to the acceptor atom indicated, and hydrophobic interactions are indicated by dashed yellow lines. N and O atoms are shown in blue and red, respectively. Residues in black are involved in major hydrogen bonding, while those in gray are involved in hydrophobic interactions.
- I, J Relative mRNA levels of (I) *Hexim1* and (J) *Npy* in si-*Myh9* of mHypoE-N41. $n = 3$ per group.
- K, L Relative mRNA levels of (K) *Hexim1* and (L) *Npy* in si-*Actg1* of mHypoE-N41. $n = 3$ per group.
- M, N Relative mRNA levels of (M) *Hexim1* and (N) *Pomc* in si-*Myh9* of mHypoE-N43/5. $n = 3$ per group.
- O, P Relative mRNA levels of (O) *Hexim1* and (P) *Pomc* in si-*Actg1* of mHypoE-N43/5. $n = 3$ per group.

Data information: Cells were treated with 0.1 mM HMBA or HMBA–biotin for 2 h. The datasets in qPCR experiments were comprised of three biological replicates and each biological replicate was an average of three technical replicates. Statistical significance was determined by two-way ANOVA followed by a *post hoc* Tukey test. $^{**}P < 0.01$, $^{***}P < 0.001$, $^{****}P < 0.0001$; ns or unless otherwise stated, no significance. Data are mean ± SEM. The exact *P*-values are reported in Appendix Table S6.
Source data are available online for this figure.

© 2023 The Authors

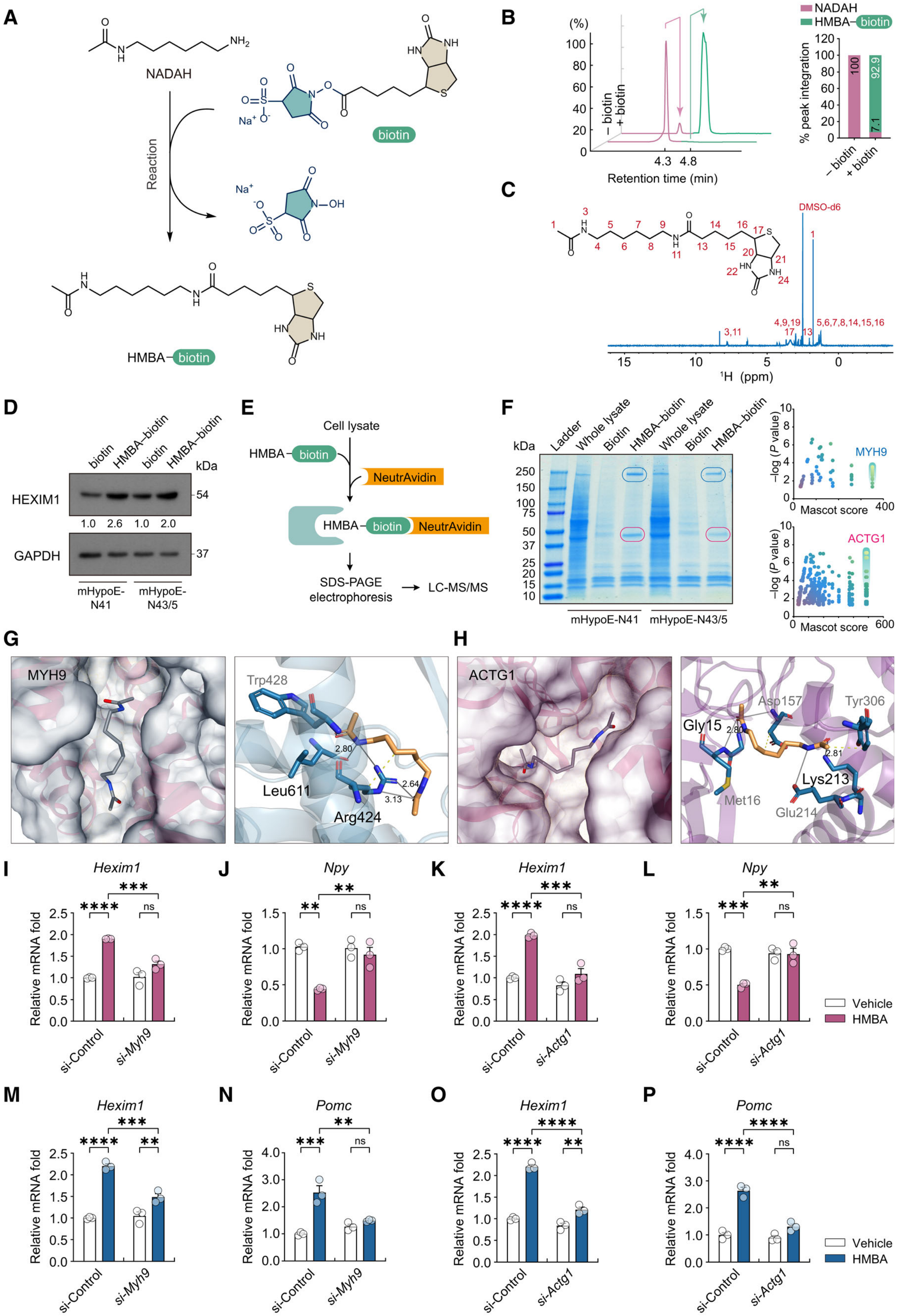
A
NADAH
Reaction
biotin
HMBA–biotin
B
NADAH
HMBA–biotin
– biotin
+ biotin
Retention time (min)
% peak integration
100
92.9
7.1
C
DMSO-d6
^{1}H (ppm)
D
biotin
HMBA–biotin
HEXIM1
GAPDH
1.0 2.6 1.0 2.0
kDa
mHypoE-N41
mHypoE-N43/5
E
Cell lysate
NeutrAvidin
SDS-PAGE electrophoresis
LC-MS/MS
F
Ladder
Whole lysate
Biotin
HMBA–biotin
mHypoE-N41
mHypoE-N43/5
–log (P value)
Mascot score
MYH9
ACTG1
G
MYH9
Trp428
Leu611
Arg424
H
ACTG1
Gly15
Asp157
Tyr306
Met16
Lys213
Glu214
I Hexim1
J Npy
K Hexim1
L Npy
M Hexim1
N Pomc
O Hexim1
P Pomc
Relative mRNA fold
si-Control
si-Myh9
si-Actg1
Vehicle
HMBA

Figure 4.

To determine the role of HMBA-induced HEXIM1 in regulating neuropeptide expression in the NPY and POMC neurons *in vivo*, NPY-hrGFP and POMC-hrGFP DIO mice fed an HFD for 4 weeks were injected with HMBA i.p., and then measured the fluorescence intensity of HEXIM1 expression in hrGFP-positive (hrGFP$^+$) cells that express NPY and POMC, respectively (Fig 5A–D and Appendix Fig S6A–C). In NPY-hrGFP DIO mice, HMBA decreased the fluorescence intensity of hrGFP (Fig 5A) and increased that of HEXIM1 in hrGFP$^+$ cells (Fig 5B). In POMC-hrGFP DIO mice, HMBA increased the fluorescence intensity of hrGFP (Fig 5C) and that of HEXIM1 in hrGFP$^+$ cells (Fig 5D). There was no difference in the number of NPY or POMC hrGFP$^+$ cells in HMBA-injected mice when compared with saline-injected mice (Appendix Fig S6B and C). In order to clarify whether HMBA has a specific effect on NPY and POMC neurons, we investigated the expression of HEXIM1 in diverse cells such as neurons, microglia, and astrocytes in the hypothalamus of NPY-hrGFP and POMC-hrGFP mice injected with HMBA (Fig EV4). Our data revealed that HEXIM1 is mainly expressed in neurons and hardly detected in astrocytes and microglia. Notably, HMBA increased the expression of HEXIM1 in NPY and POMC neurons but not in other neurons or glia in the ARC (Fig EV4). These results confirmed that HMBA decreased NPY and increased POMC by inducing HEXIM1 expression in the hypothalamic neurons.

To shed more light on the roles of MYH9 and ACTG1 in NPY or POMC neurons for metabolic regulation, we generated mice with selective double knockdown of *Myh9* and *Actg1* in *Npy*-expressing AgRP or POMC neurons. Specific Cre transgenic DIO mice fed an HFD for 4 weeks were injected with AAV-FLEXon-shRNAs (shControl or sh*Myh9* + sh*Actg1*)-EGFP viruses into the ARC (Fig 5E and Appendix Fig S7). The functional efficiency of the viral vectors was demonstrated via the expression of EGFP in the ARC (Appendix Fig S6D–G). As shown in Fig 5F and G, MYH9 and ACTG1 expression were successfully knocked down in AgRP and POMC neurons. Silencing of *Myh9* and *Actg1* in AgRP or POMC neurons failed to induce HEXIM1 expression by the administration of HMBA (Fig 5H and I), suggesting that MYH9 and ACTG1 are needed for HMBA-induced HEXIM expression in AgRP and POMC neurons.

The effects of HMBA on *Npy* and *Pomc* expression were also abolished by double knockdown of *Myh9* and *Actg1* in AgRP or POMC neurons (Fig 5J and K). Notably, silencing either genes in AgRP or POMC neurons of DIO mice during administration of HMBA for 7 days showed no differences in body weight and food intake when compared with the control group that received AAV-shControl (Fig 5L–O). Collectively, the reducing effects of HMBA on body weight and food intake were completely abrogated by silencing of *Myh9* and *Actg1* in AgRP (Fig 5L and M) and POMC (Fig 5N and O) neurons, suggesting that MYH9 and ACTG1 are required for the anti-obesity effects of HMBA.

HEXIM1 and p53 differentially regulate neuropeptide expression

Recent studies have shown that HEXIM1 interacts with p53 (Lew *et al*, 2012, 2013) and p53 in AgRP neurons regulates the expression of *Agrp* and *Npy* (Quinones *et al*, 2018). Therefore, we examined whether p53 is required for the modulation of *Npy* and *Pomc* expression by HMBA. Knockdown of *p53* by siRNA (Fig 6A and C) prevented the HMBA-induced decrease and increase in the expression of *Npy* and *Pomc*, respectively (Fig 6B and D). Interestingly, in contrast to *Hexim1*, *p53* knockdown did not affect the basal expression of *Npy* or *Pomc*, suggesting that HEXIM1 and p53 rely on different mechanisms to regulate the expression of *Npy* and *Pomc*. In short, p53 in both cell lines was dispensable for basal expression of

Figure 5. Anti-obesity effects of HMBA are abolished by ablation of *Myh9* and *Actg1* in AgRP or POMC neurons of DIO mice.

A–D Immunohistochemistry analysis for the fluorescence intensity of NPY, POMC, and HEXIM1 in the ARC of (A, B) NPY-hrGFP DIO mice and (C, D) POMC-hrGFP DIO mice after HMBA treatment. The fluorescence intensity of NPY and POMC expression was determined based on the native fluorescence of hrGFP, while the fluorescence intensity of HEXIM1 (Cy3) which co-localized with hrGFP-positive cells was determined. NPY-hrGFP and POMC-hrGFP DIO mice fed an HFD for 4 weeks were injected with HMBA (i.p., 1,000 mg/kg), and then brains were harvested 4 h after injection. $n = 32$ per group: eight brain slices were obtained from each of the four mice per group for determining hrGFP intensity in panels (A) and (C); $n = 16$ per group: four brain slices were obtained from each of the four mice per group for determining HEXIM1 intensity in panels B and D. Scale bars, 25 μm.

E Schematic diagram of studies on double knockdown of *Myh9* and *Actg1* in AgRP or POMC neurons. AgRP^{Cre} and POMC^{Cre} DIO mice fed an HFD for 4 weeks were injected with AAV-FLEXon-shRNAs (shControl or sh*Myh9* + sh*Actg1*)-EGFP PHP.eB serotype viruses into the ARC. After recovery, mice were injected daily with HMBA for 7 days (i.p., 1,000 mg/kg).

F, G Immunohistochemistry analysis for the knockdown efficiency of MYH9 and ACTG1 in the ARC of (F) AgRP^{Cre}::AAV$^{(\text{shControl or sh}Myh9+\text{sh}Actg1)}$ DIO mice and (G) POMC^{Cre}::AAV$^{(\text{shControl or sh}Myh9+\text{sh}Actg1)}$ DIO mice after HMBA treatment. The fluorescence intensity of MYH9 (Cy3) or ACTG1 (Alexa 647; Blue) which co-localized with EGFP-positive cells was determined. $n = 25$ per group: A total of 25 EGFP-positive cells were randomly counted across eight brain slices obtained from two mice per group. Scale bars, 25 μm.

H, I Immunohistochemistry analysis for the fluorescence intensity of HEXIM1 in the ARC of (H) AgRP^{Cre}::AAV$^{(\text{shControl or sh}Myh9+\text{sh}Actg1)}$ DIO mice and (I) POMC^{Cre}::AAV$^{(\text{shControl or sh}Myh9+\text{sh}Actg1)}$ DIO mice after HMBA treatment. The fluorescence intensity of HEXIM1 (Cy3) which co-localized with EGFP-positive cells was determined. $n = 24$ per group: eight brain slices were obtained from each of the three mice. Scale bars, 25 μm.

J, K Relative mRNA levels of hypothalamic neuropeptides *Npy* and *Pomc* in (J) AgRP^{Cre}::AAV$^{(\text{shControl or sh}Myh9+\text{sh}Actg1)}$ DIO mice and (K) POMC^{Cre}::AAV$^{(\text{shControl or sh}Myh9+\text{sh}Actg1)}$ DIO mice after HMBA treatment. RNA was isolated 4 h after HMBA treatment. $n = 6$ per group.

L, M Changes in (L) daily body weight (grams) (left panel) and body weight (percentages) on day 10 relative to day 0 (right panel), and (M) food intake for AgRP^{Cre}::AAV$^{(\text{shControl or sh}Myh9+\text{sh}Actg1)}$ DIO mice after HMBA treatment. $n = 7$, shControl group; $n = 6$, sh*Myh9* + sh*Actg1* group.

N, O Changes in (N) daily body weight (grams) (left panel) and body weight (percentages) on day 10 relative to day 0 (right panel), and (O) food intake for POMC^{Cre}::AAV$^{(\text{shControl or sh}Myh9+\text{sh}Actg1)}$ DIO mice after HMBA treatment. $n = 6$, saline-injected group; $n = 7$, HMBA-injected group.

Data information: (J–O) Data represent different numbers (*n*) of biological replicates. Data are represented in box and whisker plots where the central band denotes the median value, box contains interquartile ranges, while whiskers mark minimum and maximum values in panels (L) and (N). Statistical significance was determined by a two-tailed unpaired Student's *t*-test in panels (A–D) and (H–K), and two-way ANOVA followed by a *post hoc* Bonferroni test in panels (F) and (G), and two-way ANOVA followed by a *post hoc* Tukey test in panels (L–O). $^*P < 0.05$, $^{**}P < 0.01$, $^{***}P < 0.001$, $^{****}P < 0.0001$ vs. between HMBA treatment, and $^{\#}P < 0.05$, $^{\#\#}P < 0.01$, $^{\#\#\#}P < 0.001$, $^{\#\#\#\#}P < 0.0001$ vs. saline. Data are mean ± SEM. The exact *P*-values are reported in Appendix Table S6.
Source data are available online for this figure.

© 2023 The Authors

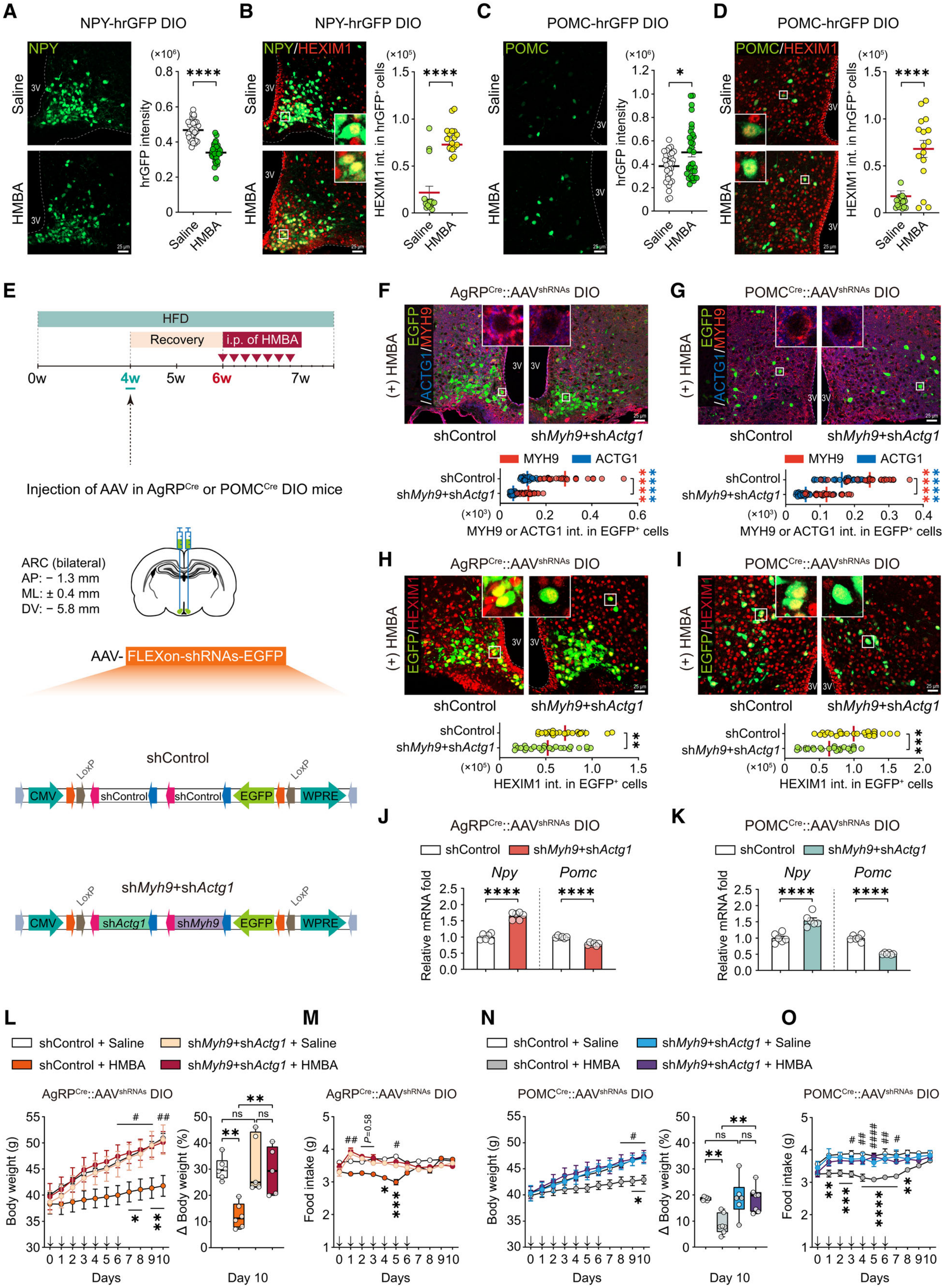
A
NPY-hrGFP DIO
B
NPY-hrGFP DIO
C
POMC-hrGFP DIO
D
POMC-hrGFP DIO
Saline
HMBA
hrGFP intensity
HEXIM1 int. in hrGFP+ cells
E
HFD
Recovery
i.p. of HMBA
0w 4w 5w 6w 7w
Injection of AAV in AgRPCre or POMCCre DIO mice
ARC (bilateral)
AP: − 1.3 mm
ML: ± 0.4 mm
DV: − 5.8 mm
AAV- FLEXon-shRNAs-EGFP
shControl
shMyh9+shActg1
CMV
LoxP
EGFP
WPRE
F
AgRPCre::AAVshRNAs DIO
G
POMCCre::AAVshRNAs DIO
MYH9 or ACTG1 int. in EGFP+ cells
H
I
HEXIM1 int. in EGFP+ cells
J
K
Npy
Pomc
Relative mRNA fold
L
M
N
O
shControl + Saline
shControl + HMBA
shMyh9+shActg1 + Saline
shMyh9+shActg1 + HMBA
Body weight (g)
Δ Body weight (%)
Food intake (g)
Days
Day 10

Figure 5.

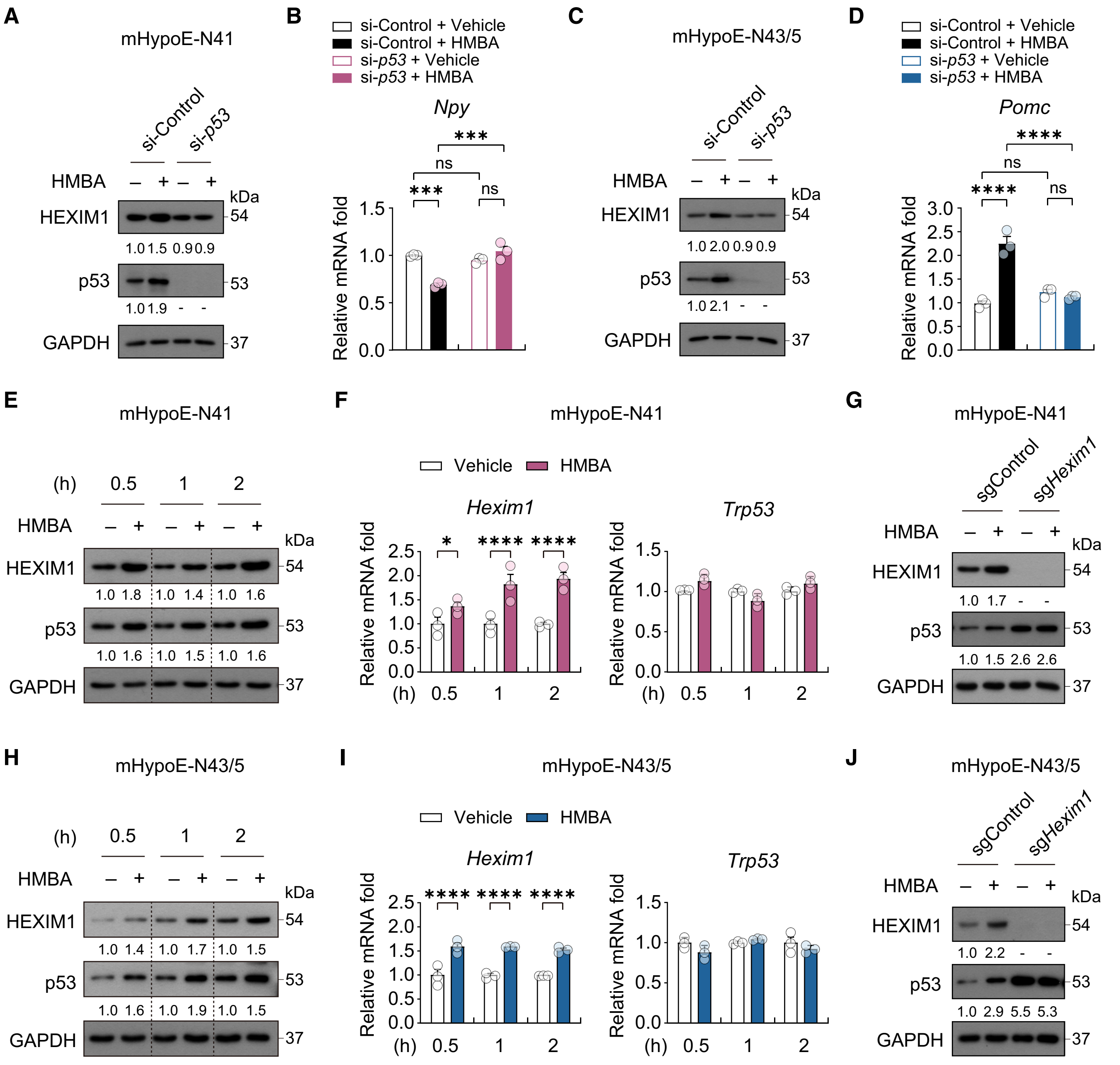

Figure 6. HMBA increases the levels of HEXIM1 and p53, affecting neuropeptide gene expression.

A Western blot analysis of HEXIM1 and p53 after HMBA treatment for 2 h with or without *p53* KD (si-*p53*) in mHypoE-N41 cells.
B Relative mRNA levels of *Npy*. $n = 3$ per group.
C Western blot analysis of HEXIM1 and p53 after HMBA treatment with or without *p53* KD in mHypoE-N43/5 cells.
D Relative mRNA levels of *Pomc*. $n = 3$ per group.
E Western blot analysis of HEXIM1 and p53 after HMBA treatment in mHypoE-N41 cells.
F Relative mRNA levels of *Hexim1* and *p53* (*Trp53*). $n = 3$ per group.
G Western blot analysis of HEXIM1 and p53 after HMBA treatment in *Hexim1* KO (sg*Hexim1*).
H Western blot analysis of HEXIM1 and p53 after HMBA treatment in mHypoE-N43/5 cells.
I Relative mRNA levels of *Hexim1* and *p53* (*Trp53*). $n = 3$ per group.
J Western blot analysis of HEXIM1 and p53 after HMBA treatment in *Hexim1* KO (sg*Hexim1*).

Data information: Cells were treated with 0.1 mM HMBA. The datasets in qPCR experiments were comprised of three biological replicates and each biological replicate was an average of three technical replicates. The numbers under western blots indicated relative quantitative mean values of three biological replicates. Statistical significance was determined by two-way ANOVA followed by a *post hoc* Tukey test. *$P < 0.05$, ***$P < 0.001$, ****$P < 0.0001$; ns or unless otherwise stated, no significance. Data are mean ± SEM. The exact *P*-values are reported in Appendix Table S6.
Source data are available online for this figure.

 © 2023 The Authors

Npy and *Pomc* but was required for HMBA-induced changes in their expression. To further delineate the cooperative action of HEXIM1 and p53 in neuropeptide gene regulation in the presence of HMBA, we first assessed the effect of HMBA on HEXIM1 and p53 protein levels. In both cell lines, HMBA increased the levels of both proteins at the 0.5, 1, and 2 h time points (Fig 6E and H). HMBA also increased the mRNA levels of *Hexim1* but not those of *p53* (*Trp53*), suggesting that HMBA regulates p53 at the protein level only (Fig 6F and I).

Notably, the basal protein level of p53 was significantly increased by *Hexim1* deletion (Fig 6G and J). Therefore, p53 is under the control of HEXIM1 at basal state. The HMBA-induced increase in p53 protein level was abolished by *Hexim1* deletion, indicating that HMBA regulates p53 in a HEXIM1-dependent manner (Fig 6G and J). Despite an increase in basal p53 protein level, our cell lines did not undergo apoptosis, as indicated by the absence of increases in cleaved forms of caspases 3 and 9 (apoptosis markers) upon HMBA treatment or *Hexim1* KO (Appendix Fig S8). Taken together, these results suggest differential roles of HEXIM1 and p53 in mediation of the anorexigenic effects of HMBA. HEXIM1 regulates the expression of p53, *Npy*, and *Pomc* at basal state as well as upon HMBA treatment, whereas p53 participates in the gene regulation of *Npy* and *Pomc* when HMBA is administered.

HMBA induces translocation of HEXIM1 and p53 to the nucleus

To elucidate the mechanism by which HMBA regulates neuropeptide expression, we first assessed changes in the localization of HEXIM1 and p53 after 2 h HMBA treatment using confocal microscopy imaging and subcellular fractionation. While HMBA increased HEXIM1 levels in both the cytoplasm and nucleus, p53 was increased in the nucleus only (Fig 7A–C and Appendix Fig S9). HMBA-induced increase in nuclear p53 was abolished by *Hexim1* deletion (Fig 7A–C and Appendix Fig S9).

To delineate the role of HEXIM1 and p53 in the nucleus, we measured the levels of neuropeptide expression after treatment with 50 ng/ml leptomycin B (LMB), an inhibitor of nuclear export, to ensure protein retention in the nucleus. LMB alone did not affect the basal mRNA levels of *Npy* or *Pomc*. However, the HMBA-induced decrease in the expression of *Npy* and increase in that of *Pomc* were further exacerbated by LMB (Fig EV5). It was unclear whether there was any further increase in nuclear localization of HEXIM1 and p53 by LMB since they were located mainly in the nucleus in both cell lines (Fig EV5). These data suggest that nuclear HEXIM1 and p53 are important for HMBA to regulate the gene expression of *Npy* and *Pomc*.

The amounts of proteins bound to HEXIM1 were analyzed by immunoprecipitating HEXIM1 from cytoplasmic and nuclear fractions (Fig 7D). Although majority of HEXIM1 and p53 proteins were present in the nucleus, their interaction occurred mostly in the cytoplasm; this complex, however, dissociated upon HMBA treatment. In contrast, the abundance of the HEXIM1–p53 complex in the nucleus was low regardless of HMBA treatment (Fig 7D). Overexpressed HEXIM1 was reported to inhibit p53 degradation by binding to p53, which disturbs the interaction between p53 and E3 ubiquitin–protein ligase mouse double minute-2 (MDM2, called HDM2 in humans), which binds to p53 along with ubiquitin (Lew *et al*, 2012). HEXIM1 competes with MDM2 for binding to the negative regulatory domain of p53 (Lew *et al*, 2013). In the cytoplasm of mHypoE-N41 and mHypoE-N43/5 cells, HMBA increased HEXIM1 binding to MDM2 but decreased HEXIM1 binding to p53 (Fig 7D). Although cytoplasmic MDM2 was markedly induced by HMBA, MDM2 and HEXIM1 did not compete for binding to p53, but rather bound to each other. Interestingly, the p53–MDM2 complex was not formed either at basal state or upon HMBA treatment (Fig 7D). MDM2 expression was also governed by MYH9 and ACTG1, as silencing of *Myh9*, *Actg1*, or both prevented MDM2 induction upon HMBA treatment (Appendix Fig S10). Immunoprecipitation results suggest that HMBA boosts the competitive binding of HEXIM1 with MDM2 rather than with p53 in the cytoplasm. Overall, the above results indicate that HMBA treatment results in the disassembly of the HEXIM1–p53 complex and translocation of free HEXIM1 and p53 to the nucleus.

HMBA regulates neuropeptide transcription

To ascertain whether nuclear HEXIM1 and p53 directly regulate transcription of neuropeptide genes, we performed chromatin immunoprecipitation assay and analyzed the amounts of HEXIM1, p53, and histone modification markers bound to the promoter regions of *Npy* (Titolo *et al*, 2008; Melas *et al*, 2013) and *Pomc* (Wu *et al*, 2014). We observed binding of HEXIM1 and p53 to promoter

Figure 7. HMBA regulates the transcription of *Npy* and *Pomc* through HEXIM1, p53, and H3K9Ac.

A, B Immunofluorescence analysis of HEXIM1 and p53 after HMBA treatment in sgControl and sg*Hexim1* of (A) mHypoE-N41 and (B) mHypoE-N43/5, and signal intensity in the cytoplasm and nucleus. $n = 10$, sgControl group of both cell lines; $n = 7$, sg*Hexim1* group of both cell lines. Scale bars, 10 µm.

C Western blot analysis of HEXIM1 and p53 in the cytoplasmic and nuclear lysates of sgControl or sg*Hexim1* cells treated with HMBA.

D Immunoprecipitation (IP) analysis of cytoplasmic and nuclear lysates.

E Chromatin immunoprecipitation (ChIP) analysis of the *Npy* promoter. $n = 3$ per group.

F ChIP analysis of the *Pomc* promoter. $n = 3$ per group.

G Schematic diagram suggesting the mechanism of the regulation of neuropeptide transcription by HMBA; HMBA binds to MYH9 and ACTG1, inducing HEXIM1 and MDM2 expression. HEXIM1 competitively interacts with MDM2 rather than p53, resulting in dissociation of the HEXIM1–p53 complex in the cytoplasm. The dissociated HEXIM1 and p53 translocate to the nucleus. Nuclear p53 and HEXIM1 bind to the *Npy* promoter, which negatively regulates *Npy* transcription, whereas nuclear p53 and H3K9Ac bind to the *Pomc* promoter, which positively regulates *Pomc* transcription.

Data information: Cells were treated with 0.1 mM HMBA for 2 h. For IP or ChIP analysis, 200 µg of total protein was used. The datasets in qPCR experiments were comprised of three biological replicates and each biological replicate was an average of three technical replicates. The numbers under western blots indicated relative quantitative mean values of three biological replicates. Statistical significance was determined by two-way ANOVA followed by a *post hoc* Tukey test. *$P < 0.05$, ****$P < 0.0001$; – or nd, not detected. Data are mean ± SEM. The exact P-values are reported in Appendix Table S6.
Source data are available online for this figure.

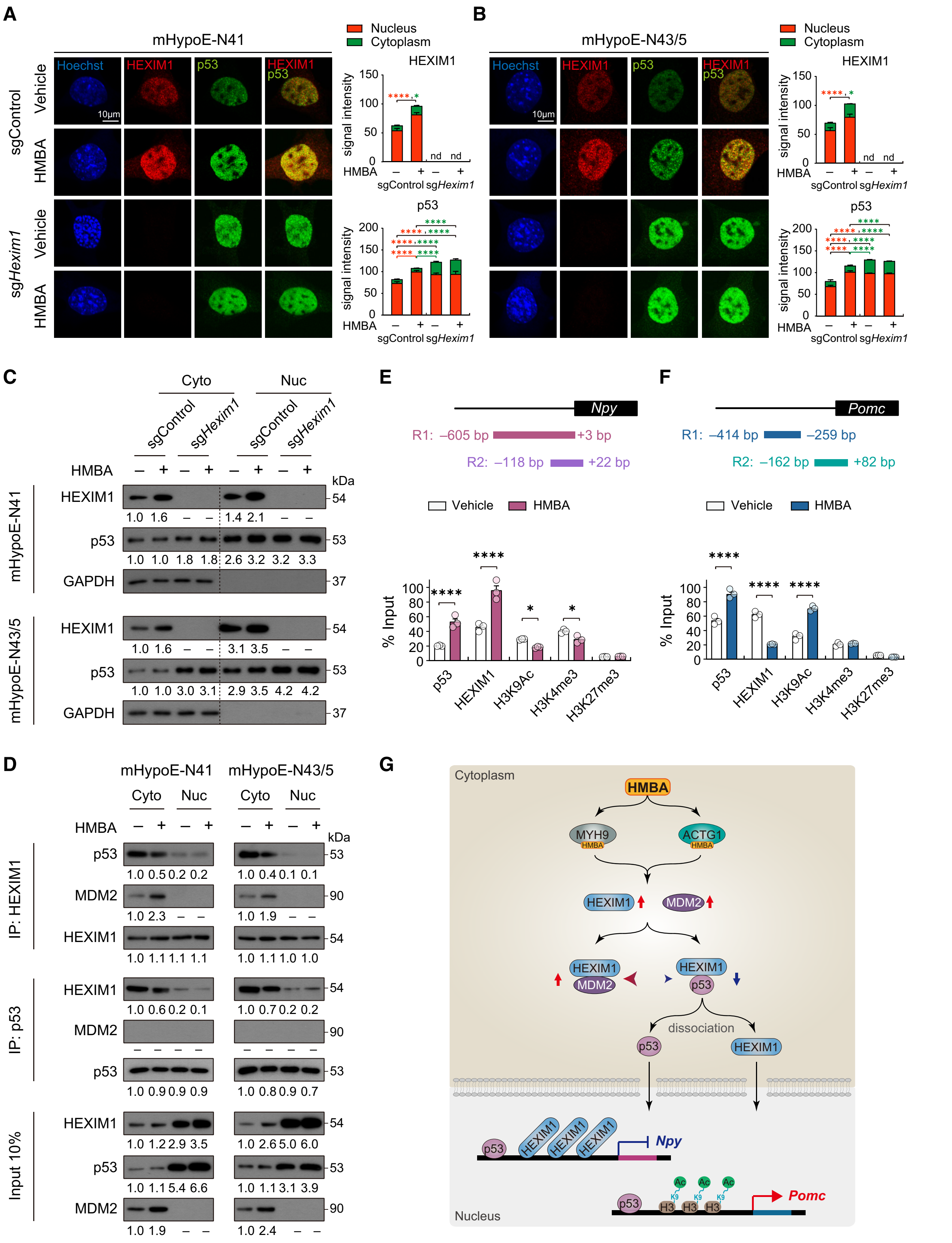

Figure 7.

© 2023 The Authors

regions 1 (R1) but not to promoter regions 2 (R2) of *Npy* and *Pomc* (Appendix Fig S11). HMBA increased the binding of both p53 and HEXIM1 to *Npy* promoter R1 (hereafter referred to as the *Npy* promoter) (Fig 7E). HMBA also increased the binding of p53 but decreased that of HEXIM1 to *Pomc* promoter R1 (hereafter referred to as the *Pomc* promoter) (Fig 7F). These data suggest different roles of HEXIM1 and p53 in the expression of *Npy* and *Pomc* upon HMBA treatment. Binding of HEXIM1 to the *Npy* or *Pomc* promoter was inversely related to their activity following HMBA treatment, for example, increased binding of HEXIM1 to the *Npy* promoter decreased the expression of *Npy* and vice versa for *Pomc*, implying a repressive role of HEXIM1. In the absence of HMBA, however, *Hexim1* KD or KO downregulated the expression of *Npy* and upregulated that of *Pomc* expression, suggesting that additional regulatory factors may be involved in the multifaceted regulation upon HMBA treatment.

Epigenetic modifications such as histone acetylation regulate *Npy* and *Pomc* (Benite-Ribeiro *et al*, 2016). In obese rats displaying increased *Npy* and decreased *Pomc* expression, the binding of histone 3 acetylated at lysine 9 (H3K9Ac) to the *Npy* promoter is increased but that to *Pomc* promoter is decreased in comparison with non-obese rats, whereas there is no change in H3K9 methylation in either gene (Mahmood *et al*, 2013). We assessed H3K9Ac in the *Npy* and *Pomc* promoters. We also evaluated histone 3 trimethylated at lysine 4 (H3K4me3) and at lysine 27 (H3K27me3), which indicate transcriptional activation and repression, respectively (Voigt *et al*, 2013). HMBA reduced the binding levels of H3K9Ac and H3K4me3 to the *Npy* promoter and elevated that of H3K9Ac to the *Pomc* promoter. The binding levels of H3K27me3 were marginal and were not changed by HMBA in either promoter (Fig 7E and F). Our data suggest that HMBA induced differential binding of HEXIM1 and H3K9Ac to the *Npy* and *Pomc* promoters, leading to opposite outcomes in gene expression.

Overall, our data suggest the following sequential events: (i) HMBA binds to MYH9 and ACTG1, inducing HEXIM1 and MDM2 expression; (ii) in the cytoplasm, HEXIM1 competitively binds to MDM2 rather than to p53, resulting in a release of HEXIM1 and p53 from the HEXIM1–p53 complex, and the free cytoplasmic HEXIM1 and p53 translocate to the nucleus; (iii) in the nucleus, p53 and HEXIM1 inhibit the transcription of *Npy*, whereas p53 and H3K9Ac activate that of *Pomc*, which results in the anorexigenic and anti-obese effects of HMBA (Fig 7G).

Discussion

Since the first synthesis of HMBA more than 4 decades ago (Reuben *et al*, 1976), research on this compound has focused on its applications in oncology, but it failed in a phase II clinical trial as an anticancer drug due to toxicity at high dosages (Andreeff *et al*, 1992; Conley *et al*, 1992). HMBA has a short biological half-life, as it is rapidly excreted into the urine and is quickly eliminated from the body, which limits the duration of its anti-cancer efficacy (Egorin *et al*, 1987a,b; Litterst *et al*, 1985). Treatment of tumors required a high concentration of HMBA to induce apoptosis or cell differentiation, for example, patients were recommended an intravenous infusion of 4,000 mg/kg for 10 consecutive days, repeated for at least 7 cycles with 4-week intervals (Young *et al*, 1988; Andreeff *et al*, 1992). Our study investigated the anti-obesity efficacy of peripheral and central injection of HMBA in DIO mice under milder conditions, that is, lower dosage and a shorter period of injection than in cancer studies.

Our results showed that HMBA-injected mice were resistant to the obesogenic effects of an HFD. Every other day i.v. or daily i.p. injections of HMBA (≤1,000 mg/kg; 4 or 7 injections, respectively) were effective in suppressing appetite and weight gain, while concentrations below 500 mg/kg were not (Appendix Fig S12). Daily i.c.v. injections of HMBA (400 nmoles; 7 injections) improved the obese phenotype and abnormal metabolic parameters, and even at a lower dose of 80 nmoles, food intake and body weight were reduced (Appendix Fig S12). Consistent with the *in vivo* data and unlike the high concentrations of HMBA (5 mM) required for cell differentiating effects *in vitro* (Reuben *et al*, 1976; Guilbaud *et al*, 1990; Kondo *et al*, 1990), relatively low concentrations of HMBA (0.01–0.1 mM) were sufficient to change the mRNA levels of *Npy* and *Pomc* in hypothalamic cells. The amounts of HMBA accumulated in the hypothalamus after i.v., i.p., or i.c.v. injections indicate that not only centrally but also peripherally delivered HMBA reached the hypothalamus and served as an anti-obesity compound, highlighting the importance of HMBA as a potential small-molecule targeting the hypothalamus for the control of metabolic homeostasis.

In breast cancer cells, 70-kDa heat-shock protein (Lama *et al*, 2017) and lysine demethylase 5B (Montano *et al*, 2019) have been identified as proteins binding HMBA. In hypothalamic cells, we found robust binding of HMBA to MYH9 and ACTG1. MYH9 and ACTG1 contribute to the regulation of tumorigenesis-associated gene expression via multiple signaling cascades (Dong *et al*, 2018; Wang *et al*, 2019; Ye *et al*, 2020; Xiao *et al*, 2021). However, their roles in the hypothalamus or functions in the metabolism have not been studied. Our findings provide new insights into the function of MYH9 and ACTG important for mediating the effects of HMBA on metabolic regulation. Notably, we found that HMBA-induced HEXIM1, which is crucial for regulating energy balance, requires MYH9 and ACTG1 in the NPY-expressing and POMC-expressing neurons. Although the precise mechanism(s) by which MYH9 and ACTG1 regulate HEXIM1 and MDM2 expression is not discovered yet, it might rely, at least in part, on p53 stabilization through an increase in the level of the HEXIM1–MDM2 complex, which regulates *Npy* and *Pomc* expression.

HEXIM1 was proposed as a mediator of HMBA actions in cancer cells (He *et al*, 2006; Turano *et al*, 2006; Yoshikawa *et al*, 2012; Ketchart *et al*, 2013, 2016; Lama *et al*, 2017). Indeed, we observed that HMBA induced HEXIM1 in the hypothalamus, and genetic ablation of *Hexim1* abolished the effects of HMBA on neuropeptide expression. At the molecular level, HMBA enhanced the formation of the HEXIM1–MDM2 complex rather than the HEXIM1–p53 complex in the cytosol, released p53 from HEXIM1, and prevented MDM2-mediated degradation of p53. Although the knockdown results clearly suggest HEXIM1, not p53, as the main regulator of the transcriptional control of neuropeptides under both basal and HMBA-treatment conditions in the hypothalamic cells, distinct regulation of *Npy* and *Pomc* may require other nuclear factors as well.

Upon HMBA treatment, the dissociated HEXIM1 and p53 translocated to the nucleus, where p53 coordinated the downregulation of *Npy* gene expression by binding to the promoter and probably forming a complex with a corepressor, for example, histone

© 2023 The Authors

deacetylase 3 (Kim *et al*, 2021), which would decrease binding of H3K9Ac to the *Npy* promoter. The reduction in H3K4me3 levels may also contribute to the downregulation of *Npy* expression. In contrast, p53 likely upregulates *Pomc* gene expression by binding to its promoter and inducing acetylation of H3K9, given the observation that p53 can induce acetylation of histone 3 or 4 (Barlev *et al*, 2001). Intriguingly, binding of HEXIM1 to the *Pomc* promoter was decreased after HMBA treatment despite an increase in nuclear HEXIM1, suggesting that HEXIM1 may facilitate *Pomc* transcription without binding to its promoter. HMBA induces HEXIM1 phosphorylation by activating the PI3K/Akt pathway, and this phosphorylation releases P-TEFb from the inactive complex with HEXIM1 and 7SK small nuclear RNA and leads to subsequent recruitment of P-TEFb to the HIV promoter to stimulate transcription in HIV-infected cells (Contreras *et al*, 2007). Hence, further studies are warranted to investigate whether other factors are involved in the regulation of *Npy* and *Pomc* genes by HMBA.

Several pharmacotherapies targeting gut-derived peptides, intestinal lipase, 5-hydroxytryptamine receptor, and β3 adrenergic receptor have been developed to prevent or treat obesity (Bhat & Sharma, 2017). Despite efforts to lessen the burden of metabolic diseases, however, the prevalence of overweight or obesity is increasing globally. This study points to a previously unappreciated effect of HMBA in the regulation of hypothalamic neuropeptides by which it changes whole-body energy homeostasis. Our understanding of the mode of action of HMBA and its mechanisms underlying the phenotypic changes in appetite, energy expenditure, and overall energy balance might provide ideas and platforms for identification and development of a new chemotype of anti-obesity compounds.

Materials and Methods

Chemicals and reagents

Hexamethylene bisacetamide (HMBA) was purchased from Abcam (142438). N-acetyl-1,6-diaminohexane (NADAH) was purchased from Angene (AG00E3KH). 1,6-diaminohexane (DAH; A14212) and 6-aminohexanoic acid (AmHA; A14719) were purchased from Thermo Scientific. Leptomycin B (L2913), N-methyl-N-(trimethylsilyl) trifluoroacetamide (MSTFA; Supelco 69479), and BCl_3-methanol (12%, w/w; Supelco 91379) were purchased from Sigma-Aldrich. HPLC-grade water (W6) and acetonitrile (A996) were purchased from Thermo Scientific. Formic acid (Supelco 5.33002) was purchased from Merck.

Animal studies

C57BL/6N male and female mice at 4 weeks of age were purchased from Koatech (Pyeongtaek, Republic of Korea). NPY-hrGFP (B6.FVB-Tg(Npy-hrGFP)1Lowl/J), POMC-hrGFP mice (FVB-Tg (Pomc1-hrGFP)1Lowl/J), AgRP-IRES-Cre (AgRP^{Cre}; Agrp$^{tm1(cre)Lowl}$/J), and POMC-Cre (POMC^{Cre}; Tg(Pomc1-cre)16Lowl/J) male mice were obtained from The Jackson Laboratory. NPY-hrGFP and POMC-hrGFP mice were maintained hemizygous for the hrGFP transgene by crossing with C57BL/6J mice. Adult male and female mice were housed in a specific pathogen-free animal facility in individually ventilated cages under a 12-h light/dark cycle (light: 7:00 am to 7:00 pm; dark: 7:00 pm to 7:00 am) at 22 ± 3°C and humidity controlled at 50 ± 10%. Mice had *ad libitum* access to water and were fed an NCD (12% kcal from fat; Lab Supply, 5053) or HFD (60% kcal from fat; Envigo, 06414) for 4 weeks. Food intake per cage and individual body weight were measured daily. All animal studies followed the National Institutes of Health guidelines and were approved by the Institutional Animal Care and Use Committee at the Daegu Gyeongbuk Institute of Science and Technology Laboratory Animal Resource Center (approval number DGIST-IACUC-21112513).

Hypothalamic cell line culture

The embryonic mouse hypothalamic cells lines mHypoE-N41 (N41, neuropeptide Y-expressing cells; Cellutions Biosystems) and mHypoE-N43/5 (N43/5, pro-opiomelanocortin-expressing cells; Cellutions Biosystems) were maintained in DMEM (Sigma-Aldrich, D5796) with 10% fetal bovine serum (TCB, 101) and 1% penicillin and streptomycin (HyClone, SV30010) at 37°C. Cell lines were tested for mycoplasma by Cellutions Biosystems (no authentication).

Gene knockout by CRISPR

The CRISPR-Cas9 system was used to delete the *Hexim1* gene from hypothalamic cells. The small guide RNA for *Hexim1* gene inactivation (5′-GGCACCCGTTCCTCCGCGCTTGG-3′; 23 bp, reverse direction) was designed in exon 1 and was synthesized by ToolGen (Seoul, Republic of Korea). Hypothalamic cells were transfected with the Cas9 and small guide RNA plasmids (1:4 ratio) using TurboFect (Thermo Scientific, R0534). Homogenous *Hexim1* knockout was achieved by selection using 1 mg/ml hygromycin B (Enzo Life Science, ALX-380-306-G001) at 36 h after transfection, followed by subculture in fresh media. To validate the knockout, genomic DNA was extracted using a G-spin Total DNA Extraction Mini Kit (iNtRON Biotechnology, 17046). The gDNA concentration was determined using a NanoDrop spectrophotometer (Thermo Scientific). PCR was performed with 100 ng of gDNA using Ex Taq DNA polymerase (TaKaRa, RR001C). Primer sequences are listed in Appendix Table S3. The PCR products were sequenced at Bionics. Multiple-sequence alignment was performed using blastn at NCBI and 4peaks software (Nucleobytes). The *Hexim1* knockout efficiency was verified using western blotting.

Gene knockdown by siRNA transfection

ON-TARGETplus siRNAs for control (D-001810-10-20), *Hexim1* (L-053085-01-0020), and *Trp53* (L-040642-00-0010) were purchased from Dharmacon. Pre-designed siRNAs for *Myh9* (Myh9-Mus-5632) and *Actg1* (Actg1-Mus-432) were purchased from Bionics (Seoul, Republic of Korea). Cells were transfected with siRNAs (100 nM) for 48 h using Lipofectamine 3000 (Invitrogen). The knockdown efficiency was verified by western blotting or real-time quantitative reverse-transcription PCR (qRT–PCR).

Intraperitoneal (i.p.) or intravenous (i.v.) injections of HMBA

HMBA (diluted in saline; 1,000 mg/kg) or saline was injected into mice daily i.p. for 7 days. In the case of i.v. injection, mice were

 © 2023 The Authors

initially injected with 500 mg/kg of HMBA into the tail vein on day 1, followed by three injections of 1,000 mg/kg bi-daily. Administration was performed before the beginning of the dark cycle (4:30 pm). After HMBA injection, body weight and food consumption changes were measured over 10 days. Mice were housed in individually ventilated cages and had *ad libitum* access to water and food. To determine relative mRNA expression and HMBA amounts using LC–MS/MS, the hypothalami and plasma were harvested 4 h after injection. Blood samples were taken from the facial vein using a lancet.

Cannula implantation

Mice at 8 weeks of age were anesthetized with an i.p. injection of a mixture of Zoletil 50 (30 mg/kg body weight; Virbac) and Rompun (10 mg/kg body weight; Bayer Korea) in saline. Mice were placed in a stereotaxic frame (Stoelting Co.), and a 26-gauge guide cannula (Plastic One, Dusseldorf, Germany) was implanted into the third ventricle (3 V) according to the following coordinates: −1.3 mm posterior to the bregma and −5.4 mm below the surface of the skull. The guide cannula was secured to the skull by dental cement (Dentsply Sirona, LAD010) and covered with a dust cap. Mice were allowed to recover for 10 days after surgery. Body weight and food intake were measured daily after recovery.

Intracerebroventricular (i.c.v.) injection of HMBA

HMBA (2 µl, diluted in saline; 400 nmoles) or saline were i.c.v. injected at a rate of 0.8 µl/min into the 3 V using a Hamilton syringe pump (KD Scientific, Legato series) before the beginning of the dark cycle (4:30 pm). Mice were housed in individually ventilated cages and had *ad libitum* access to water and food. Mice fed an NCD were injected daily for 10 days, and mice fed an HFD were injected daily for 7 days. To determine relative mRNA expression and HMBA amounts using LC–MS/MS, the hypothalami were harvested 4 h after injection. Plasma was collected from the facial vein.

Stereotaxic microinjection of adeno-associated virus (AAV)

Ultra-purified Cre-dependent AAV-FLEXon-shRNAs (shControl or sh*Myh9* + sh*Actg1*)-EGFP PHP.eB serotype viruses were purchased from VectorBuilder (Guangzhou, China) for silencing both MYH9 and ACTG1 genes of the AgRP^{Cre} or POMC^{Cre} DIO mice. Viral vectors are listed in Appendix Fig S7. AgRP^{Cre} or POMC^{Cre} DIO mice fed an HFD for 4 weeks were anesthetized by an i.p. injection of a mixture of Zoletil 50 (30 mg/kg body weight; Virbac) and Rompun (10 mg/kg body weight; Bayer Korea) in saline. Mice were placed in a stereotaxic frame (Stoelting Co.), and then the 2 µl of viruses were stereotaxically injected into the ARC using a 10 µl Hamilton syringe mounted to a microinjection pump (KD Scientific, Legato series) at a rate of 0.2 µl/min. Injection coordinates were 1.3 mm caudal to the bregma, 0.4 mm bilateral to the midline, and 5.8 mm below the pial surface. Mice were fed an HFD *ad libitum* during the 2-week recovery. After the recovery, mice were i.p. injected with HMBA daily for 7 days and body weight and food intake were monitored for a total of 10 days starting from the first day of injection. The transduction efficiency of the viral vectors was demonstrated via the expression of EGFP in the ARC using immunohistochemistry.

Body composition and indirect calorimetry

Body composition was measured using a nuclear magnetic resonance MiniSpec LF50 analyzer (Bruker) prior to metabolic measurements. Indirect calorimetry was performed when body weight monitoring after HMBA injections was completed. For indirect calorimetry measurements, mice were housed individually with free access to water and HFD in metabolic chambers of the Comprehensive Lab Animal Monitoring System (CLAMS; Columbus Instruments). Mice were allowed to acclimatize in metabolic chambers for 24 h. After acclimation, mice were injected with HMBA once i.v., but not i.c.v., and then metabolic parameters were collected over the next 24 h. Oxygen consumption (VO_2), carbon dioxide production (VCO_2), heat production, and locomotor activity were determined using an Oxymax System (Columbus Instruments). VO_2, VCO_2, and heat production were normalized to body weight. The respiratory exchange ratio (RER) was calculated as VCO_2/VO_2. Locomotor activity was determined by measuring interruptions of the infrared beams (*X*- and *Z*-axes of total ambulatory counts). Energy expenditure was analyzed by ANCOVA using body weight as covariate as previously described (Tschop *et al*, 2011; Muller *et al*, 2021).

Brown adipose tissue thermogenesis

The surface interscapular brown adipose tissue temperatures of DIO mice were recorded 3 days after the last HMBA injection using a FLIR infrared camera (FLIR Systems, E6). Infrared pictures of non-anesthetized mice were taken from a distance of 30 cm. Temperature was quantified using FLIR analysis software.

Glucose and insulin tolerance test

Glucose and insulin tolerance tests were performed in DIO mice after monitoring body weight and food intake from the last i.c.v. injection of HMBA. Mice were fasted overnight for glucose tolerance tests and 6 h for insulin tolerance tests. Glucose (2 g/kg; Sigma-Aldrich, G7021) or human insulin (1 U/kg; Sigma-Aldrich, I9278) was administrated i.p. Blood was collected from the tail vein and blood glucose was determined at 0, 15, 30, 60, 90, and 120 min after glucose or insulin injection. Glucose levels were determined using an Accu-Check II glucometer (Roche).

LABORAS

HMBA (diluted in saline; 1,000 mg/kg for i.p., 400 nmoles for i.c.v.) or saline was injected into mice daily i.p. or i.c.v. for 7 days. In the case of i.v. injection, mice were injected with 500 mg/kg of HMBA on day 1, followed by three injections of 1,000 mg/kg bi-daily. After the last dosing of HMBA (on day 7 for i.p. or i.c.v.; day 8 for i.v. at 4:30 pm), mice were placed in every single LABORAS cage and recorded for 24 h starting from the dark cycle at 7:00 pm. Mice activities including locomotion, climbing, rearing, grooming, eating, and drinking were recorded and automatically analyzed using the Laboratory Animal Behavior Observation Registration and Analysis System (LABORAS, Metris).

CTA test

Conditioned taste aversion (CTA) test was performed as previously described (Rosenblum *et al*, 1993; Berman & Dudai, 2001; Eisenberg

et al, 2003; Akirav et al, 2006; Slouzkey et al, 2013). Male DIO mice were housed in individually ventilated cages and were pre-trained for 2 consecutive days to get their water rations for 20 min once a day. On the conditioning day, water was replaced with 0.3 M sucrose during the 20 min access session (referred to as pre). Twenty minutes later, mice received lithium chloride (LiCl, 0.05 M, 2% body weight; i.p.) to induce malaise, or received HMBA (i.v., i.p., or i.c.v.) as unconditioned stimulus. On the next day, mice were given a retrieval session (referred to as post) entailing 20 min access to 0.3 M sucrose. All water bottles were weighted before and after each session (pre/post) to measure the total amount (g) of sucrose consumption.

LC–MS/MS analysis

Hypothalami were extracted with a mixture of 400 μl of 80% methanol and 300 μl of 80% acetonitrile and were homogenized using a MagNA Lyser (Roche). Samples were sonicated (10 cycles, 40 s each, medium intensity) using a Bioruptor sonicator (Diagenode) and centrifuged at 16,000 *g* for 15 min. Plasma (50 μl) was extracted with 800 μl of methanol:chloroform:water, 2:1:1 (v:v). The aqueous phase was evaporated to dryness under nitrogen gas (TurboVap, Biotage). Dried samples were reconstituted with 200 μl of buffer A (10 mM ammonium acetate with 0.1% formic acid in water) and filtered through a 0.2 μm syringe filter. LC–MS/MS analysis was carried out on an Agilent 1290 ultra-performance liquid chromatography system coupled with an Agilent 6490 triple quadrupole mass spectrometer. Samples (2 μl) were separated on a Phenomenex Synergi HydroRP column (150 mm × 2 mm, 4 μm, 80 Å) maintained at 45°C with 10 mM ammonium acetate with 0.1% formic acid in water as buffer A and acetonitrile as buffer B. The flow rate was 0.3 ml/min and the total run time was 9 min. The gradient was as follows: 0–2 min at 100% buffer A; 2–5 min, linear gradient to 90% buffer B; 5–7 min, 90% buffer B; 7–7.1 min, return to the initial conditions; and 7.1–9 min, re-equilibration. The system was run-in multiple-reaction monitoring mode using optimized collision energy with positive electrospray ionization mode. The precursor-to-fragment ion transitions were selected according to Smith et al (2011), and NADAH, DAH, and AmHA were selected on the basis of our mass spectrometry settings (Appendix Table S2).

GC–MS/MS analysis

Serum (50 μl) underwent sonication using cold methanol:chloroform:water solution in a ratio of 2:1:1 (v/v/v), which included an internal standard (20 nM D2-oleate). Following sonication, the samples were subjected to centrifugation at 4°C for 10 min at 12,000 *g*. The organic phases were subsequently dried in a TurboVap evaporator (Biotage). To reconstitute the samples, they were dissolved in hexane containing 0.5 M KOH-MeOH and allowed to incubate at 25°C for 10 min. BCl_3 methanol (12% w/w) was then introduced, and the samples were heated at 70°C for 10 min, followed by rapid cooling on ice for 5 min. Next, a mixture of hexane and water in a 2:1 (v/v) ratio was added to the samples, which were then vortexed. Finally, the supernatants were transferred into autosampler vials. To quantify free fatty acids in serum, GC–MS/MS analysis was carried out in an Agilent 7000B gas chromatography system coupled with an Agilent 7000C triple quadrupole mass spectrometer. Samples were separated on an Ultra HP-5 ms capillary column (30 m × 0.25 μm, i.d., 0.25 μm film thickness, Agilent J&W Scientific). The instrument temperature was set as follows: inlet, 250°C; transfer line, 290°C; ion source, 230°C; and quadrupoles, 150°C. The capillary voltage was set at 70 eV. The gradient was run at a flow rate of 1.2 ml He/min and 4°C/min with 50°C up to 240°C. Metabolomics data were processed using the Mass Hunter software package (Agilent Technologies).

Triglyceride analysis

Serum triglyceride levels were measured using the Triglyceride Quantification Colorimetric/Fluorometric Kit (BioVision, K622-100) after the completion of body weight and food intake measurements after the last i.c.v. injection of HMBA. Mice were fasted overnight for 16 h. Blood was collected through the facial vein. Serum (2 μl) was mixed with 50 μl of assay buffer containing 0.4 μl/well of the probe and 2 μl of TG enzyme mix. Lipase reaction was allowed to proceed at room temperature for 60 min. Fluorescence was measured at Ex/Em = 545/590 nm.

Biotinylation of HMBA

A 10 mM solution of EZ-Link Sulfo-NHS-Biotin reagent (Thermo Scientific, 21217) was reacted with 1 mM solution of NADAH at room temperature for 30 min. The mass-to-charge ratios of biotinylated HMBA ($C_{18}H_{32}N_4O_3S$ $[M + H]^+$) and NADAH ($C_8H_{18}N_2O$ $[M + H]^+$) were calculated using ChemDraw 20.0 as 385.2 and 158.9, respectively, and were confirmed by LC–MS/MS. LC–MS/MS was carried out on an Agilent 1290 ultra-performance liquid chromatography system coupled with an Agilent 6490 triple quadrupole mass spectrometer. Samples (2 μl) were separated on a Phenomenex Synergi HydroRP column (150 mm × 2 mm, 4 μm, 80 Å) maintained at 45°C with 10 mM ammonium acetate with 0.1% formic acid in water as buffer A and acetonitrile as buffer B. The flow rate was 0.3 ml/min and the total run time was 9 min. The gradient was as follows: 0–2 min at 100% buffer A; 2–5 min, linear gradient to 90% buffer B; 5–7 min, 90% buffer B; 7–7.1 min, return to the initial conditions; and 7.1–9 min, re-equilibration. The system was run in multiple-reaction monitoring mode using optimized collision energy with positive electrospray ionization mode. The precursor-to-fragment ion transitions of biotinylated HMBA and NADAH were selected on the basis of our mass spectrometry settings (Appendix Table S2). All the NMR spectra were recorded on a Bruker 400 MHz spectrometer in DMSO-d_6. Chemical shifts (δ) for ^1H NMR spectra were analyzed using Mnova NMR 14 software (Mestrelab Research) and are reported in parts per million. The final mixture characterization was as follows. ^1H NMR (400 MHz, DMSO-d_6) δ 7.78 (dd, J = 23.4, 5.4 Hz, 3H), 6.42 (s, 1H), 6.36 (s, 1H), 4.34–4.26 (m, 1H), 4.16–4.08 (m, 1H), 3.66 (dd, J = 8.6, 2.5 Hz, 1H), 3.09 (ddd, J = 8.6, 6.2, 4.4 Hz, 1H), 2.99 (ttd, J = 7.5, 5.4, 2.6 Hz, 6H), 2.88–2.77 (m, 2H), 2.68–2.53 (m, 4H), 2.04 (t, J = 7.4 Hz, 2H), 1.77 (s, 6H), 1.67–1.54 (m, 0H), 1.49 (s, 2H), 1.53–1.40 (m, 1H), 1.40–1.32 (m, 10H), 1.29 (d, J = 7.0 Hz, 1H), and 1.24 (qd, J = 6.0, 2.5 Hz, 8H).

Biotin–neutravidin pull-down assay

Cells were sonicated (5 cycles, 15 s each, high intensity) in lysis buffer described above containing 1× protease and phosphatase

© 2023 The Authors

inhibitor cocktails but no Tris–HCl at 4°C using a Bioruptor sonicator. The cell lysate was incubated with biotinylated HMBA at room temperature for 1 h and then incubated with neutravidin resin (Thermo Scientific, 53150) for 30 min in a disposable column (Thermo Scientific, 29922). The mixture was centrifuged at 400 *g* for 1 min. The resin was washed with phosphate-buffered saline five times to remove non-specific binding. Proteins bound to biotinylated HMBA were eluted and denatured in 2× Laemmli sample buffer (Bio-Rad, 1610737) supplemented with 10 mM dithiothreitol, and electrophoresed on a 4–15% gradient SDS–PAGE (Bio-Rad, 4561084). The gel was visualized with InstantBlue Coomassie protein-staining solution (Abcam, ab119211).

LC–MS/MS for peptide analysis of HMBA-binding proteins

Coomassie blue-stained bands were in-gel digested with trypsin. Nano-LC–MS/MS analysis was performed with an Easy-nLC system connected to an LTQ Orbitrap XL mass spectrometer (Thermo Fisher San Jose) equipped with a nano-electrospray source. Samples were separated on an Agilent C18 Nanobore column (150 mm × 0.075 mm, 3 μm) with 0.1% formic acid and 3% acetonitrile in water as buffer A and 0.1% formic acid in acetonitrile as buffer B. The flow rate was 1 μl/min and the total run time was 100 min. The linear gradient was as follows: 0–80 min, 0 to 40% buffer B; 80–84 min, 40 to 60% buffer B; 84–88 min, 60 to 95% buffer B; 88–94 min, 95 to 100% buffer B; and 94–100 min, re-equilibration. Mass spectra were acquired using data-dependent acquisition with a full mass scan (m/z 350–1,200) followed by 10 MS/MS scans. For MS1 full scans, the orbitrap resolution was 15,000 and the automatic gain control value was 2×10^5. For MS/MS in the LTQ, the automatic gain control value was 1×10^4. Protein identification through peptide sequence matching was performed using the Mascot algorithm (Matrix Science). The criteria for database search were as follows: taxonomy, *Mus musculus*; fixed modification, carbamidomethylated cysteine residues; variable modification, oxidized methionine residues; maximum allowed missed cleavages, 2; MS tolerance, ± 10 ppm; MS/MS tolerance, 0.8 Da; and peptide filtration, a significance threshold of $P < 0.05$.

Computational protein–ligand docking modeling

The interactions between MYH9 or ACTG1 and HMBA were predicted by computational docking using AutoDock free open-source software (Forli *et al*, 2016). AutoDock was run using the AlphaFold predictions of MYH9 and ACTG1 (Q8VDD5 and P63260, respectively; taxonomy, *Mus musculus*) from UniProt (http://www.uniprot.org), and a chemical table file (hexamethylene bisacetamide, CID 3616) from PubChem (NCBI), with the following parameters: Lamarckian genetic algorithm runs, 300; the maximum number of energy evaluations, 30 million; and grid map setting points and spacing, 126 × 126 × 126 and 1 Å, respectively, in all dimensions. The top candidate docking model was used in this study. Additionally, we verified and visualized three-dimensional protein–ligand complexes using BIOVIA Discovery Studio (Dassault Systèmes, Release 2022), PLIP (Adasme *et al*, 2021), Protein Plus (Schoning-Stierand *et al*, 2020), and PyMOL 2.5.2; the two-dimensional diagrams of these complexes were generated by LIGPLOT$^+$ (Wallace *et al*, 1995).

Quantitative real-time PCR

Samples were prepared as previously reported (Park *et al*, 2019). Briefly, total RNA was isolated from mouse hypothalamus, brown adipose tissue, or cells with Trizol reagent (Invitrogen, 15596018). The RNA pellet was dissolved in nuclease-free water (Promega, P1195), and total RNA concentration was determined using a NanoDrop spectrophotometer (Thermo Scientific). cDNA was synthesized from 3 μg of RNA using GoScript Reverse Transcriptase (Promega, A5001). mRNA levels were quantified by qRT–PCR with a TB (SYBR) Green Premix Ex Taq II (TaKaRa, RR820L). Primer sequences are listed in Appendix Table S3. The mRNA levels were normalized to those of *Gapdh*.

Chromatin immunoprecipitation–quantitative PCR

Cells were cross-linked with 1.4% formaldehyde for 15 min at room temperature and then quenched with 125 mM glycine for 5 min. After harvesting the cells, cells were centrifuged at 16,000 *g* for 5 min. Cell pellet was resuspended in lysis buffer (150 mM NaCl, 50 mM Tris–HCl pH 7.5, 5 mM EDTA, 0.5% v/v NP-40, and 1% v/v Triton X-100) containing 1× protease and phosphatase inhibitor cocktails. To shear DNA, the lysate was sonicated (15 cycles, 30 s each, high intensity) at 4°C using a Bioruptor sonicator. After sonication, the samples were centrifuged at 16,000 *g* for 10 min and the supernatants were incubated overnight at 4°C with 2 μg of primary antibodies or control IgG and then reacted with Dynabeads Protein G (Invitrogen, 10007D) at 4°C for 2 h. DNA was eluted with elution buffer (1% SDS, 50 mM Tris pH 8.0, and 10 mM EDTA), and cross-linking was reversed with 200 mM NaCl, RNase A (10 mg/ml), and Proteinase K (20 mg/ml) at 65°C overnight. DNA was purified using a phenol:chloroform:isoamyl alcohol mixture (25:24:1, v/v) (Thermo Scientific, 15593031) and was quantified by qRT–PCR. Primer sequences for each promoter are listed in Appendix Table S4. The expression levels were normalized to the amount of input sample.

Immunofluorescence

Cells were fixed in 4% paraformaldehyde for 15 min at room temperature, washed three times with PBS, permeabilized in 0.05% Triton X-100 for 15 min at room temperature, rewashed with PBS, and blocked in 5% normal donkey serum with 1% bovine serum albumin for 1 h at room temperature. Cells were incubated overnight at 4°C with primary antibodies (HEXIM1, 1:150; p53, 1:150) in blocking buffer. Cells were washed three times with PBS, incubated with secondary antibodies (Cy3 to detect HEXIM1 and Alexa488 to detect p53; both 1:400) in blocking buffer for 1 h at room temperature, washed with PBS, and stained with Hoechst 33342 for 5 min at room temperature. Fluorescence images were captured using a Carl Zeiss LSM 780 or LSM 800 confocal laser-scanning microscope and analyzed in Zen software (Carl Zeiss). The list of antibodies is provided in Appendix Table S5. To measure the signal intensities of HEXIM1 or p53-stained cells, the nucleus/cytoplasm signals of individual cells per slide were analyzed using ImageJ software (National Institute of Health).

Immunohistochemistry

Mice were anesthetized with an i.p. injection of a mixture of Zoletil 50 (30 mg/kg body weight; Virbac) and Rompun (10 mg/kg body

© 2023 The Authors

weight; Bayer Korea) in saline. They were then perfused with PBS followed by 4% paraformaldehyde in PBS (pH 7.4) to fix the brain tissue. (i) Cryopreservation: the brains were extracted and post-fixed in 4% paraformaldehyde for 16 h at 4°C, then transferred to 30% sucrose in PBS. The brains were embedded in optimal cutting temperature compound (Leica) frozen on dry ice, and stored at −80°C. The brains were sliced (thickness: 40 μm), blocked in 5% normal donkey serum with 1% bovine serum albumin for 1 h at room temperature, washed three times with PBS, permeabilized in 0.05% Triton X-100 for 15 min at room temperature, rewashed with PBS, and blocked in 5% normal donkey serum with 1% bovine serum albumin for 1 h at room temperature. The brain slices were incubated overnight at 4°C with primary antibody (HEXIM1, 1:400) in blocking buffer. After washing, brain slices were incubated with secondary antibody (Cy3 to detect HEXIM1; 1:500) in blocking buffer for 2 h at room temperature and then washed and stained with Hoechst 33342 for 5 min at room temperature. (ii) Paraffin embedding: the brains were extracted and post-fixed in 4% paraformaldehyde for 9 h at 4°C, then dehydrated at 70–100% gradient ethanol for 15 min, washed with ultra-pure water for 10 min, and blocked in 5% normal donkey serum with 1% bovine serum albumin for 1 h at room temperature. The brain slices were incubated overnight at 4°C with primary antibody (MYH9 and ACTG1, 1:250) in blocking buffer. After washing, brain slices were incubated with secondary antibody (Alexa flour 647 to detect ACTG1 and 1:400; Cy3 to detect MYH9, 1:500) in blocking buffer for 2 h at room temperature and then washed and stained with Hoechst 33342 for 5 min at room temperature. The antibodies are listed in Appendix Table S5. Fluorescence images were taken with a Carl Zeiss LSM 800 confocal laser-scanning microscope and analyzed in Zen software (Carl Zeiss). To measure the numbers and intensities of GFP-positive or HEXIM1-stained cells, brain slices per mouse were analyzed using Fiji of ImageJ 2 software (National Institute of Health).

The paper explained

Problem

Obesity is considered a pandemic with an increasing prevalence worldwide. Lack of new knowledge of key components and mechanisms regulating metabolism is one of the obstacles to therapeutic designs for the treatment of obesity. Hypothalamic neuropeptides such as NPY and POMC are pivotal in regulating energy balance. Therefore, pharmacological interventions that can modulate their expressions are considered one of the promising strategies.

Results

Hexamethylene bisacetamide (HMBA) prevents diet-induced obesity by inducing hypophagia and increasing energy expenditure, leading to weight loss in mice. Of note, HMBA modulates the expression of hypothalamic neuropeptides NPY and POMC toward the direction of negative energy balance. HMBA binds to MYH9 and ACTG1 proteins, inducing HEXIM1 expression in NPY and POMC neurons. HMBA facilitates the interaction of HEXIM1 with MDM2, dissociating the HEXIM1–p53 complex, which leads to the downregulation of NPY and upregulation of POMC expression.

Impact

Our findings propose the molecular mechanisms by which HMBA-induced HEXIM1 regulates neuropeptide expression to affect systemic energy balance. In addition, our study may advance our understanding of hypothalamic control through the intervention of HMBA as an anti-obesity compound and suggest a new therapeutic strategy for the treatment of metabolic diseases such as obesity and diabetes.

Western blotting

Cells were lysed in a buffer solution composed of the following components: 50 mM Tris–HCl (pH 7.4), 250 mM sucrose, 5 mM sodium pyrophosphate, 1 mM EDTA, 1 mM EGTA, 1% Triton X-100 (obtained from Sigma-Aldrich, T8787), 0.1 mM benzamidine (obtained from Sigma-Aldrich, B6506), 1 mM dithiothreitol (obtained from Sigma-Aldrich, 43816), 0.5 mM phenylmethylsulfonyl fluoride (obtained from Sigma-Aldrich, P7626), 50 mM sodium fluoride, a protease inhibitor cocktail (purchased from Millipore, 535140), and a phosphatase inhibitor cocktail (obtained from Sigma-Aldrich, P5726). To determine protein concentrations, the Pierce BCA assay kit from Thermo Fisher Scientific (23228) was used. Next, 10 μg of lysates were separated using SDS–polyacrylamide gels and transferred onto polyvinylidene difluoride membranes from Millipore (IPVH00010). The membranes were subjected to a 35-min blotting process at 20 V in transfer buffer consisting of 25 mM Tris base and 192 mM glycine. Following this, the membranes were blocked for 1 h using a solution of 5% skim milk in 1× TBST buffer (20 mM Tris, 125 mM NaCl, and 0.1% Tween 20, obtained from Sigma-Aldrich, P1379) at pH 7.4. Primary antibodies were then applied and incubated either at room temperature for 1 h or at 4°C overnight. Subsequently, the membranes underwent three washes with 1× TBST and were subsequently incubated with secondary antibodies linked to horseradish peroxidase. The visualization of proteins was accomplished using either the Supersignal West Pico Chemiluminescent Substrate from Thermo Fisher Scientific (NCI4080KR) or the WesternBright ECL system from Advansta (K-12045-D50), following the manufacturer's guidelines. The antibodies are listed in Appendix Table S5. Blots were quantified using ImageJ software (National Institutes of Health), and the level of each protein was normalized to that of GAPDH.

Nuclear and cytoplasmic fractionation

Nuclear and cytoplasmic fractions of cells were obtained using an NE-PER Nuclear and Cytoplasmic Extraction Reagent Kit (Thermo Scientific, 78835).

Immunoprecipitation

Nuclear or cytoplasmic lysates (200 μg total protein) were diluted with lysis buffer (150 mM NaCl and 25 mM Tris–HCl pH 7.2) containing protease and phosphatase cocktail inhibitors. The samples were incubated overnight at 4°C with 2 μg of primary antibodies and then reacted with Pierce Protein A agarose beads (Thermo Scientific, 20333) at 4°C for 6 h. Bound proteins were eluted with 4× Laemmli sample buffer (Bio-Rad, #161-0737) containing 10% 2-mercaptoethanol (Sigma-Aldrich, M6250) and incubated at 95°C for 5 min. The supernatants were separated by SDS–PAGE, and the subsequent procedure was the same as for western blotting.

© 2023 The Authors

Statistical analysis and drawing chemical structures

GraphPad Prism software (v.10.0.2) was used for graphing and statistical analysis. For comparison between two groups, datasets were analyzed by a two-tailed unpaired Student's *t*-test. Multiple comparisons were analyzed by one- or two-way ANOVA (Tukey or Bonferroni *post hoc* test) to determine the statistical significance between groups. Differences in energy expenditure were calculated by ANCOVA using body weight as covariate. All quantitative data were represented as mean ± standard error of the mean (SEM). A value of $P < 0.05$ was considered significantly different. ChemDraw software (v.20.0) was used for drawing chemical structures. The sample size estimate was not determined. Animals were assigned to the groups randomly and no mice were excluded from the experiments. No blinding was done in the analysis.

Data availability

This study includes no data deposited in external repositories.

Expanded View for this article is available online.

Acknowledgement
This work was supported by the National Research Foundation of Korea (No. 2020R1A2B5B02001468 and 2023R1A2C1005739).

Author contributions
Seokjae Park: Conceptualization; formal analysis; validation; investigation; visualization; methodology; writing – original draft. **Sungjoon Oh:** Formal analysis; validation; methodology; writing – original draft. **Nayoun Kim:** Formal analysis; validation; methodology; writing – original draft. **Eun-Kyoung Kim:** Conceptualization; supervision; funding acquisition; methodology; project administration; writing – review and editing.

Disclosure and competing interests statement
The authors declare that they have no conflict of interest.

References

Adasme MF, Linnemann KL, Bolz SN, Kaiser F, Salentin S, Haupt VJ, Schroeder M (2021) PLIP 2021: expanding the scope of the protein-ligand interaction profiler to DNA and RNA. *Nucleic Acids Res* 49: W530–W534

Akirav I, Khatsrinov V, Vouimba RM, Merhav M, Ferreira G, Rosenblum K, Maroun M (2006) Extinction of conditioned taste aversion depends on functional protein synthesis but not on NMDA receptor activation in the ventromedial prefrontal cortex. *Learn Mem* 13: 254–258

Anderson KA, Ribar TJ, Lin F, Noeldner PK, Green MF, Muehlbauer MJ, Witters LA, Kemp BE, Means AR (2008) Hypothalamic CaMKK2 contributes to the regulation of energy balance. *Cell Metab* 7: 377–388

Andreeff M, Stone R, Michaeli J, Young CW, Tong WP, Sogoloff H, Ervin T, Kufe D, Rifkind RA, Marks PA (1992) Hexamethylene bisacetamide in myelodysplastic syndrome and acute myelogenous leukemia: a phase II clinical trial with a differentiation-inducing agent. *Blood* 80: 2604–2609

Barlev NA, Liu L, Chehab NH, Mansfield K, Harris KG, Halazonetis TD, Berger SL (2001) Acetylation of p53 activates transcription through recruitment of coactivators/histone acetyltransferases. *Mol Cell* 8: 1243–1254

Benite-Ribeiro SA, Putt DA, Soares-Filho MC, Santos JM (2016) The link between hypothalamic epigenetic modifications and long-term feeding control. *Appetite* 107: 445–453

Berman DE, Dudai Y (2001) Memory extinction, learning anew, and learning the new: dissociations in the molecular machinery of learning in cortex. *Science* 291: 2417–2419

Bhat SP, Sharma A (2017) Current drug targets in obesity pharmacotherapy – a review. *Curr Drug Targets* 18: 983–993

Bluher M (2019) Obesity: global epidemiology and pathogenesis. *Nat Rev Endocrinol* 15: 288–298

Brady LS, Smith MA, Gold PW, Herkenham M (1990) Altered expression of hypothalamic neuropeptide mRNAs in food-restricted and food-deprived rats. *Neuroendocrinology* 52: 441–447

Brandariz-Nunez D, Correas-Sanahuja M, Maya-Gallego S, Martin Herranz I (2021) Neurotoxicity associated with acyclovir and valacyclovir: a systematic review of cases. *J Clin Pharm Ther* 46: 918–926

Broadley KJ, Kelly DR (2001) Muscarinic receptor agonists and antagonists. *Molecules* 6: 142–193

Brothers SP, Wahlestedt C (2010) Therapeutic potential of neuropeptide Y (NPY) receptor ligands. *EMBO Mol Med* 2: 429–439

Callery PS, Egorin MJ, Geelhaar LA, Nayar MS (1986) Identification of metabolites of the cell-differentiating agent hexamethylene bisacetamide in humans. *Cancer Res* 46: 4900–4903

Conley BA, Egorin MJ, Sinibaldi V, Sewack G, Kloc C, Roberts L, Zuhowski EG, Forrest A, Van Echo DA (1992) Approaches to optimal dosing of hexamethylene bisacetamide. *Cancer Chemother Pharmacol* 31: 37–45

Contreras X, Barboric M, Lenasi T, Peterlin BM (2007) HMBA releases P-TEFb from HEXIM1 and 7SK snRNA via PI3K/Akt and activates HIV transcription. *PLoS Pathog* 3: 1459–1469

Dey A, Chao SH, Lane DP (2007) HEXIM1 and the control of transcription elongation: from cancer and inflammation to AIDS and cardiac hypertrophy. *Cell Cycle* 6: 1856–1863

Dey A, Wong E, Kua N, Teo HL, Tergaonkar V, Lane D (2008) Hexamethylene bisacetamide (HMBA) simultaneously targets AKT and MAPK pathway and represses NF kappaB activity: implications for cancer therapy. *Cell Cycle* 7: 3759–3767

Dhar-Mascareno M, Ramirez SN, Rozenberg I, Rouille Y, Kral JG, Mascareno EJ (2016) Hexim1, a novel regulator of leptin function, modulates obesity and glucose disposal. *Mol Endocrinol* 30: 314–324

Dong X, Han Y, Sun Z, Xu J (2018) Actin Gamma 1, a new skin cancer pathogenic gene, identified by the biological feature-based classification. *J Cell Biochem* 119: 1406–1419

Egorin MJ, Sigman LM, Van Echo DA, Forrest A, Whitacre MY, Aisner J (1987a) Phase I clinical and pharmacokinetic study of hexamethylene bisacetamide (NSC 95580) administered as a five-day continuous infusion. *Cancer Res* 47: 617–623

Egorin MJ, Zuhowski EG, Cohen AS, Geelhaar LA, Callery PS, Van Echo DA (1987b) Plasma pharmacokinetics and urinary excretion of hexamethylene bisacetamide metabolites. *Cancer Res* 47: 6142–6146

Egorin MJ, Snyder SW, Cohen AS, Zuhowski EG, Subramanyam B, Callery PS (1988) Metabolism of hexamethylene bisacetamide and its metabolites in leukemic cells. *Cancer Res* 48: 1712–1716

Eisenberg M, Kobilo T, Berman DE, Dudai Y (2003) Stability of retrieved memory: inverse correlation with trace dominance. *Science* 301: 1102–1104

Forli S, Huey R, Pique ME, Sanner MF, Goodsell DS, Olson AJ (2016) Computational protein-ligand docking and virtual drug screening with the AutoDock suite. *Nat Protoc* 11: 905–919

© 2023 The Authors

Fu J, Gaetani S, Oveisi F, Lo Verme J, Serrano A, Rodriguez De Fonseca F, Rosengarth A, Luecke H, Di Giacomo B, Tarzia G *et al* (2003) Oleylethanolamide regulates feeding and body weight through activation of the nuclear receptor PPAR-alpha. *Nature* 425: 90 – 93

Garcia de Yebenes E, Li S, Fournier A, St-Pierre S, Pelletier G (1995) Regulation of proopiomelanocortin gene expression by neuropeptide Y in the rat arcuate nucleus. *Brain Res* 674: 112 – 116

Gonzalez-Yanes C, Serrano A, Bermudez-Silva FJ, Hernandez-Dominguez M, Paez-Ochoa MA, Rodriguez de Fonseca F, Sanchez-Margalet V (2005) Oleylethanolamide impairs glucose tolerance and inhibits insulin-stimulated glucose uptake in rat adipocytes through p38 and JNK MAPK pathways. *Am J Physiol Endocrinol Metab* 289: E923 – E929

Guilbaud NF, Gas N, Dupont MA, Valette A (1990) Effects of differentiation-inducing agents on maturation of human MCF-7 breast cancer cells. *J Cell Physiol* 145: 162 – 172

Guzman M, Lo Verme J, Fu J, Oveisi F, Blazquez C, Piomelli D (2004) Oleoylethanolamide stimulates lipolysis by activating the nuclear receptor peroxisome proliferator-activated receptor alpha (PPAR-alpha). *J Biol Chem* 279: 27849 – 27854

He N, Pezda AC, Zhou Q (2006) Modulation of a P-TEFb functional equilibrium for the global control of cell growth and differentiation. *Mol Cell Biol* 26: 7068 – 7076

Hu Z, Cha SH, Chohnan S, Lane MD (2003) Hypothalamic malonyl-CoA as a mediator of feeding behavior. *Proc Natl Acad Sci USA* 100: 12624 – 12629

Huang Y, Lin X, Lin S (2021) Neuropeptide Y and metabolism syndrome: an update on perspectives of clinical therapeutic intervention strategies. *Front Cell Dev Biol* 9: 695623

Huszar D, Lynch CA, Fairchild-Huntress V, Dunmore JH, Fang Q, Berkemeier LR, Gu W, Kesterson RA, Boston BA, Cone RD *et al* (1997) Targeted disruption of the melanocortin-4 receptor results in obesity in mice. *Cell* 88: 131 – 141

Ketchart W, Smith KM, Krupka T, Wittmann BM, Hu Y, Rayman PA, Doughman YQ, Albert JM, Bai X, Finke JH *et al* (2013) Inhibition of metastasis by HEXIM1 through effects on cell invasion and angiogenesis. *Oncogene* 32: 3829 – 3839

Ketchart W, Yeh IJ, Zhou H, Thiagarajan PS, Lathia J, Reizes O, Exner A, Su B, Montano MM (2016) Induction of HEXIM1 activities by HMBA derivative 4a1: functional consequences and mechanism. *Cancer Lett* 379: 60 – 69

Kim EH, Koh DI, Ryu YS, Park SS, Hong SW, Moon JH, Shin JS, Kim MJ, Kim DY, Hong JK *et al* (2021) Role of p53 in transcriptional repression of SVCT2. *Mol Biol Rep* 48: 1651 – 1658

Kondo Y, Yura Y, Iga H, Yanagawa T, Yoshida H, Furumoto N, Sato M (1990) Effect of hexamethylene bisacetamide and cyclosporin A on recovery of herpes simplex virus type 2 from the *in vitro* model of latency in a human neuroblastoma cell line. *Cancer Res* 50: 7852 – 7857

Kusuhara M, Nagasaki K, Kimura K, Maass N, Manabe T, Ishikawa S, Aikawa M, Miyazaki K, Yamaguchi K (1999) Cloning of hexamethylene-bis-acetamide-inducible transcript, HEXIM1, in human vascular smooth muscle cells. *Biomed Res* 20: 273 – 279

Lama R, Gan C, Idippily N, Bobba V, Danielpour D, Montano M, Su B (2017) HMBA is a putative HSP70 activator stimulating HEXIM1 expression that is down-regulated by estrogen. *J Steroid Biochem Mol Biol* 168: 91 – 101

Lamb J, Crawford ED, Peck D, Modell JW, Blat IC, Wrobel MJ, Lerner J, Brunet JP, Subramanian A, Ross KN *et al* (2006) The Connectivity Map: using gene-expression signatures to connect small molecules, genes, and disease. *Science* 313: 1929 – 1935

Lew QJ, Chia YL, Chu KL, Lam YT, Gurumurthy M, Xu S, Lam KP, Cheong N, Chao SH (2012) Identification of HEXIM1 as a positive regulator of p53. *J Biol Chem* 287: 36443 – 36454

Lew QJ, Chu KL, Chia YL, Cheong N, Chao SH (2013) HEXIM1, a new player in the p53 pathway. *Cancers (Basel)* 5: 838 – 856

Li Z, Yi CX, Katiraei S, Kooijman S, Zhou E, Chung CK, Gao Y, van den Heuvel JK, Meijer OC, Berbee JFP *et al* (2018) Butyrate reduces appetite and activates brown adipose tissue via the gut-brain neural circuit. *Gut* 67: 1269 – 1279

Litterst CL, Roth JS, Kelley JA (1985) Distribution, elimination, metabolism and bioavailability of hexamethylenebisacetamide in rats. *Invest New Drugs* 3: 263 – 272

Loftus TM, Jaworsky DE, Frehywot GL, Townsend CA, Ronnett GV, Lane MD, Kuhajda FP (2000) Reduced food intake and body weight in mice treated with fatty acid synthase inhibitors. *Science* 288: 2379 – 2381

Mahmood S, Smiraglia DJ, Srinivasan M, Patel MS (2013) Epigenetic changes in hypothalamic appetite regulatory genes may underlie the developmental programming for obesity in rat neonates subjected to a high-carbohydrate dietary modification. *J Dev Orig Health Dis* 4: 479 – 490

Matias I, Vergoni AV, Petrosino S, Ottani A, Pocai A, Bertolini A, Di Marzo V (2008) Regulation of hypothalamic endocannabinoid levels by neuropeptides and hormones involved in food intake and metabolism: insulin and melanocortins. *Neuropharmacology* 54: 206 – 212

Melas PA, Lennartsson A, Vakifahmetoglu-Norberg H, Wei Y, Aberg E, Werme M, Rogdaki M, Mannervik M, Wegener G, Brene S *et al* (2013) Allele-specific programming of Npy and epigenetic effects of physical activity in a genetic model of depression. *Transl Psychiatry* 3: e255

Mickala P, Boutin H, Bellanger C, Chevalier C, MacKenzie ET, Dauphin F (1996) *In vivo* binding, pharmacokinetics and metabolism of the selective M2 muscarinic antagonists [3H]AF-DX 116 and [3H]AF-DX 384 in the anesthetized rat. *Nucl Med Biol* 23: 173 – 179

Montano MM, Yeh IJ, Chen Y, Hernandez C, Kiselar JG, de la Fuente M, Lawes AM, Nieman MT, Kiser PD, Jacobberger J *et al* (2019) Inhibition of the histone demethylase, KDM5B, directly induces re-expression of tumor suppressor protein HEXIM1 in cancer cells. *Breast Cancer Res* 21: 138

Muller TD, Klingenspor M, Tschop MH (2021) Revisiting energy expenditure: how to correct mouse metabolic rate for body mass. *Nat Metab* 3: 1134 – 1136

Myers MG Jr, Olson DP (2012) Central nervous system control of metabolism. *Nature* 491: 357 – 363

Myers MG Jr, Affinati AH, Richardson N, Schwartz MW (2021) Central nervous system regulation of organismal energy and glucose homeostasis. *Nat Metab* 3: 737 – 750

Park S, Oh TS, Kim S, Kim EK (2019) Palmitate-induced autophagy liberates monounsaturated fatty acids and increases Agrp expression in hypothalamic cells. *Anim Cells Syst (Seoul)* 23: 384 – 391

Poggioli R, Vergoni AV, Bertolini A (1986) ACTH-(1-24) and alpha-MSH antagonize feeding behavior stimulated by kappa opiate agonists. *Peptides* 7: 843 – 848

Quinones M, Al-Massadi O, Folgueira C, Bremser S, Gallego R, Torres-Leal L, Haddad-Tovolli R, Garcia-Caceres C, Hernandez-Bautista R, Lam BYH *et al* (2018) p53 in AgRP neurons is required for protection against diet-induced obesity via JNK1. *Nat Commun* 9: 3432

Reuben RC, Wife RL, Breslow R, Rifkind RA, Marks PA (1976) A new group of potent inducers of differentiation in murine erythroleukemia cells. *Proc Natl Acad Sci USA* 73: 862 – 866

Roseberry AG, Liu H, Jackson AC, Cai X, Friedman JM (2004) Neuropeptide Y-mediated inhibition of proopiomelanocortin neurons in the arcuate nucleus shows enhanced desensitization in ob/ob mice. *Neuron* 41: 711 – 722

Rosenblum K, Meiri N, Dudai Y (1993) Taste memory: the role of protein synthesis in gustatory cortex. *Behav Neural Biol* 59: 49 – 56

© 2023 The Authors

Schoning-Stierand K, Diedrich K, Fahrrolfes R, Flachsenberg F, Meyder A, Nittinger E, Steinegger R, Rarey M (2020) ProteinsPlus: interactive analysis of protein-ligand binding interfaces. *Nucleic Acids Res* 48: W48 – W53

Schwartz MW, Woods SC, Porte D Jr, Seeley RJ, Baskin DG (2000) Central nervous system control of food intake. *Nature* 404: 661 – 671

Shimokawa T, Kumar MV, Lane MD (2002) Effect of a fatty acid synthase inhibitor on food intake and expression of hypothalamic neuropeptides. *Proc Natl Acad Sci USA* 99: 66 – 71

Siegel DS, Zhang X, Feinman R, Teitz T, Zelenetz A, Richon VM, Rifkind RA, Marks PA, Michaeli J (1998) Hexamethylene bisacetamide induces programmed cell death (apoptosis) and down-regulates BCL-2 expression in human myeloma cells. *Proc Natl Acad Sci USA* 95: 162 – 166

Slouzkey I, Rosenblum K, Maroun M (2013) Memory of conditioned taste aversion is erased by inhibition of PI3K in the insular cortex. *Neuropsychopharmacology* 38: 1143 – 1153

Smith KM, Ketchart W, Zhou X, Montano MM, Xu Y (2011) Determination of hexamethylene bisacetamide, an antineoplastic compound, in mouse and human plasma by LC-MS/MS. *J Chromatogr B Analyt Technol Biomed Life Sci* 879: 2206 – 2212

Stanley BG, Leibowitz SF (1985) Neuropeptide Y injected in the paraventricular hypothalamus: a powerful stimulant of feeding behavior. *Proc Natl Acad Sci USA* 82: 3940 – 3943

Swinburn BA, Sacks G, Hall KD, McPherson K, Finegood DT, Moodie ML, Gortmaker SL (2011) The global obesity pandemic: shaped by global drivers and local environments. *Lancet* 378: 804 – 814

Tavares E, Maldonado R, Minano FJ (2007) N-procalcitonin: central effects on feeding and energy homeostasis in rats. *Endocrinology* 148: 1891 – 1901

Tavares E, Maldonado R, Minano FJ (2013) Aminoprocalcitonin-mediated suppression of feeding involves the hypothalamic melanocortin system. *Am J Physiol Endocrinol Metab* 304: E1251 – E1262

Titolo D, Mayer CM, Dhillon SS, Cai F, Belsham DD (2008) Estrogen facilitates both phosphatidylinositol 3-kinase/Akt and ERK1/2 mitogen-activated protein kinase membrane signaling required for long-term neuropeptide Y transcriptional regulation in clonal, immortalized neurons. *J Neurosci* 28: 6473 – 6482

Tschop MH, Speakman JR, Arch JR, Auwerx J, Bruning JC, Chan L, Eckel RH, Farese RV Jr, Galgani JE, Hambly C *et al* (2011) A guide to analysis of mouse energy metabolism. *Nat Methods* 9: 57 – 63

Turano M, Napolitano G, Dulac C, Majello B, Bensaude O, Lania L (2006) Increased HEXIM1 expression during erythroleukemia and neuroblastoma cell differentiation. *J Cell Physiol* 206: 603 – 610

Uhlen M, Fagerberg L, Hallstrom BM, Lindskog C, Oksvold P, Mardinoglu A, Sivertsson A, Kampf C, Sjostedt E, Asplund A *et al* (2015) Proteomics. Tissue-based map of the human proteome. *Science* 347: 1260419

Vohra MS, Benchoula K, Serpell CJ, Hwa WE (2022) AgRP/NPY and POMC neurons in the arcuate nucleus and their potential role in treatment of obesity. *Eur J Pharmacol* 915: 174611

Voigt P, Tee WW, Reinberg D (2013) A double take on bivalent promoters. *Genes Dev* 27: 1318 – 1338

Wallace AC, Laskowski RA, Thornton JM (1995) LIGPLOT: a program to generate schematic diagrams of protein-ligand interactions. *Protein Eng* 8: 127 – 134

Wang B, Qi X, Liu J, Zhou R, Lin C, Shangguan J, Zhang Z, Zhao L, Li G (2019) MYH9 promotes growth and metastasis via activation of MAPK/AKT signaling in colorectal cancer. *J Cancer* 10: 874 – 884

Wishart DS, Feunang YD, Marcu A, Guo AC, Liang K, Vazquez-Fresno R, Sajed T, Johnson D, Li C, Karu N *et al* (2018) HMDB 4.0: the human metabolome database for 2018. *Nucleic Acids Res* 46: D608 – D617

Wu Y, Patchev AV, Daniel G, Almeida OF, Spengler D (2014) Early-life stress reduces DNA methylation of the Pomc gene in male mice. *Endocrinology* 155: 1751 – 1762

Xiao L, Peng H, Yan M, Chen S (2021) Silencing ACTG1 expression induces prostate cancer epithelial mesenchymal transition through MAPK/ERK signaling pathway. *DNA Cell Biol* 40: 1445 – 1455

Yahagi N, Shimano H, Matsuzaka T, Najima Y, Sekiya M, Nakagawa Y, Ide T, Tomita S, Okazaki H, Tamura Y *et al* (2003) p53 activation in adipocytes of obese mice. *J Biol Chem* 278: 25395 – 25400

Ye G, Yang Q, Lei X, Zhu X, Li F, He J, Chen H, Ling R, Zhang H, Lin T *et al* (2020) Nuclear MYH9-induced CTNNB1 transcription, targeted by staurosporin, promotes gastric cancer cell anoikis resistance and metastasis. *Theranostics* 10: 7545 – 7560

Yik JH, Chen R, Nishimura R, Jennings JL, Link AJ, Zhou Q (2003) Inhibition of P-TEFb (CDK9/Cyclin T) kinase and RNA polymerase II transcription by the coordinated actions of HEXIM1 and 7SK snRNA. *Mol Cell* 12: 971 – 982

Yoshikawa N, Shimizu N, Maruyama T, Sano M, Matsuhashi T, Fukuda K, Kataoka M, Satoh T, Ojima H, Sawai T *et al* (2012) Cardiomyocyte-specific overexpression of HEXIM1 prevents right ventricular hypertrophy in hypoxia-induced pulmonary hypertension in mice. *PLoS One* 7: e52522

Young CW, Fanucchi MP, Declan Walsh T, Baltzer L, Yaldaei S, Stevens YW, Gordon C, Tong W, Rifkind RA, Marks PA (1988) Phase I trial and clinical pharmacological evaluation of hexamethylene bisacetamide administration by ten-day continuous intravenous infusion at twenty-eight-day intervals. *Cancer Res* 48: 7304 – 7309

Zhang Y, Cong Y, Teng Y (2016) Acute renal injury induced by valacyclovir hydrochloride: a case report. *Exp Ther Med* 12: 4025 – 4028

License: This is an open access article under the terms of the Creative Commons Attribution License, which permits use, distribution and reproduction in any medium, provided the original work is properly cited.

Expanded View Figures

Figure EV1. HMBA does not induce sickness behaviors in DIO mice.

A Behaviors of male DIO mice injected i.v. with HMBA. $N = 4$ per group.
B Locomotion (position distribution, distance traveled, and time of immobility) of i.v. injected group. Position distribution images were taken by integrating four individual mouse cages. $n = 4$ per group.
C Conditioned taste aversion test of i.v. injected group. $n = 5$ per group.
D Behaviors of male DIO mice injected i.p. with HMBA. $n = 4$ per group.
E Locomotion (position distribution, distance traveled, and time of immobility) of i.p. injected group. Position distribution images were taken by integrating four individual mouse cages. $n = 4$ per group.
F Conditioned taste aversion test of i.p. injected group. $n = 5$ per group.
G Behaviors of male DIO mice injected intracerebroventricular (i.c.v.) with HMBA. $n = 4$ per group.
H Locomotion (position distribution, distance traveled, and time of immobility) of i.c.v. injected group. Position distribution images were taken by integrating four individual mouse cages. $n = 4$ per group.
I Conditioned taste aversion test of i.c.v. injected group. $n = 5$ per group.

Data information: Data represent different numbers (n) of biological replicates. Data are represented in box and whisker plots where the central band denotes the median value, box contains interquartile ranges, while whiskers mark minimum and maximum values in panels (C), (F), and (I). The behaviors of mice were monitored immediately after the last dosing of HMBA i.v., i.p., or i.c.v. in LABORAS cages for 24 h. Mice received lithium chloride (LiCl, 0.05 M, 2% body weight; i.p.) to induce malaise, or received HMBA (i.v., i.p., or i.c.v.). Statistical significance was determined by a two-tailed unpaired Student's t-test in panels (A), (B), (D), (E), (G), and (H), and one-way ANOVA followed by a *post hoc* Tukey test in panels (C), (F), and (I). $^{*}P < 0.05$, $^{***}P < 0.001$, $^{****}P < 0.0001$. Data are mean ± SEM. The exact P-values are reported in Appendix Table S6.

 © 2023 The Authors

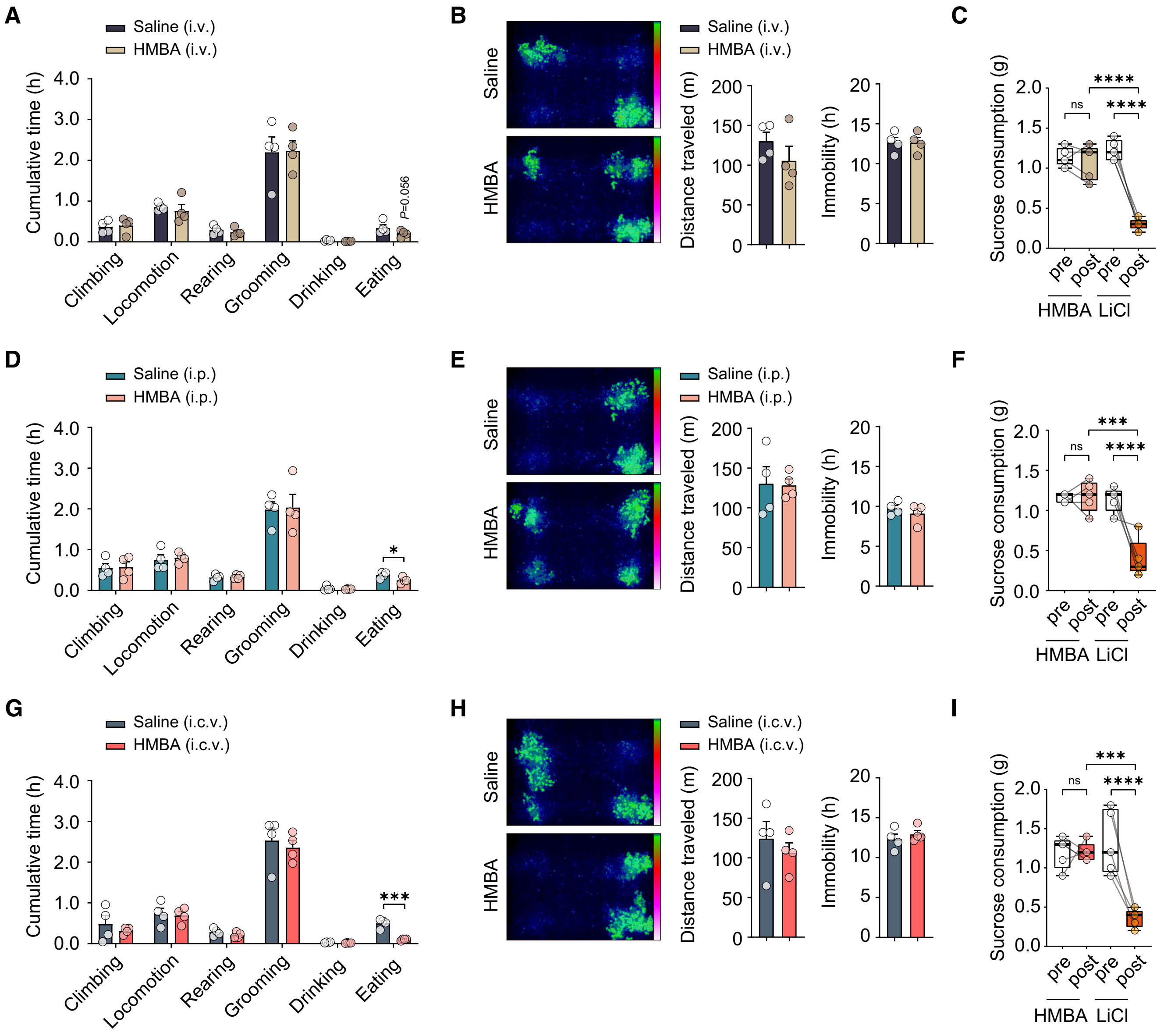

Figure EV1.

Figure EV2. HMBA complexes with MYH9 and ACTG1.

A, B The most energetically favored poses of HMBA in the potential binding pockets of (A) MYH9 and (B) ACTG1 were predicted by AutoDock v4.2.6 and were depicted in sphere view by Protein Imager. aa, amino acid sequence.

C, D Ligplot (two-dimensional docking models) analysis of (C) the MYH9–HMBA complex and (D) ACTG1–HMBA complex. HMBA is shown in yellow. Hydrogen bonds are indicated by dashed green lines with the distance (Å) from the donor atom to the acceptor atom indicated. Hydrophobic interactions are shown by red spokes radiating toward the ligand atoms they contact, and blue spokes indicate hydrophobic interactions with potential hydrogen bonds. C, N, and O atoms are shown in black, blue, and red, respectively.

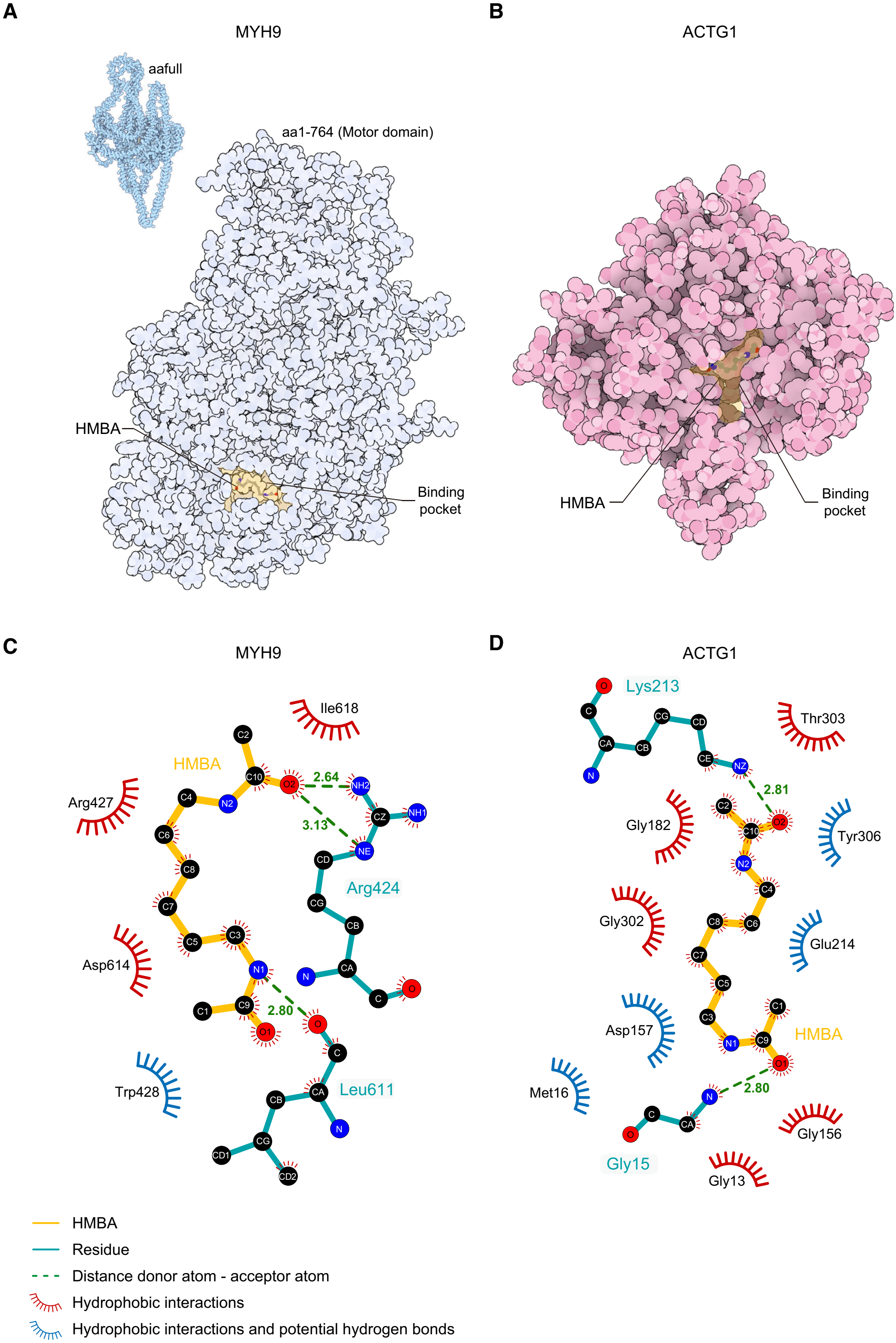

Figure EV2.

© 2023 The Authors

Figure EV3. MYH9 and ACTG1 are required for the regulation of neuropeptide expression by HMBA.

A, B Knockdown efficiency of *Myh9* (si-*Myh9*) and *Actg1* (si-*Actg1*) assessed from relative mRNA levels in (A) mHypoE-N41 (related to Fig 4I–L) and (B) mHypoE-N43/5 (related to Fig 4M–P). $n = 3$ per group.

C Relative mRNA levels of *Myh9*, *Actg1*, and *Hexim1* in *Myh9* and *Actg1* double-knockdown (si-*Myh9* + si-*Actg1*) mHypoE-N41. $n = 3$ per group.

D Western blot analysis of *Myh9* and *Actg1* double-knockdown mHypoE-N41.

E Relative mRNA levels of *Npy* in *Myh9* and *Actg1* double-knockdown mHypoE-N41. $n = 3$ per group.

F Relative mRNA levels of *Myh9*, *Actg1*, and *Hexim1* in *Myh9* and *Actg1* double-knockdown mHypoE-N43/5. $n = 3$ per group.

G Western blot analysis of *Myh9* and *Actg1* double-knockdown mHypoE-N43/5.

H Relative mRNA levels of *Pomc* in *Myh9* and *Actg1* double-knockdown mHypoE-N43/5. $n = 3$ per group.

Data information: After single or double knockdown, cells were treated or not with 0.1 mM HMBA for 2 h. The datasets in qPCR experiments were comprised of three biological replicates and each biological replicate was an average of three technical replicates. Western blots were repeated twice independently with similar results. Statistical significance was determined by two-way ANOVA followed by a *post hoc* Tukey test. $^{**}P < 0.01$, $^{***}P < 0.001$, $^{****}P < 0.0001$; ns or unless otherwise stated, no significance. Data are mean ± SEM. The exact *P*-values are reported in Appendix Table S6.

© 2023 The Authors

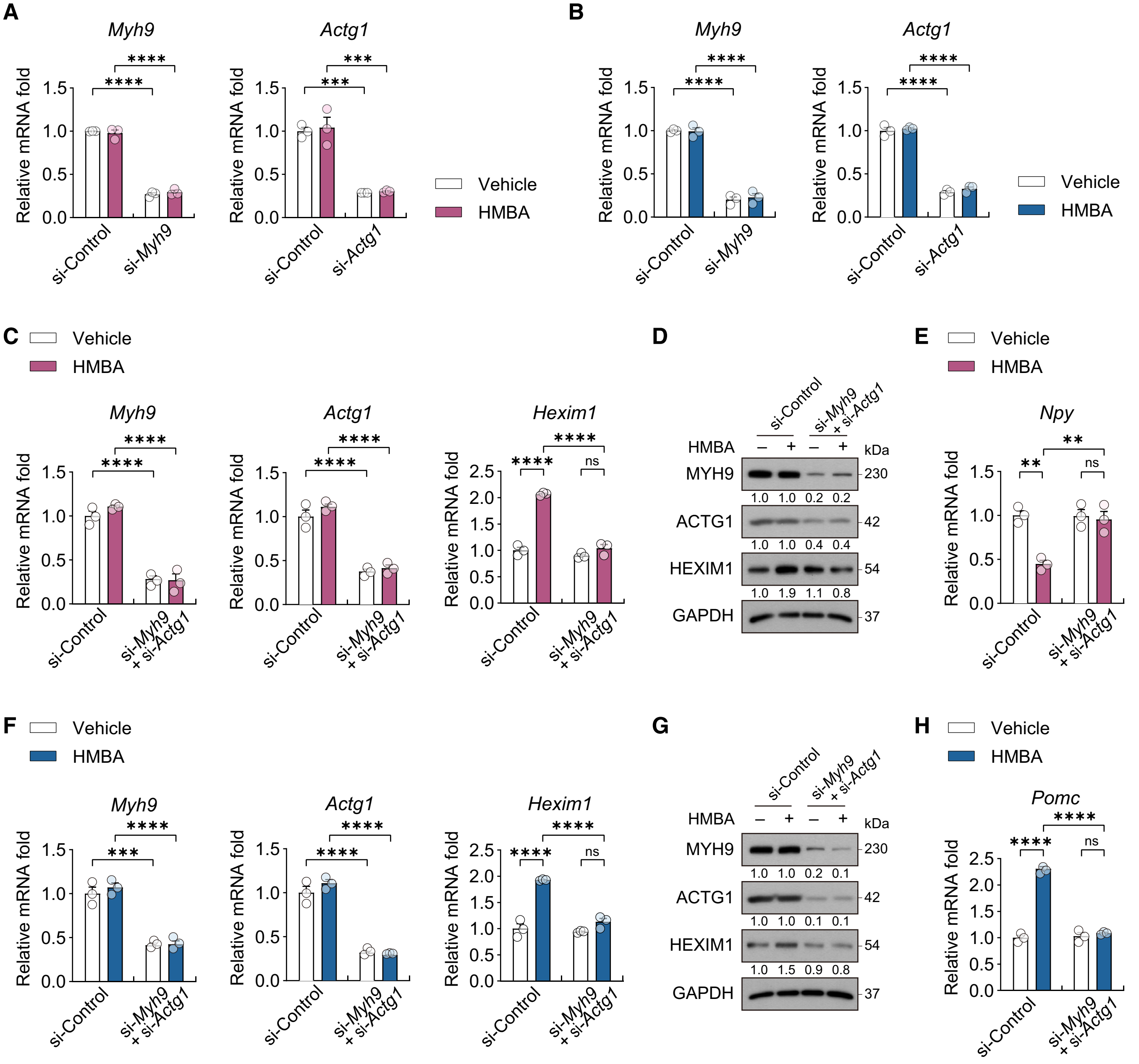

Figure EV3.

 © 2023 The Authors

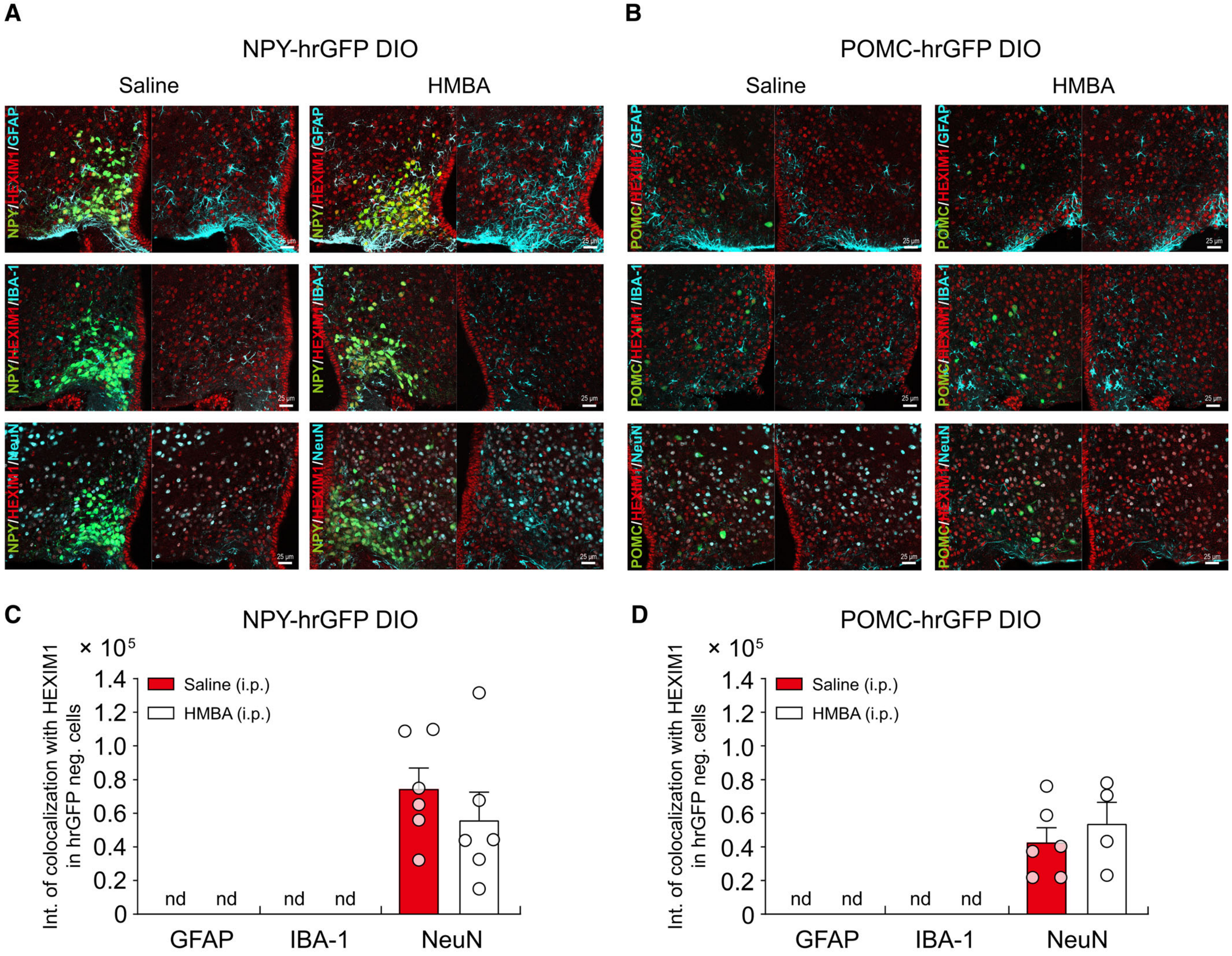

Figure EV4. HMBA specifically induces HEXIM1 expression in NPY and POMC neurons rather than other cells in the ARC.

A, B Immunohistochemistry analysis for the fluorescence intensity of GFAP (astrocyte), IBA-1 (microglia), NeuN (mature neuron), and HEXIM1 in the ARC of (A) NPY-hrGFP DIO mice and (B) POMC-hrGFP DIO mice after HMBA treatment. Scale bars, 25 μm.

C, D The fluorescence intensity of HEXIM1 (Cy3) which co-localized with hrGFP-negative cells in the ARC of (C) NPY-hrGFP DIO mice and (D) POMC-hrGFP DIO mice after HMBA treatment. $n = 6$ per group (saline- or HMBA-injected NPY-hrGFP DIO mice, and saline-injected POMC-hrGFP DIO mice): three brain slices were obtained from each of the two mice per group; $n = 4$: two brain slices were obtained from each of the two HMBA-injected POMC-hrGFP DIO mice.

Data information: NPY-hrGFP and POMC-hrGFP DIO mice fed an HFD for 4 weeks were injected with HMBA (i.p., 1,000 mg/kg), and then brains were harvested 4 h after injection. Statistical significance was determined by a two-tailed unpaired Student's *t*-test. nd, not detected. Data are mean ± SEM. The exact *P*-values are reported in Appendix Table S6.

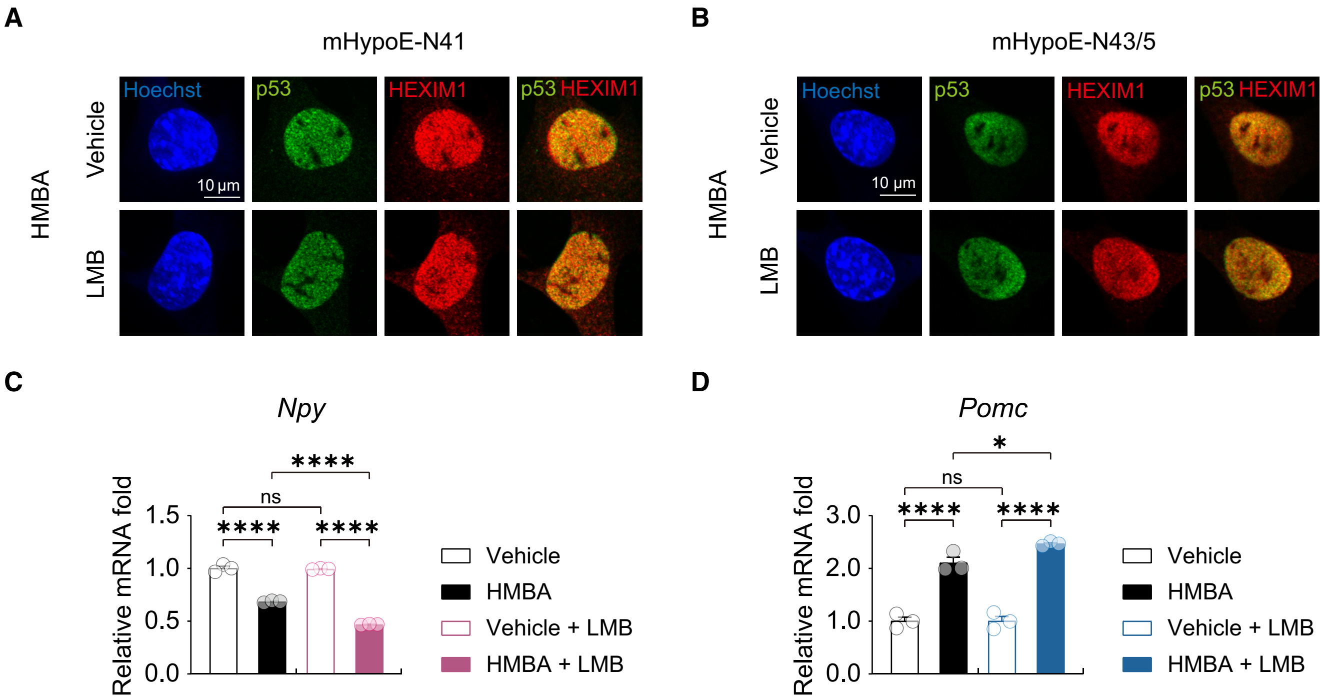

Figure EV5. Inhibition of nuclear export of both HEXIM1 and p53 by leptomycin B effectively modulates neuropeptide expression.

A, B Immunofluorescence analysis of HEXIM1 and p53 after HMBA and leptomycin B (LMB) treatment in (A) mHypoE-N41 and (B) mHypoE-N43/5.

C Relative mRNA levels of *Npy* in mHypoE-N41. $n = 3$ per group.

D Relative mRNA levels of *Pomc* in mHypoE-N43/5. $n = 3$ per group.

Data information: Cells were pre-treated with 0.1 mM HMBA for 1 h and then treated with 50 ng/ml LMB. Scale bars, 10 μm. The datasets in qPCR experiments were comprised of three biological replicates and each biological replicate was an average of three technical replicates. Statistical significance was determined by two-way ANOVA followed by a *post hoc* Tukey test. $^*P < 0.05$, $^{**}P < 0.01$, $^{***}P < 0.001$, $^{****}P < 0.0001$; ns or unless otherwise stated, no significance. Data are mean ± SEM. The exact *P*-values are reported in Appendix Table S6.

 © 2023 The Authors

Appendix

HMBA ameliorates obesity by MYH9 and ACTG1-dependent regulation of hypothalamic neuropeptides

Appendix Figures 3

Appendix Figure S1. 3

Appendix Figure S2. 5

Appendix Figure S3. 6

Appendix Figure S4. 7

Appendix Figure S5. 8

Appendix Figure S6. 10

Appendix Figure S7. 12

Appendix Figure S8. 14

Appendix Figure S9. 15

Appendix Figure S10. 16

Appendix Figure S11. 17

Appendix Figure S12. 18

Appendix Tables **20**

Appendix Table S1. 20

Appendix Table S2. 21

Appendix Table S3. 22

Appendix Table S4. 23

Appendix Table S5. 24

Appendix Table S6. 25

References **29**

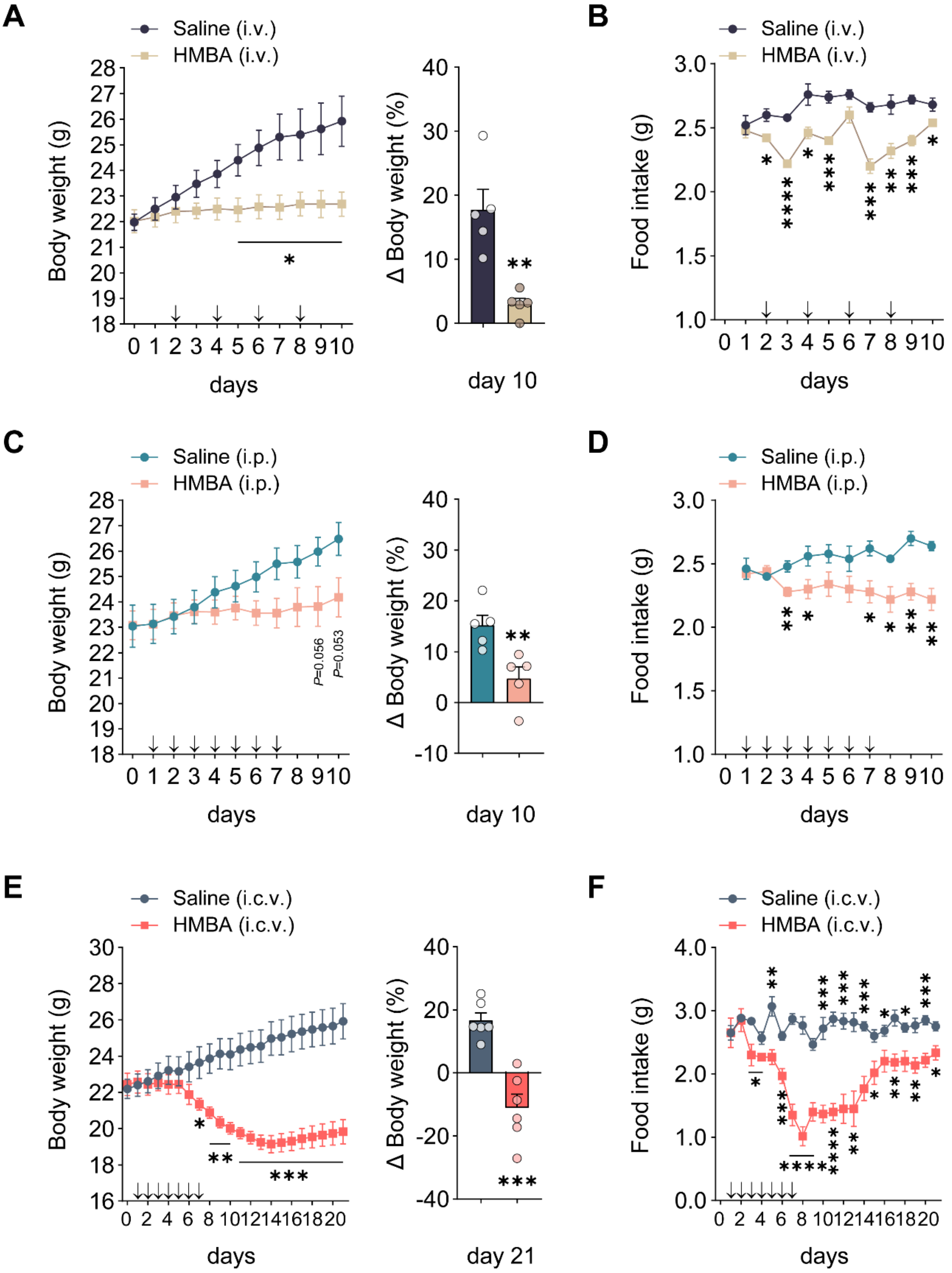

A
Saline (i.v.)
HMBA (i.v.)
Body weight (g)
days
Δ Body weight (%)
day 10
B
Food intake (g)
C
Saline (i.p.)
HMBA (i.p.)
P=0.056
P=0.053
D
E
Saline (i.c.v.)
HMBA (i.c.v.)
day 21
F

Appendix Figure S1. Peripheral and central administration of HMBA has anti-obesity effects in female DIO mice

A–B. Changes in (A) daily body weight (grams) (left panel) and body weight (percentages) on day 10 relative to day 0 (right panel), and (B) food intake in female DIO mice injected i.v. with HMBA. Mice were injected with 500 mg/kg of HMBA on day 1, followed by three injections of 1,000 mg/kg bi-daily. n = 5 per group. Arrows represent the days of each injection.

C–D. Changes in (C) daily body weight (grams) (left panel) and body weight (percentages) on day 10 relative to day 0 (right panel), and (D) food intake in female DIO mice injected i.p. with HMBA. Mice were injected with 1,000 mg/kg of HMBA daily for 7 days. n = 5 per group. Arrows represent the days of each injection.

E–F. Changes in (E) daily body weight (grams) (left panel) and body weight (percentages) on day 21 relative to day 0 (right panel), and (F) food intake in female DIO mice injected i.c.v. with HMBA. Mice were injected with 400 nmoles of HMBA (2 μl per injection) daily for 7 days. n = 6 per group. Arrows represent the days of each injection.

Data information: Data represent different numbers (n) of biological replicates. Statistical significance was determined by a two-tailed unpaired Student's t-test. *P<0.05, **P<0.01, ***P<0.001, ****P<0.001 vs. Saline. Data are mean ± SEM.

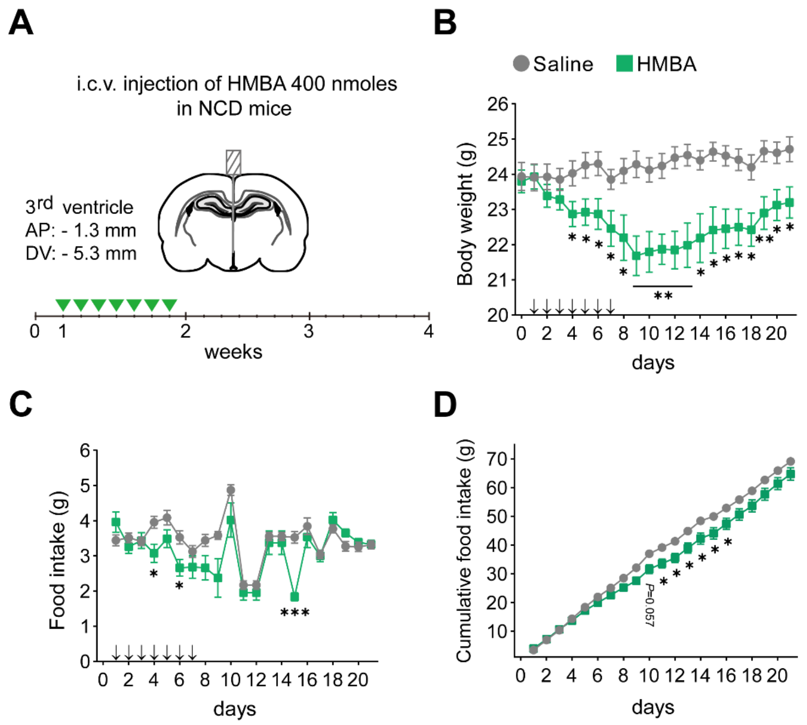

Appendix Figure S2. Central administration of HMBA has anti-obesity effect in mice NCD mice

A. Schematic of the experimental design. HMBA (400 nmoles per injection) was injected daily for 7 days. AP, anterior/posterior; DV, dorsal/ventral. Triangles represent the days of each injection.

B–D. Changes in **(B)** body weight, **(C)** food intake, and **(D)** cumulative food intake. n = 7, Saline; n = 8, HMBA. Arrows represent the days of each injection.

Data information: Data represent different numbers (n) of biological replicates. Statistical significance was determined by a two-tailed unpaired Student's t-test. *P<0.05, **P<0.01, ***P<0.001 vs. Saline. Data are mean ± SEM.

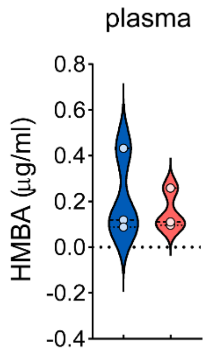

Appendix Figure S3. Central administration of HMBA does not affect plasma HMBA concentration

HMBA (400 nmoles per injection) was injected intracerebroventricularly daily for 7 days. Blue, saline injection; red, HMBA injection. $n = 3$ per group.

Data information: Data represent three biological replicates. Statistical significance was determined by a two-tailed unpaired Student's *t*-test. There was no significant difference between the groups. Data are mean ± SEM.

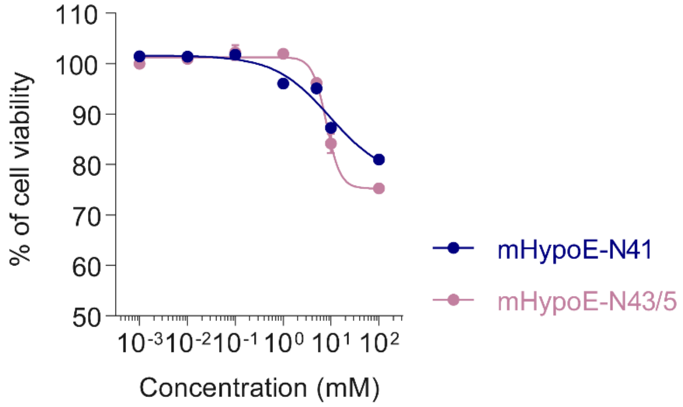

Appendix Figure S4. Cell viability in hypothalamic cell lines at different HMBA concentrations

Cells were treated with various doses (10^{-3}, 10^{-2}, 10^{-1}, 10^{0}, 10^{1}, 10^{2} mM) of HMBA for 24 h. Cell viability was measured using a CellTiter-Blue cell viability assay kit. Data are mean ± SEM. $n = 5$ per group; Data represent five technical replicates.

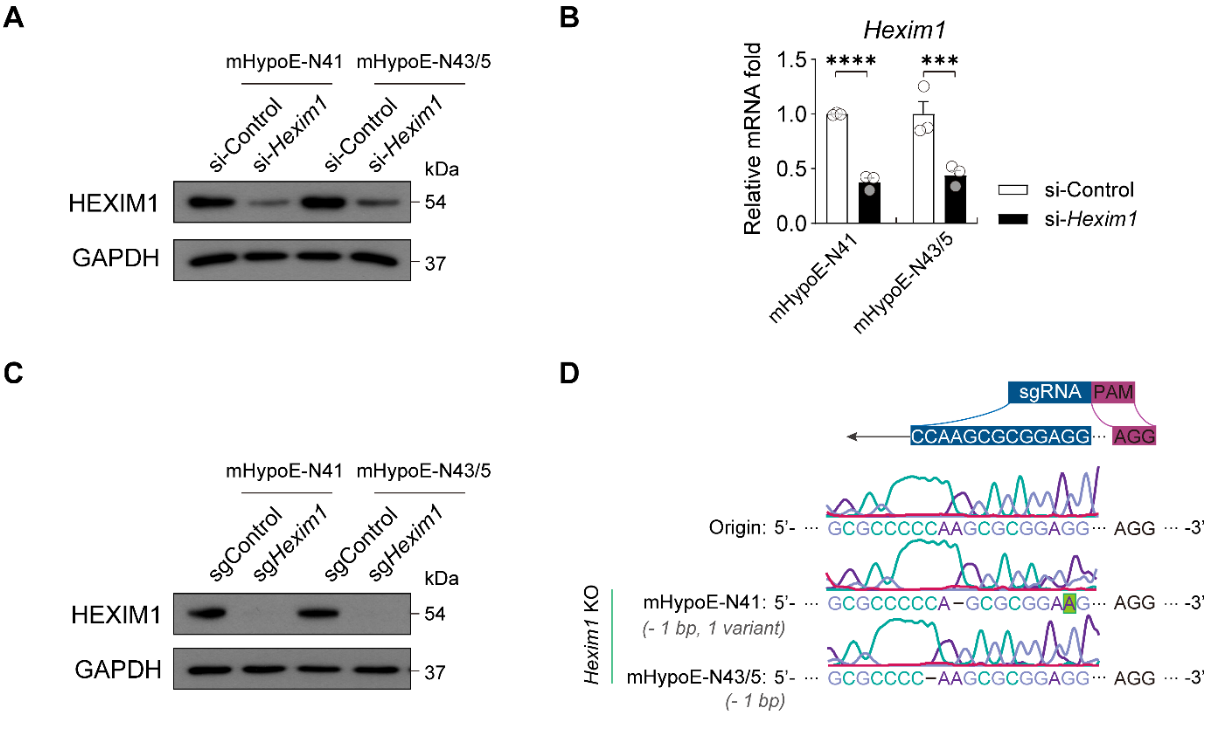

Appendix Figure S5. The efficiency of *Hexim1* knockdown and knockout

A. Western blot analysis of *Hexim1* knockdown (si-*Hexim1*) in mHypoE-N41 and mHypoE-N43/5.

B. Relative *Hexim1* mRNA levels after *Hexim1* knockdown in mHypoE-N41 and mHypoE-N43/5. $n = 3$ per group.

C. Western blot analysis of *Hexim1* knockout (sg*Hexim1*) in mHypoE-N41 and mHypoE-N43/5.

D. Confirmation of *Hexim1* knockout in mHypoE-N41 and mHypoE-N43/5 by sequencing. One base pair was deleted and substituted in mHypoE-N41, and one base pair was deleted in mHypoE-N43/5.

Data information: qPCR data represent three technical replicates. Statistical significance was

determined by a two-tailed unpaired Student's t-test. ***P<0.001, ****P<0.0001. Data are mean ± SEM.

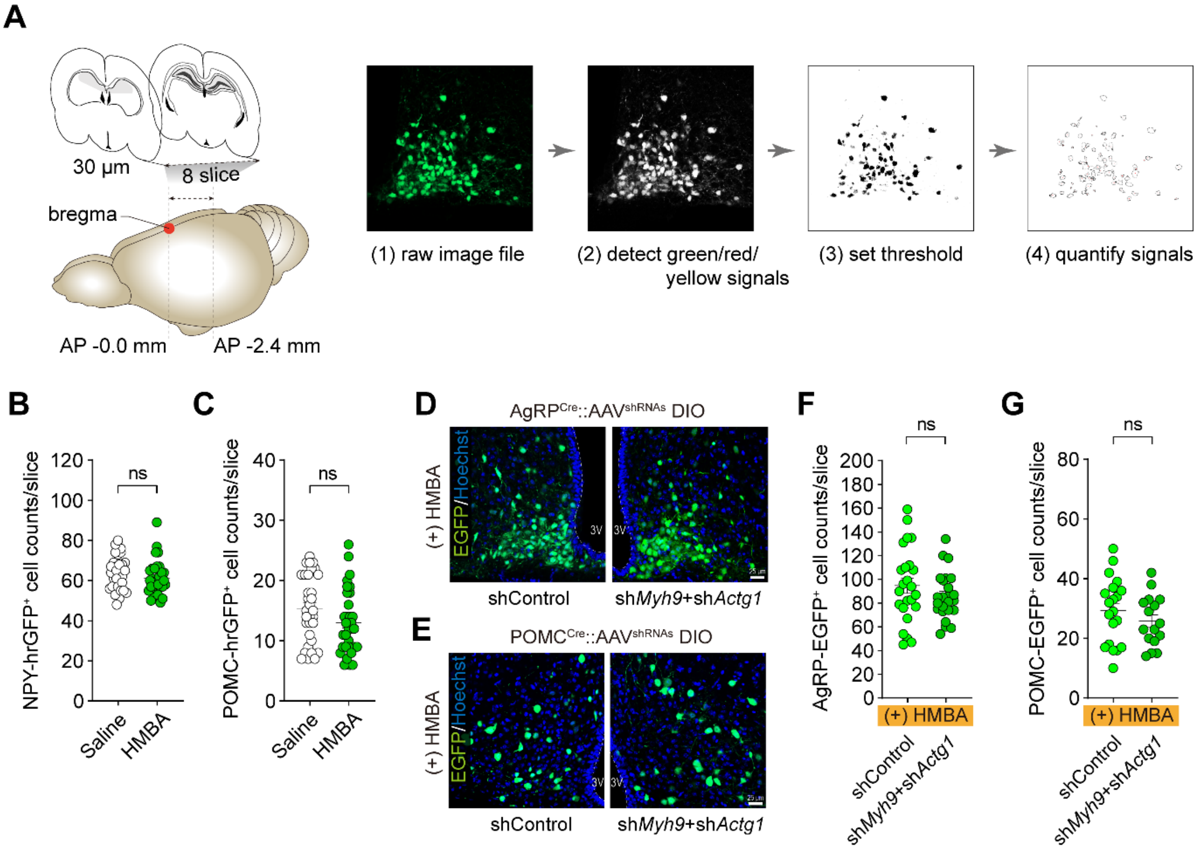

Appendix Figure S6. The number of hrGFP or EGFP-positive cells remains unchanged

A. Schematic diagram of experimental design for immunohistochemistry analysis. NPY-hrGFP and POMC-hrGFP DIO mice fed an HFD for 4 weeks were injected with HMBA (i.p., 1,000 mg/kg), and then brains were harvested 4 h after injection. AgRP^{Cre} and POMC^{Cre} DIO mice fed an HFD for 4 weeks were injected with AAV-FLEXon-shRNAs (shControl or sh*Myh9*+sh*Actg1*)-EGFP viruses into the ARC. After recovery, mice were injected with HMBA (i.p., 1,000 mg/kg), and then brains were harvested 4 h after injection. Brain slices were prepared posteriorly from the bregma, with a maximum of 8 slices per mouse and a thickness

of 30 μm. The number and intensity of positive signals (green, red, yellow) were analyzed via Fiji of ImageJ 2 software using the following sequence: (1) load raw image file, (2) signal detection by colors, (3) threshold setting of detectable cell size, (4) quantification of cell number and intensity.

B–C. The number of hrGFP-positive ($hrGFP^{+}$) cells per slice in (B) NPY-hrGFP DIO mice and (C) POMC-hrGFP DIO mice. *n* = 32 per group (eight brain slices were obtained from each of the four mice per group).

D–E. Immunohistochemistry analysis for the functional efficiency of AAV in the ARC of (**D**) $AgRP^{Cre}$::$AAV^{(shControl\ or\ sh\mathit{Myh9}+sh\mathit{Actg1})}$ DIO mice and **(E)** $POMC^{Cre}$::$AAV^{(shControl\ or\ sh\mathit{Myh9}+sh\mathit{Actg1})}$ DIO mice after HMBA treatment. Scale bars, 25 μm.

F–G. The number of EGFP-positive ($EGFP^{+}$) cells per slice in **(F)** $AgRP^{Cre}$::$AAV^{(shControl\ or\ sh\mathit{Myh9}+sh\mathit{Actg1})}$ DIO mice and **(G)** $POMC^{Cre}$::$AAV^{(shControl\ or\ sh\mathit{Myh9}+sh\mathit{Actg1})}$ DIO mice. *n* = 24 per group (eight brain slices were obtained from each of the three mice per group).

Data information: Statistical significance was determined by a two-tailed unpaired Student's t-test. ns, no significance. Data are mean ± SEM.

A

Plasmid DNA 1 : pAAV[FLEXon]-CMV>LL:rev(EGFP:Control[shRNA#1]:Control[shRNA#2]):rev(LL):WPRE
Vector size : 6055 bp
Viral genome size: 3436 bp
Virus titer (QC) : 4.20 x 10^{13} GC/mL

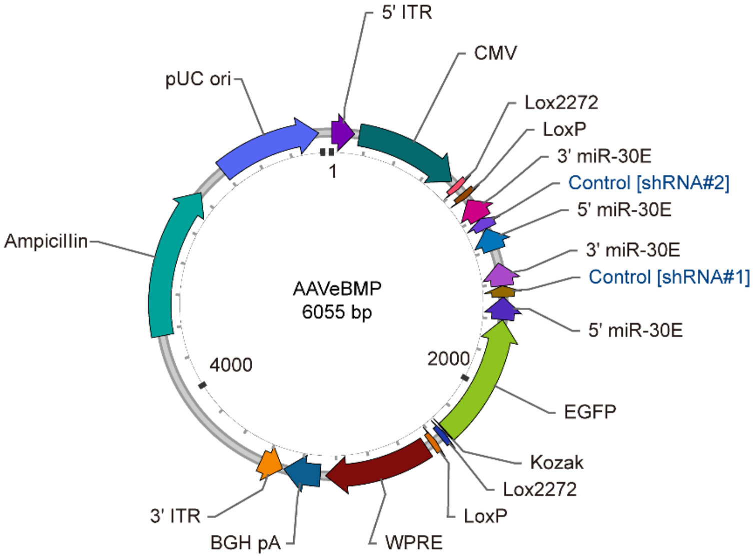

B

Plasmid DNA 2 : pAAV[FLEXon]-CMV>LL:rev(EGFP:m*Myh9*[shRNA#1]:m*Actg1*[shRNA#2]):rev(LL):WPRE
Vector size : 6055 bp
Viral genome size: 3436 bp
Virus titer (QC) : 1.06 x 10^{13} GC/mL

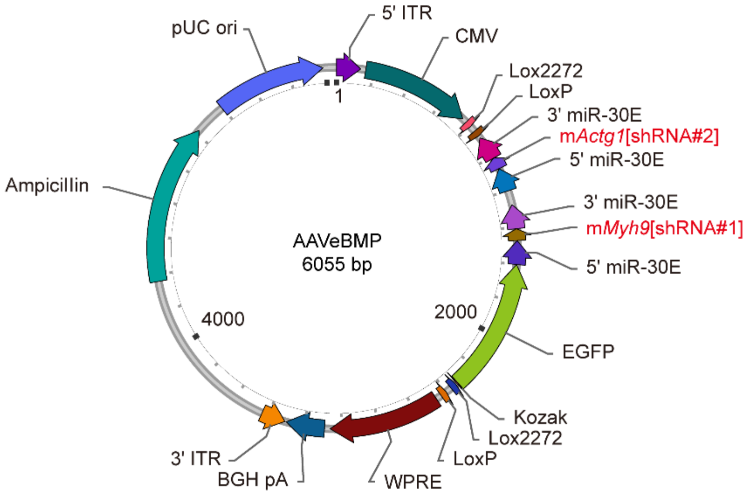

Appendix Figure S7. Schematic representation of plasmid DNA used for producing Cre-dependent adeno-associated virus (AAV) viruses

The AAV-PHP.eB serotype was used to package EGFP-expressing AAV. A miRNA (miR)-based shRNA system was inserted into the pAAV FLEX Cre-on vector to allow for multiple shRNAs to be expressed on one vector. Sites of shRNAs inserted were *Myh9* (shRNA #1), *Actg1* (shRNA #2), and Control (shRNA #1, 2). The titrations of ultra-purified AAV were validated using quantitative PCR.

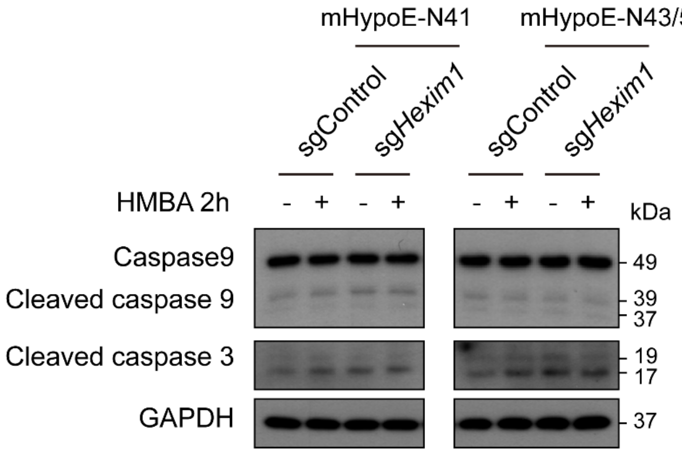

Appendix Figure S8. HMBA or *Hexim1* deletion does not cause apoptosis

Western blot analysis of caspase 9, cleaved caspase 9, and cleaved caspase 3 after HMBA treatment in sgControl or sg*Hexim1* mHypoE-N41 and mHypoE-N43/5 cells. Cells were treated or not with 0.1 mM HMBA for 2 h. The experiment was repeated twice independently with similar results.

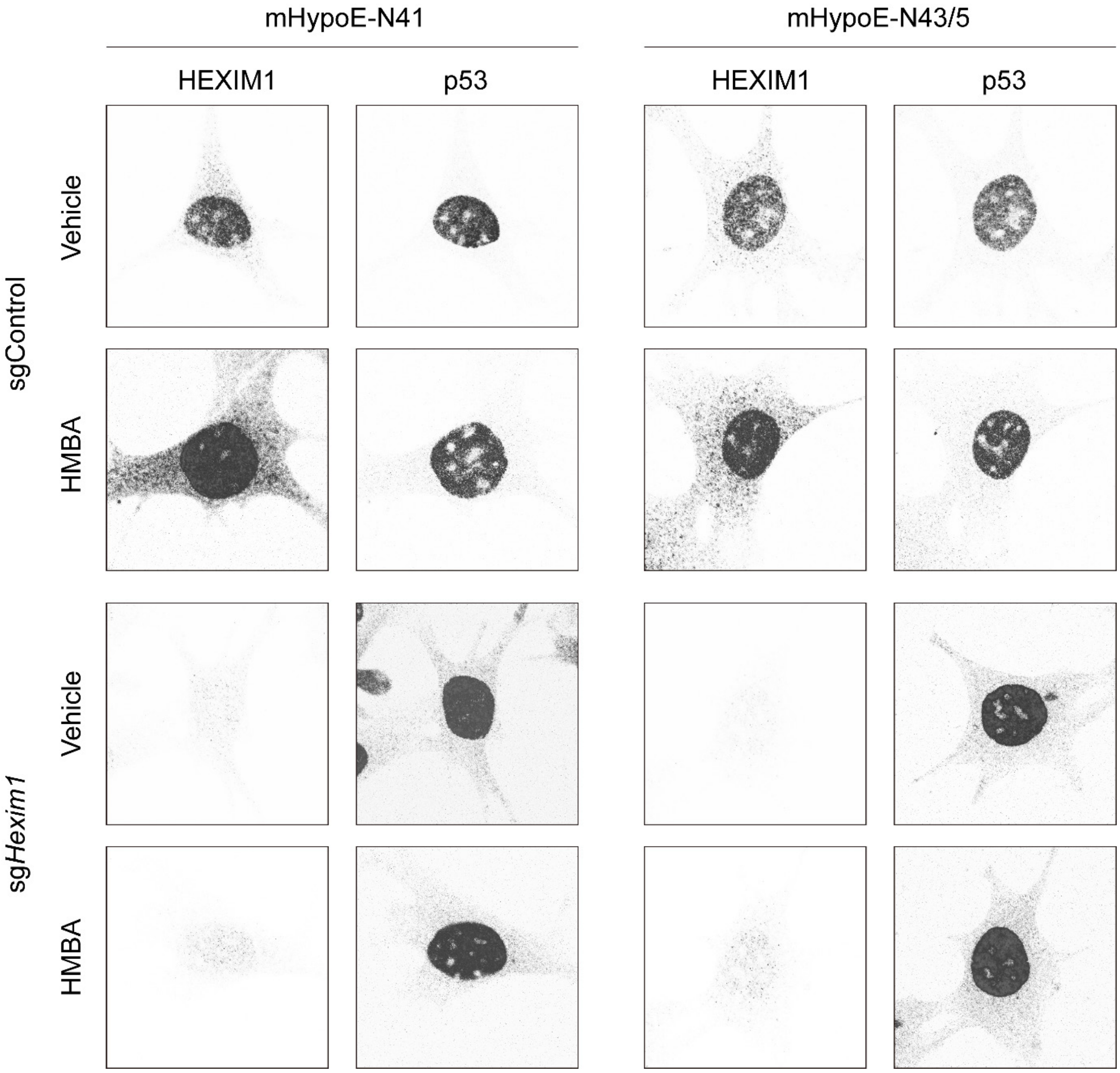

Appendix Figure S9. Localization of HEXIM1 and p53 after HMBA treatment

Immunofluorescence analysis of sgControl and sg*Hexim1* mHypoE-N41 and mHypoE-N43/5 cells after HMBA treatment. Cells were treated with 0.1 mM HMBA for 2 h. Images show the same cells as in Fig 7A and B; black-and-white inverted images are shown to better visualize the localization of HEXIM1 and p53.

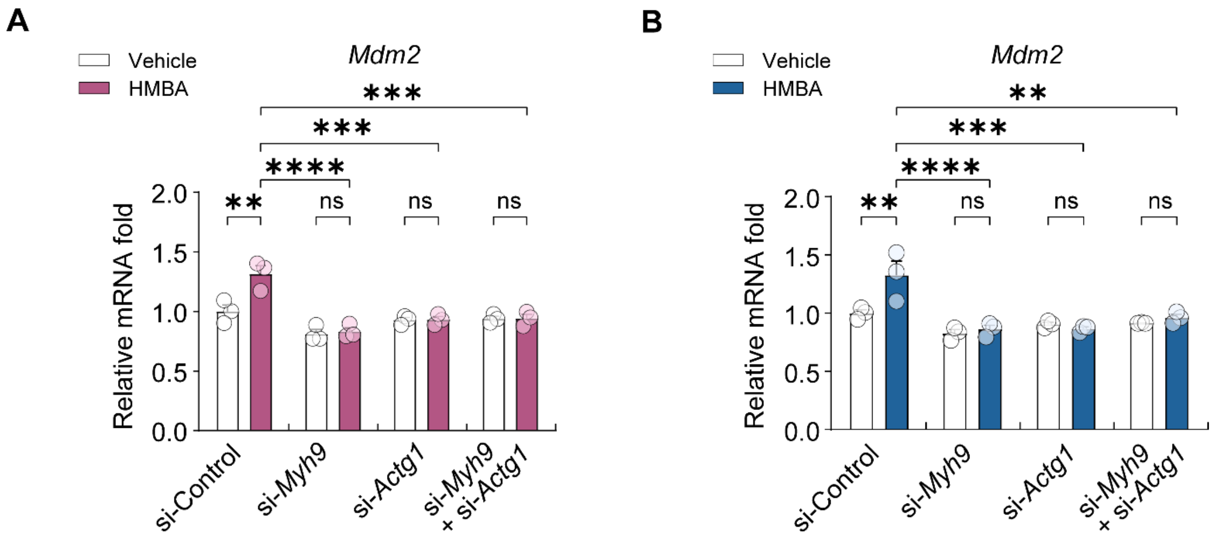

Appendix Figure S10. HMBA induces *Mdm2* expression in the presence of MYH9 and ACTG1

A, B. Relative mRNA levels of *Mdm2* in (**A**) mHypoE-N41 and (**B**) mHypoE-N43/5. $n = 3$ per group.

Data information: After the knockdown of *Myh9*, *Actg1*, or both, cells were treated with 0.1 mM HMBA for 2 h. The datasets in qPCR experiments were comprised of three biological replicates and each biological replicate was an average of three technical replicates. Statistical significance was determined by two-way ANOVA followed by a post hoc Bonferroni test. ***P*<0.01, ****P*<0.001, *****P*<0.0001; ns or unless otherwise stated, no significance. Data are mean ± SEM.

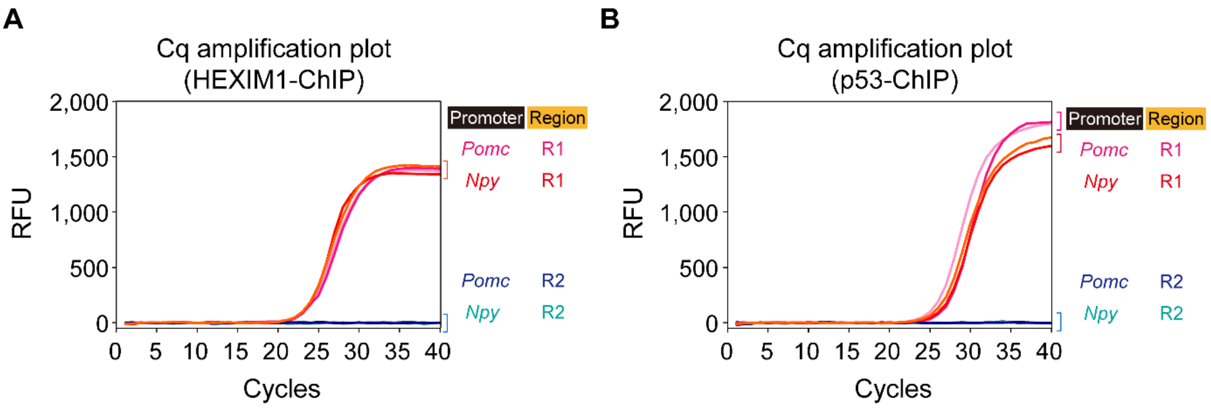

Appendix Figure S11. HEXIM1 and p53 bind to regions 1 but not regions 2 of the *Npy* and *Pomc* promoters

A, B. The quantification cycle (Cq) amplification plots from ChIP-qPCR analysis of the binding of **(A)** HEXIM1 or **(B)** p53 to the *Npy* and *Pomc* promoters. R1, promoter region 1; R2, promoter region 2. Relative fluorescence units (RFU) are used to indicate fluorescence intensity. Details of promoter regions are shown in Fig 7D and E and the primers used for each region are listed in Appendix Table 4.

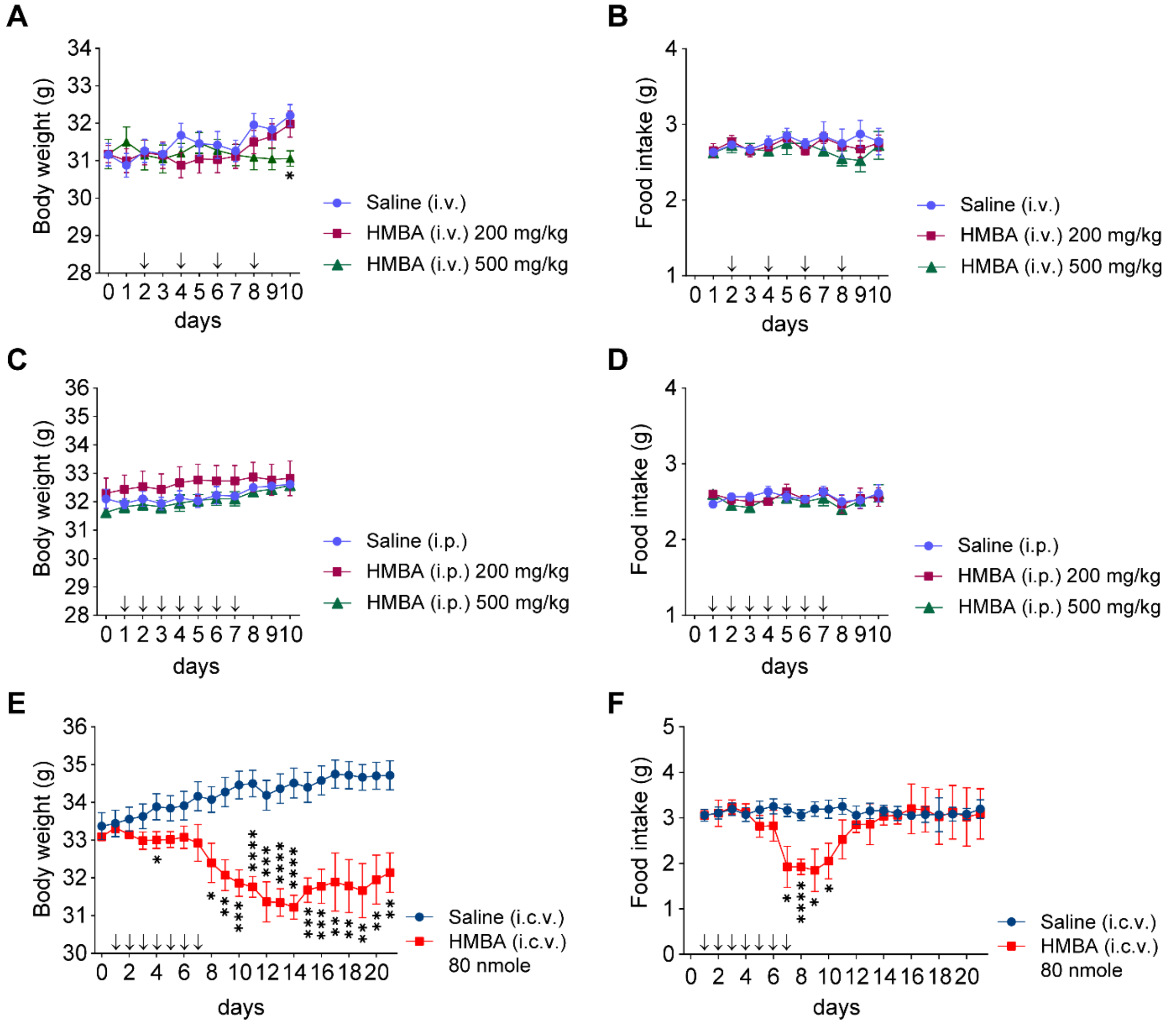

Appendix Figure S12. The effects of low-dose HMBA in male DIO mice

A–B. Changes in (A) daily body weight and (B) food intake in male DIO mice injected i.v. with HMBA. Mice were injected with 200 or 500 mg/kg of HMBA bi-daily. *n* = 5 per group. Arrows represent the days of each injection.

C–D. Changes in (C) daily body weight and (D) food intake in male DIO mice injected i.p.

with HMBA (n = 5). Mice were injected with 200 or 500 mg/kg of HMBA daily for 7 days. *n* = 3, saline and HMBA (i.p.) 200 mg/kg group; *n* = 4, HMBA (i.p.) 500 mg/kg group. Arrows represent the days of each injection.

E–F. Changes in (E) daily body weight and (F) food intake in male DIO mice injected i.c.v. with HMBA. Mice were injected with 80 nmoles of HMBA (2 µl per injection) daily for 7 days. *n* = 8 per group. Arrows represent the days of each injection.

Data information: Data represent different numbers (*n*) of biological replicates. Statistical significance was determined by a two-tailed unpaired Student's *t*-test. **P*<0.05, ***P*<0.01, ****P*<0.001, *****P*<0.001 vs. Saline. Data are mean ± SEM.

Appendix Table S1. Top 10 small molecules in the Connectivity Map database

Rank	Name	References[1]	Function
1	Oleoylethanolamide	Fu *et al*, 2003	Anorexigenic effect
2	Lithocholic acid	Yousef *et al*, 1988	Induces cholestasis
3	Mestanolone	Harris, 1961	Anabolic steroid
4	Hydroxycholesterol	Asghari *et al*, 2019	Induces obesity
5	Otenzepad	None	None (anti-muscarinic)
6	Valaciclovir	None	None (anti-viral)
7	Artemether	Fu *et al*, 2020	Improves glycolipid metabolism
8	Hexamethylene bisacetamide	None	None (anti-cancer)
9	Guggulsterone	Yang *et al*, 2012	Hypolipidemic effect
10	Geranylgeraniol	Campia *et al*, 2009	Reduces cholesterol synthesis

[1] References and function refer to metabolism-related content only

Appendix Table S2. Precursor-to-fragment ion transitions in LC-MS/MS analysis

**Analyte**[2]	**Precursor ion (*m/z*)**	**Fragment ion (*m/z*)**	**Collision energy (ev)**
HMBA	201.1	159.2	10
NADAH	158.9	99.9	8
DAH	132.0	114.0	10
AmHA	117.1	100.1	20
HMBA–Biotin	385.2	326.1	16

[2] Metabolites were analyzed using positive electrospray ionization mode

Appendix Table S3. Sequences of primers used for qRT-PCR

Gene	Forward primer (5′ → 3′)	Reverse primer (5′ → 3′)
Agrp	CTGCAGACCGAGCAGAAGA	TGCGACTACAGAGGTTCGTG
Actg1	TACCCTATTGAGCACGGCAT	CGCAGCTCGTTGTAGAAGGT
Cart	CGAGAAGAAGTACGGCCAAGTCC	GGAATATGGGAACCGAAGGTGG
Gapdh	ATCACTGCCACCCAGAAGAC	ACACTTGGGGGTAGGAACA
Hexim1	TACAGGTGCTGCTGTTGTCC	CTCCAGGGAGCTCAATTCTG
Hexim2	AGGGTGAATGTCTCGTGCAG	TGCCCACCTACAGACACAAC
Npy	CAGAAAACGCCCCCAGAA	AAAAGTCGGGAGAACAAGTTTCATT
Mdm2	CCGAGTTTCTCTGTGAAGGAGC	GTCTGCTCTCACTCAGCGATGT
Myh9	GGCCCTGCTAGATGAGGAGT	CTTGGGCTTCTGGAACTTGG
Trp53 (*p53*)	CTCCGAAGACTGGATGACTGC	CAACAGATCGTCCATGCAGTG
Pomc	GAACAGCCCCTGACTGAAAA	ACGTTGGGGTACACCTTCAC

Appendix Table S4. Sequences of primers used for ChIP-qPCR

	Gene	Forward primer (5′ → 3′)	Reverse primer (5′ → 3′)
Promoter region 1	*Npy* −605/+3bp	GCGAGGAGCACAGTGGCGTT	AGCAGGGACCGCTTAGATTGCCT
	Pomc −414/−259bp	AATCTGCGACATAACAAATCCCC	AGAACTGGACAGAGGCTTAGCGT
Promoter region 2	*Npy* −118/+22bp	CCCAAGTACAGTGTCTGGTCCCT	TGGGCGCCTCTGCGAGGAAT
	Pomc −162/+82bp	CTTTCCAGGCAGATGTGCCTTG	CAGCTCTCTGGGGACAAAGATGTT

Appendix Table S5. List of antibodies

Antibody	Manufacturer	Catalog Number	Dilution or amount
ACTG1	Santa Cruz Biotechnology	SC-65638	1:1000 for WB<br>1:400 for IHC
Caspase 9/Cleaved Caspase 9	Cell Signaling Technology (CST)	9504	1:1000
Cleaved Caspase 3	CST	9661	1:1000
H3K9Ac	Invitrogen	MA5-33384	2 μg for ChIP
H3K4me3	Abcam	ab8580	2 μg for ChIP
H3K27me3	Abcam	ab6002	2 μg for ChIP
HEXIM1	Bethyl Laboratories	A303-112A	1:1000 for WB<br>1:150 for IF<br>1:400 for IHC<br>2 μg for IP/ChIP
IBA-1	Abcam	ab5076	1:400 for IHC
MDM2	CST	51541	1:1000 for WB
MYH9	CST	3403	1:1000 for WB<br>1:500 for IHC
NeuN	Millipore	MAB377	1:300 for IHC
p53	Santa Cruz Biotechnology	SC-99	1:1000 for WB
	Santa Cruz Biotechnology	SC-126	1:200 for IF
GAPDH	CST	2118	1:10000
GFAP	Dako	Z0334	1:600 for IHC
Goat anti-Rabbit IgG, HRP	Invitrogen	31460	1:10000
Mouse anti-Rabbit IgG, HRP	Abcam	99697	1:5000
Goat anti-Mouse IgG, HRP	CST	7076	1:10000
Donkey anti-mouse IgG, Alexa Fluor 488	Jackson ImmunoResearch Laboratories	715-545-150	1:500
Donkey anti-mouse IgG, Alexa Fluor 647	Jackson ImmunoResearch Laboratories	715-605-151	1:500
Donkey anti-rabbit IgG, Cy3	Jackson ImmunoResearch Laboratories	711-165-152	1:500
Donkey anti-goat IgG, Cy3	Jackson ImmunoResearch Laboratories	705-165-147	1:500

Appendix Table S6. The exact *P* values

Figure	Panel	Statistics analysis	*P* value	Note
1	C	Two-tailed t test	0.0470	Body weight (g), day 5
			0.0225	Body weight (g), day 7
			0.0150	Body weight (g), day 8
			0.0022	Body weight (g), day 9
			0.0018	Body weight (g), day 10
			<0.0001	Body weight (%) on day 10
	D	Two-tailed t test	0.0191	Food intake (g), day 3
			0.0054	Food intake (g), day 4
			<0.0001	Food intake (g), day 8
			0.0107	Food intake (g), day 9
	E	Two-tailed t test	0.0384	Body weight (g), day 7
			0.0221	Body weight (g), day 8
			0.0189	Body weight (g), day 9
			0.0199	Body weight (g), day 10
			0.0074	Body weight (%) on day 10
	F	Two-tailed t test	0.0049	Food intake (g), day 8
			0.0035	Food intake (g), day 9
			0.0203	Food intake (g), day 10
	G	One-way ANOVA	<0.0001	Saline (i.v.) vs HMBA (i.v.) in plasma
			0.0036	Saline (i.p.) vs HMBA (i.p.) in plasma
			0.0369	HMBA (i.v.) vs HMBA (I.p.) in plasma
			<0.0001	Saline (i.v.) vs HMBA (i.v.) in hypothalamus
			<0.0001	Saline (i.p.) vs HMBA (i.p.) in hypothalamus
			0.0001	HMBA (i.v.) vs HMBA (I.p.) in hypothalamus
	H	Two-tailed t test	0.0009	*Npy*
			0.0007	*Agrp*
			0.0235	*Pomc*
			0.0011	*Cart*
			0.0252	*Hexim1*
	I	Two-tailed t test	0.0001	*Npy*
			<0.0001	*Agrp*
			0.0326	*Pomc*
			0.0267	*Cart*
			<0.0001	*Hexim1*
	J	Two-tailed t test	0.0307	fat mass
	K	Two-tailed t test	0.0010	
	L	Two-tailed t test	<0.0001	
	M	ANCOVA	0.0139	Left panel
		Two-tailed t test	0.0161	Right panel
	N	Two-tailed t test	0.0483, 0.0485	Light, total
	O		0.0017, 0.0093, 0.0039	Dark, light, total
	P		0.0348, 0.0365	Dark, total
	Q		0.0027, 0.0128, 0.0057	Dark, light, total
2	B	Two-tailed t test	0.0141	Body weight (g), day 7
			0.0047	Body weight (g), day 8
			0.0002	Body weight (g), day 9
			<0.0001	Body weight (g), day 10 ~ 21
			<0.0001	Body weight (%) on day 21
	C	Two-tailed t test	0.0132	Food intake (g), day 2
			0.0123	Food intake (g), day 3
			0.0006	Food intake (g), day 6
			<0.0001	Food intake (g), day 8 ~ 17
			0.0053	Food intake (g), day 19
			0.0022	Food intake (g), day 20
			<0.0001	Food intake (g), day 21
	D	Two-tailed t test	0.0275	*Npy*
			0.0449	*Agrp*
			0.0004	*Pomc*
			0.0121	*Cart*
			0.0001	*Hexim1*
	E	Two-tailed t test	0.0002	fat mass
			0.0005	lean mass

	F	Two-tailed t test	0.0311	
	G	Two-tailed t test	0.0081	
	H	ANCOVA	0.0011	Left panel
		Two-tailed t test	<0.0001	Right panel
	J	Two-tailed t test	0.0153, 0.0025, 0.0205	Dark, light, total
	K		0.0087, 0.0043, 0.0178	Dark, light, total
	L		0.0001	All
	O	Two-way ANOVA	<0.0001, 0.0091	15 ~ 90 min, 120 min
		Two-tailed t test	0.0041	AUC
	P	Two-way ANOVA	0.0364, <0.0001, 0.0041, 0.0022, <0.0001	0, 15, 30, 60, 90 ~ 10 min
		Two-tailed t test	0.0110	AUC
	Q	Two-tailed t test	0.0052	
	R	Two-tailed t test	0.0125, 0.0097	Total, in SFA, in PUFA
3	F	Two-way ANOVA	<0.0001	2 h
			0.0005	4 h
			0.0003	8 h
			0.0004	24 h
	G	Two-way ANOVA	0.0018	0.5 h
			0.0065	1 h
			<0.0001	2 h
			<0.0001	4 h
			0.0049	8 h
			0.0003	24 h
	H	Two-way ANOVA	<0.0001	All
	I	Two-way ANOVA	0.0388, <0.0001	10^{-2}, 10^{-1} in 1 h
			0.0003, <0.0001	10^{-2}, 10^{-1} in 2 h
	K	Two-way ANOVA	0.0123	0.5 h
			<0.0001	1 h
			<0.0001	2 h
			<0.0001	4 h
			<0.0001	8 h
			<0.0001	24 h
	L	Two-way ANOVA	<0.0001	0.5 h
			<0.0001	1 h
			<0.0001	2 h
			0.0002	4 h
	M	Two-way ANOVA	0.0148	Vehicle vs HMBA in si-Control
			>0.9999	Vehicle vs HMBA in si-*Hexim1*
			0.0038	Vehicle (si-Control vs si-*Hexim1*)
			>0.9999	HMBA (si-Control vs si-*Hexim1*)
	N		<0.0001	Vehicle vs HMBA in si-Control
			0.9996	Vehicle vs HMBA in si-*Hexim1*
			0.1300	Vehicle (si-Control vs si-*Hexim1*)
			<0.0001	HMBA (si-Control vs si-*Hexim1*)
	O		0.0001	Vehicle vs HMBA in sgControl
			>0.9999	Vehicle vs HMBA in sg*Hexim1*
			<0.0001	Vehicle (sgControl vs sg*Hexim1*)
			<0.0001	HMBA (sgControl vs sg*Hexim1*)
	P		<0.0001	Vehicle vs HMBA in sgControl
			0.6734	Vehicle vs HMBA in sg*Hexim1*
			0.0361	Vehicle (sgControl vs sg*Hexim1*)
			<0.0001	HMBA (sgControl vs sg*Hexim1*)
4	I	Two-way ANOVA	<0.0001	Vehicle vs HMBA in si-Control
			0.0566	Vehicle vs HMBA in si-*Myh9*
			0.0009	HMBA (si-Control vs si-*Myh9*)
	J		0.0013	Vehicle vs HMBA in si-Control
			0.6083	Vehicle vs HMBA in si-*Myh9*
			0.0034	HMBA (si-Control vs si-*Myh9*)
	K		<0.0001	Vehicle vs HMBA in si-Control
			0.1362	Vehicle vs HMBA in si-*Actg1*
			0.0002	HMBA (si-Control vs si-*Actg1*)
	L		0.0005	Vehicle vs HMBA in si-Control
			0.9044	Vehicle vs HMBA in si-*Actg1*
			0.0011	HMBA (si-Control vs si-*Actg1*)
	M	Two-way ANOVA	<0.0001	Vehicle vs HMBA in si-Control
			0.0079	Vehicle vs HMBA in si-*Myh9*

			0.0003	HMBA (si-Control vs si-*Myh9*)
	N		0.0002	Vehicle vs HMBA in si-Control
			0.6627	Vehicle vs HMBA in si-*Myh9*
			0.0026	HMBA (si-Control vs si-*Myh9*)
	O		<0.0001	Vehicle vs HMBA in si-Control
			0.0033	Vehicle vs HMBA in si-*Actg1*
			<0.0001	HMBA (si-Control vs si-*Actg1*)
	P		<0.0001	Vehicle vs HMBA in si-Control
			0.0738	Vehicle vs HMBA in si-*Actg1*
			<0.0001	HMBA (si-Control vs si-*Actg1*)
5	A	Two-tailed t test	<0.0001	
	B		<0.0001	
	C		0.0157	
	D		<0.0001	
	F	Two-way ANOVA	<0.0001	All
	G		<0.0001	All
	H	Two-tailed t test	0.0092	
	I	Two-tailed t test	0.0004	
	J	One-way ANOVA	<0.0001	*Npy*
			<0.0001	*Pomc*
	K		<0.0001	*Npy*
			<0.0001	*Pomc*
	L	Two-way ANOVA	0.0358	shControl (Saline vs HMBA), day 7
			0.0193	shControl (Saline vs HMBA), day 8
			0.0059	shControl (Saline vs HMBA), day 9
			0.0017	shControl (Saline vs HMBA), day 10
			0.0242	HMBA (shControl vs sh*Myh9*+sh*Actg1*), day 6
			0.0239	HMBA (shControl vs sh*Myh9*+sh*Actg1*), day 7
			0.0200	HMBA (shControl vs sh*Myh9*+sh*Actg1*), day 8
			0.0138	HMBA (shControl vs sh*Myh9*+sh*Actg1*), day 9
			0.0087	HMBA (shControl vs sh*Myh9*+sh*Actg1*), day 10
			0.0038	(%), shControl (Saline vs HMBA)
			0.9430	(%), shMyh9+shActg1 (Saline vs HMBA)
			0.9856	(%), Saline (shControl vs sh*Myh9*+sh*Actg1*)
			0.0097	(%), HMBA (shControl vs sh*Myh9*+sh*Actg1*)
	M	Two-way ANOVA	0.0159	shControl (Saline vs HMBA), day 4
			0.0003	shControl (Saline vs HMBA), day 5
			0.0036	HMBA (shControl vs sh*Myh9*+sh*Actg1*), day 1
			0.0575	HMBA (shControl vs sh*Myh9*+sh*Actg1*), day 2
			0.0582	HMBA (shControl vs sh*Myh9*+sh*Actg1*), day 3
			0.0105	HMBA (shControl vs sh*Myh9*+sh*Actg1*), day 5
	N	Two-way ANOVA	0.0383	shControl (Saline vs HMBA), day 9
			0.0186	shControl (Saline vs HMBA), day 10
			0.0329	HMBA (shControl vs sh*Myh9*+sh*Actg1*), day 8
			0.0197	HMBA (shControl vs sh*Myh9*+sh*Actg1*), day 9
			0.0104	HMBA (shControl vs sh*Myh9*+sh*Actg1*), day 10
			0.0038	(%), shControl (Saline vs HMBA)
			0.9983	(%), shMyh9+shActg1 (Saline vs HMBA)
			0.9873	(%), Saline (shControl vs sh*Myh9*+sh*Actg1*)
			0.0046	(%), HMBA (shControl vs sh*Myh9*+sh*Actg1*)
	O	Two-way ANOVA	0.0015	shControl (Saline vs HMBA), day 1
			0.0009	shControl (Saline vs HMBA), day 2
			0.0003	shControl (Saline vs HMBA), day 3
			<0.0001	shControl (Saline vs HMBA), day 4 ~ day 7
			0.0031	shControl (Saline vs HMBA), day 8
			0.0440	HMBA (shControl vs sh*Myh9*+sh*Actg1*), day 3
			0.0033	HMBA (shControl vs sh*Myh9*+sh*Actg1*), day 4
			<0.0001	HMBA (shControl vs sh*Myh9*+sh*Actg1*), day 5
			0.0017	HMBA (shControl vs sh*Myh9*+sh*Actg1*), day 6
			0.0200	HMBA (shControl vs sh*Myh9*+sh*Actg1*), day 7
6	B	Two-way ANOVA	0.0007	Vehicle vs HMBA in si-Control
			0.1394	Vehicle vs HMBA in si-*p53*
			0.5516	Vehicle (si-Control vs si-*p53*)
			0.0003	HMBA (si-Control vs si-*p53*)
	D	Two-way ANOVA	<0.0001	Vehicle vs HMBA in si-Control
			0.7118	Vehicle vs HMBA in si-*p53*
			0.1406	Vehicle (si-Control vs si-*p53*)

			<0.0001	HMBA (si-Control vs si-*p53*)
	F	Two-way ANOVA	0.0433	0.5 h in *Hexim1*
			<0.0001	1 h in *Hexim1*
			<0.0001	2 h in *Hexim1*
	I	Two-way ANOVA	<0.0001	0.5 h in *Hexim1*
			<0.0001	1 h in *Hexim1*
			<0.0001	2 h in *Hexim1*
7	A	Two-way ANOVA	<0.0001, 0.0401	HEXIM1
			<0.0001	p53 (all)
	B	Two-way ANOVA	<0.0001, 0.0203	HEXIM1
			<0.0001	p53 (all)
	E	Two-way ANOVA	<0.0001	p53
			<0.0001	HEXIM1
			0.0428	H3K9Ac
			0.0339	H3K4me3
			>0.9999	H3K27me3
	F	Two-way ANOVA	<0.0001	p53
			<0.0001	HEXIM1
			<0.0001	H3K9Ac
			0.9876	H3K4me3
			0.8217	H3K27me3
EV 1	A	Two-tailed t test	0.0555	Eating
	C	One-way ANOVA	0.9095	Pre vs post in HMBA
			<0.0001	Pre vs post in LiCl
			<0.0001	Post (HMBA vs LiCl)
	D	Two-tailed t test	0.0464	Eating
	F	One-way ANOVA	0.9977	Pre vs post in HMBA
			<0.0001	Pre vs post in LiCl
			<0.0001	Post (HMBA vs LiCl)
	G	Two-tailed t test	0.0004	Eating
	I	One-way ANOVA	>0.9999	Pre vs post in HMBA
			<0.0001	Pre vs post in LiCl
			0.0003	Post (HMBA vs LiCl)
EV 3	A	Two-way ANOVA	<0.0001	All in *Myh9* panel
			0.0002	Vehicle (si-Control vs si-*Actg1*)
			0.0002	HMBA (si-Control vs si-*Actg1*)
	B	Two-way ANOVA	<0.0001	All
	C	Two-way ANOVA	<0.0001	All in *Myh9* and *Actg1* panel
			<0.0001	si-Control (Vehicle vs HMBA) in *Hexim1*
			0.3985	si-*Myh9*+si-*Actg1* (Vehicle vs HMBA) in *Hexim1*
			<0.0001	HMBA (si-control vs si-*Myh9*+si-*Actg1*) in *Hexim1*
	E	Two-way ANOVA	0.0017	si-Control (Vehicle vs HMBA)
			0.9776	si-*Myh9*+si-*Actg1* (Vehicle vs HMBA)
			0.0029	HMBA (si-control vs si-*Myh9*+si-*Actg1*)
	F	Two-way ANOVA	0.0001	Vehicle (si-control vs si-*Myh9*+si-*Actg1*) in *Myh9*
			<0.0001	HMBA (si-control vs si-*Myh9*+si-*Actg1*) in *Myh9*
			<0.0001	All in *Actg1* panel
			<0.0001	si-Control (Vehicle vs HMBA) in *Hexim1*
			0.2983	si-*Myh9*+si-*Actg1* (Vehicle vs HMBA) in *Hexim1*
			<0.0001	HMBA (si-control vs si-*Myh9*+si-*Actg1*) in *Hexim1*
	H	Two-way ANOVA	<0.0001	si-Control (Vehicle vs HMBA)
			0.8015	si-*Myh9*+si-*Actg1* (Vehicle vs HMBA)
			<0.0001	HMBA (si-control vs si-*Myh9*+si-*Actg1*)
EV 4	C	Two-tailed t test	0.3925	
	D	Two-tailed t test	0.4754	
EV 5	C	Two-way ANOVA	<0.0001	Vehicle vs HMBA
			<0.0001	Vehicle+LMB vs HMBA+LMB
			0.9701	Vehicle vs Vehicle+LMB
			<0.0001	HMBA vs HMBA+LMB
	D	Two-way ANOVA	<0.0001	Vehicle vs HMBA
			<0.0001	Vehicle+LMB vs HMBA+LMB
			>0.9999	Vehicle vs Vehicle+LMB
			0.0239	HMBA vs HMBA+LMB

References

Asghari A, Ishikawa T, Hiramitsu S, Lee WR, Umetani J, Bui L, Korach KS, Umetani M (2019) 27-Hydroxycholesterol promotes adiposity and mimics adipogenic diet-induced inflammatory signaling. *Endocrinology* 160: 2485-2494

Campia I, Lussiana C, Pescarmona G, Ghigo D, Bosia A, Riganti C (2009) Geranylgeraniol prevents the cytotoxic effects of mevastatin in THP-1 cells, without decreasing the beneficial effects on cholesterol synthesis. *Br J Pharmacol* 158: 1777-1786

Fu J, Gaetani S, Oveisi F, Lo Verme J, Serrano A, Rodriguez De Fonseca F, Rosengarth A, Luecke H, Di Giacomo B, Tarzia G, Piomelli D (2003) Oleylethanolamide regulates feeding and body weight through activation of the nuclear receptor PPAR-alpha. *Nature* 425: 90-93

Fu W, Ma Y, Li L, Liu J, Fu L, Guo Y, Zhang Z, Li J, Jiang H (2020) Artemether regulates metaflammation to improve glycolipid metabolism in db/db mice. *Diabetes Metab Syndr Obes* 13: 1703-1713

Harris LH (1961) The protein anabolic action of mestanolone. *J Clin Endocrinol Metab* 21: 1099-1105

Yang D, Yang J, Shi D, Xiao D, Chen YT, Black C, Deng R, Yan B (2012) Hypolipidemic agent Z-guggulsterone: metabolism interplays with induction of carboxylesterase and bile salt export pump. *J Lipid Res* 53: 529-539

Yousef IM, Tuchweber B, Morazain R, Kugelmass R, Gauvin M, Roy CC, Weber AM (1988) Cholesterol synthesis in the pathogenesis of lithocholic acid-induced cholestasis. *Lipids* 23: 230-233
