# Supplementary figures and images for: HMBA ameliorates obesity by MYH9‐ and ACTG1‐dependent regulation of hypothalamic neuropeptides
Source: EMBO Mol Med. 2023 Nov 20;15(12):e18024. doi: 10.15252/emmm.202318024 (PMC10701615; doi:10.15252/emmm.202318024)

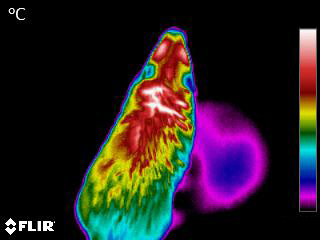

Supplement: Supplementary file 4 — Source Data for Figure 1 [file EMMM-15-e18024-s001.zip › Fig_1K-L/Fig_1K_Thermal_image_HMBA.jpg]

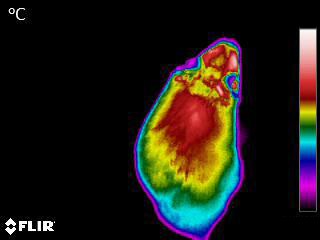

Supplement: Supplementary file 4 — Source Data for Figure 1 [file EMMM-15-e18024-s001.zip › Fig_1K-L/Fig_1K_Thermal_image_saline.jpg]

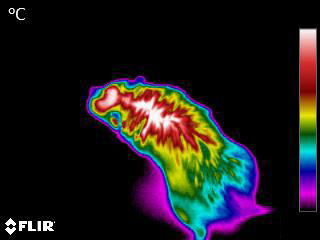

Supplement: Supplementary file 5 — Source Data for Figure 2 [file EMMM-15-e18024-s008.zip › Fig_2F-G/Fig_2F_Thermal_image_HMBA.jpg]

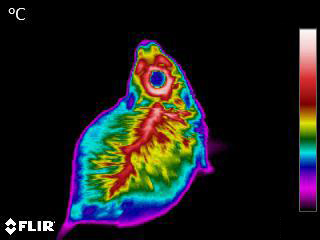

Supplement: Supplementary file 5 — Source Data for Figure 2 [file EMMM-15-e18024-s008.zip › Fig_2F-G/Fig_2F_Thermal_image_Saline.jpg]

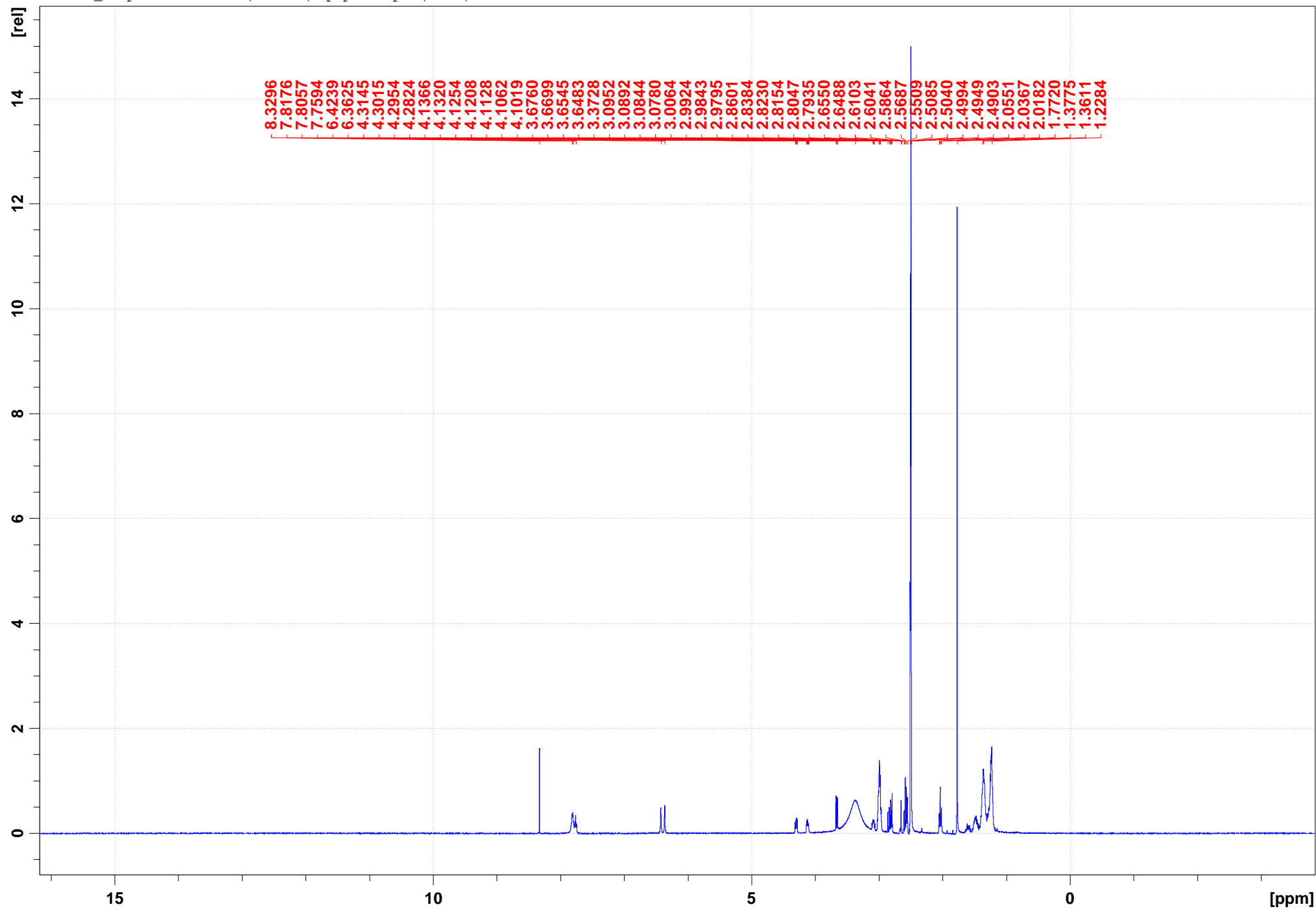

Supplement: Supplementary file 7 — Source Data for Figure 4 [file EMMM-15-e18024-s009.zip › Fig_4C/Fig_4C_NMR_raw.pdf]

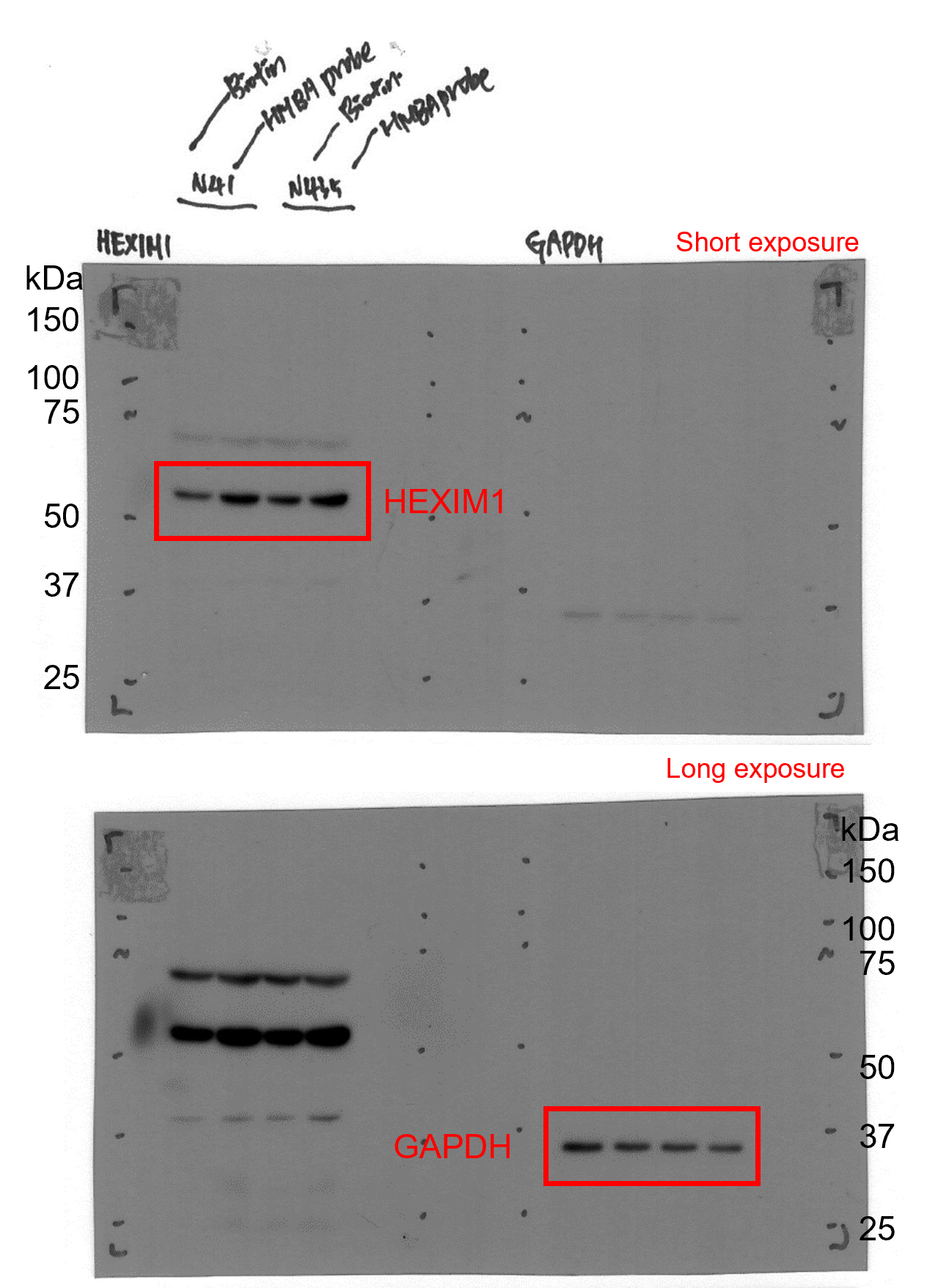

Supplement: Supplementary file 7 — Source Data for Figure 4 [file EMMM-15-e18024-s009.zip › Fig_4D/Fig_4D_Uncropped_blots.tiff]

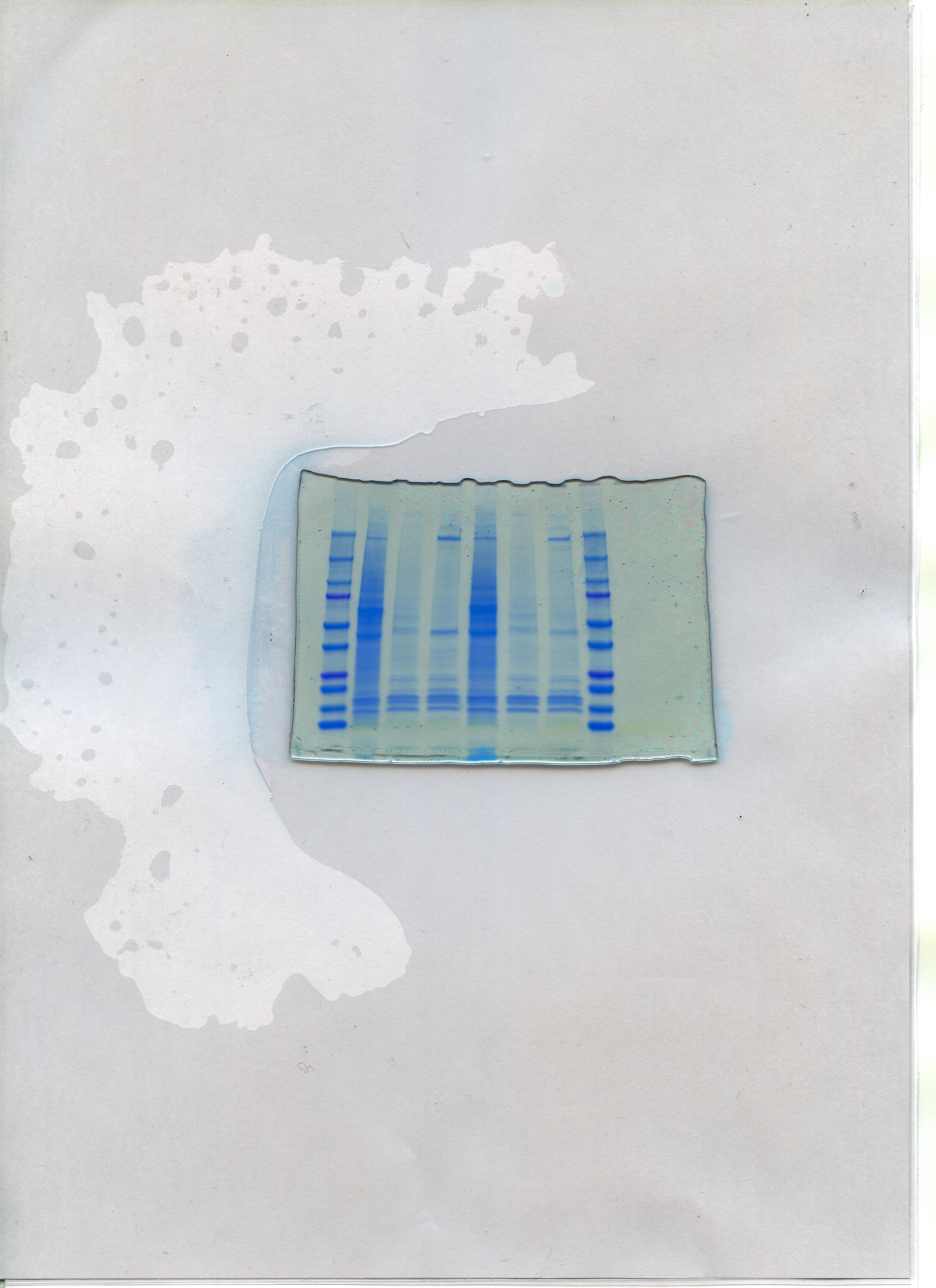

Supplement: Supplementary file 7 — Source Data for Figure 4 [file EMMM-15-e18024-s009.zip › Fig_4F/Fig_4F_gel_image.tiff]

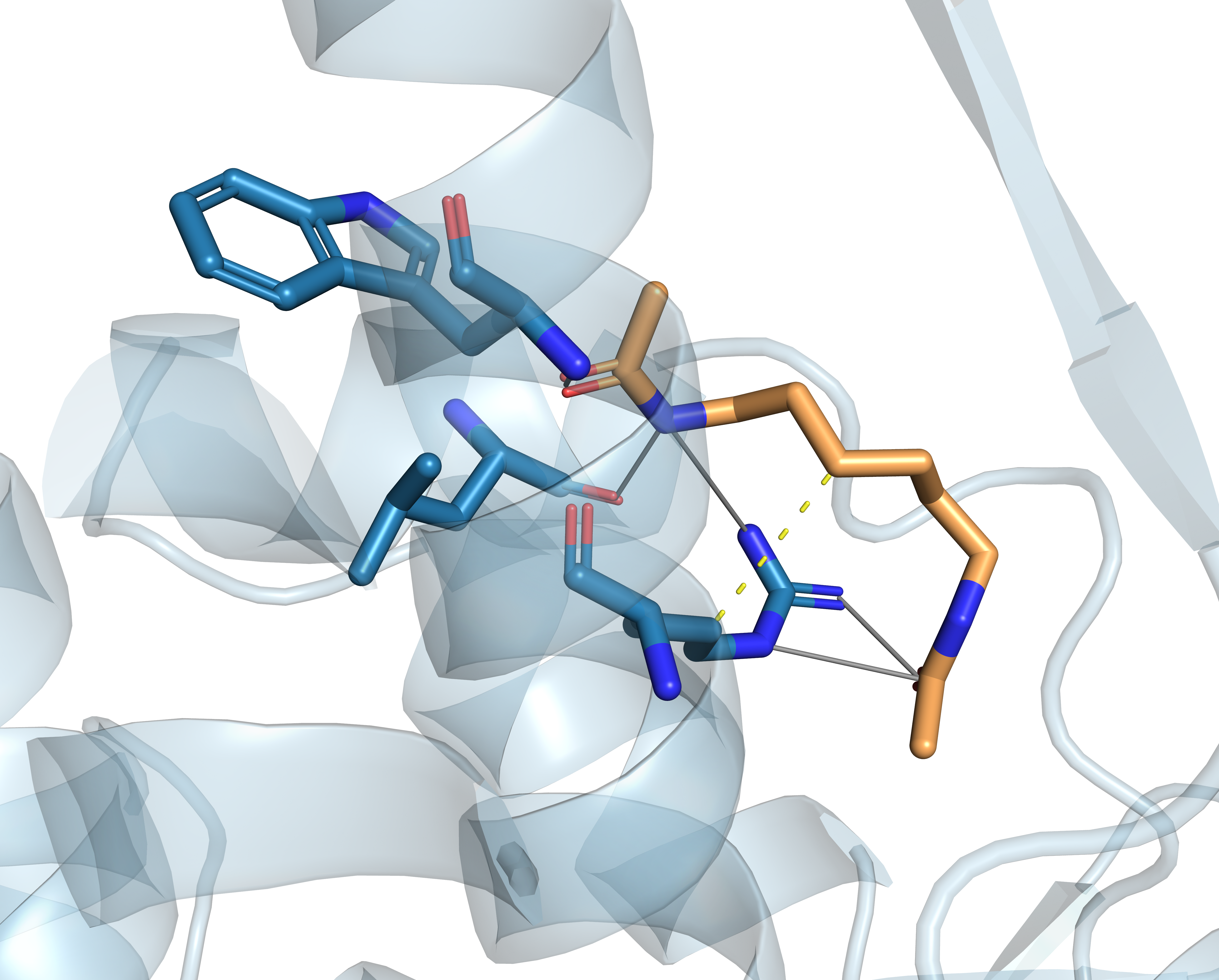

Supplement: Supplementary file 7 — Source Data for Figure 4 [file EMMM-15-e18024-s009.zip › Fig_4G/Fig_4G_left_panel.tiff]

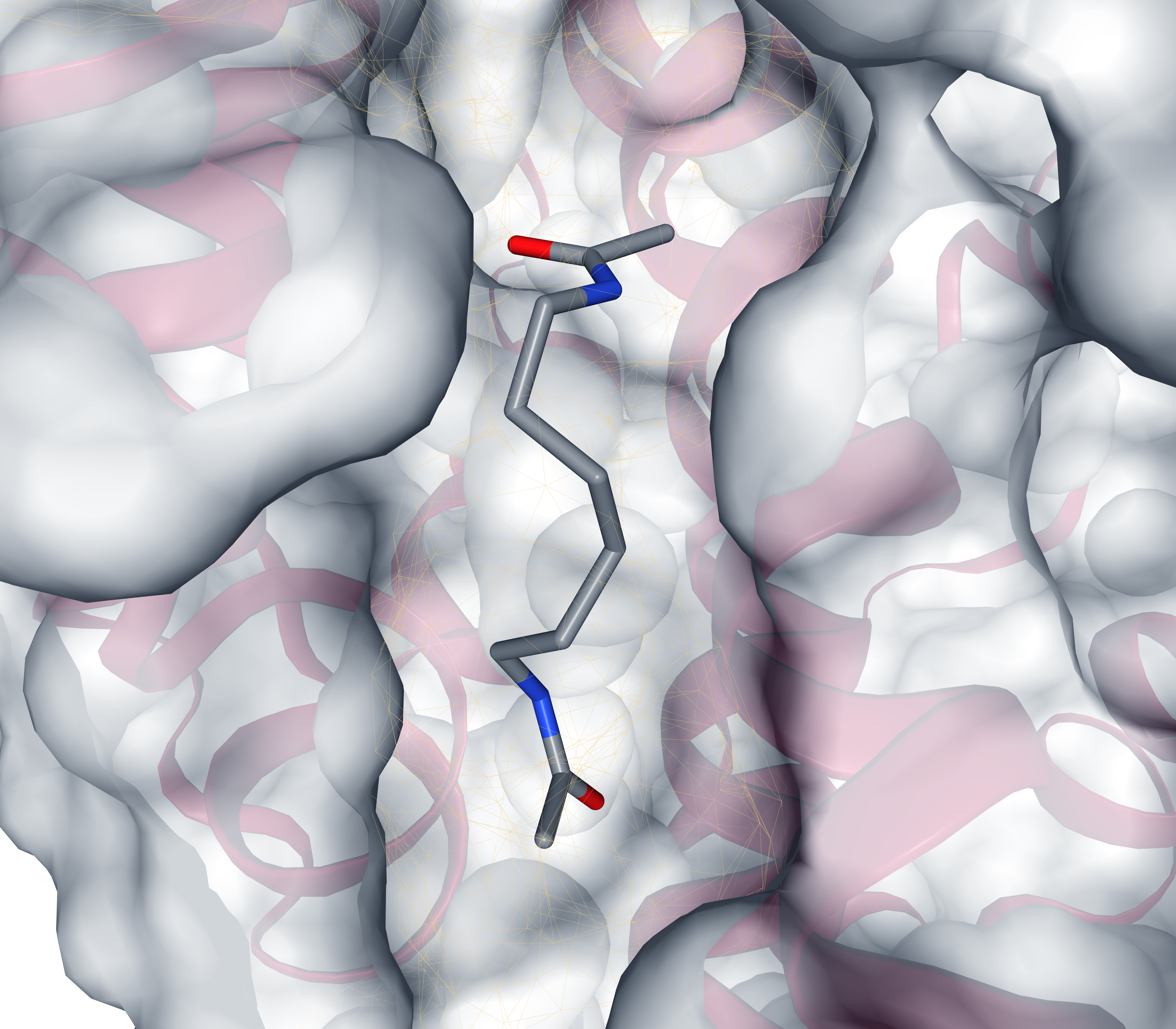

Supplement: Supplementary file 7 — Source Data for Figure 4 [file EMMM-15-e18024-s009.zip › Fig_4G/Fig_4G_right_panel.tiff]

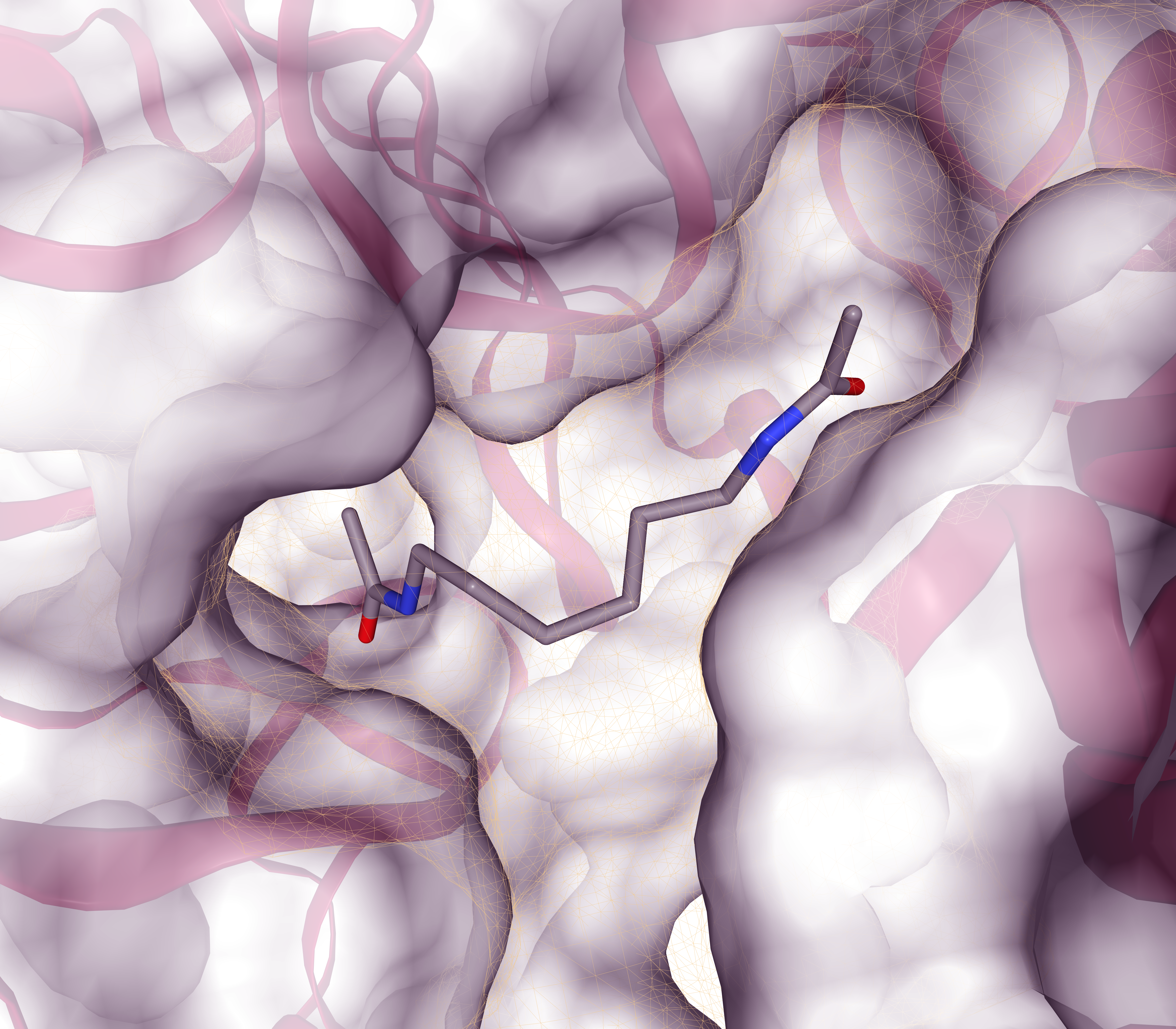

Supplement: Supplementary file 7 — Source Data for Figure 4 [file EMMM-15-e18024-s009.zip › Fig_4H/Fig_4H_left_panel.tiff]

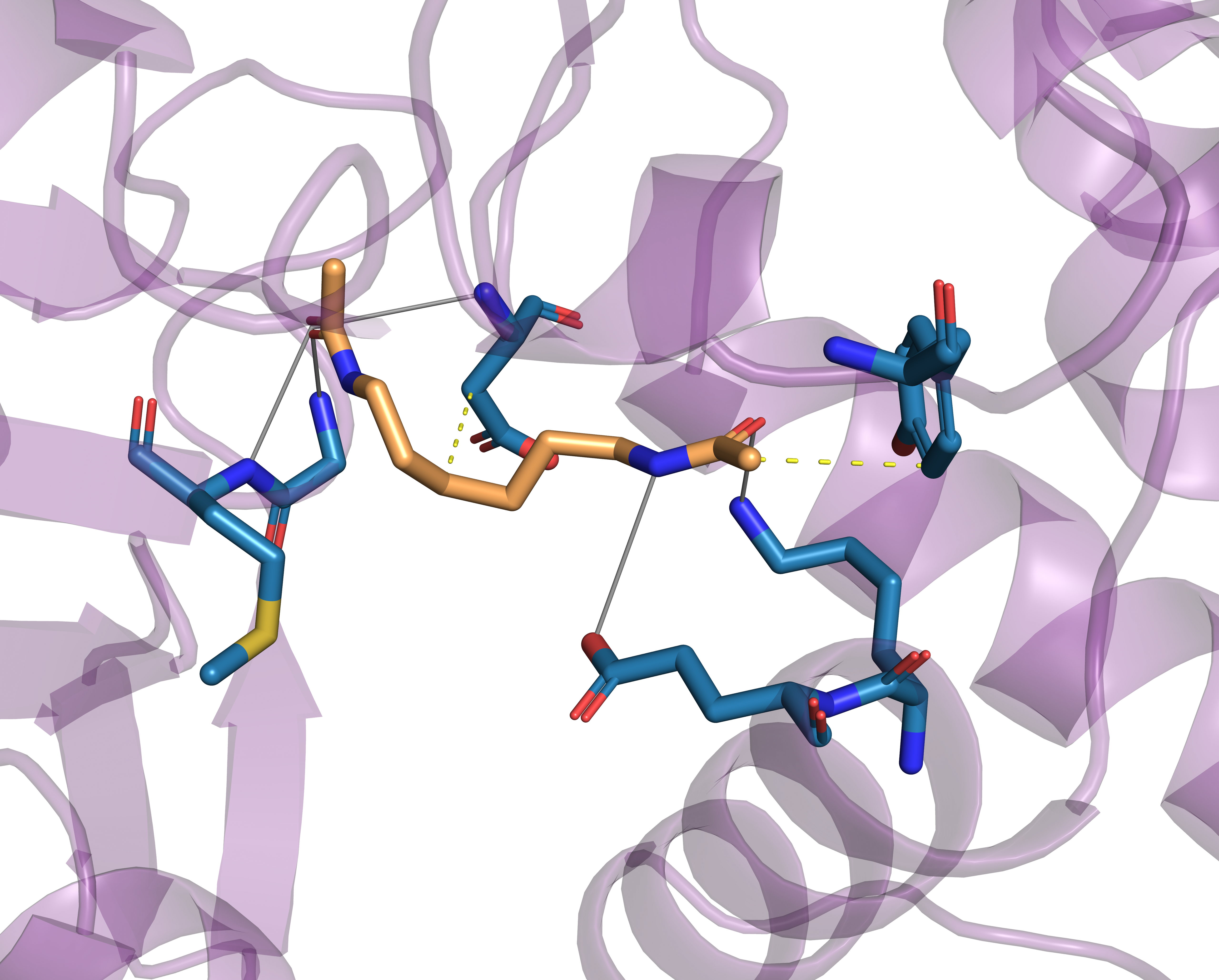

Supplement: Supplementary file 7 — Source Data for Figure 4 [file EMMM-15-e18024-s009.zip › Fig_4H/Fig_4H_right_panel.tiff]

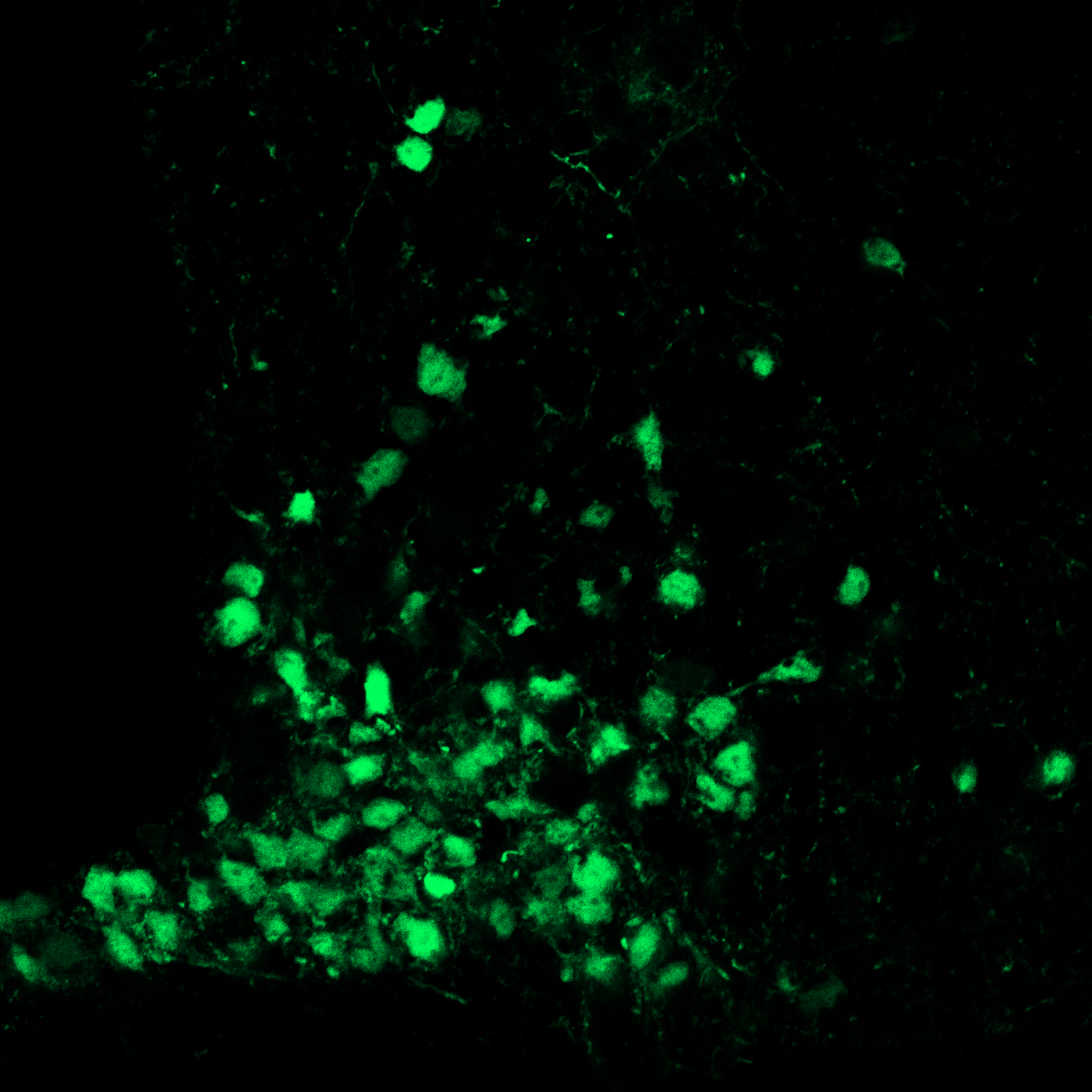

Supplement: Supplementary file 8 — Source Data for Figure 5 [file EMMM-15-e18024-s005.zip › Fig_5A/Fig_5A_NPY_HMBA.tiff]

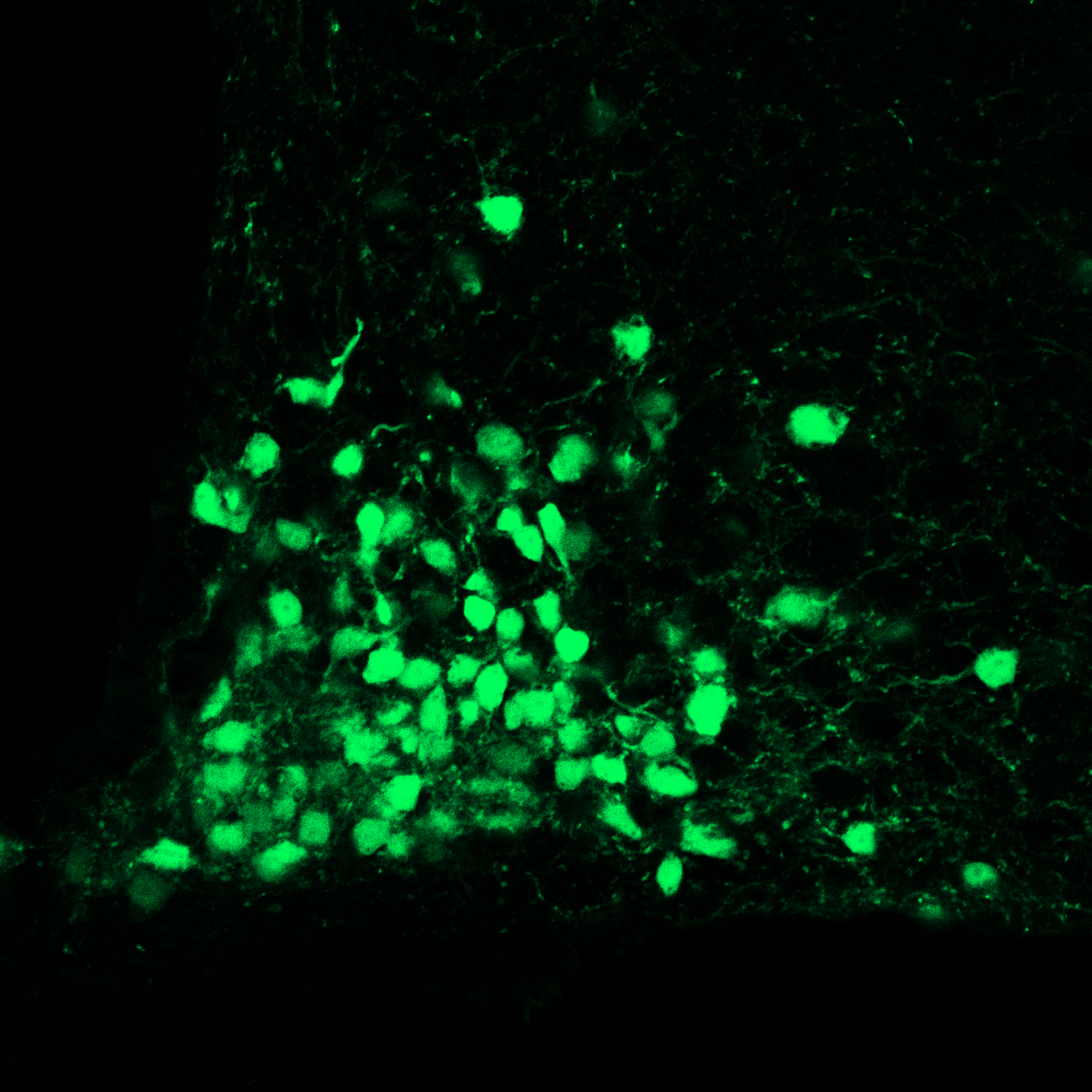

Supplement: Supplementary file 8 — Source Data for Figure 5 [file EMMM-15-e18024-s005.zip › Fig_5A/Fig_5A_NPY_saline.tiff]

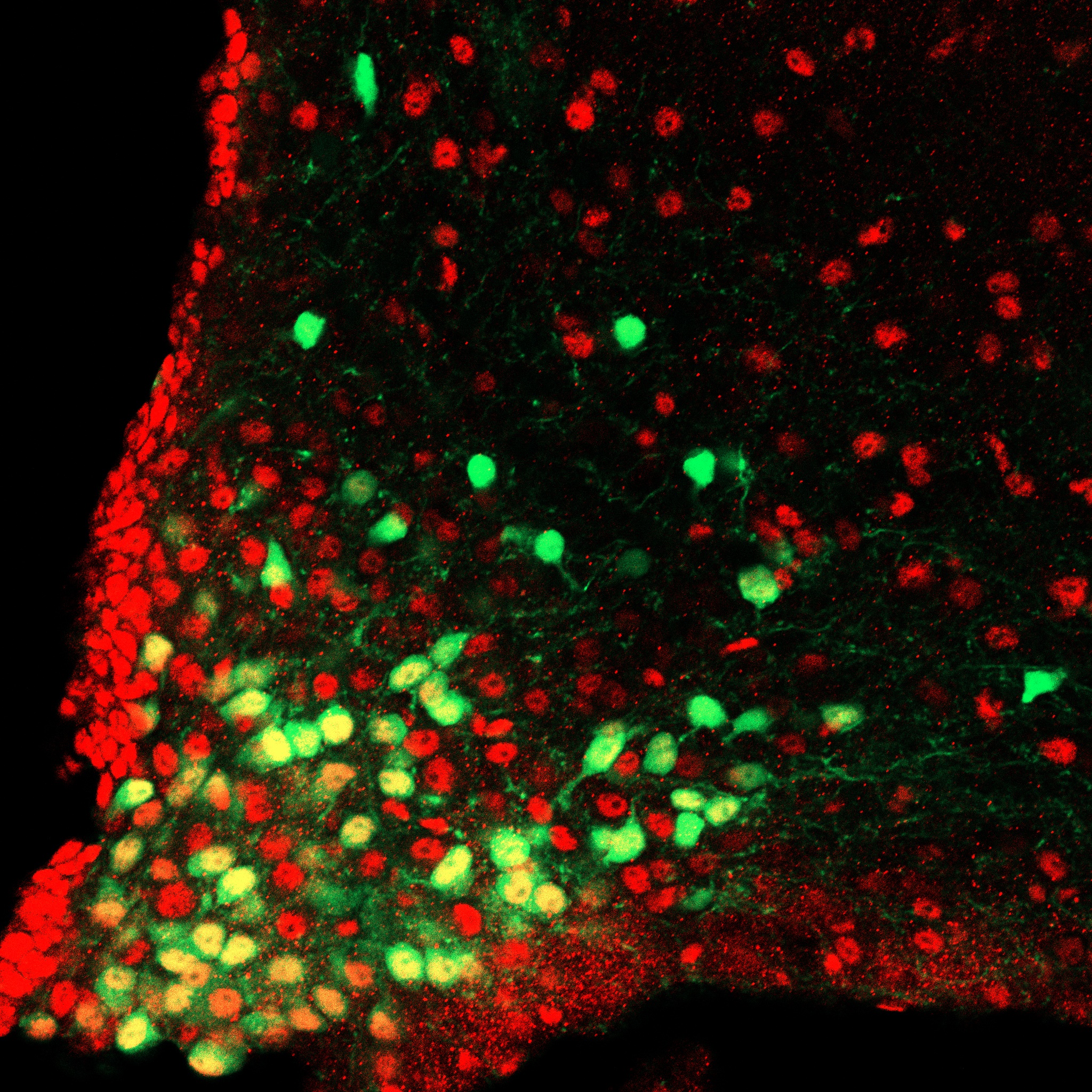

Supplement: Supplementary file 8 — Source Data for Figure 5 [file EMMM-15-e18024-s005.zip › Fig_5B/Fig_5B_NPY_HEXIM1_HMBA.tiff]

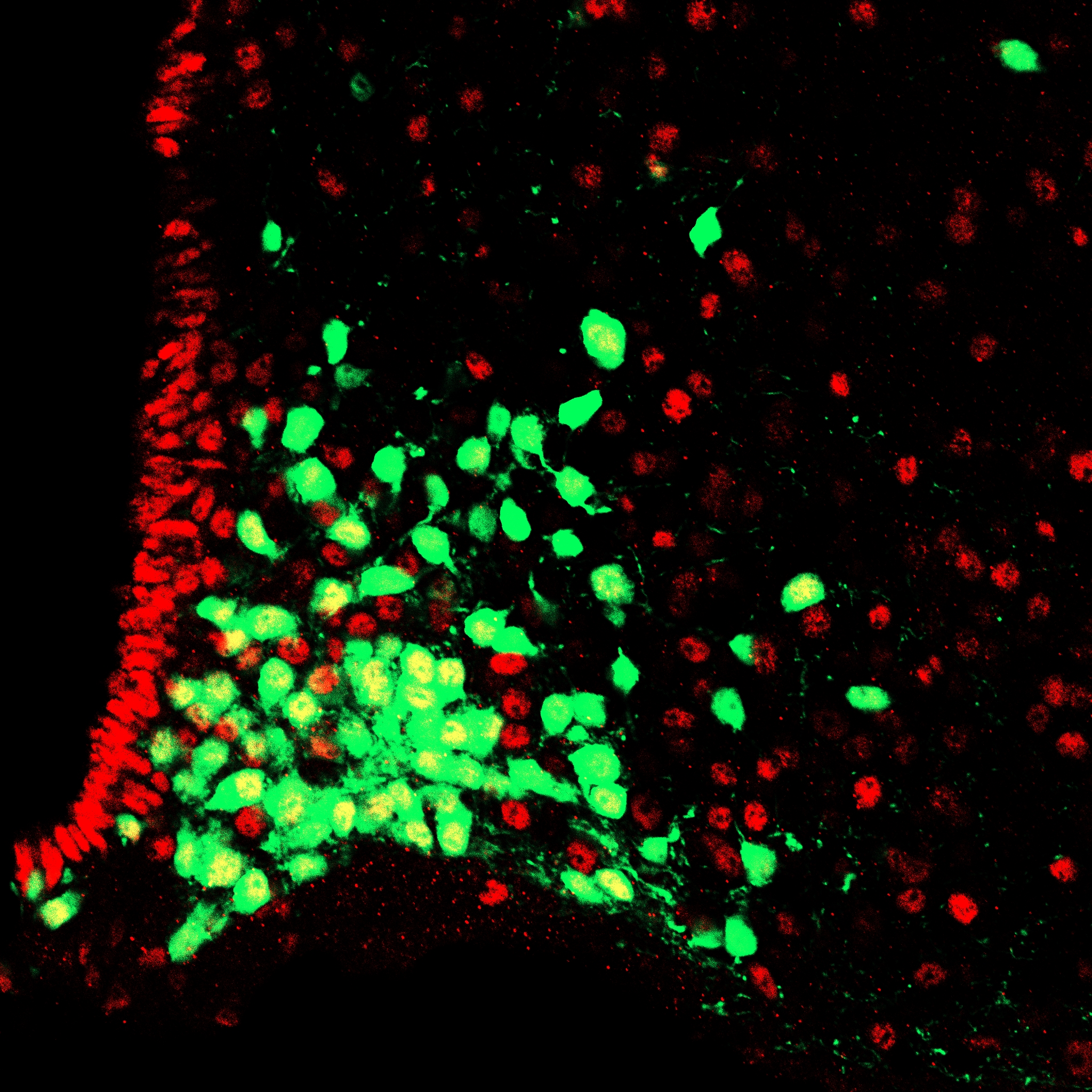

Supplement: Supplementary file 8 — Source Data for Figure 5 [file EMMM-15-e18024-s005.zip › Fig_5B/Fig_5B_NPY_HEXIM1_Saline.tiff]

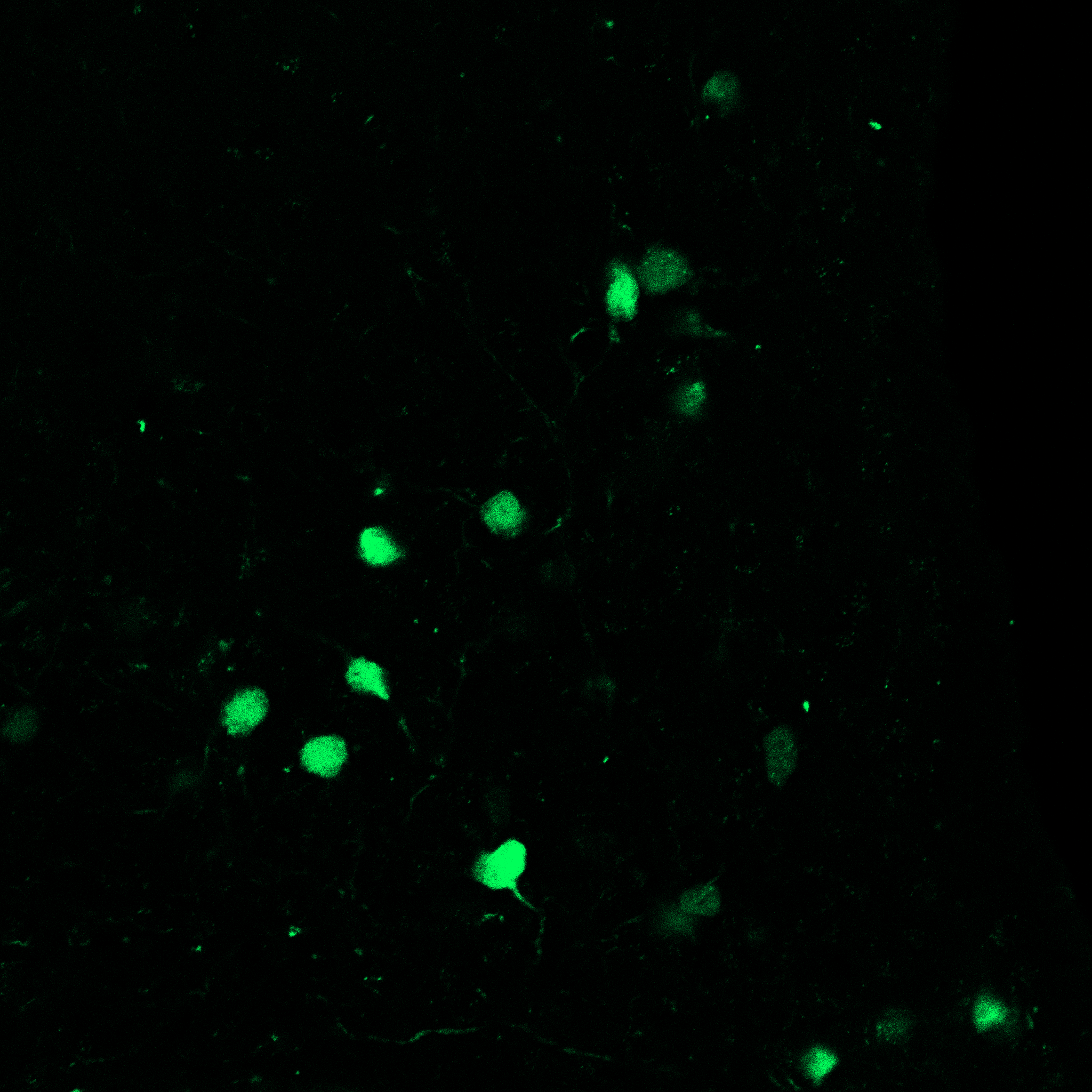

Supplement: Supplementary file 8 — Source Data for Figure 5 [file EMMM-15-e18024-s005.zip › Fig_5C/Fig_5C_POMC_HMBA.tiff]

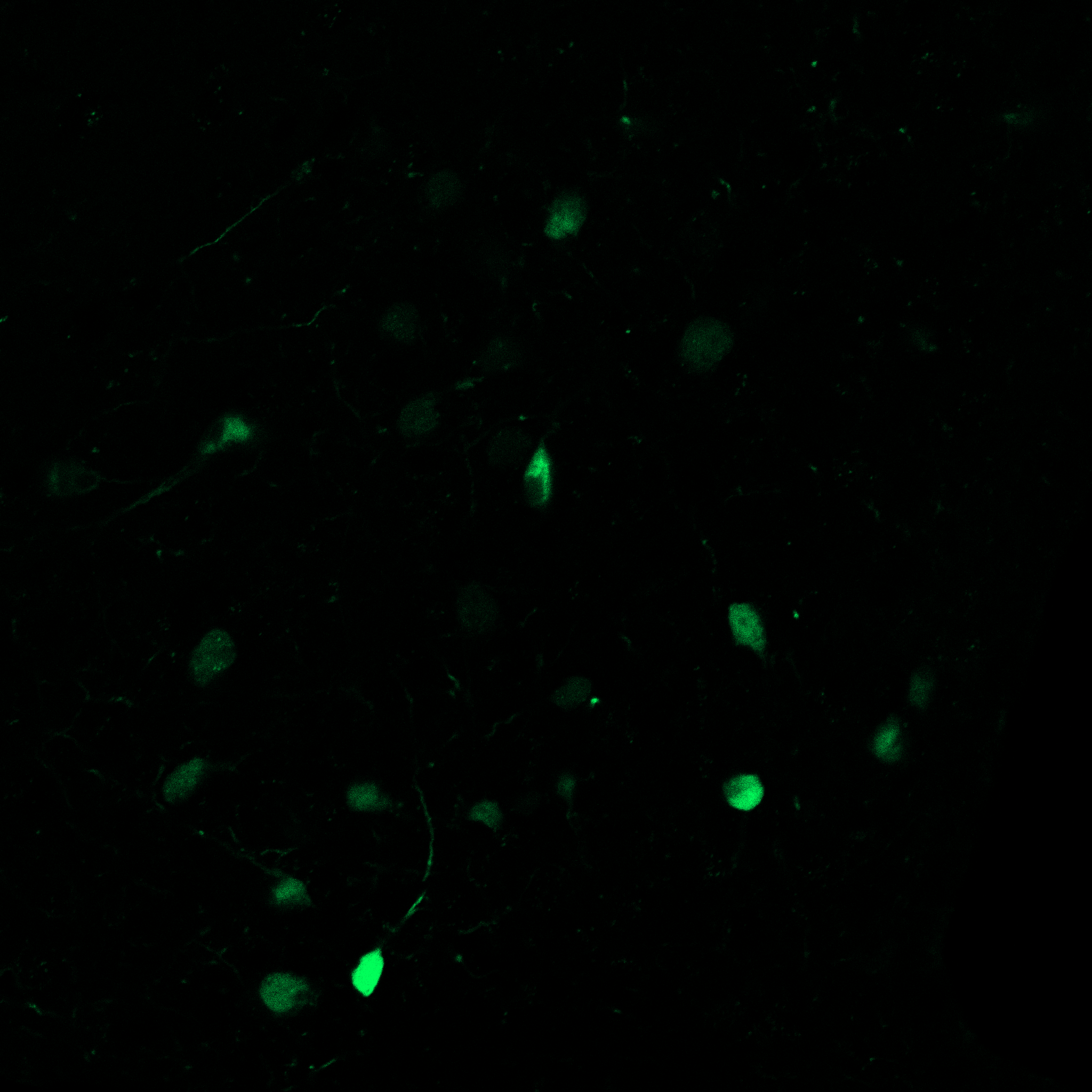

Supplement: Supplementary file 8 — Source Data for Figure 5 [file EMMM-15-e18024-s005.zip › Fig_5C/Fig_5C_POMC_Saline.tiff]

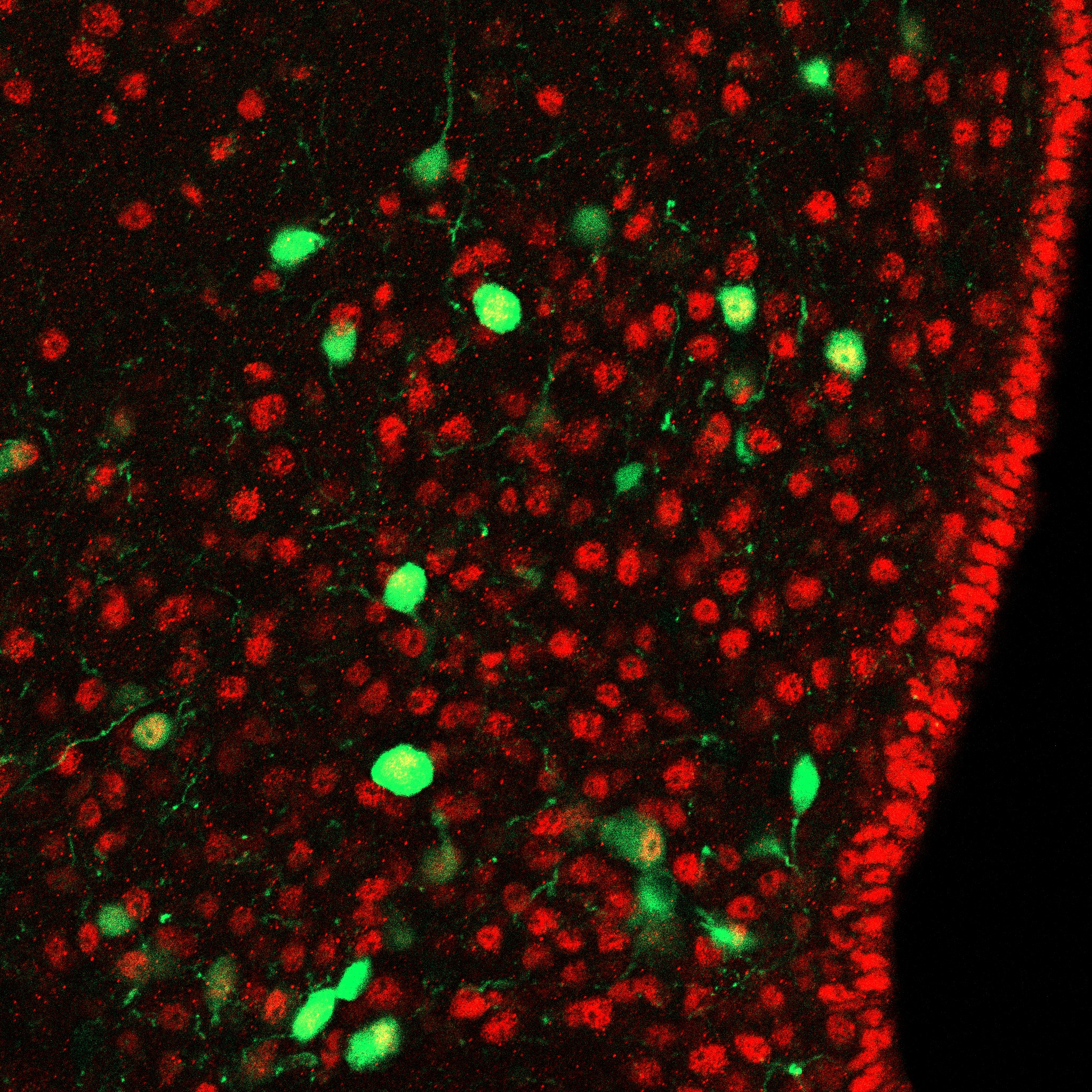

Supplement: Supplementary file 8 — Source Data for Figure 5 [file EMMM-15-e18024-s005.zip › Fig_5D/Fig_5D_POMC_HEXIM1_HMBA.tiff]

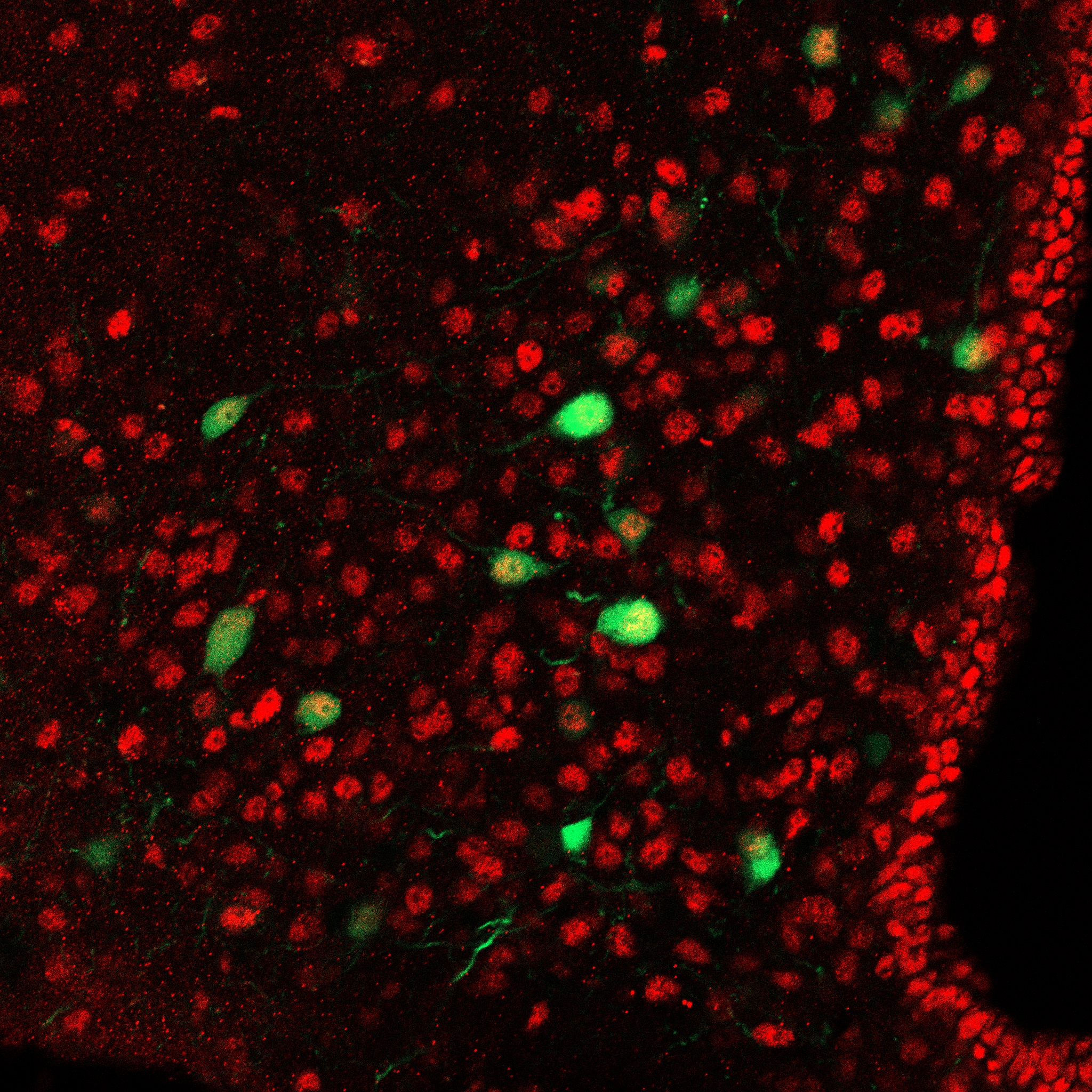

Supplement: Supplementary file 8 — Source Data for Figure 5 [file EMMM-15-e18024-s005.zip › Fig_5D/Fig_5D_POMC_HEXIM1_Saline.tiff]

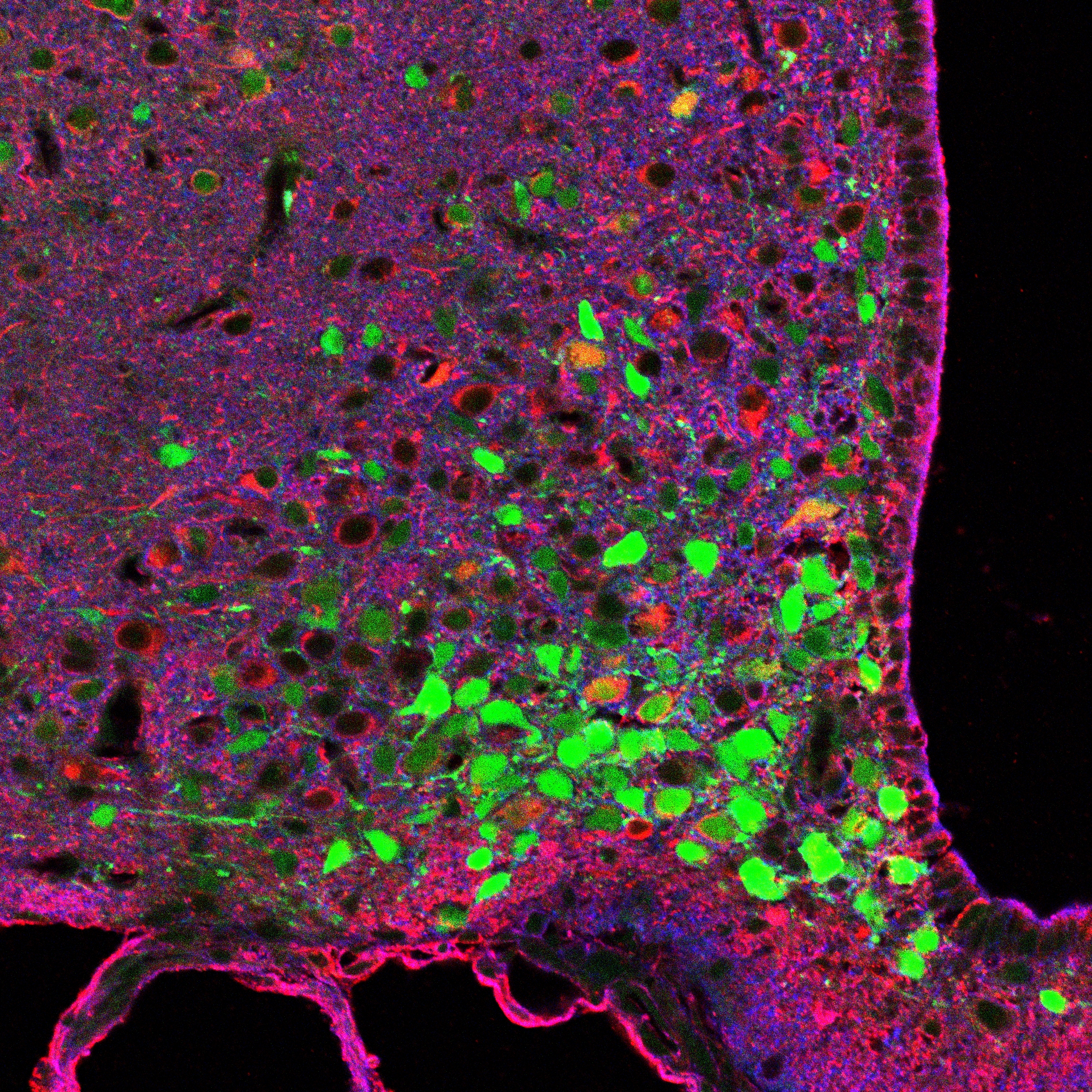

Supplement: Supplementary file 8 — Source Data for Figure 5 [file EMMM-15-e18024-s005.zip › Fig_5F/Fig_5F_shControl.tiff]

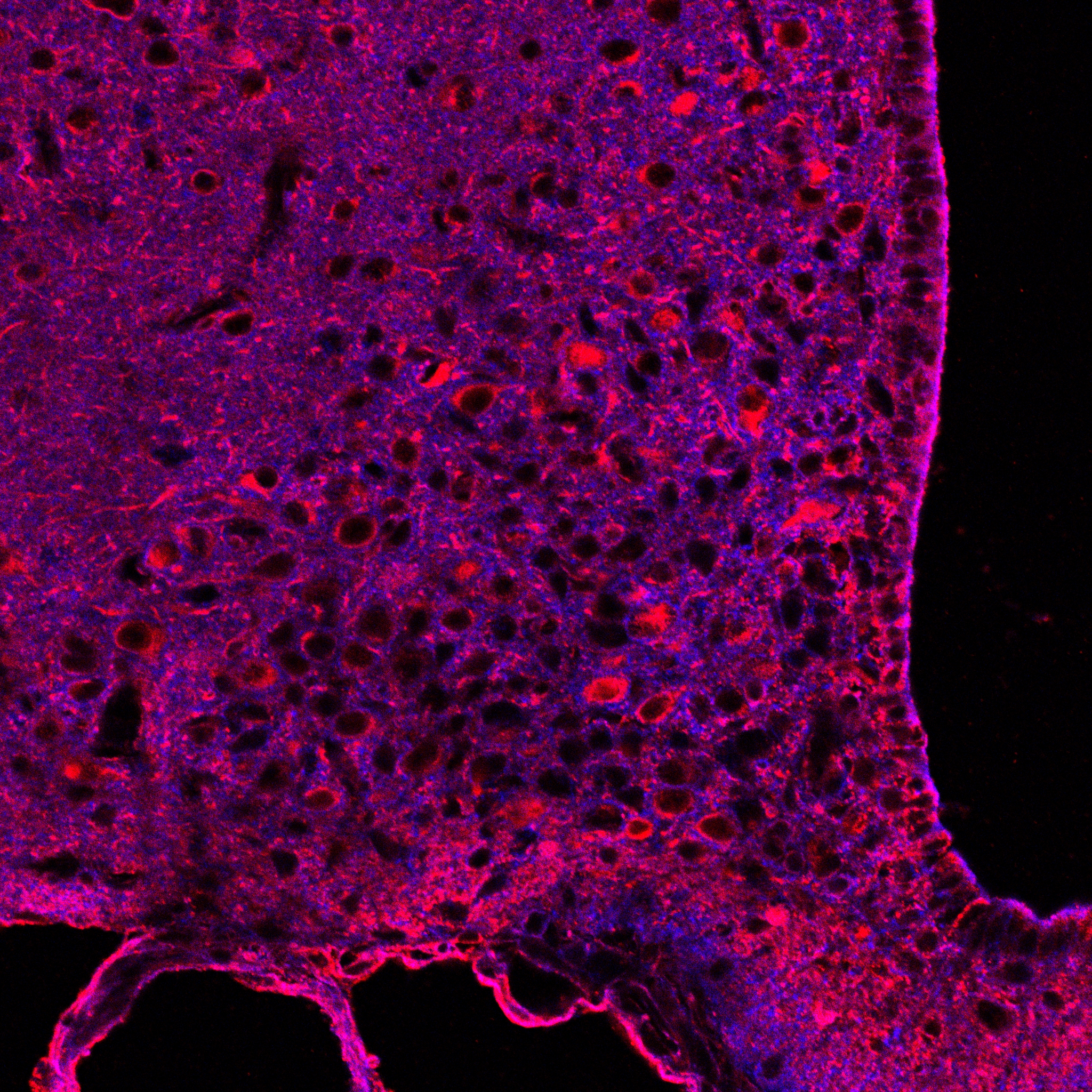

Supplement: Supplementary file 8 — Source Data for Figure 5 [file EMMM-15-e18024-s005.zip › Fig_5F/Fig_5F_shControl_EGFP(-).tiff]

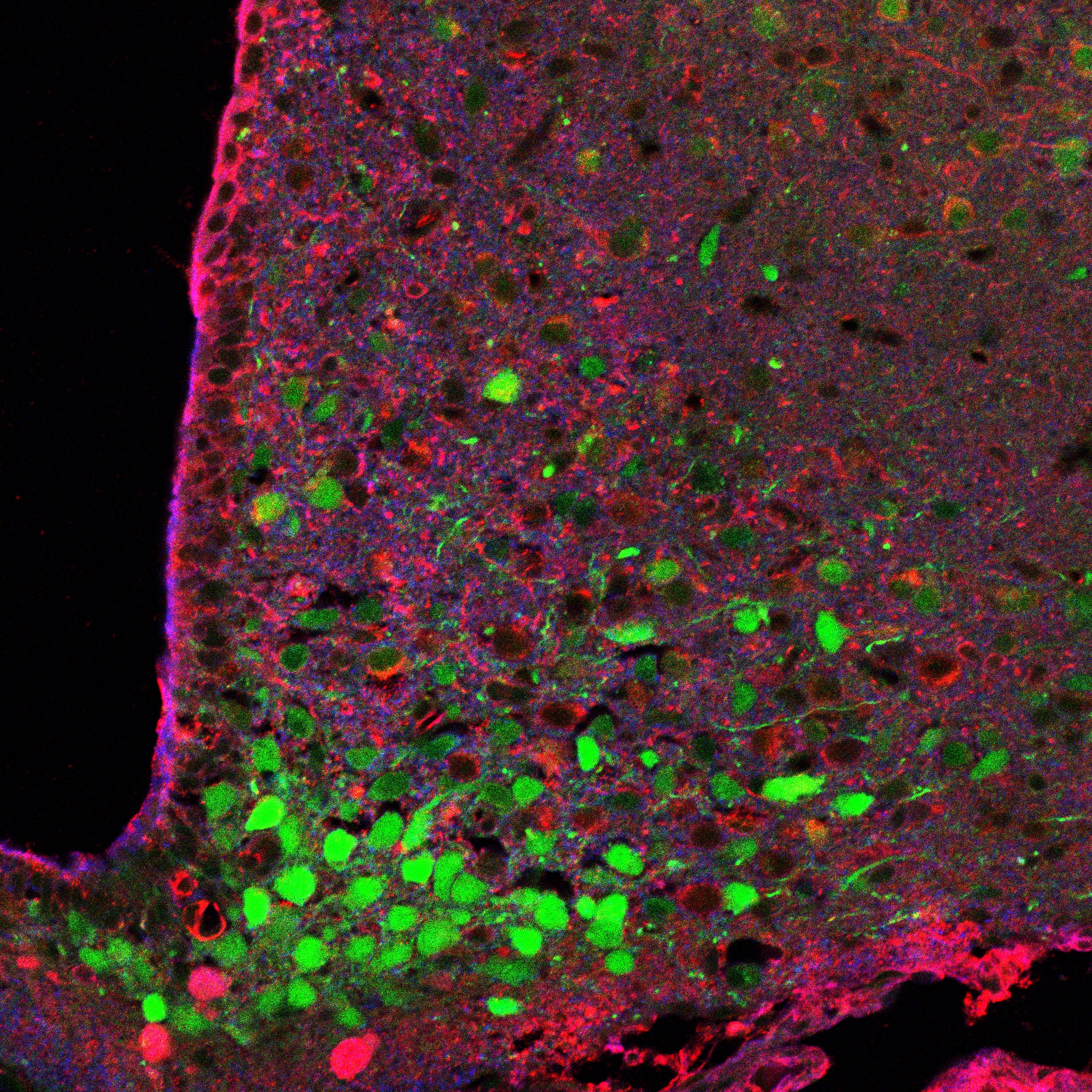

Supplement: Supplementary file 8 — Source Data for Figure 5 [file EMMM-15-e18024-s005.zip › Fig_5F/Fig_5F_shMyh9shActg1.tiff]

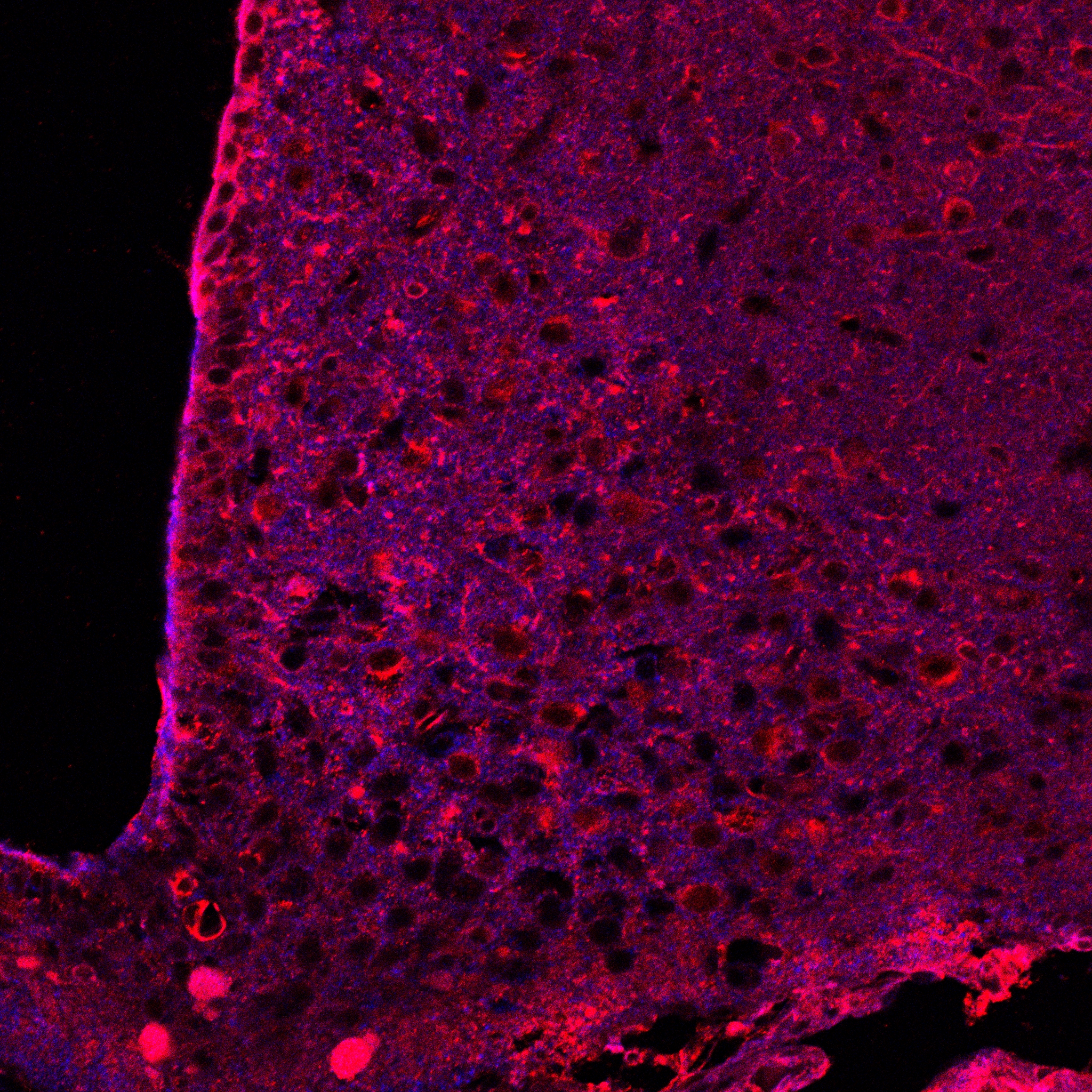

Supplement: Supplementary file 8 — Source Data for Figure 5 [file EMMM-15-e18024-s005.zip › Fig_5F/Fig_5F_shMyh9shActg1_EGFP(-).tiff]

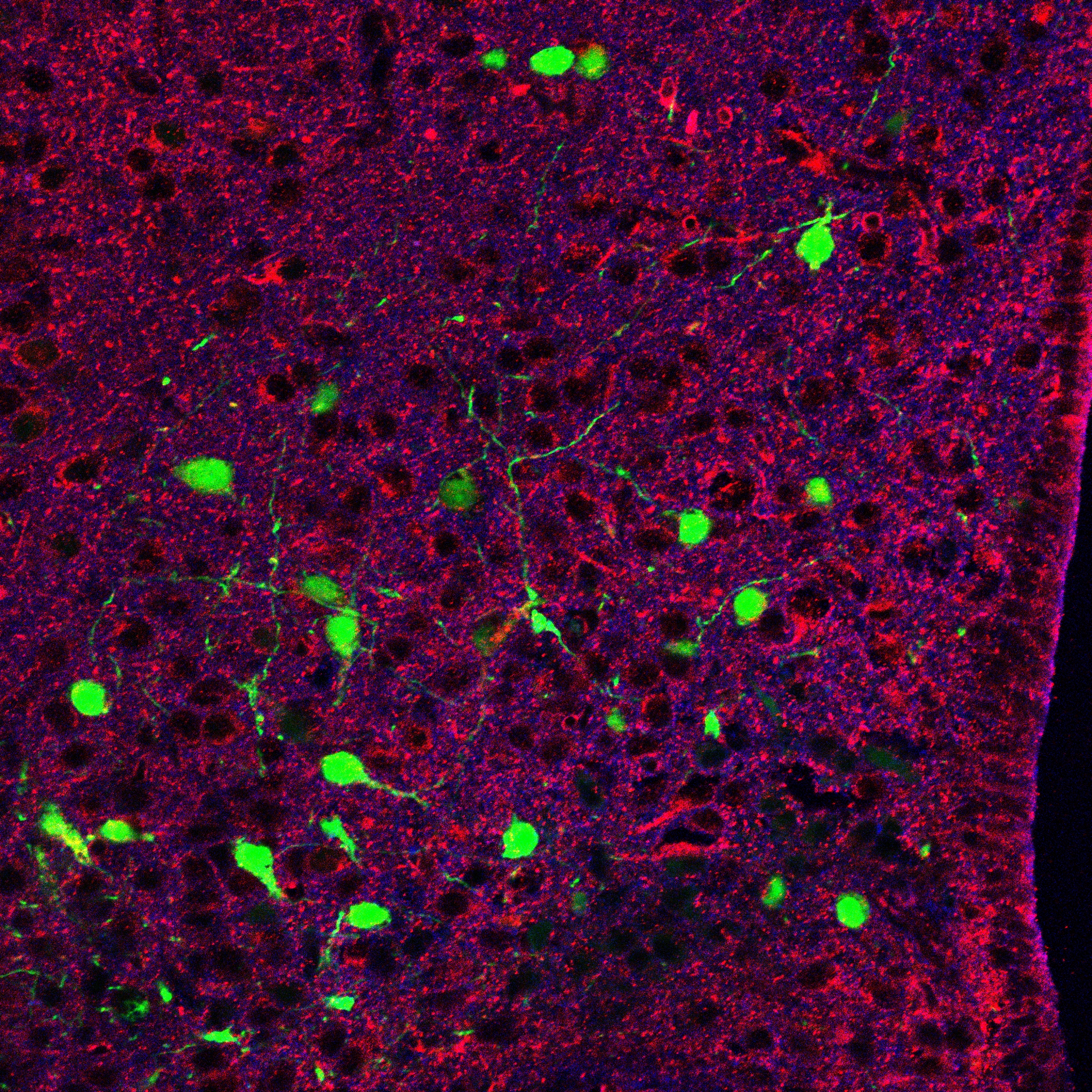

Supplement: Supplementary file 8 — Source Data for Figure 5 [file EMMM-15-e18024-s005.zip › Fig_5G/Fig_5G_shControl.tiff]

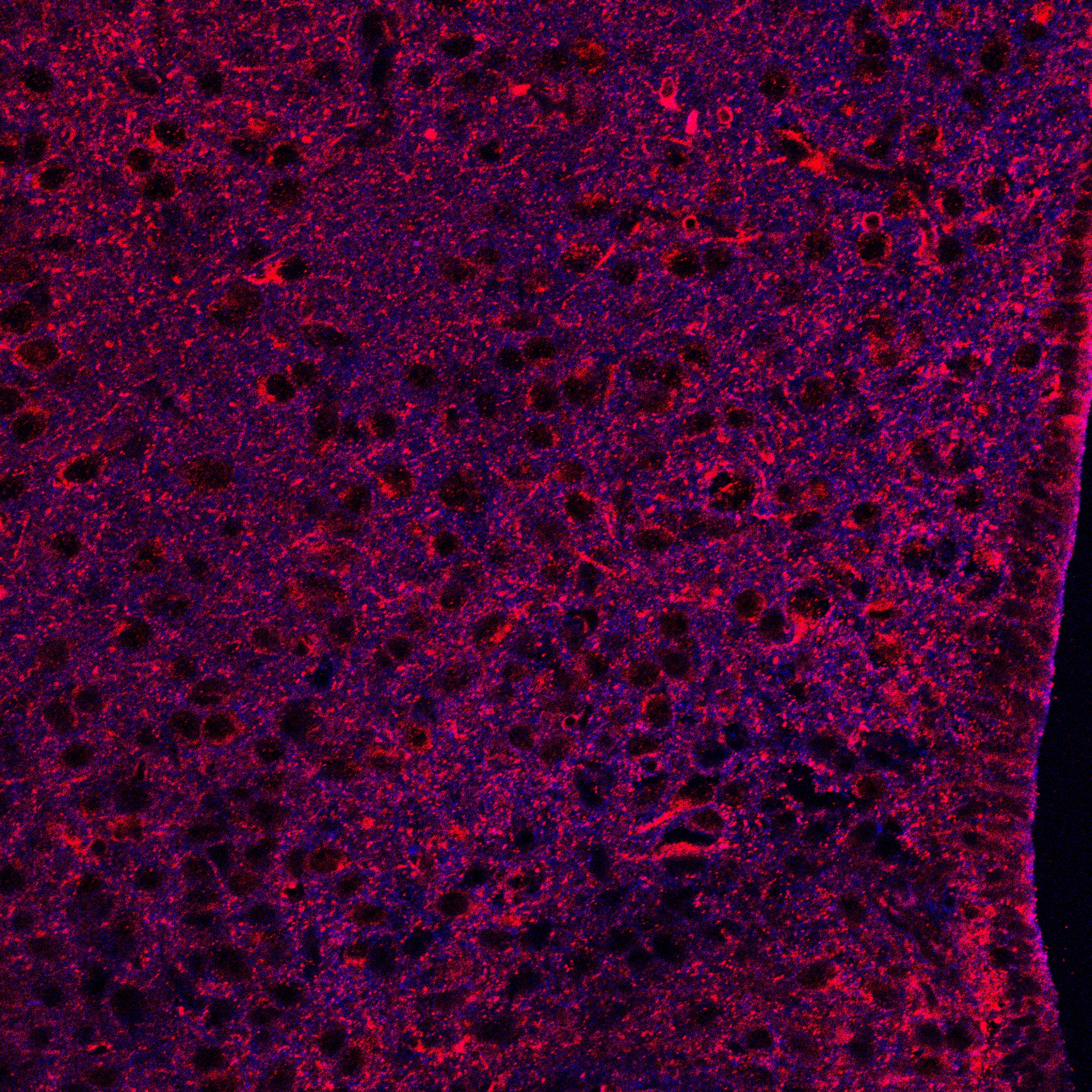

Supplement: Supplementary file 8 — Source Data for Figure 5 [file EMMM-15-e18024-s005.zip › Fig_5G/Fig_5G_shControl_EGFP(-).tiff]

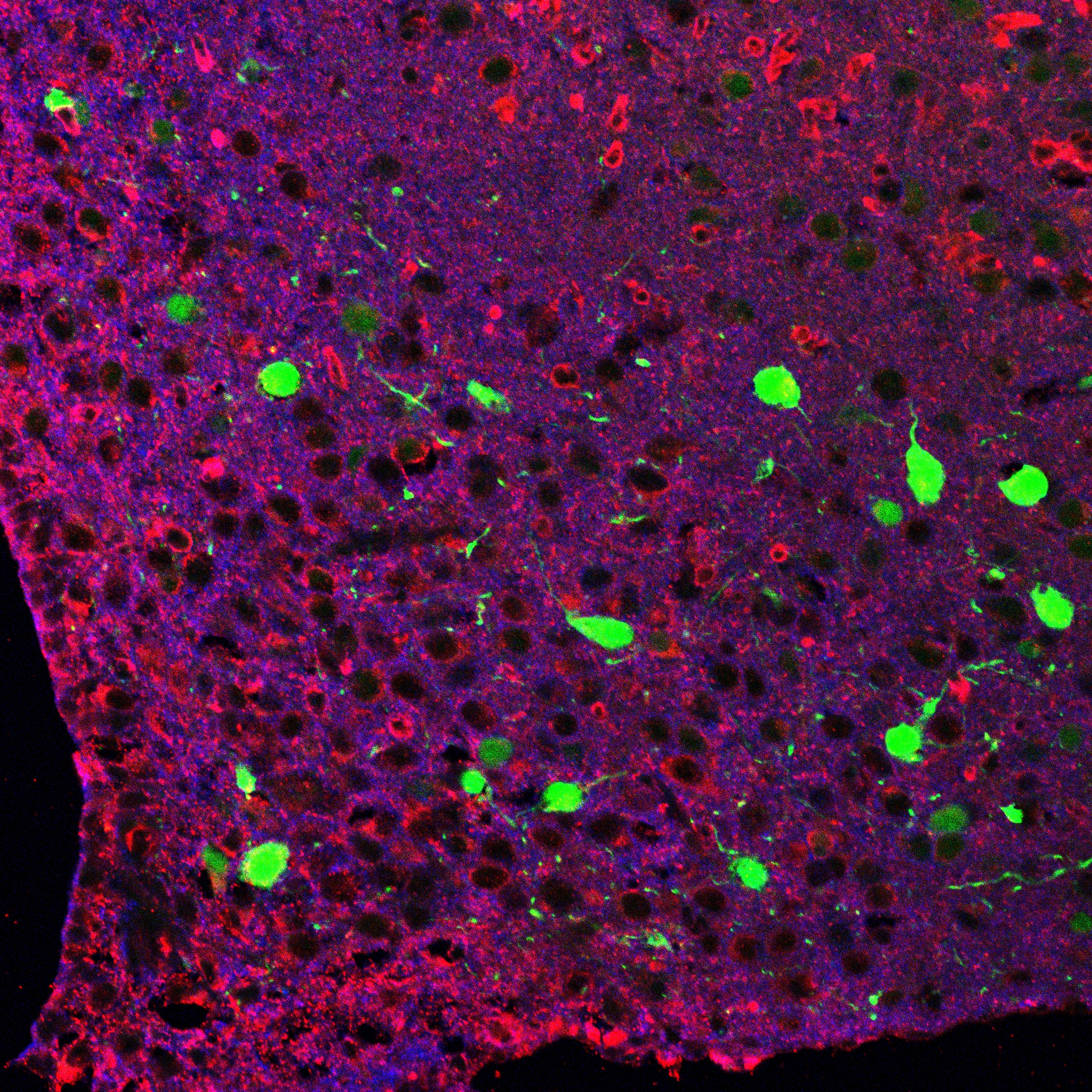

Supplement: Supplementary file 8 — Source Data for Figure 5 [file EMMM-15-e18024-s005.zip › Fig_5G/Fig_5G_shMyh9shActg1.tiff]

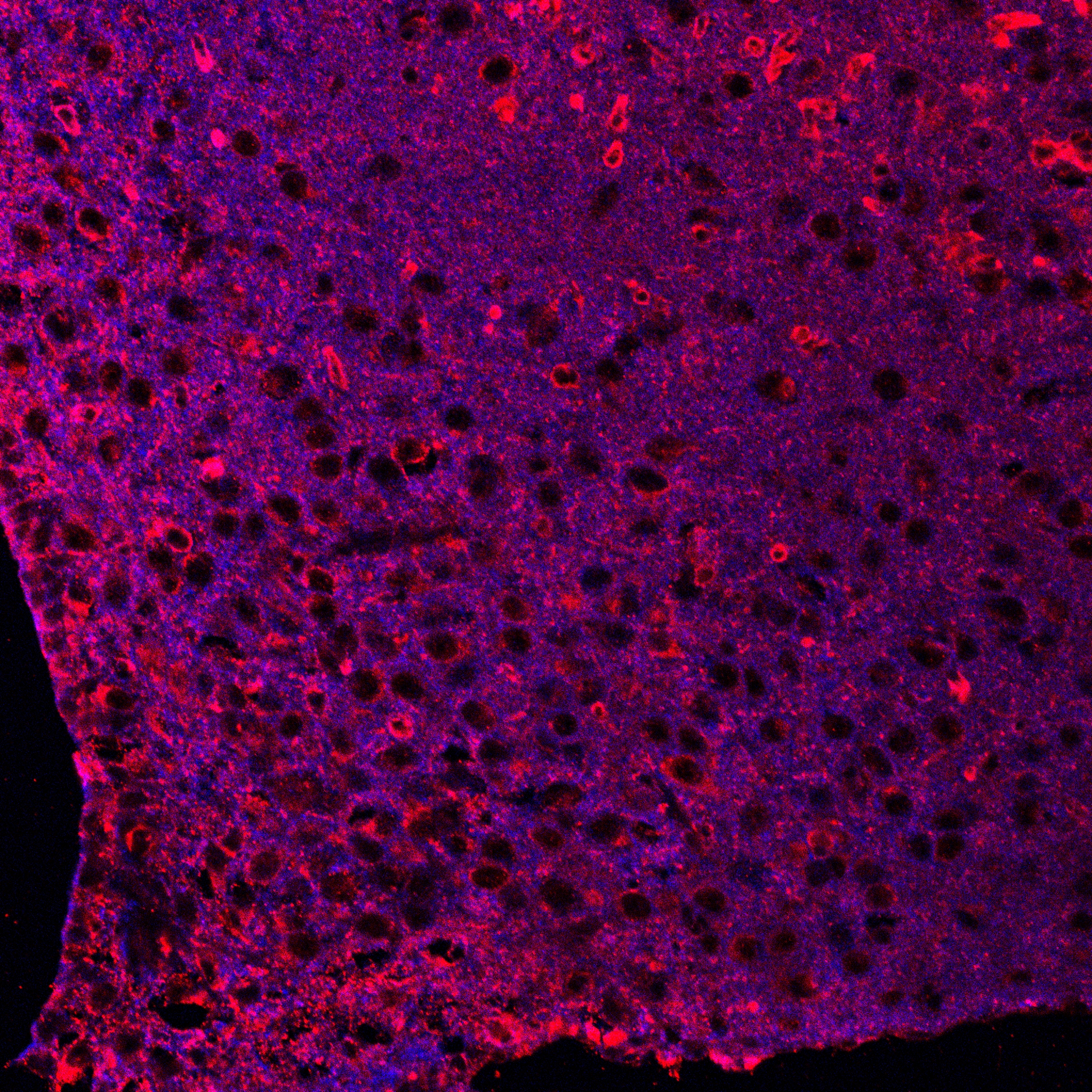

Supplement: Supplementary file 8 — Source Data for Figure 5 [file EMMM-15-e18024-s005.zip › Fig_5G/Fig_5G_shMyh9shActg1_EGFP(-).tiff]

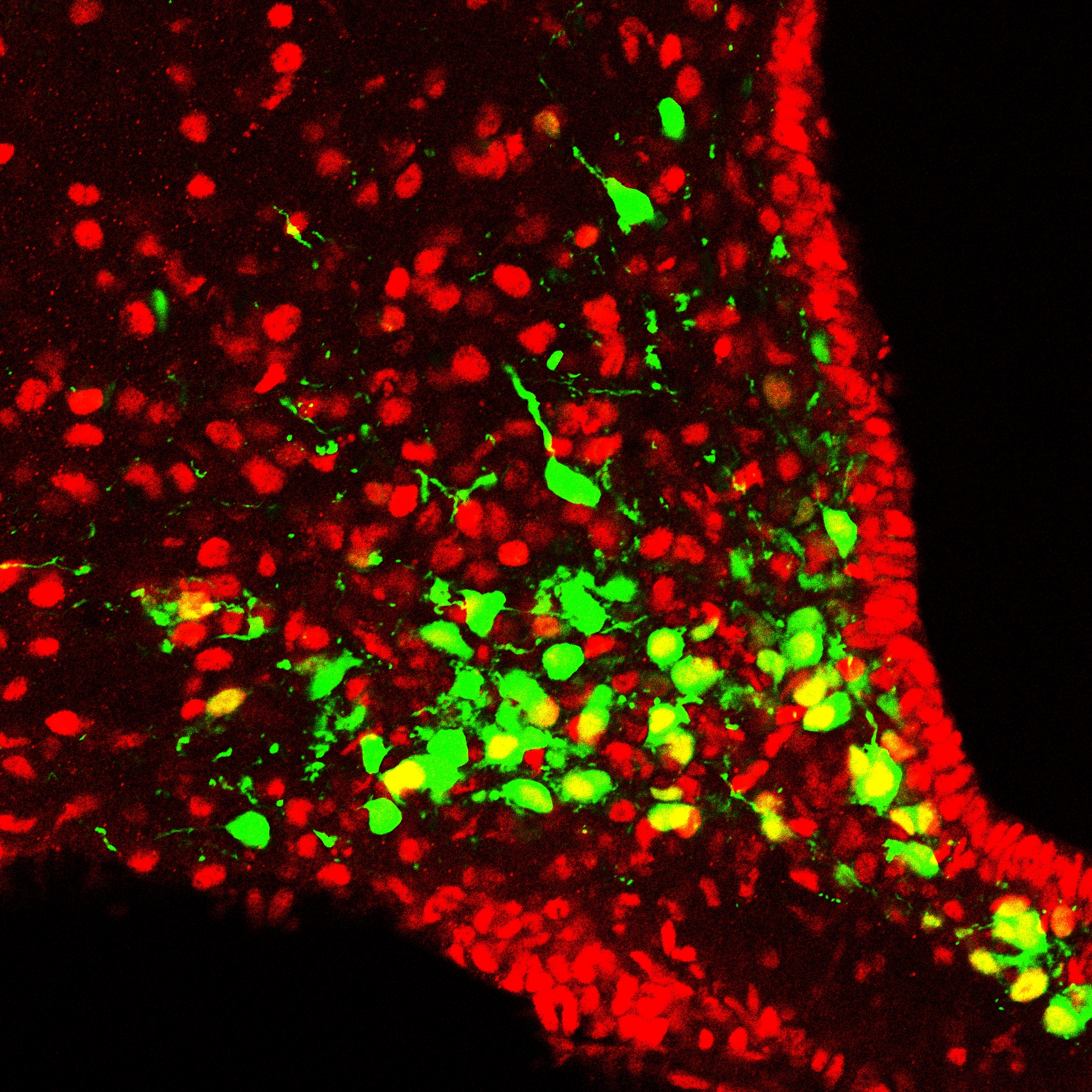

Supplement: Supplementary file 8 — Source Data for Figure 5 [file EMMM-15-e18024-s005.zip › Fig_5H/Fig_5H_EGFP_HEXIM1_shControl.tiff]

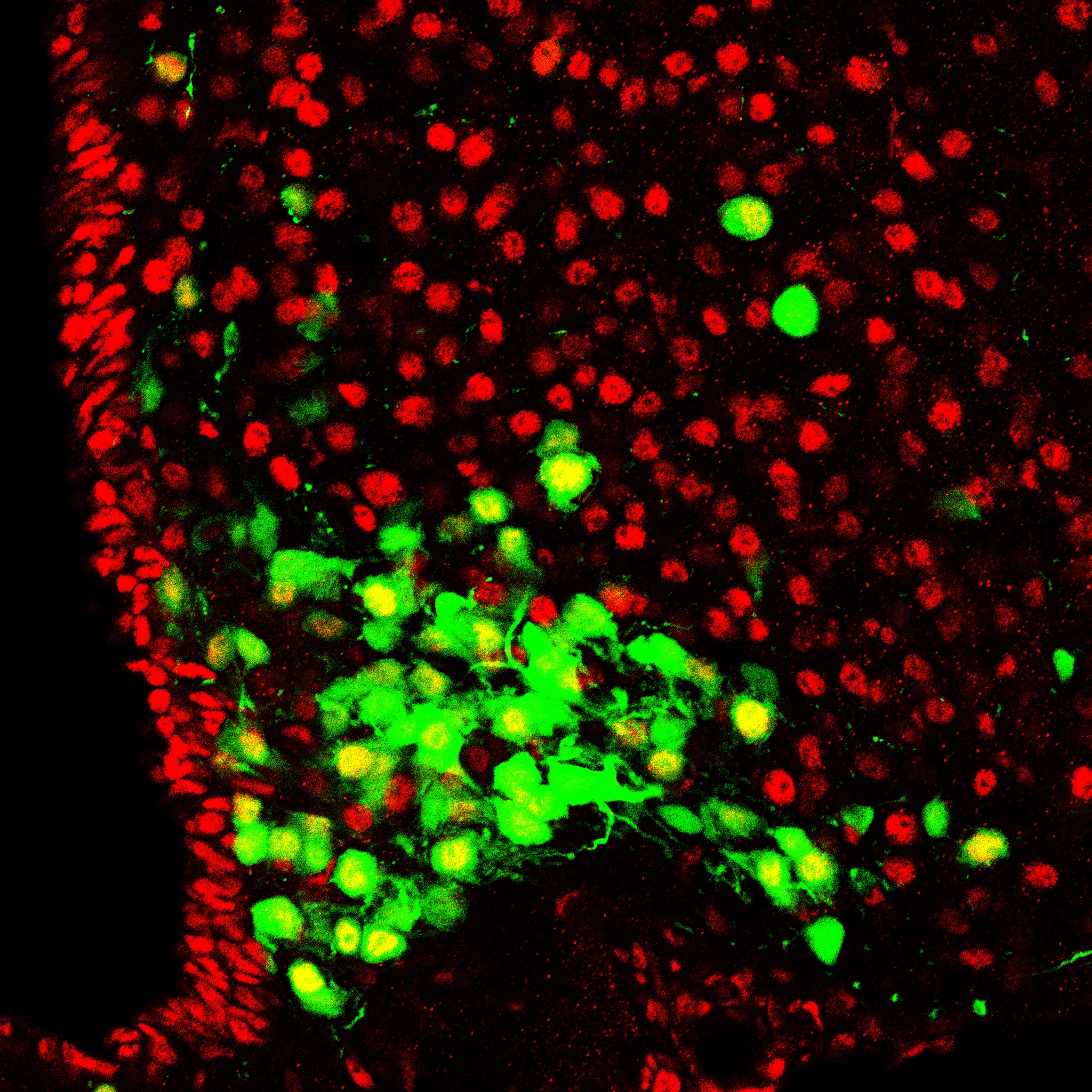

Supplement: Supplementary file 8 — Source Data for Figure 5 [file EMMM-15-e18024-s005.zip › Fig_5H/Fig_5H_EGFP_HEXIM1_shMyh9shActg1.tiff]

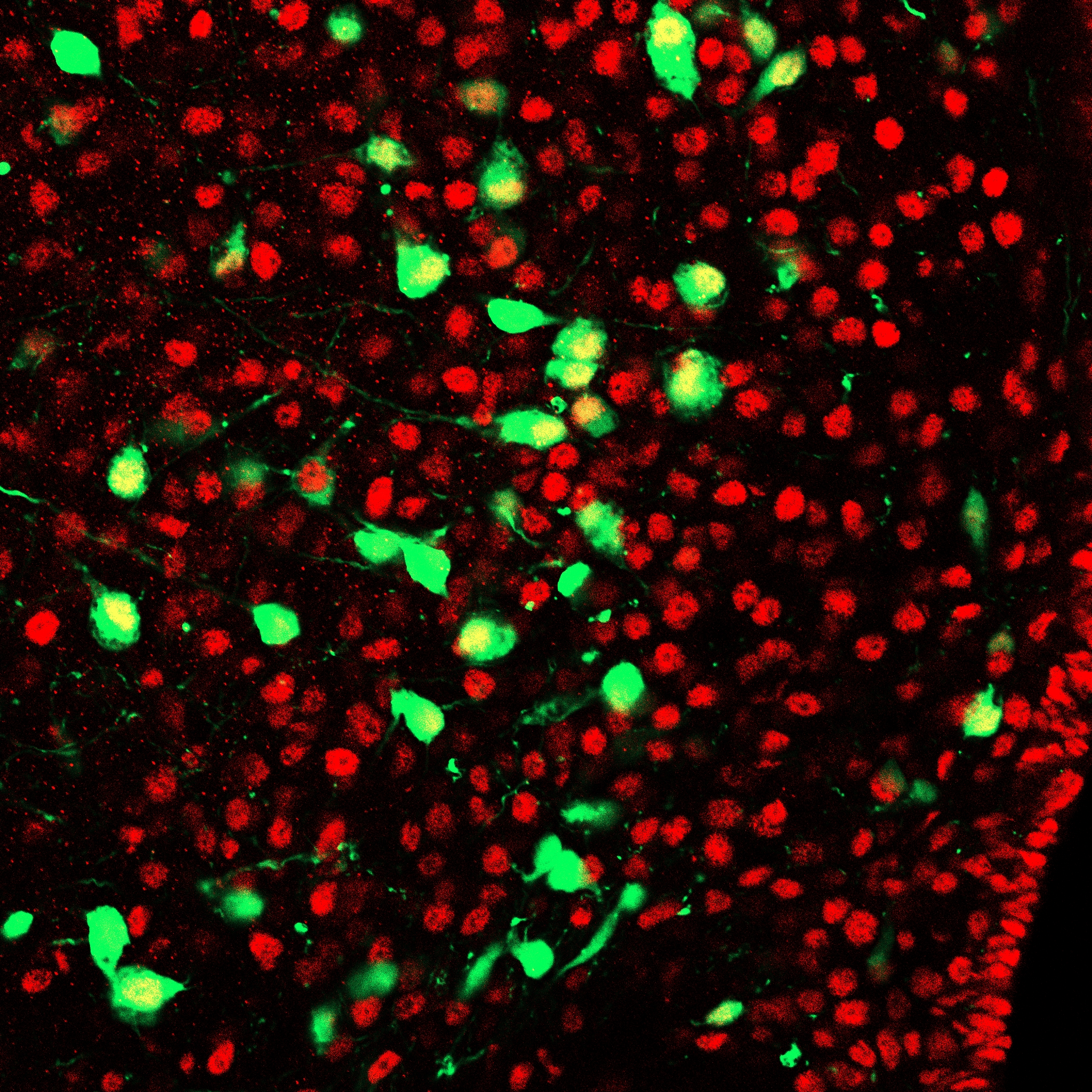

Supplement: Supplementary file 8 — Source Data for Figure 5 [file EMMM-15-e18024-s005.zip › Fig_5I/Fig_5I_EGFP_HEXIM1_shControl.tiff]

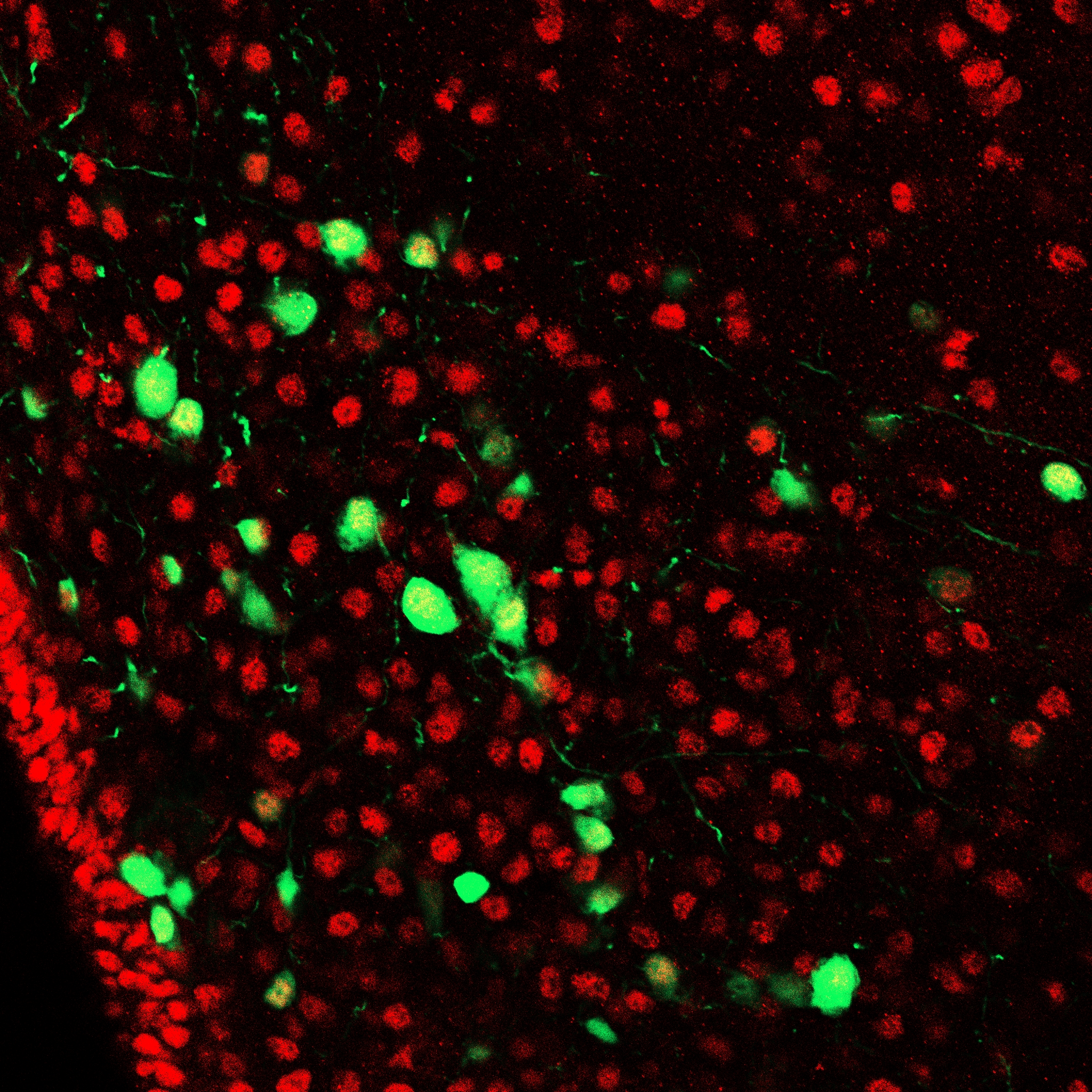

Supplement: Supplementary file 8 — Source Data for Figure 5 [file EMMM-15-e18024-s005.zip › Fig_5I/Fig_5I_EGFP_HEXIM1_shMyh9shActg1.tiff]

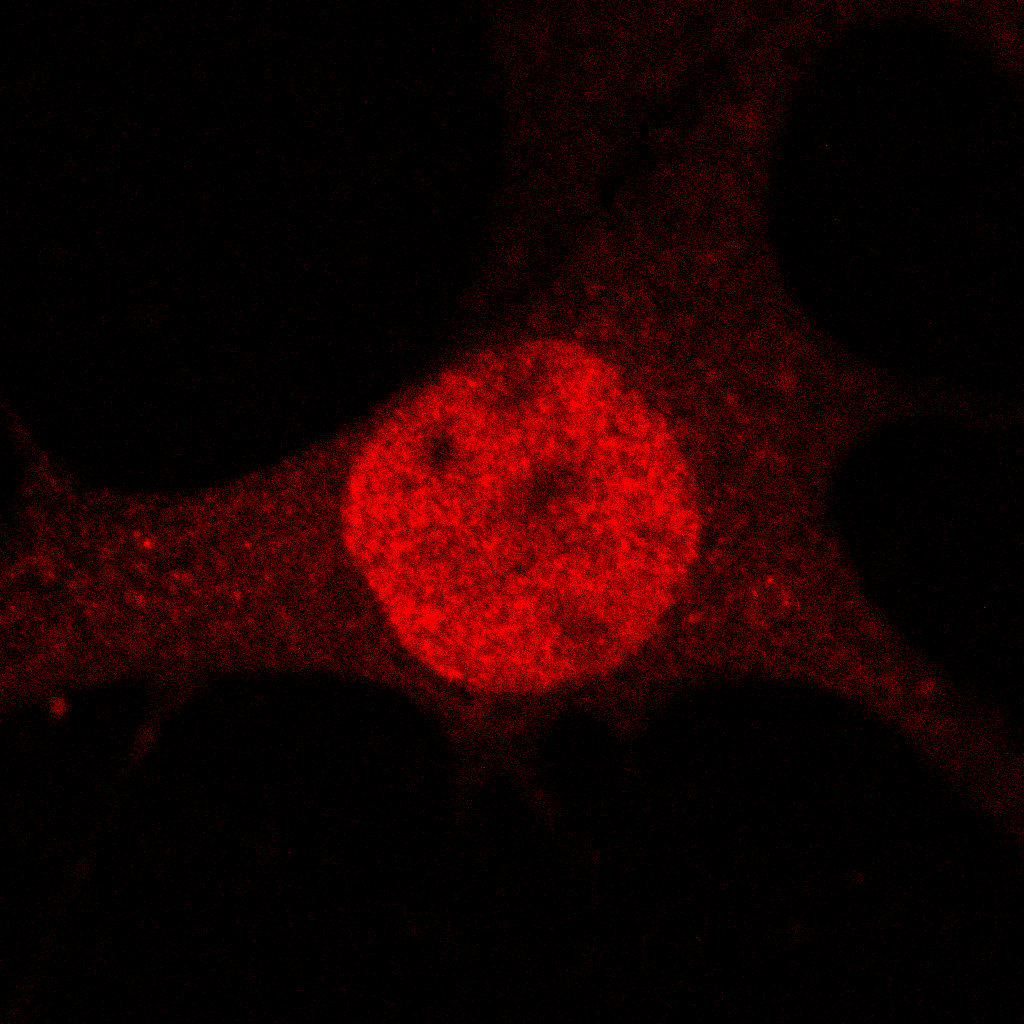

Supplement: Supplementary file 10 — Source Data for Figure 7 [file EMMM-15-e18024-s010.zip › Fig_7A-B/Fig_7A_ICC_image/sgControl+HMBA/HEXIM1.tiff]

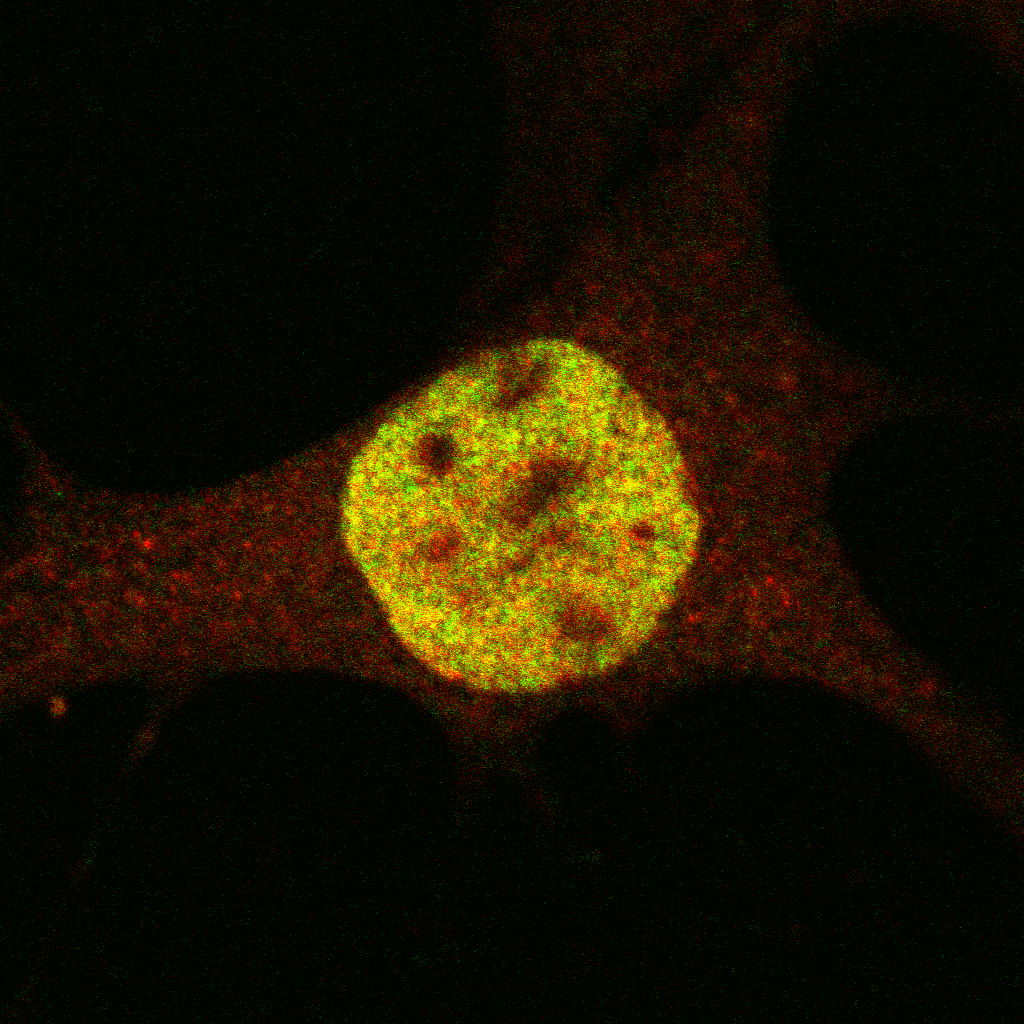

Supplement: Supplementary file 10 — Source Data for Figure 7 [file EMMM-15-e18024-s010.zip › Fig_7A-B/Fig_7A_ICC_image/sgControl+HMBA/HEXIM1_p53.tiff]

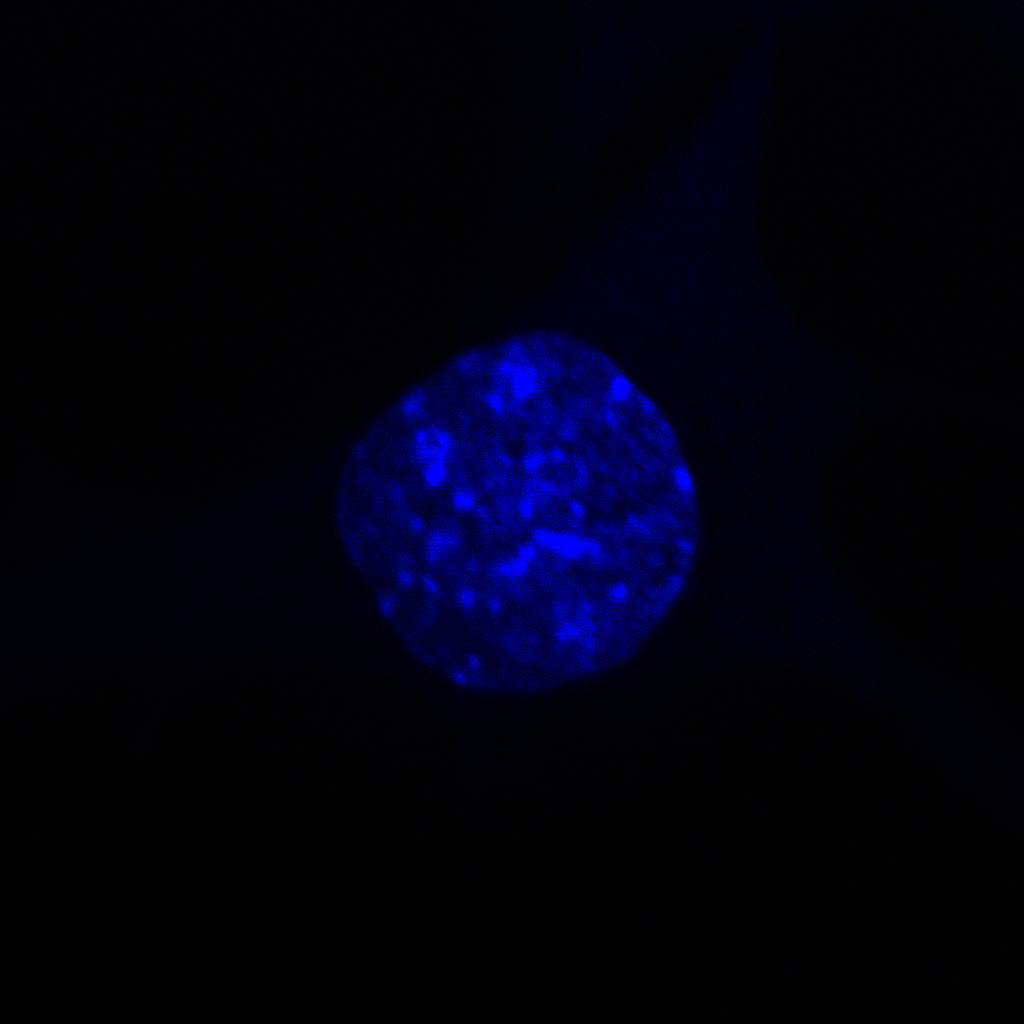

Supplement: Supplementary file 10 — Source Data for Figure 7 [file EMMM-15-e18024-s010.zip › Fig_7A-B/Fig_7A_ICC_image/sgControl+HMBA/Hoechst.tiff]

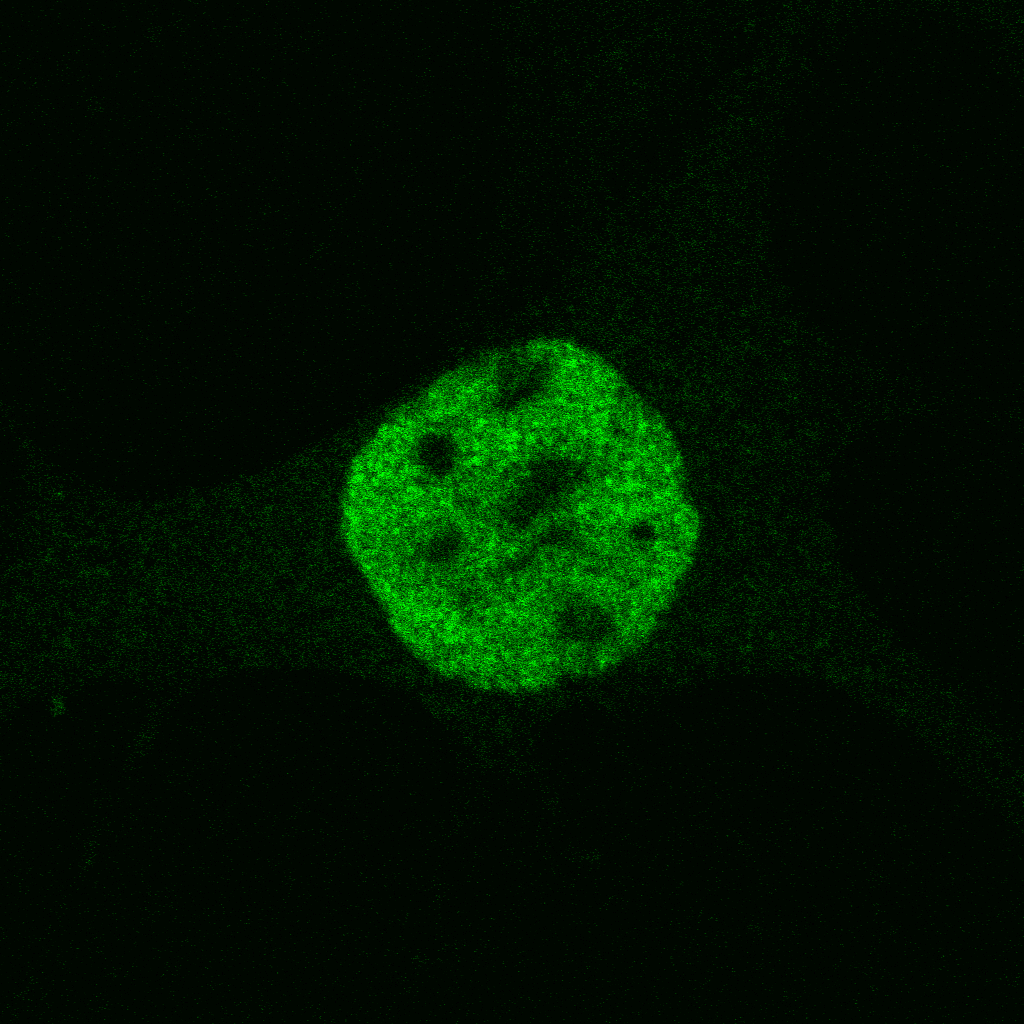

Supplement: Supplementary file 10 — Source Data for Figure 7 [file EMMM-15-e18024-s010.zip › Fig_7A-B/Fig_7A_ICC_image/sgControl+HMBA/p53.tiff]

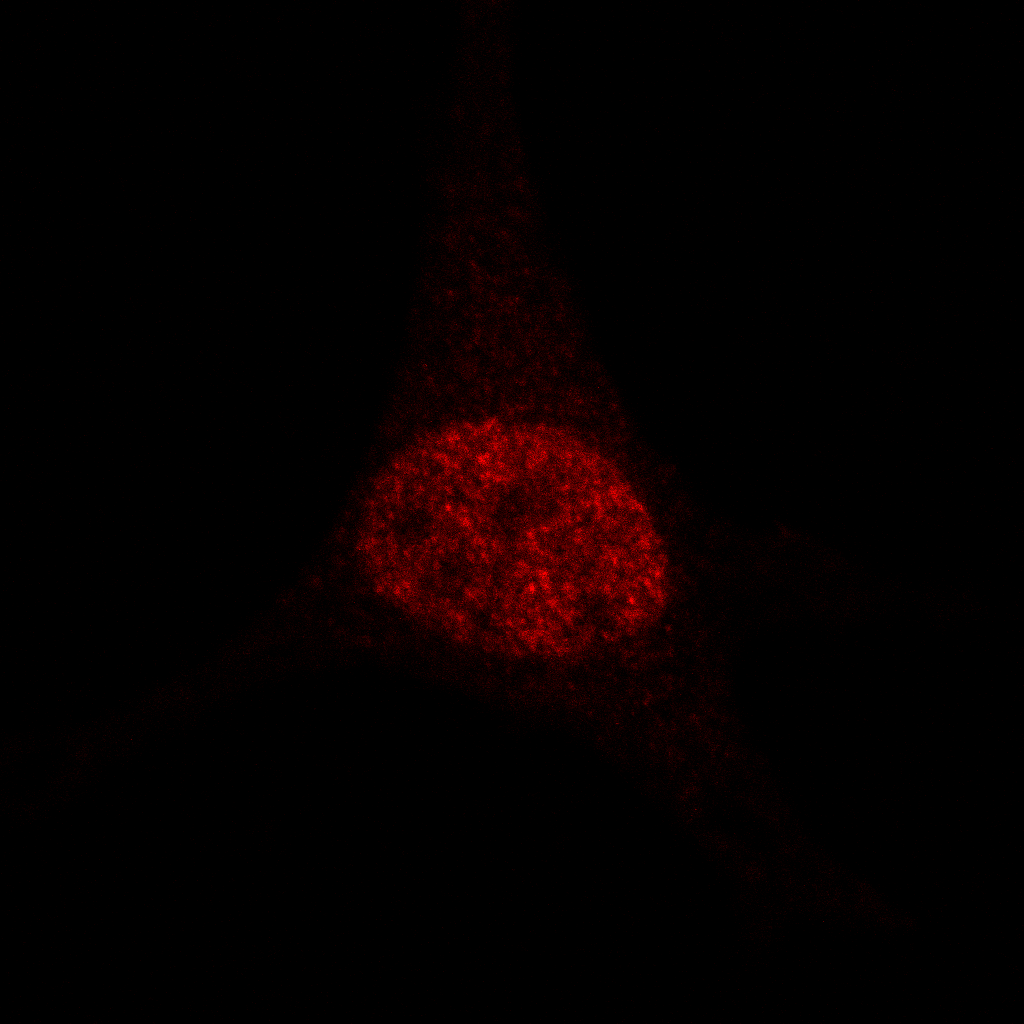

Supplement: Supplementary file 10 — Source Data for Figure 7 [file EMMM-15-e18024-s010.zip › Fig_7A-B/Fig_7A_ICC_image/sgControl+Vehicle/HEXIM1.tiff]

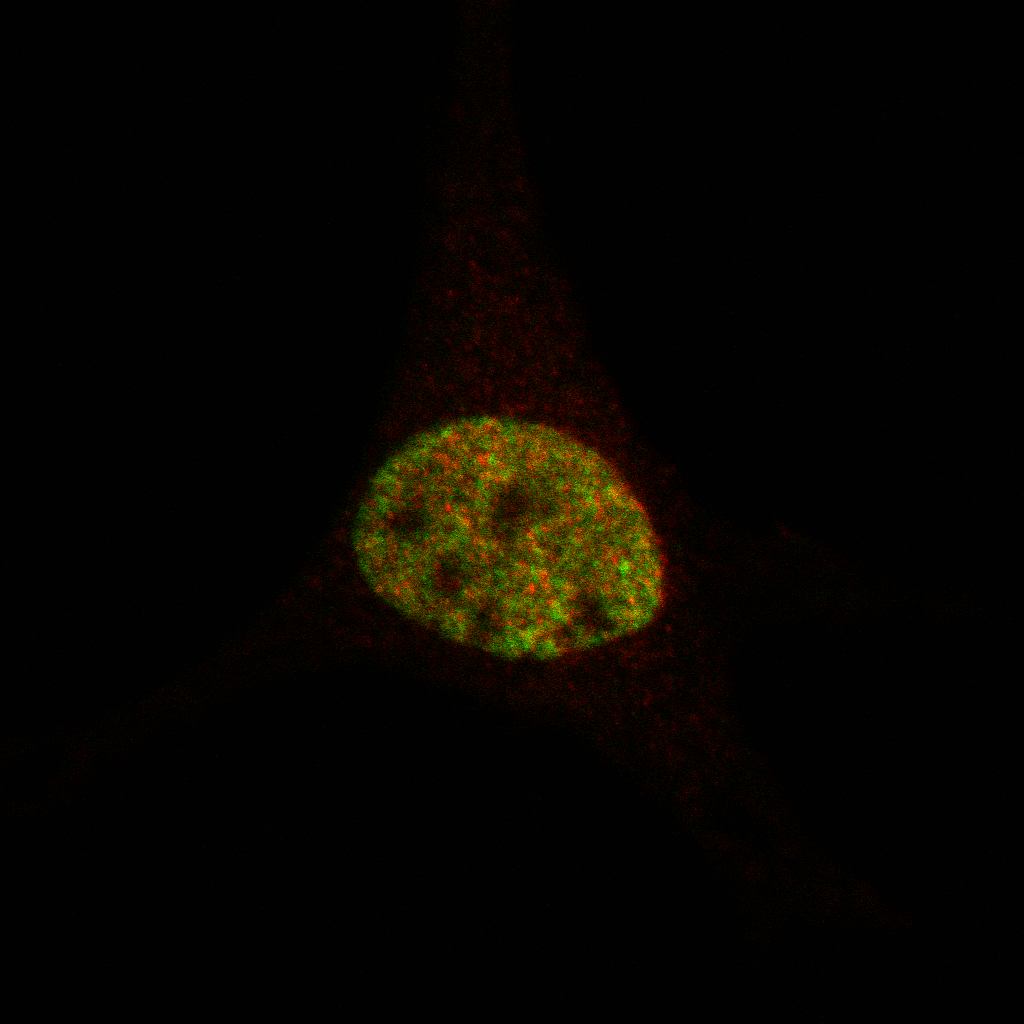

Supplement: Supplementary file 10 — Source Data for Figure 7 [file EMMM-15-e18024-s010.zip › Fig_7A-B/Fig_7A_ICC_image/sgControl+Vehicle/HEXIM1_p53.tiff]

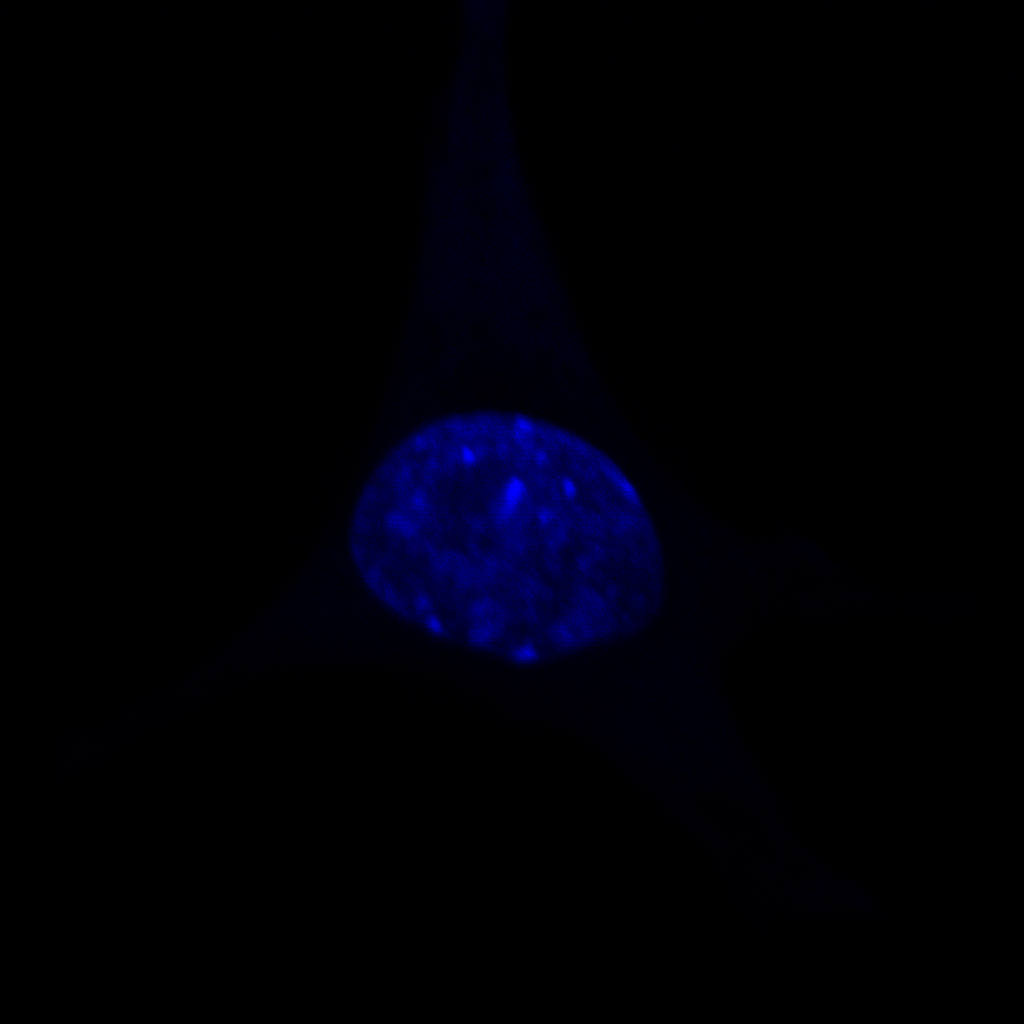

Supplement: Supplementary file 10 — Source Data for Figure 7 [file EMMM-15-e18024-s010.zip › Fig_7A-B/Fig_7A_ICC_image/sgControl+Vehicle/Hoechst.tiff]

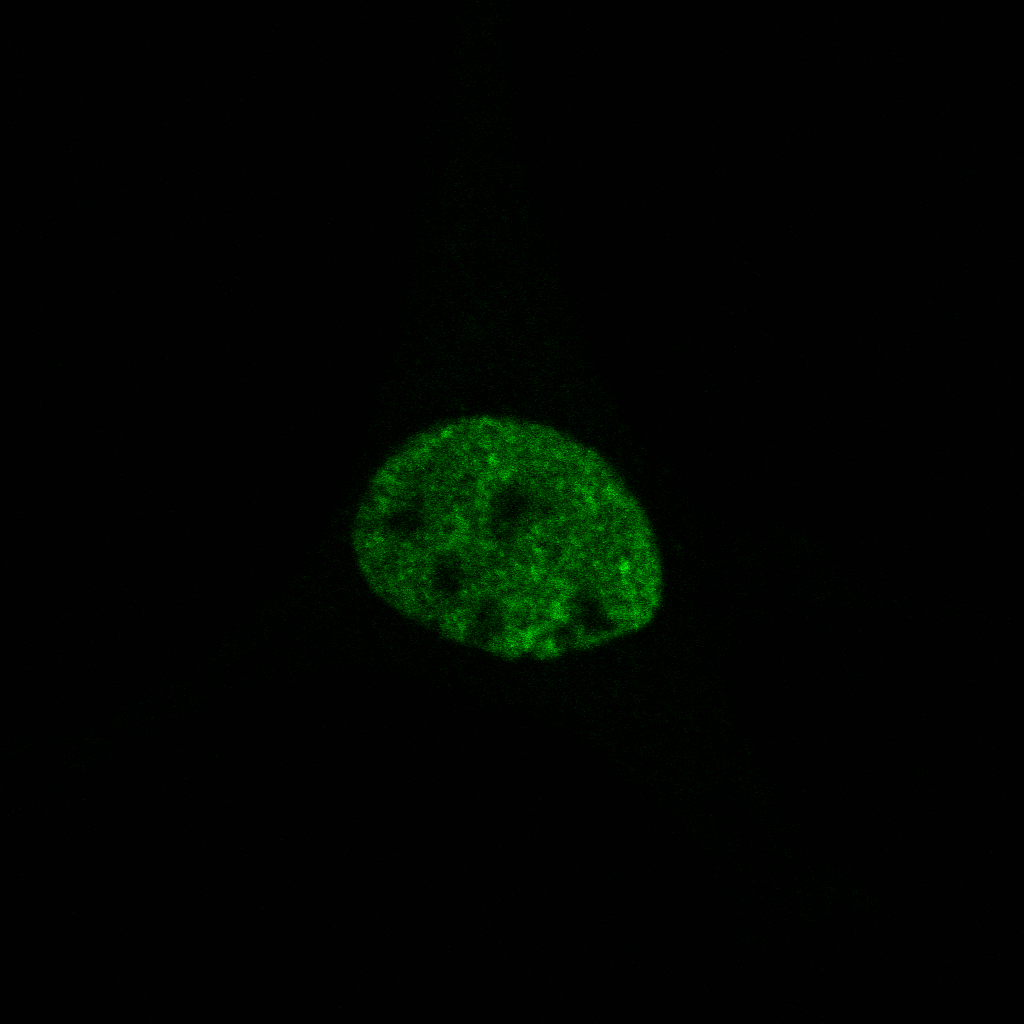

Supplement: Supplementary file 10 — Source Data for Figure 7 [file EMMM-15-e18024-s010.zip › Fig_7A-B/Fig_7A_ICC_image/sgControl+Vehicle/p53.tiff]

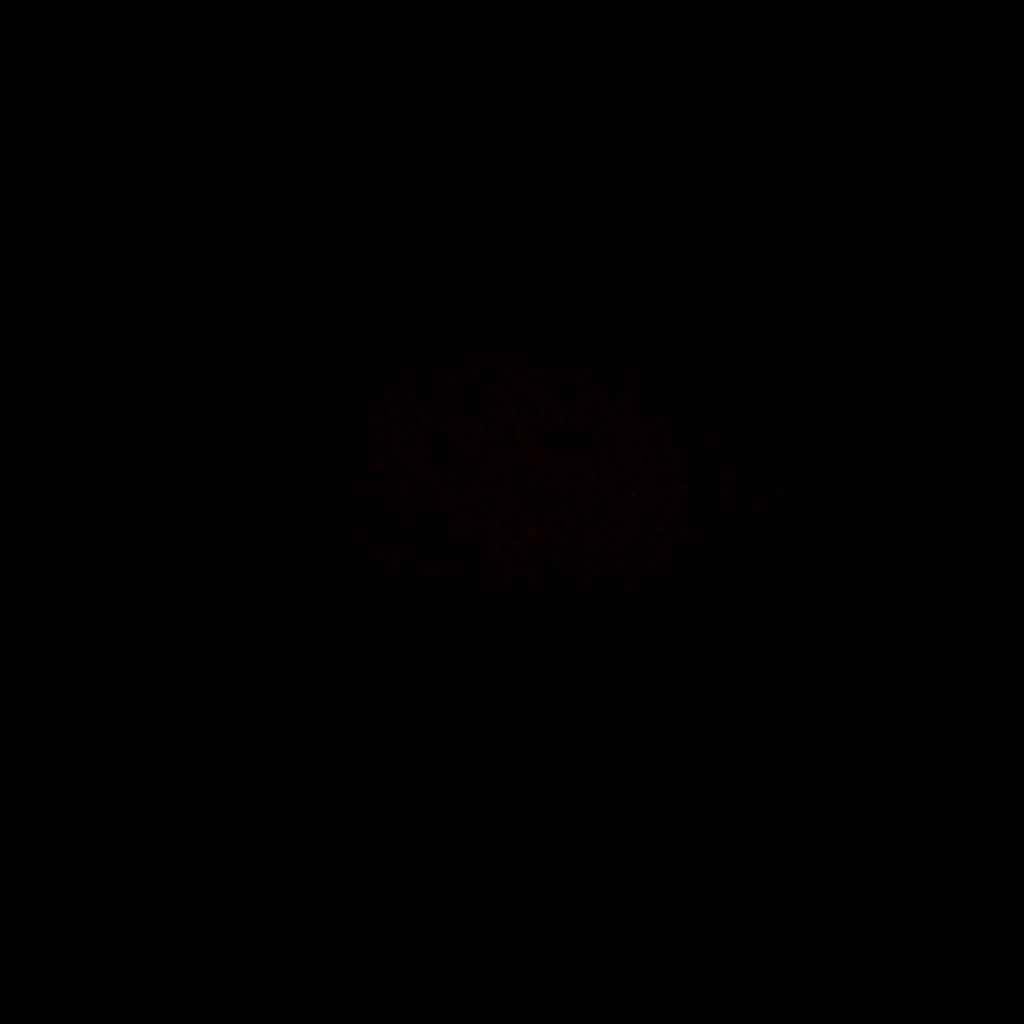

Supplement: Supplementary file 10 — Source Data for Figure 7 [file EMMM-15-e18024-s010.zip › Fig_7A-B/Fig_7A_ICC_image/sgHexim1+HMBA/HEXIM1.tiff]

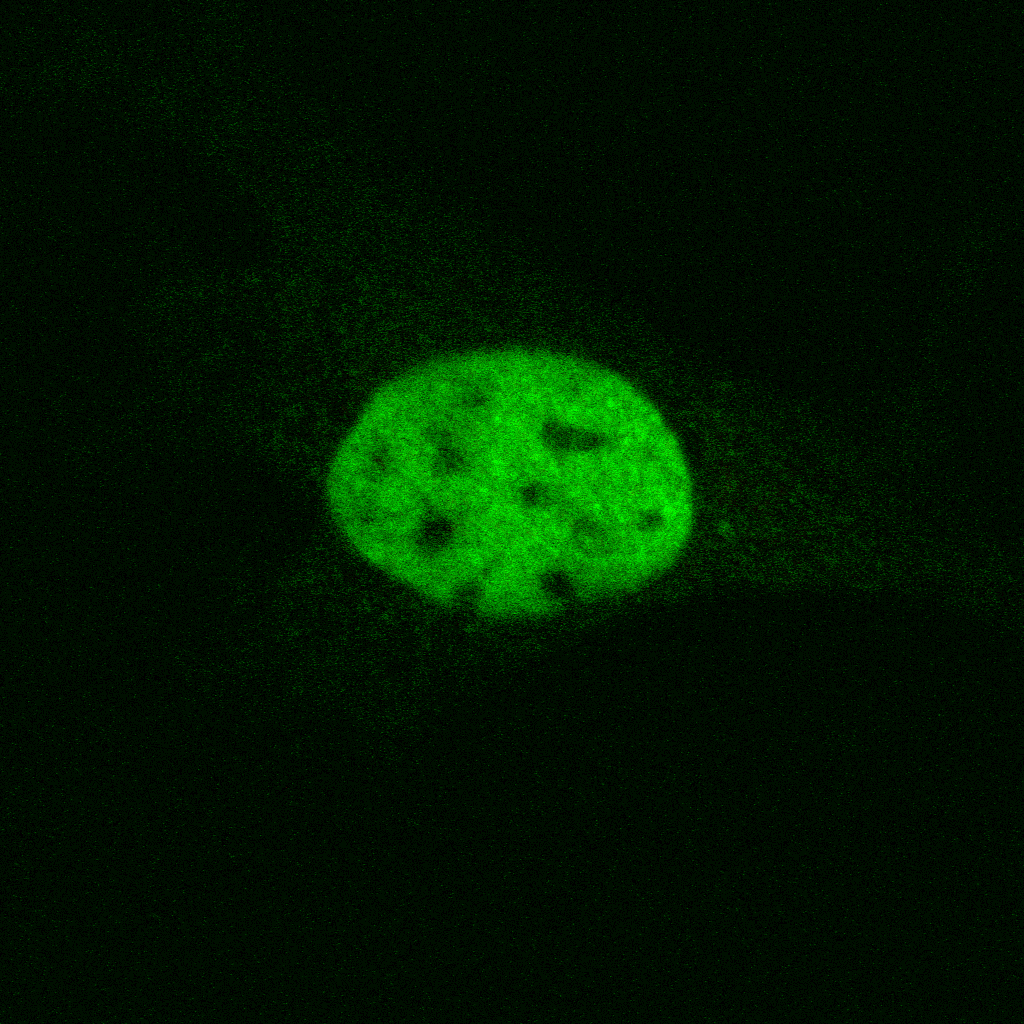

Supplement: Supplementary file 10 — Source Data for Figure 7 [file EMMM-15-e18024-s010.zip › Fig_7A-B/Fig_7A_ICC_image/sgHexim1+HMBA/HEXIM1_p53.tiff]

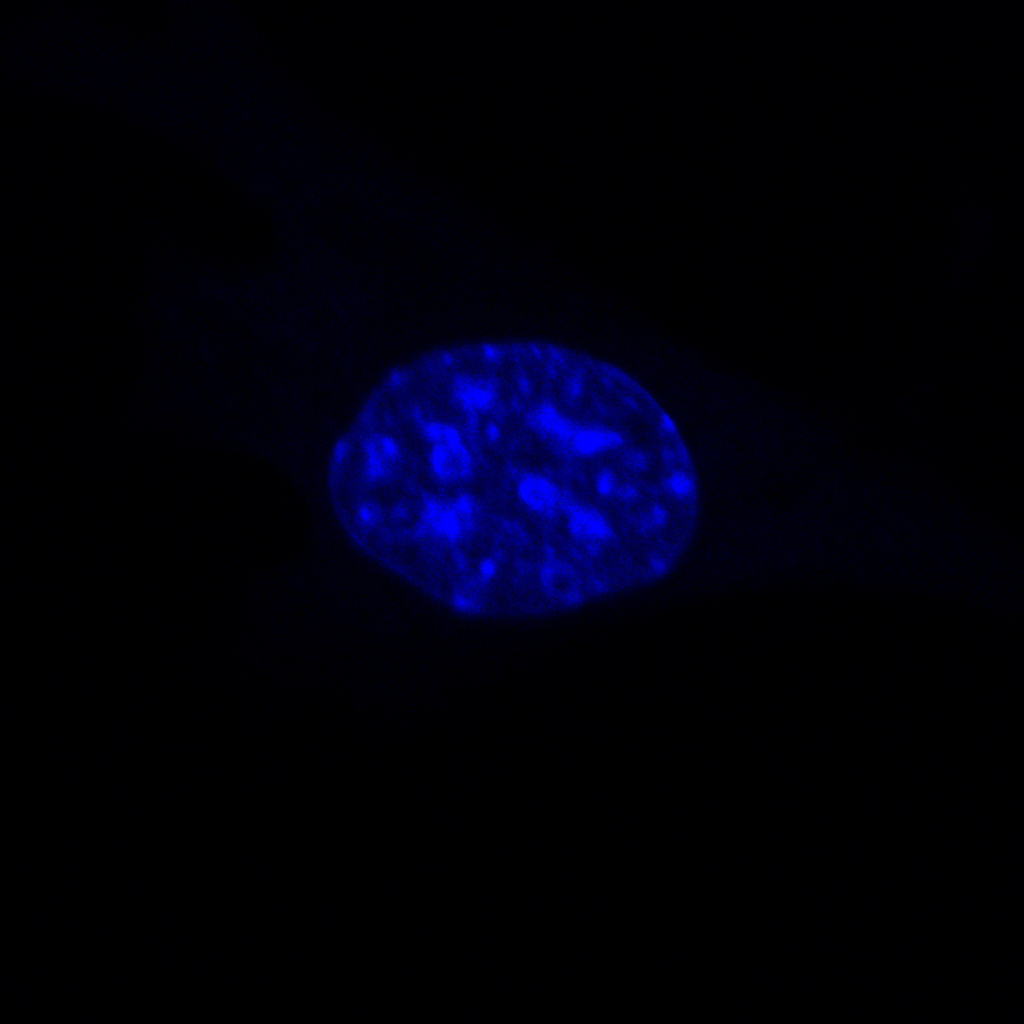

Supplement: Supplementary file 10 — Source Data for Figure 7 [file EMMM-15-e18024-s010.zip › Fig_7A-B/Fig_7A_ICC_image/sgHexim1+HMBA/Hoechst.tiff]

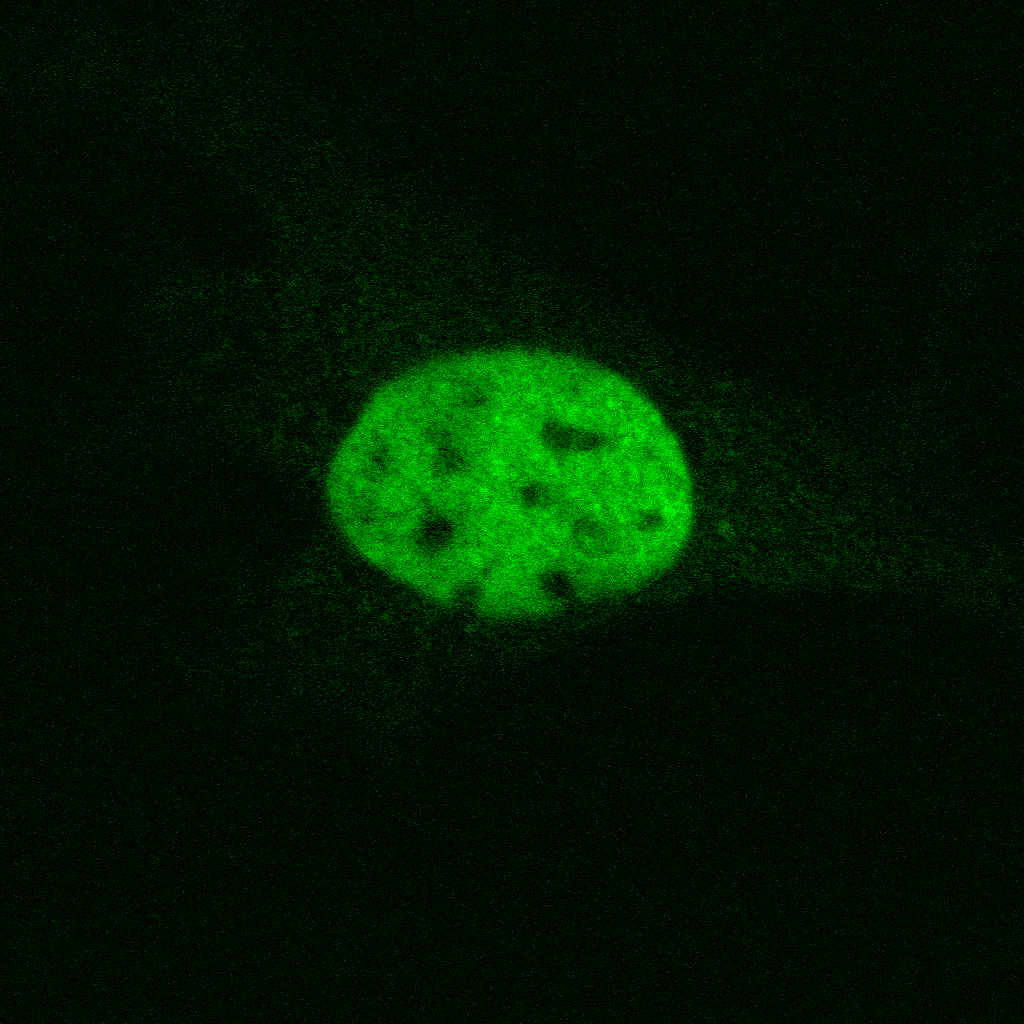

Supplement: Supplementary file 10 — Source Data for Figure 7 [file EMMM-15-e18024-s010.zip › Fig_7A-B/Fig_7A_ICC_image/sgHexim1+HMBA/p53.tiff]

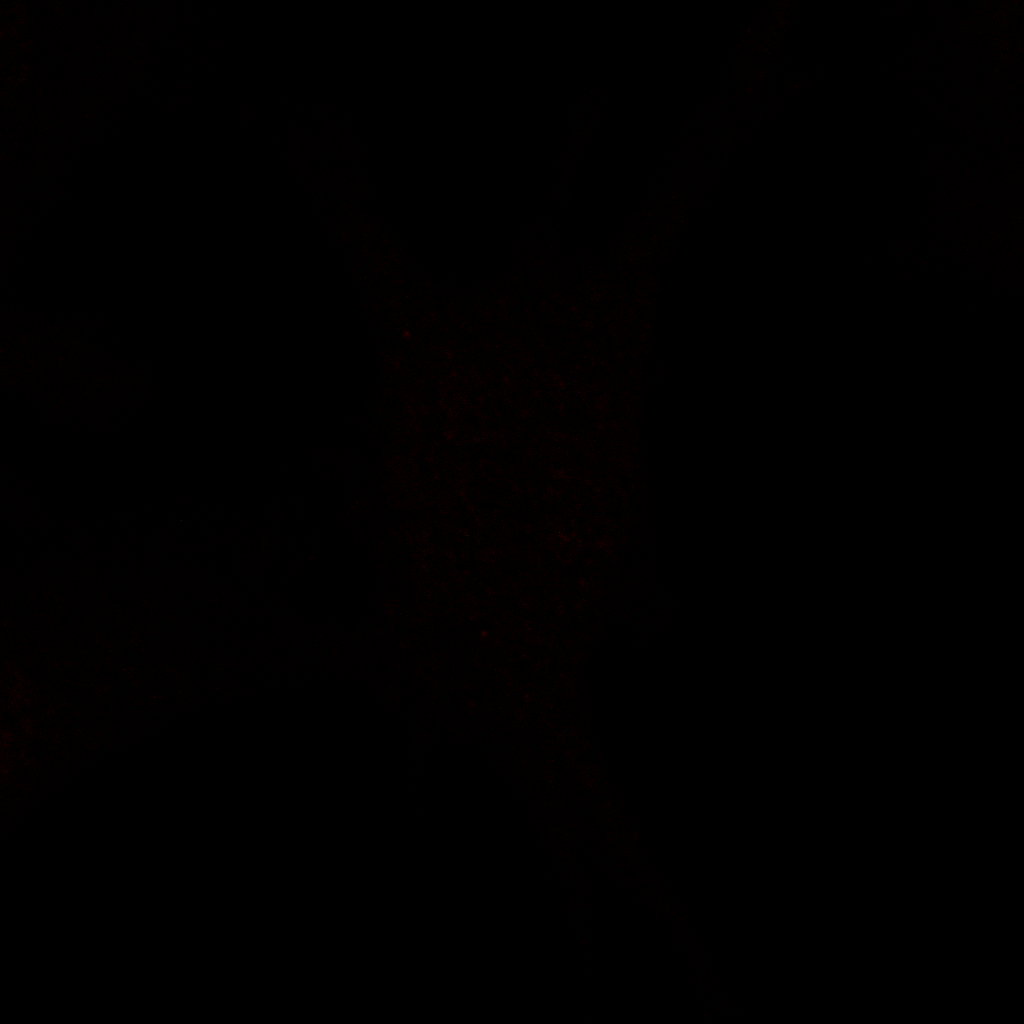

Supplement: Supplementary file 10 — Source Data for Figure 7 [file EMMM-15-e18024-s010.zip › Fig_7A-B/Fig_7A_ICC_image/sgHexim1+Vehicle/HEXIM1.tiff]

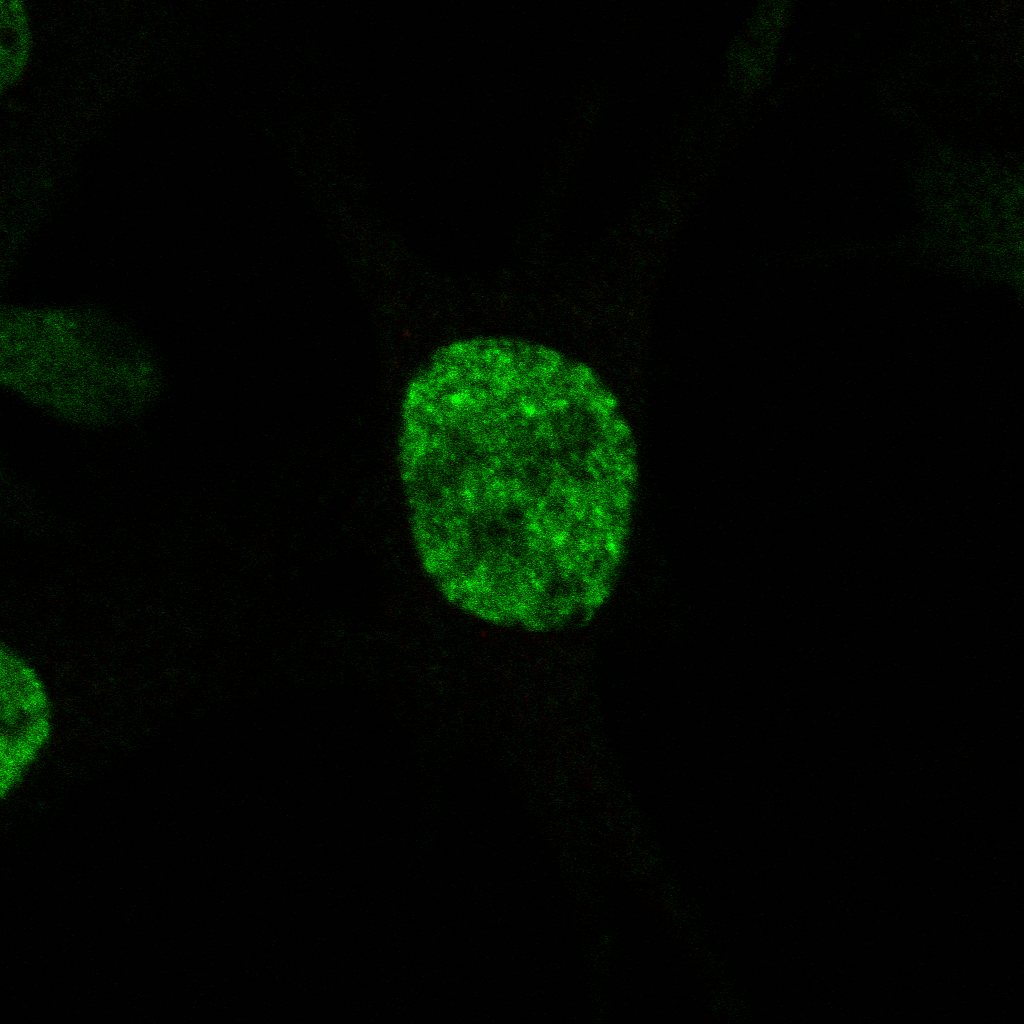

Supplement: Supplementary file 10 — Source Data for Figure 7 [file EMMM-15-e18024-s010.zip › Fig_7A-B/Fig_7A_ICC_image/sgHexim1+Vehicle/HEXIM1_p53.tiff]

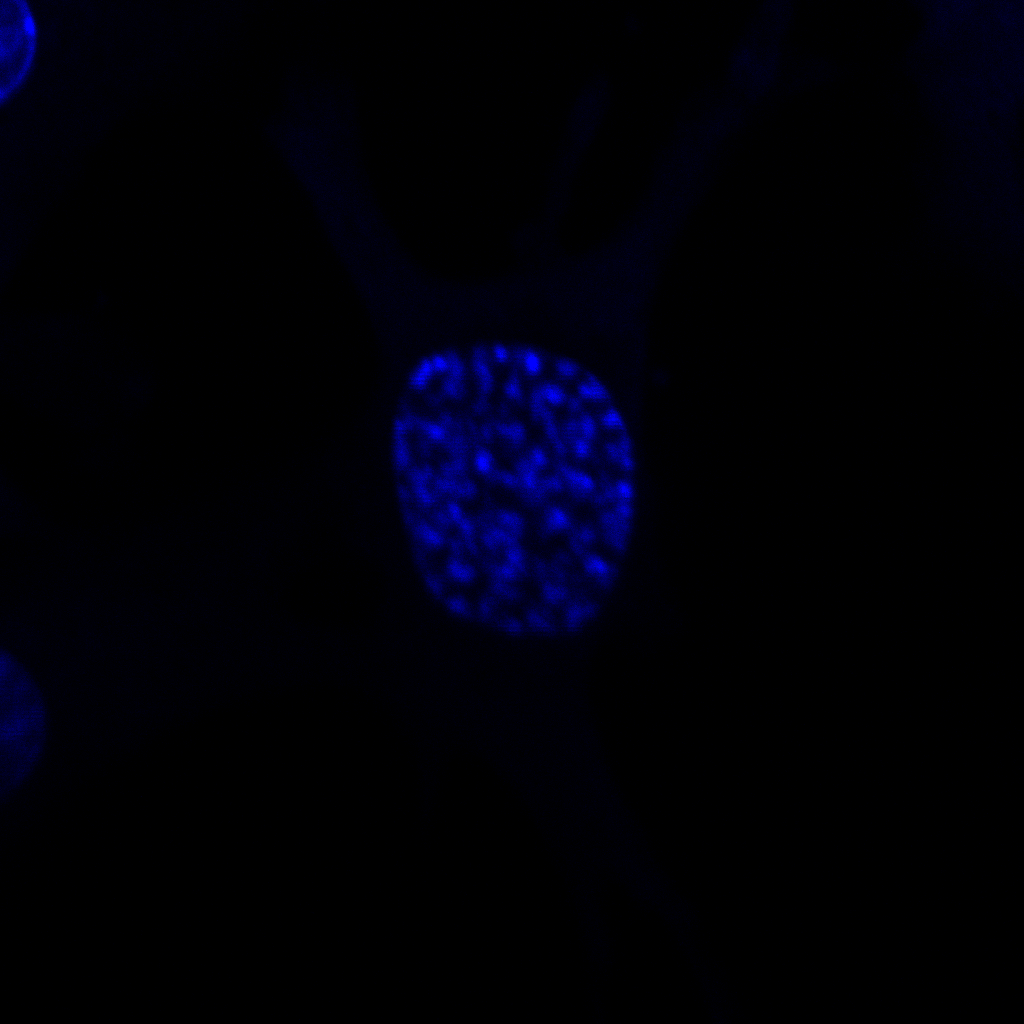

Supplement: Supplementary file 10 — Source Data for Figure 7 [file EMMM-15-e18024-s010.zip › Fig_7A-B/Fig_7A_ICC_image/sgHexim1+Vehicle/Hoechst.tiff]

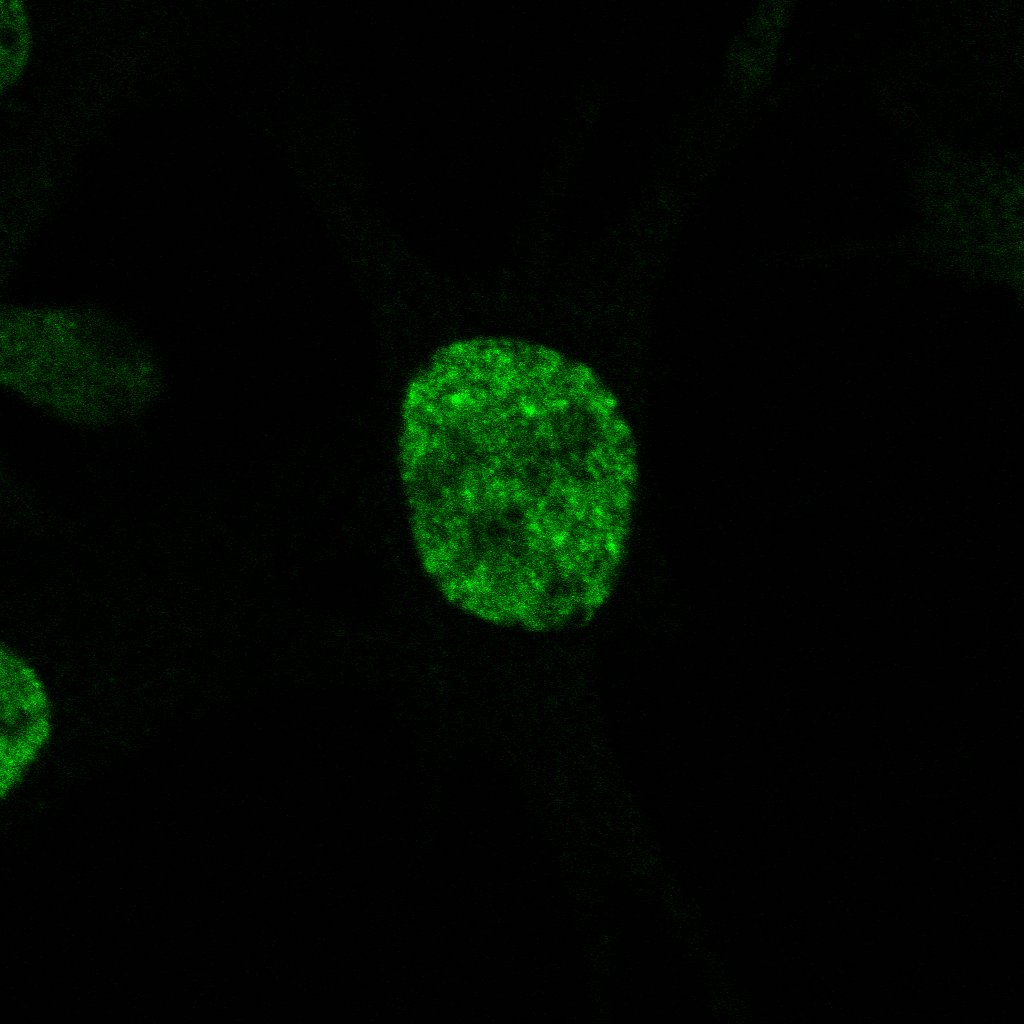

Supplement: Supplementary file 10 — Source Data for Figure 7 [file EMMM-15-e18024-s010.zip › Fig_7A-B/Fig_7A_ICC_image/sgHexim1+Vehicle/p53.tiff]

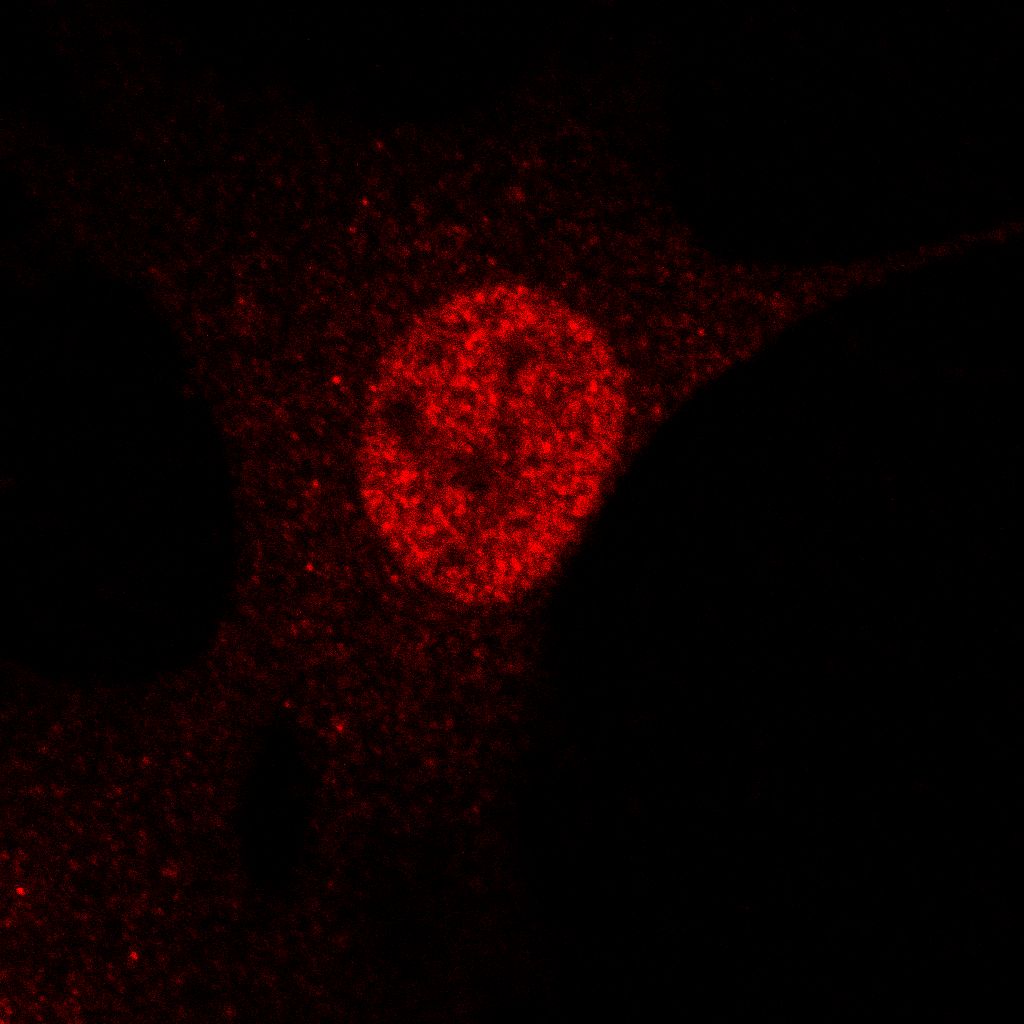

Supplement: Supplementary file 10 — Source Data for Figure 7 [file EMMM-15-e18024-s010.zip › Fig_7A-B/Fig_7B_ICC_image/sgControl+HMBA/HEXIM1.tiff]

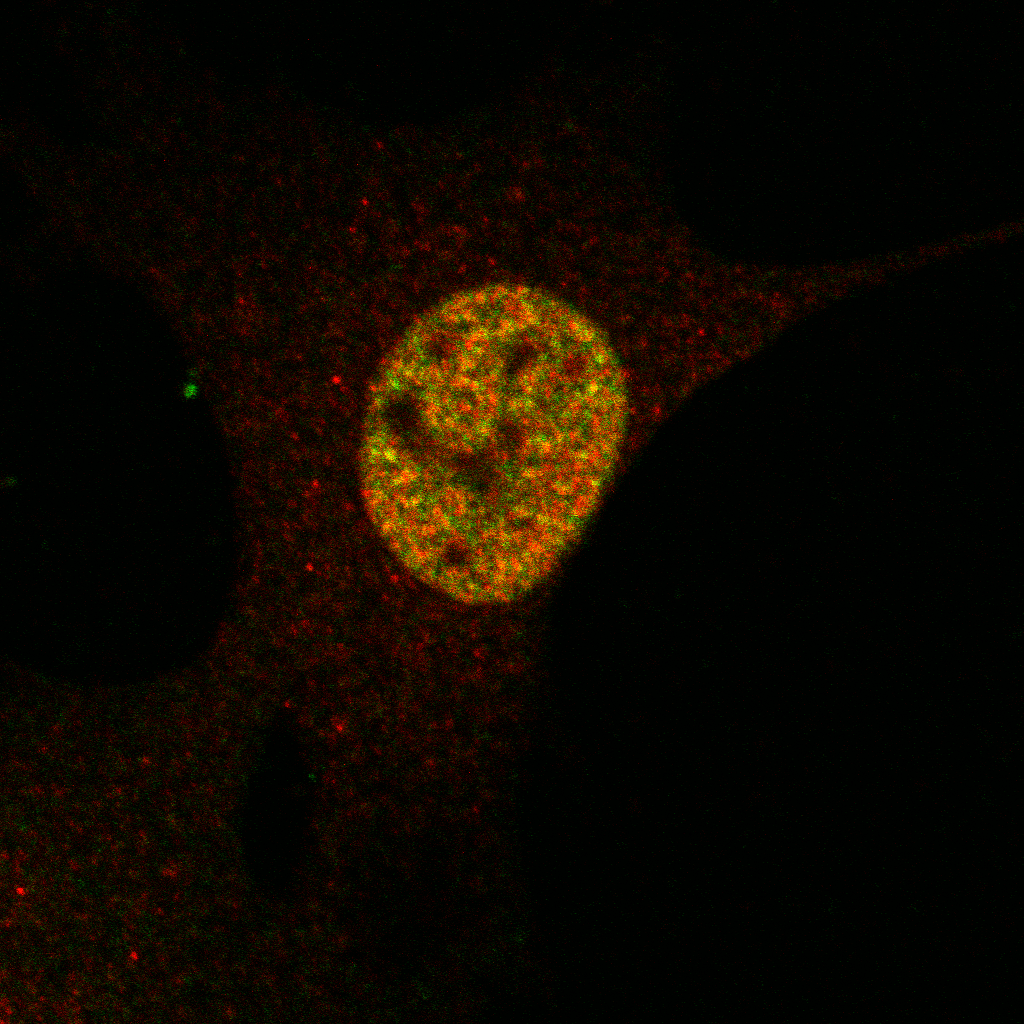

Supplement: Supplementary file 10 — Source Data for Figure 7 [file EMMM-15-e18024-s010.zip › Fig_7A-B/Fig_7B_ICC_image/sgControl+HMBA/HEXIM1_p53.tiff]

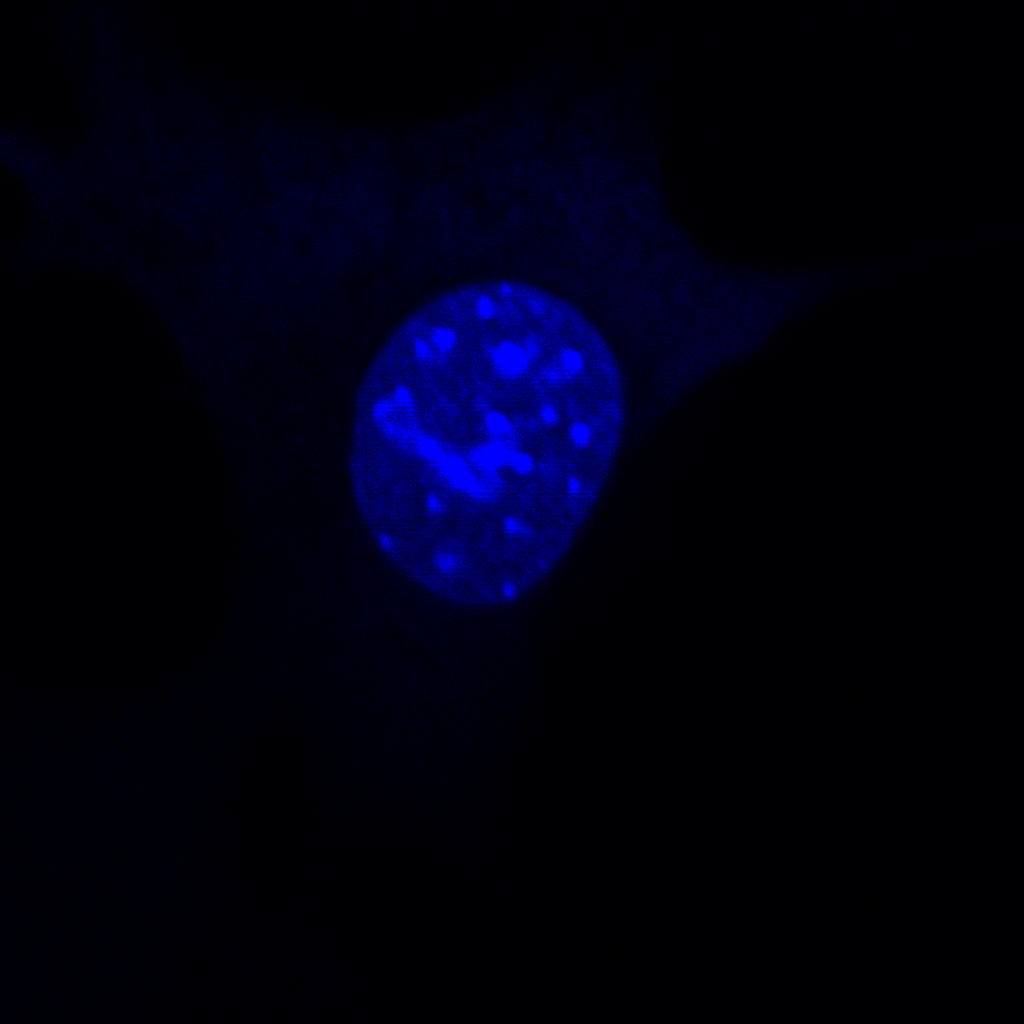

Supplement: Supplementary file 10 — Source Data for Figure 7 [file EMMM-15-e18024-s010.zip › Fig_7A-B/Fig_7B_ICC_image/sgControl+HMBA/Hoechst.tiff]

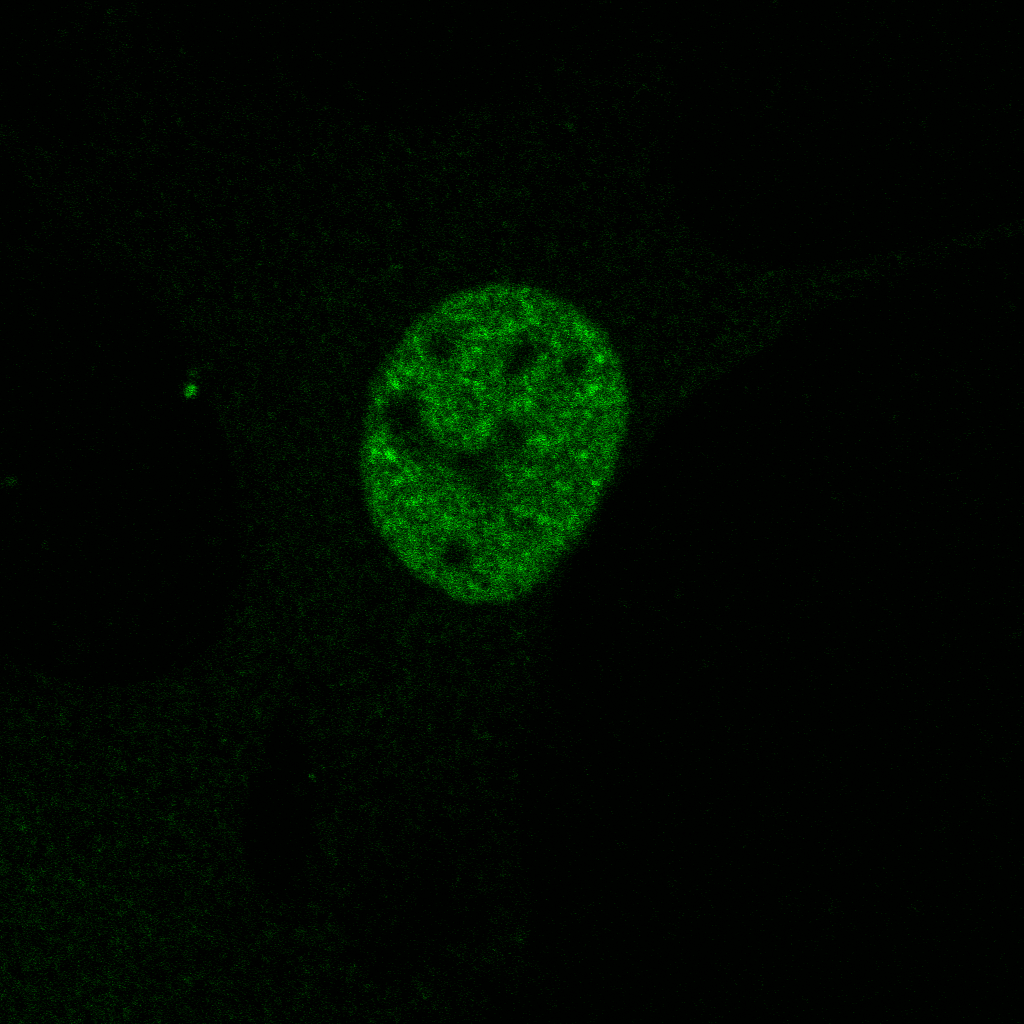

Supplement: Supplementary file 10 — Source Data for Figure 7 [file EMMM-15-e18024-s010.zip › Fig_7A-B/Fig_7B_ICC_image/sgControl+HMBA/p53.tiff]

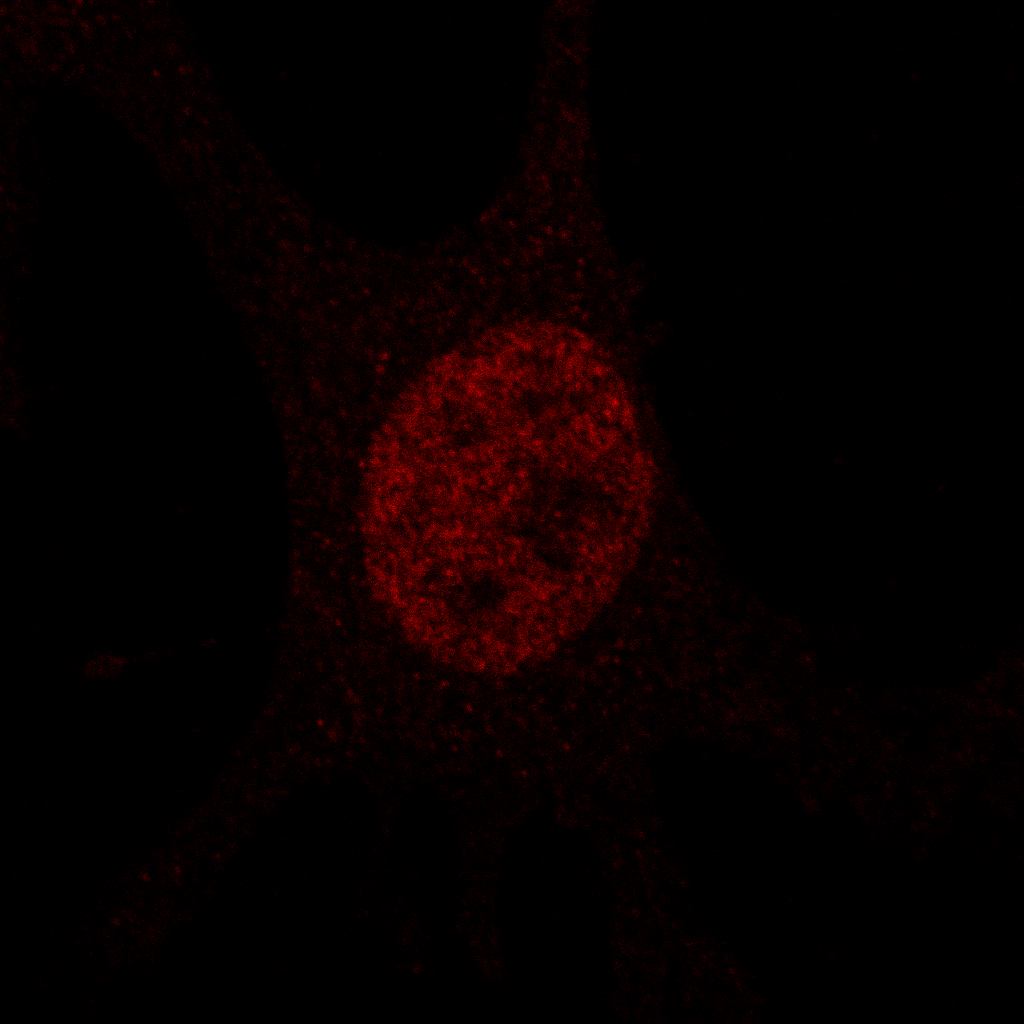

Supplement: Supplementary file 10 — Source Data for Figure 7 [file EMMM-15-e18024-s010.zip › Fig_7A-B/Fig_7B_ICC_image/sgControl+Vehicle/HEXIM1.tiff]

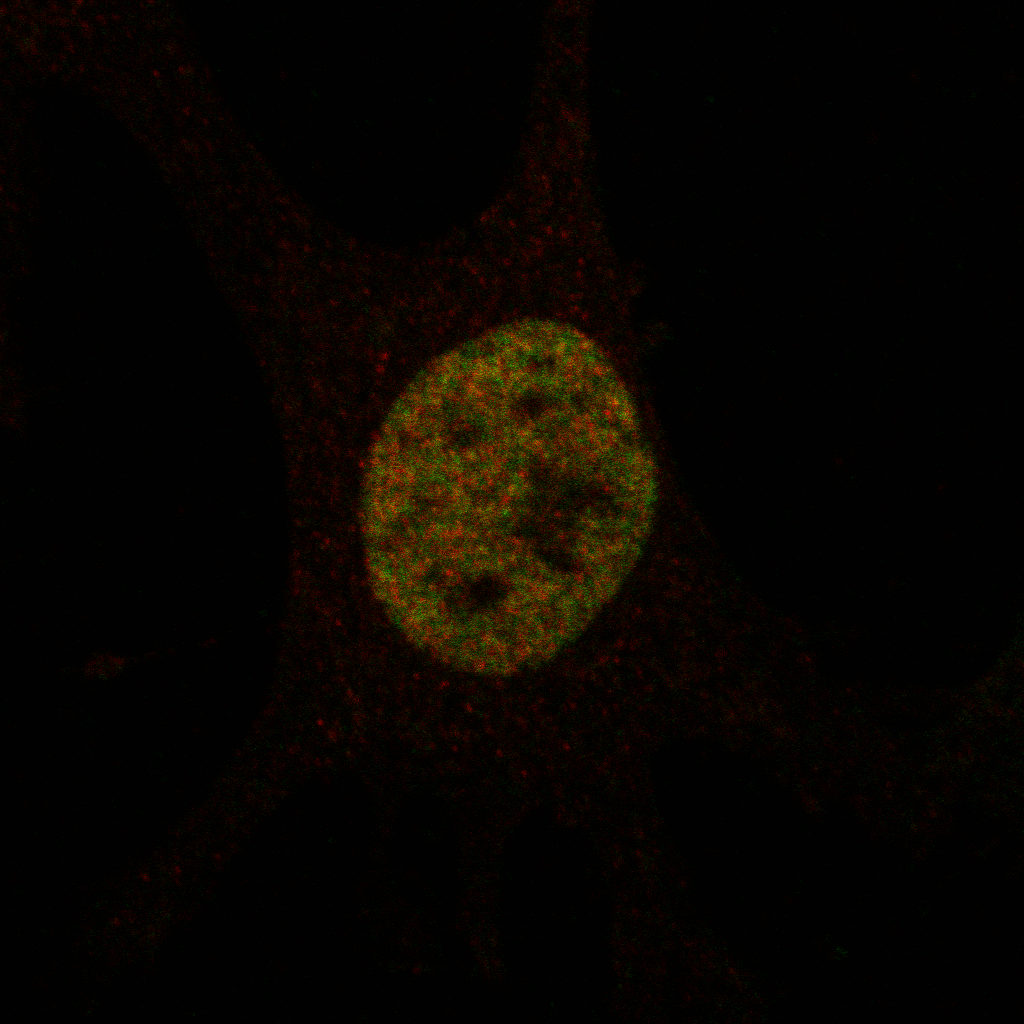

Supplement: Supplementary file 10 — Source Data for Figure 7 [file EMMM-15-e18024-s010.zip › Fig_7A-B/Fig_7B_ICC_image/sgControl+Vehicle/HEXIM1_p53.tiff]

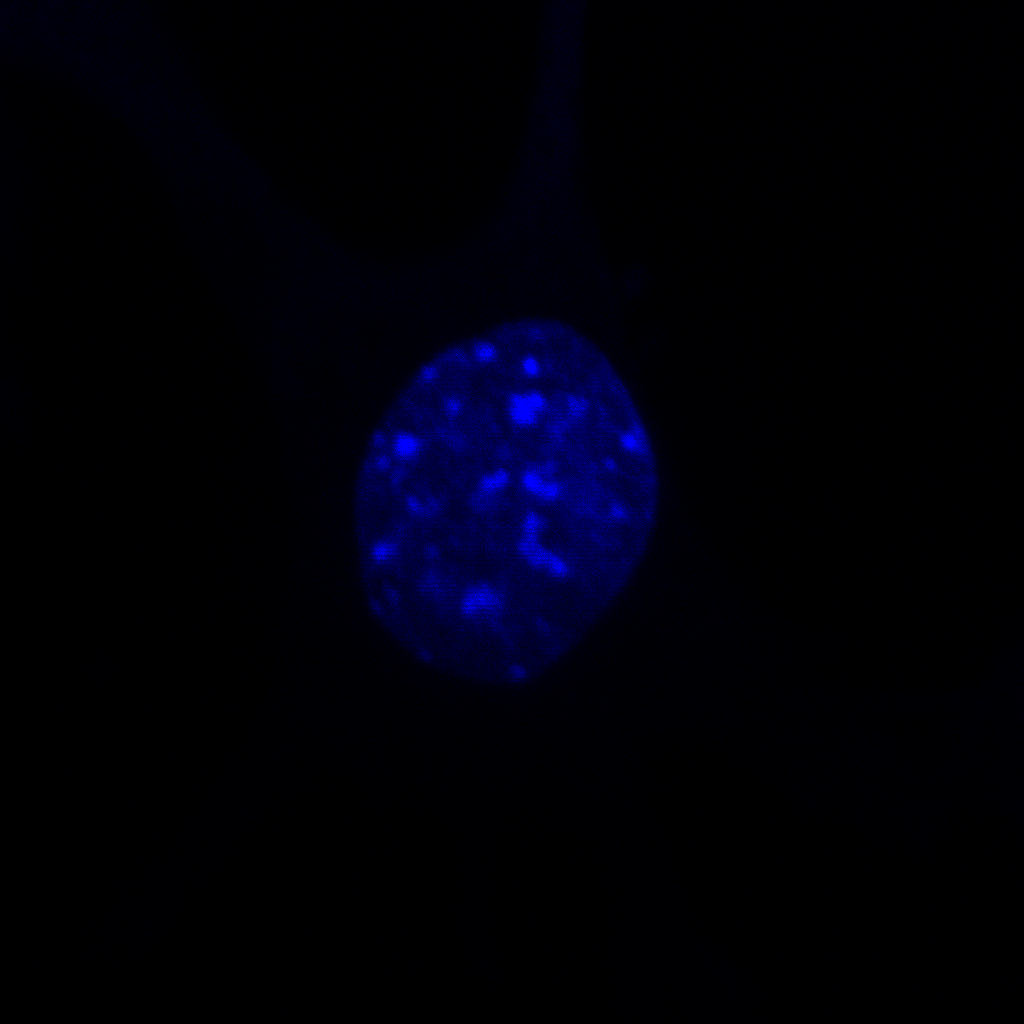

Supplement: Supplementary file 10 — Source Data for Figure 7 [file EMMM-15-e18024-s010.zip › Fig_7A-B/Fig_7B_ICC_image/sgControl+Vehicle/Hoechst.tiff]

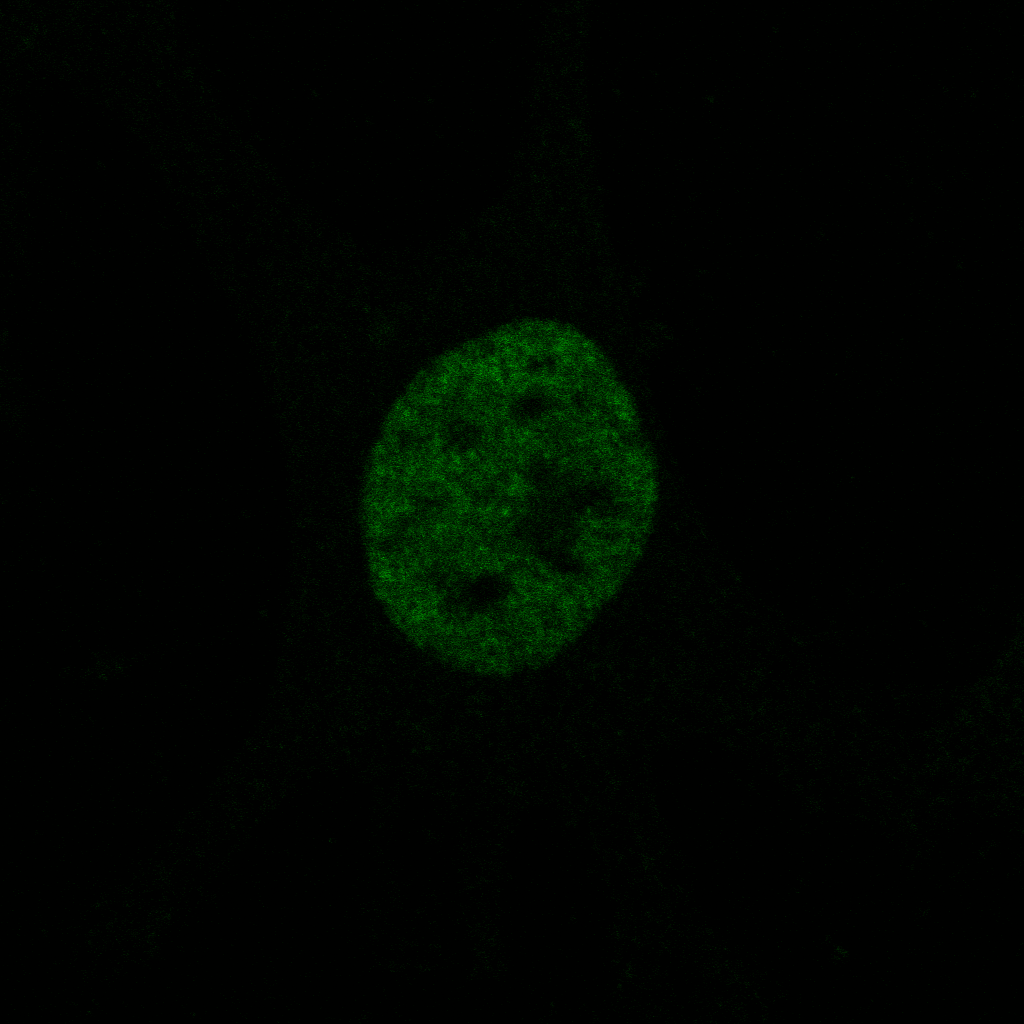

Supplement: Supplementary file 10 — Source Data for Figure 7 [file EMMM-15-e18024-s010.zip › Fig_7A-B/Fig_7B_ICC_image/sgControl+Vehicle/p53.tiff]

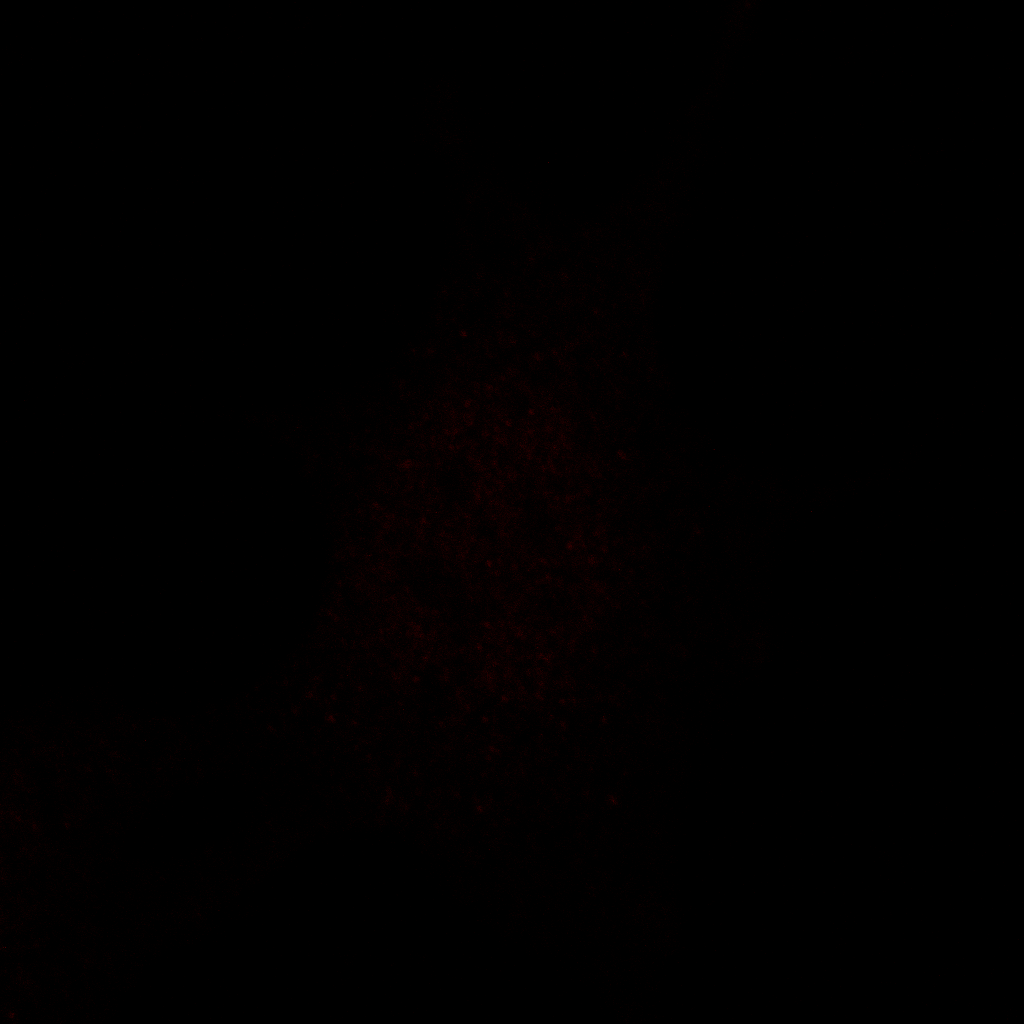

Supplement: Supplementary file 10 — Source Data for Figure 7 [file EMMM-15-e18024-s010.zip › Fig_7A-B/Fig_7B_ICC_image/sgHexim1+HMBA/HEXIM1.tiff]

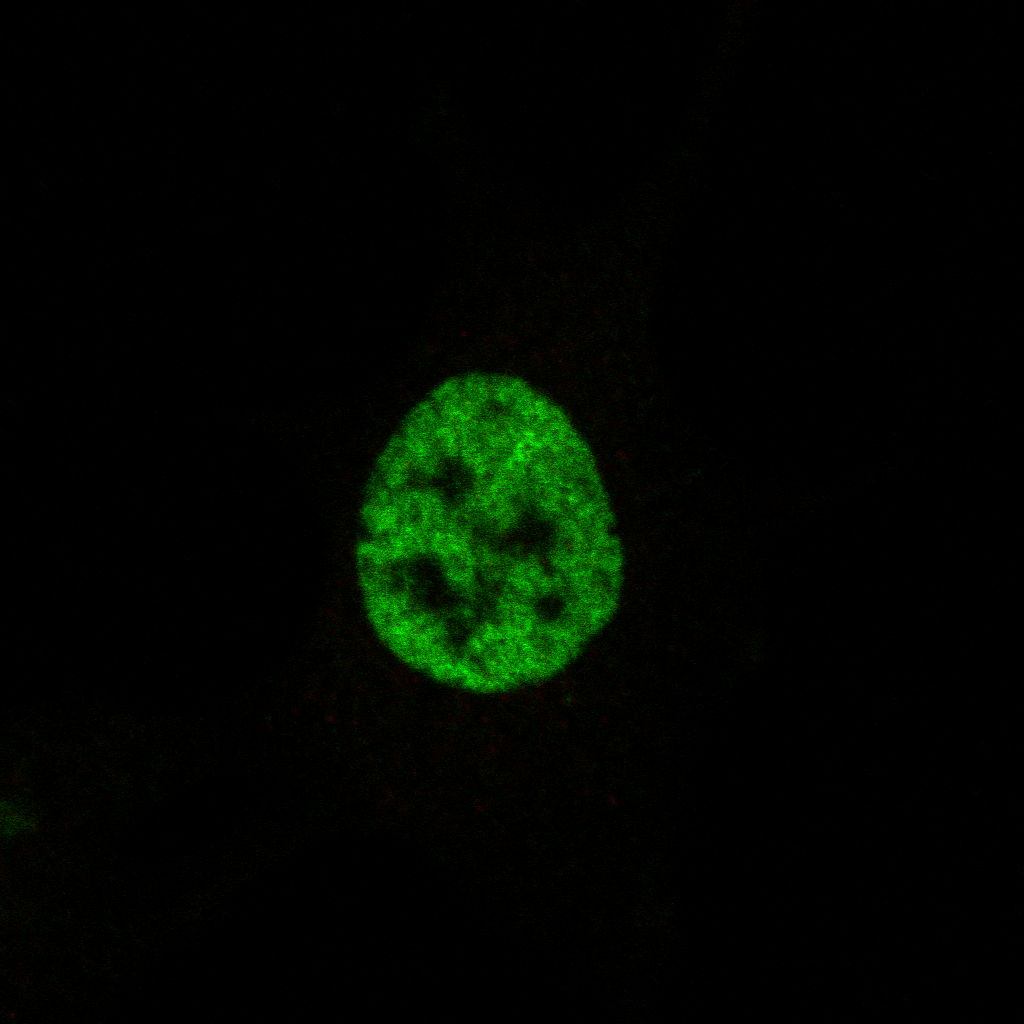

Supplement: Supplementary file 10 — Source Data for Figure 7 [file EMMM-15-e18024-s010.zip › Fig_7A-B/Fig_7B_ICC_image/sgHexim1+HMBA/HEXIM1_p53.tiff]

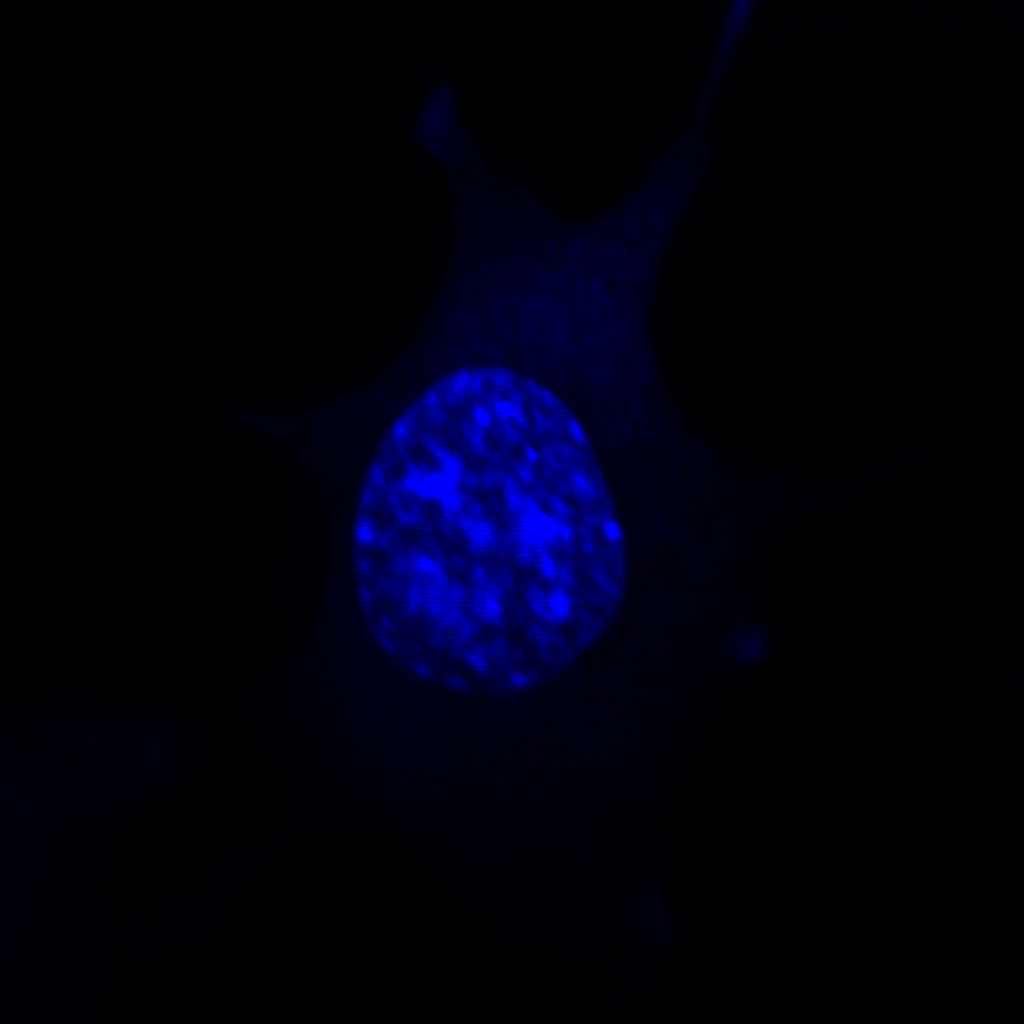

Supplement: Supplementary file 10 — Source Data for Figure 7 [file EMMM-15-e18024-s010.zip › Fig_7A-B/Fig_7B_ICC_image/sgHexim1+HMBA/Hoechst.tiff]

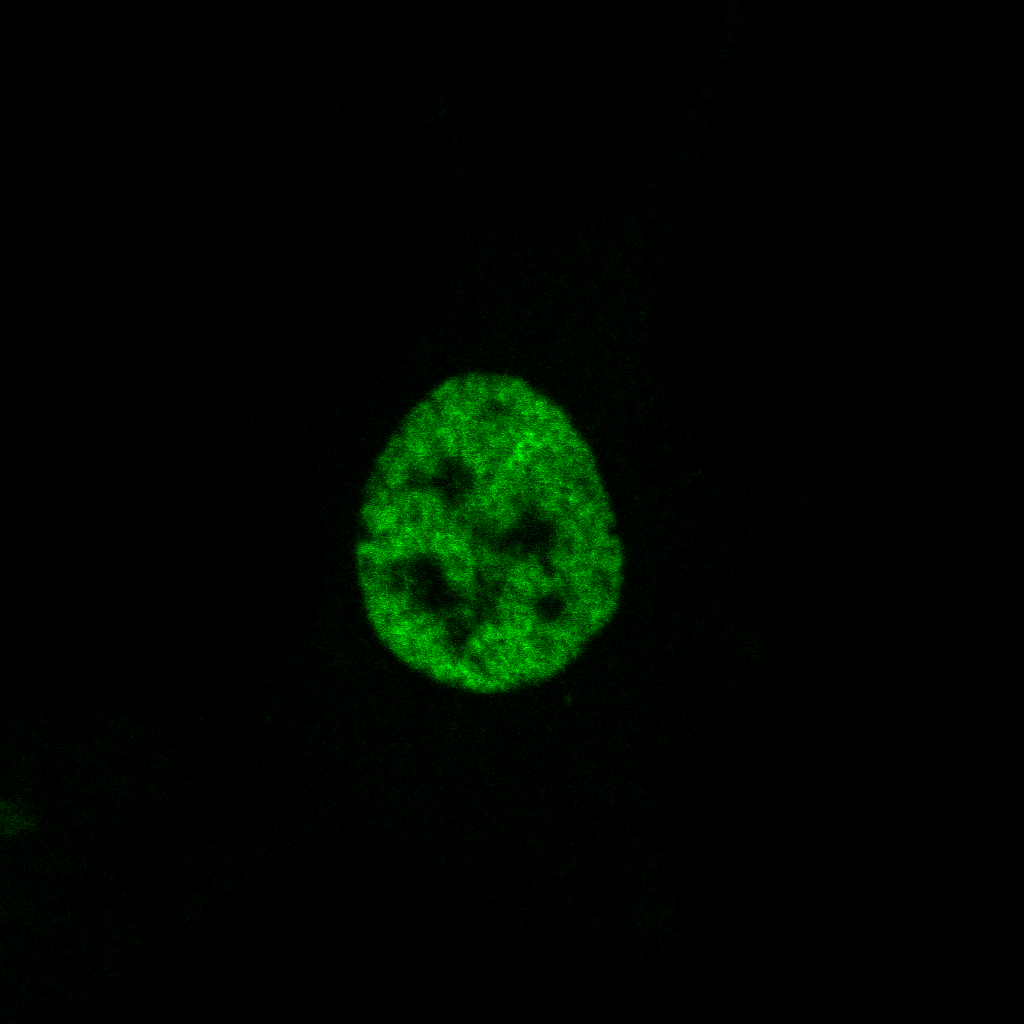

Supplement: Supplementary file 10 — Source Data for Figure 7 [file EMMM-15-e18024-s010.zip › Fig_7A-B/Fig_7B_ICC_image/sgHexim1+HMBA/p53.tiff]

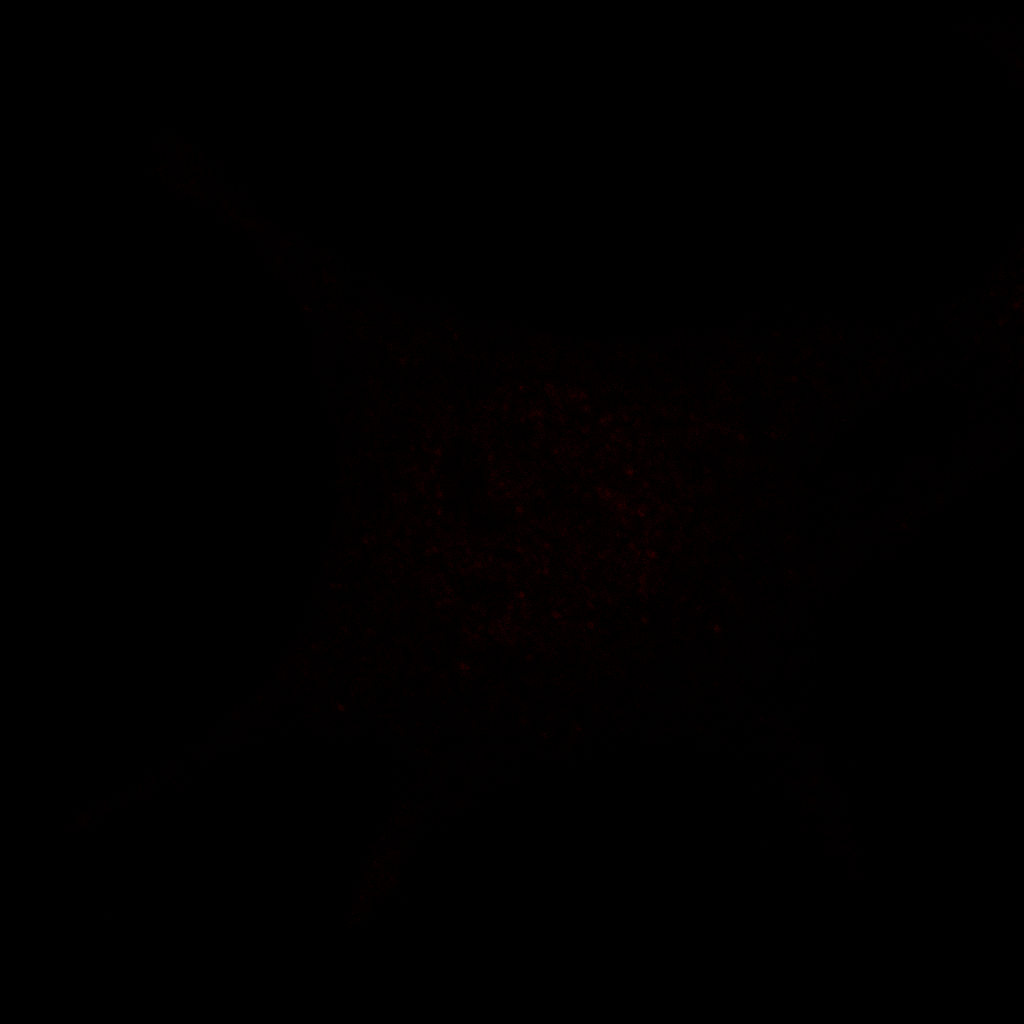

Supplement: Supplementary file 10 — Source Data for Figure 7 [file EMMM-15-e18024-s010.zip › Fig_7A-B/Fig_7B_ICC_image/sgHexim1+Vehicle/HEXIM1.tiff]

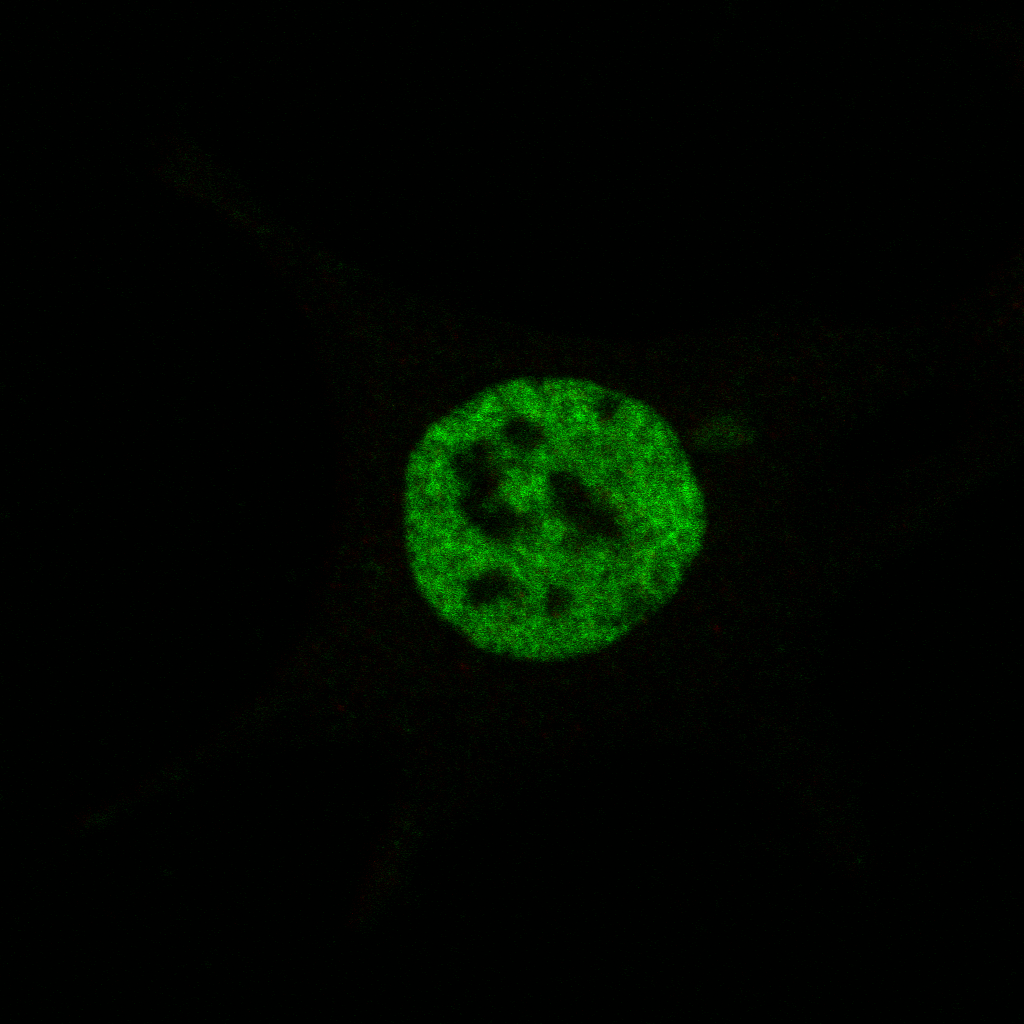

Supplement: Supplementary file 10 — Source Data for Figure 7 [file EMMM-15-e18024-s010.zip › Fig_7A-B/Fig_7B_ICC_image/sgHexim1+Vehicle/HEXIM1_p53.tiff]

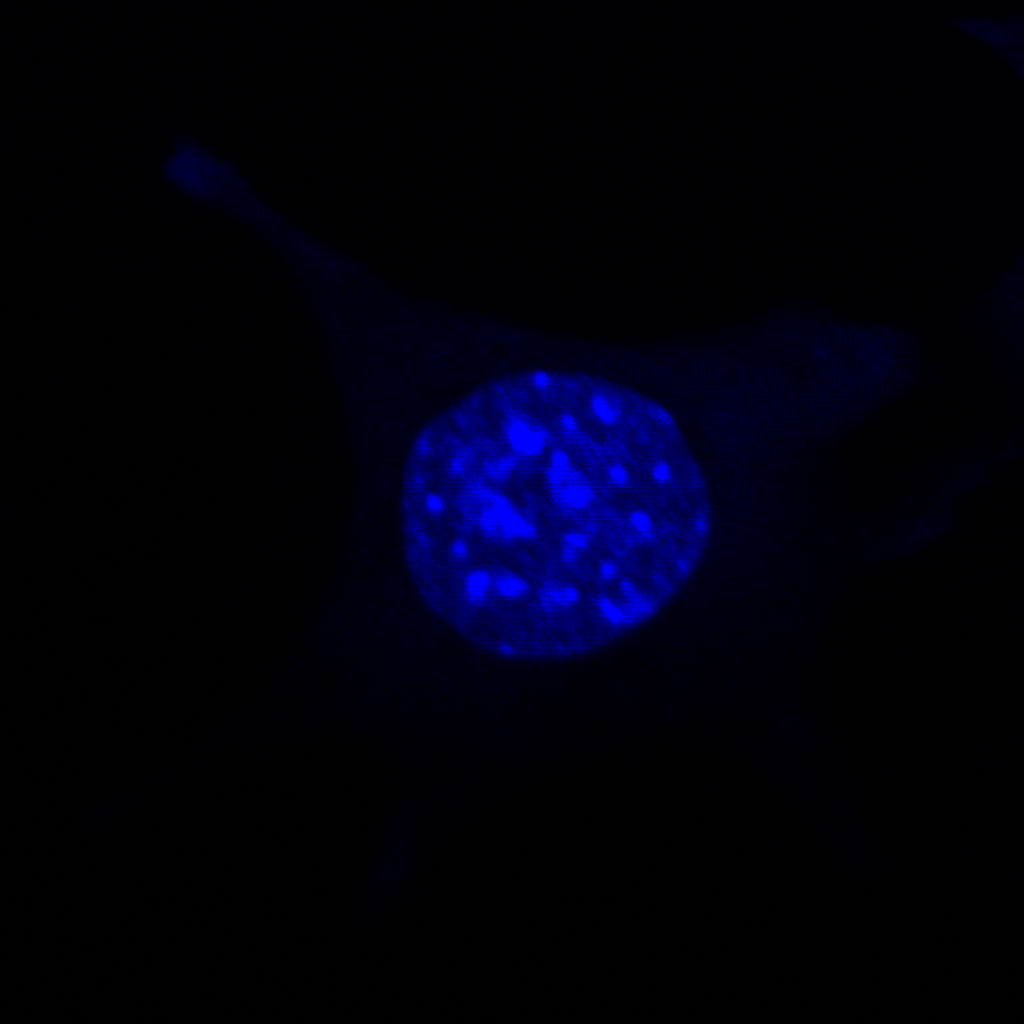

Supplement: Supplementary file 10 — Source Data for Figure 7 [file EMMM-15-e18024-s010.zip › Fig_7A-B/Fig_7B_ICC_image/sgHexim1+Vehicle/Hoechst.tiff]

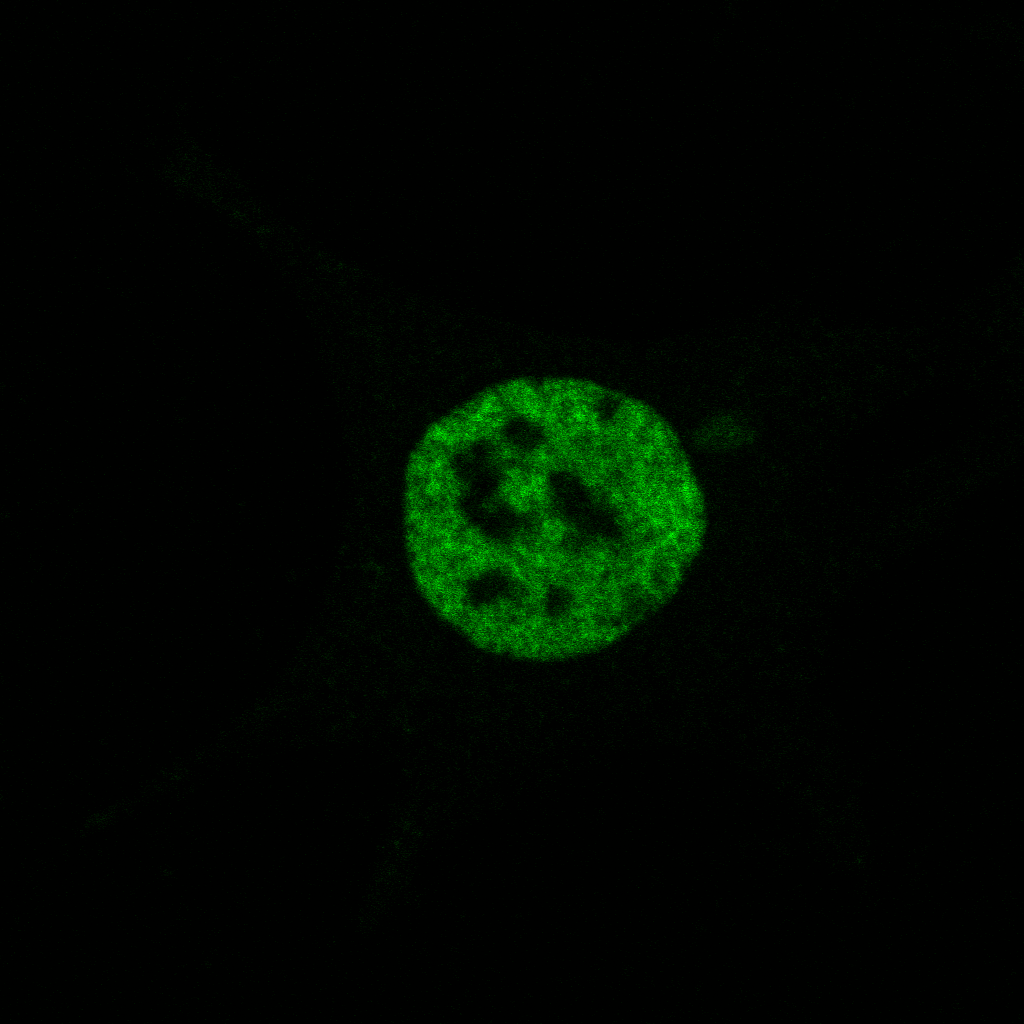

Supplement: Supplementary file 10 — Source Data for Figure 7 [file EMMM-15-e18024-s010.zip › Fig_7A-B/Fig_7B_ICC_image/sgHexim1+Vehicle/p53.tiff]
