# Supplementary material for: HMBA ameliorates obesity by MYH9‐ and ACTG1‐dependent regulation of hypothalamic neuropeptides
Source: EMBO Mol Med. 2023 Nov 20;15(12):e18024. doi: 10.15252/emmm.202318024 (PMC10701615; doi:10.15252/emmm.202318024)

Uncropped blots for Figure 6

Fig. 6A

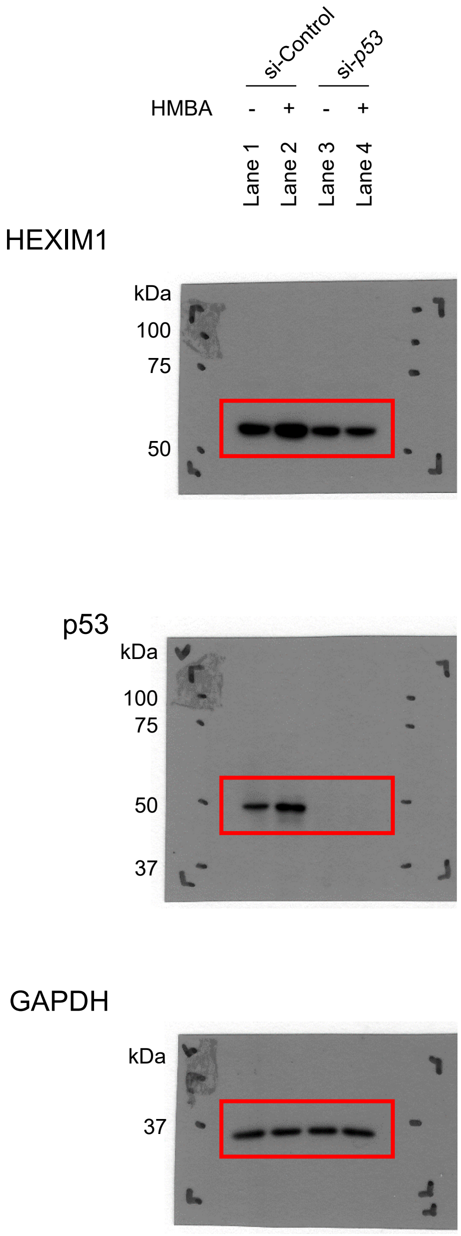

Fig. 6C

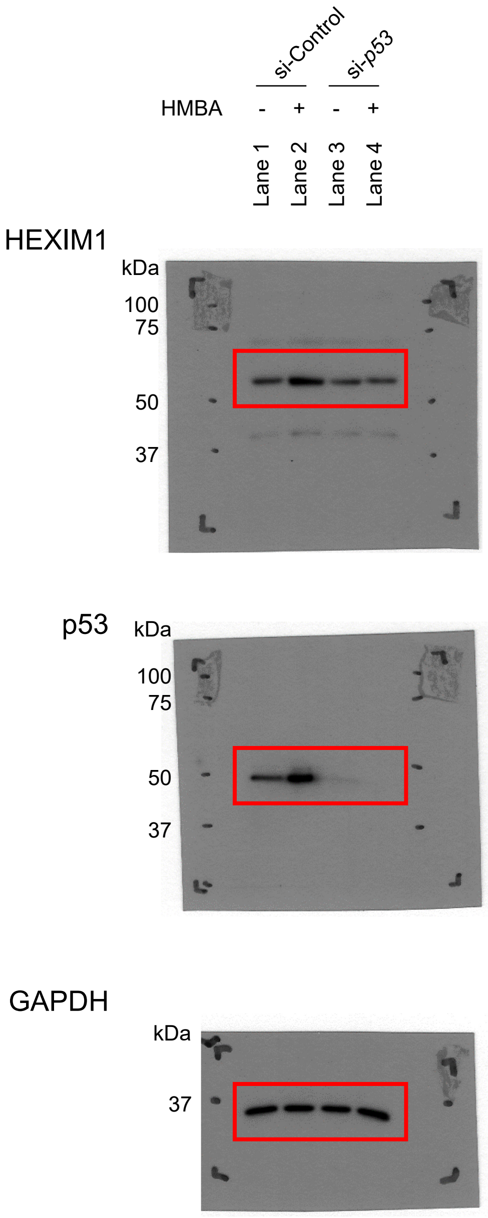

Uncropped blots for Figure 6

Fig. 6E

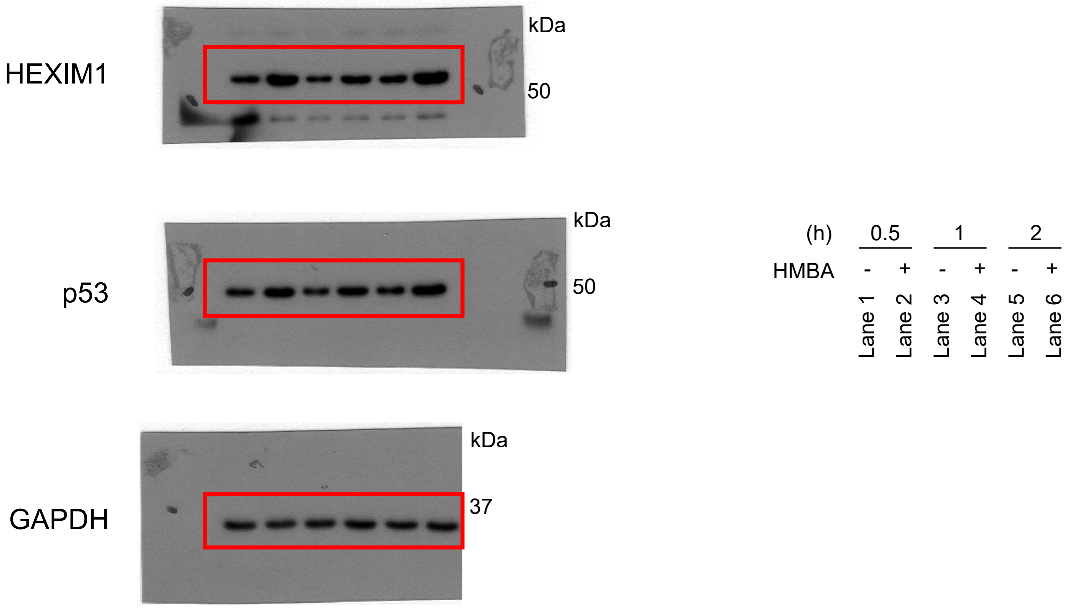

Fig. 6G

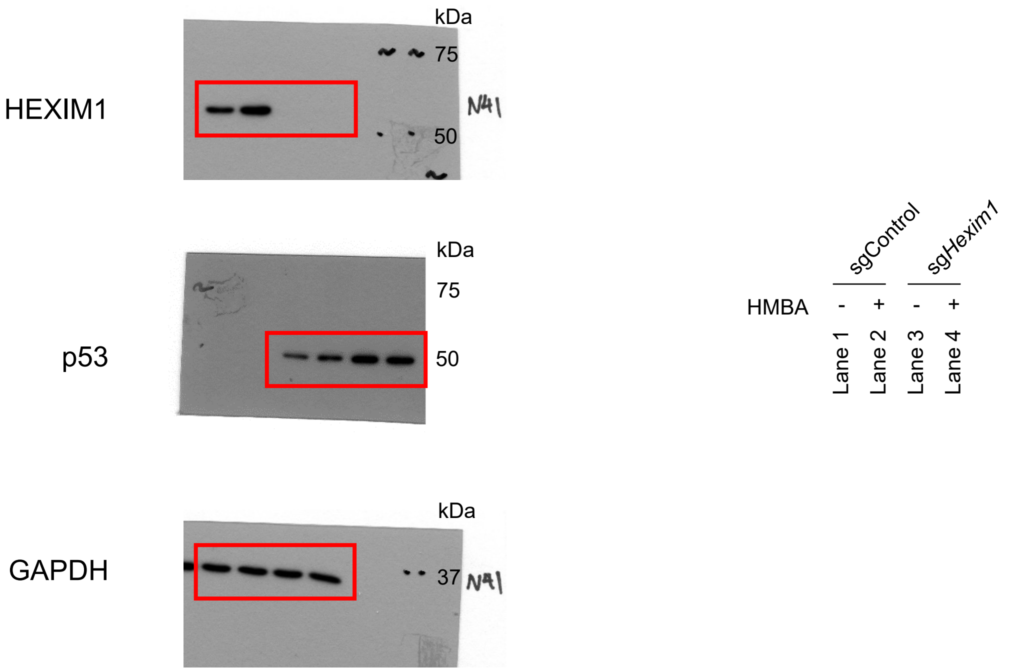

Uncropped blots for Figure 6

Fig. 6H

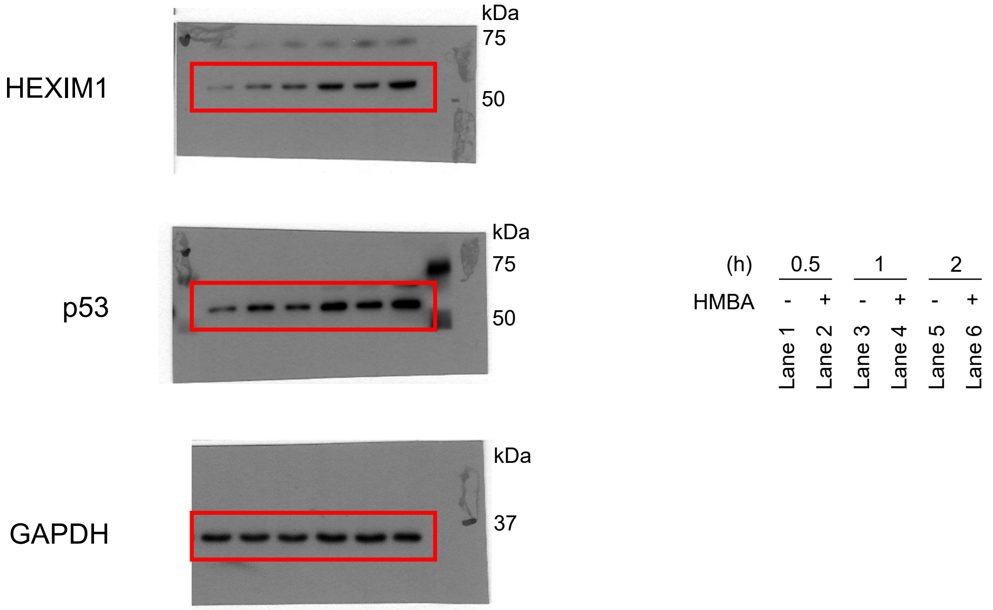

Fig. 6J

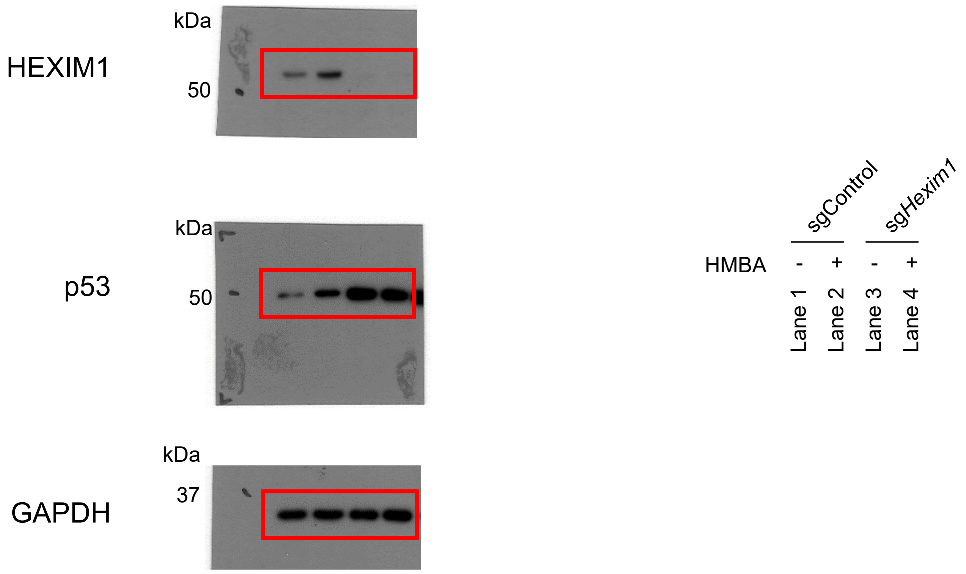

Supplement: Supplementary file 9 — Source Data for Figure 6 [file EMMM-15-e18024-s011.zip › Fig_6_Uncropped_blots_(A,C,E,G,H,J).pdf]
