# Supplementary material for: HMBA ameliorates obesity by MYH9‐ and ACTG1‐dependent regulation of hypothalamic neuropeptides
Source: EMBO Mol Med. 2023 Nov 20;15(12):e18024. doi: 10.15252/emmm.202318024 (PMC10701615; doi:10.15252/emmm.202318024)
Supplement: Supplementary file 10 — Source Data for Figure 7 [file EMMM-15-e18024-s010.zip › Fig_7_Uncropped_blots_(C,D).pdf]

Uncropped blots for Figure 7

Fig. 7C

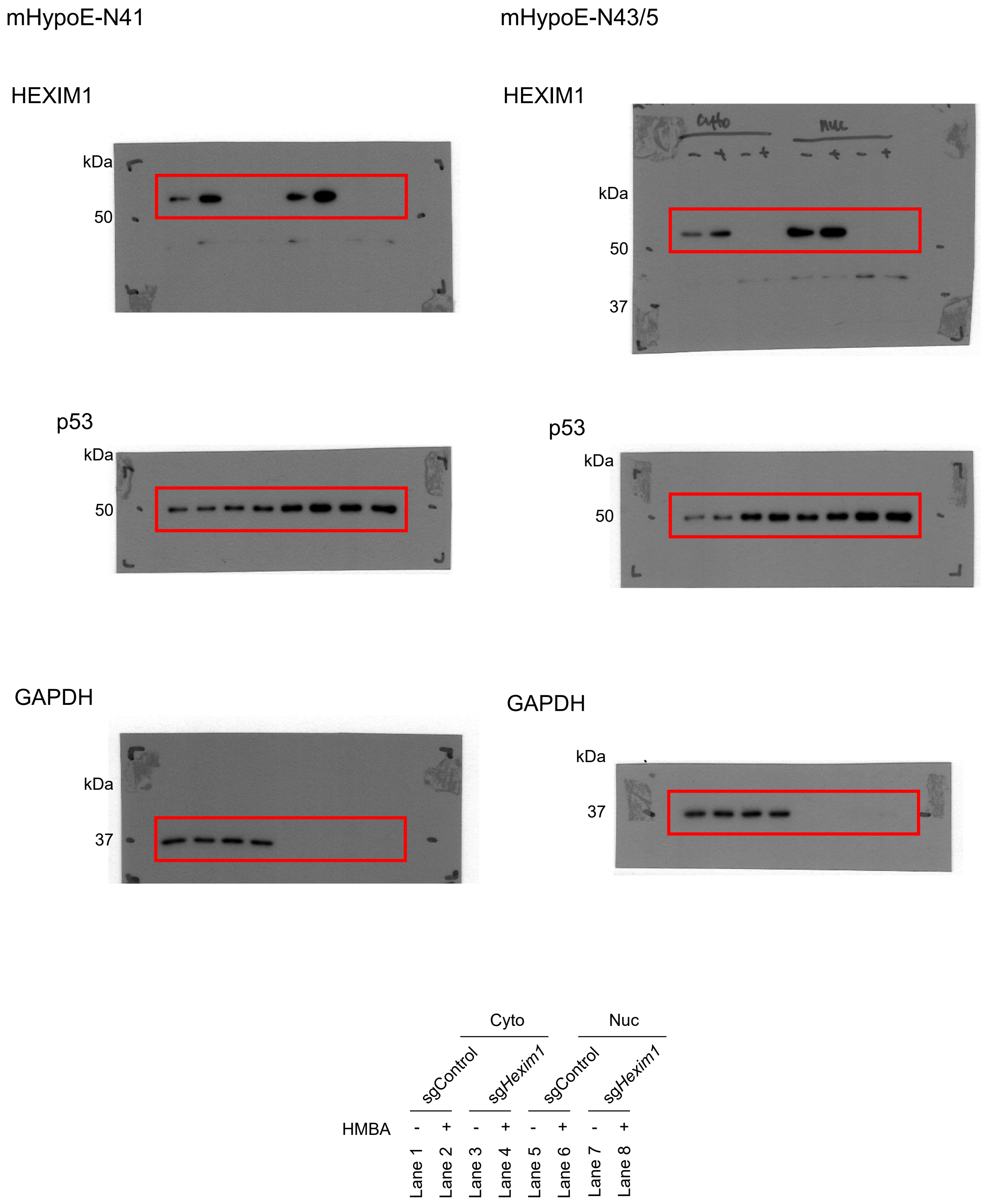

Uncropped blots for Figure 7

Fig. 7D

mHypoE-N41

IP: HEXIM1

mHypoE-N43/5

IP: HEXIM1

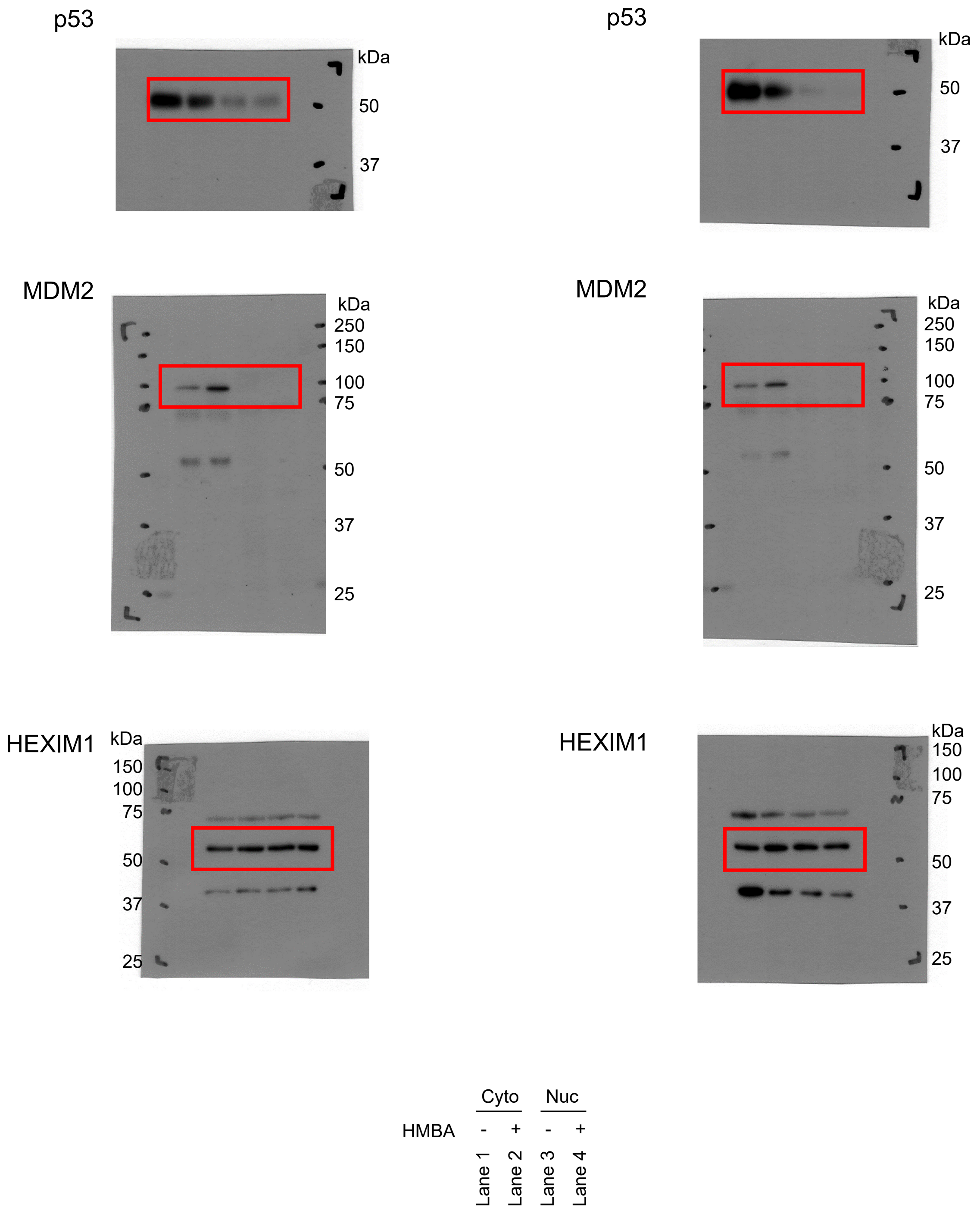

Uncropped blots for Fig 7D

Fig. 7D

mHypoE-N41 | mHypoE-N43/5

IP: p53 | IP: p53

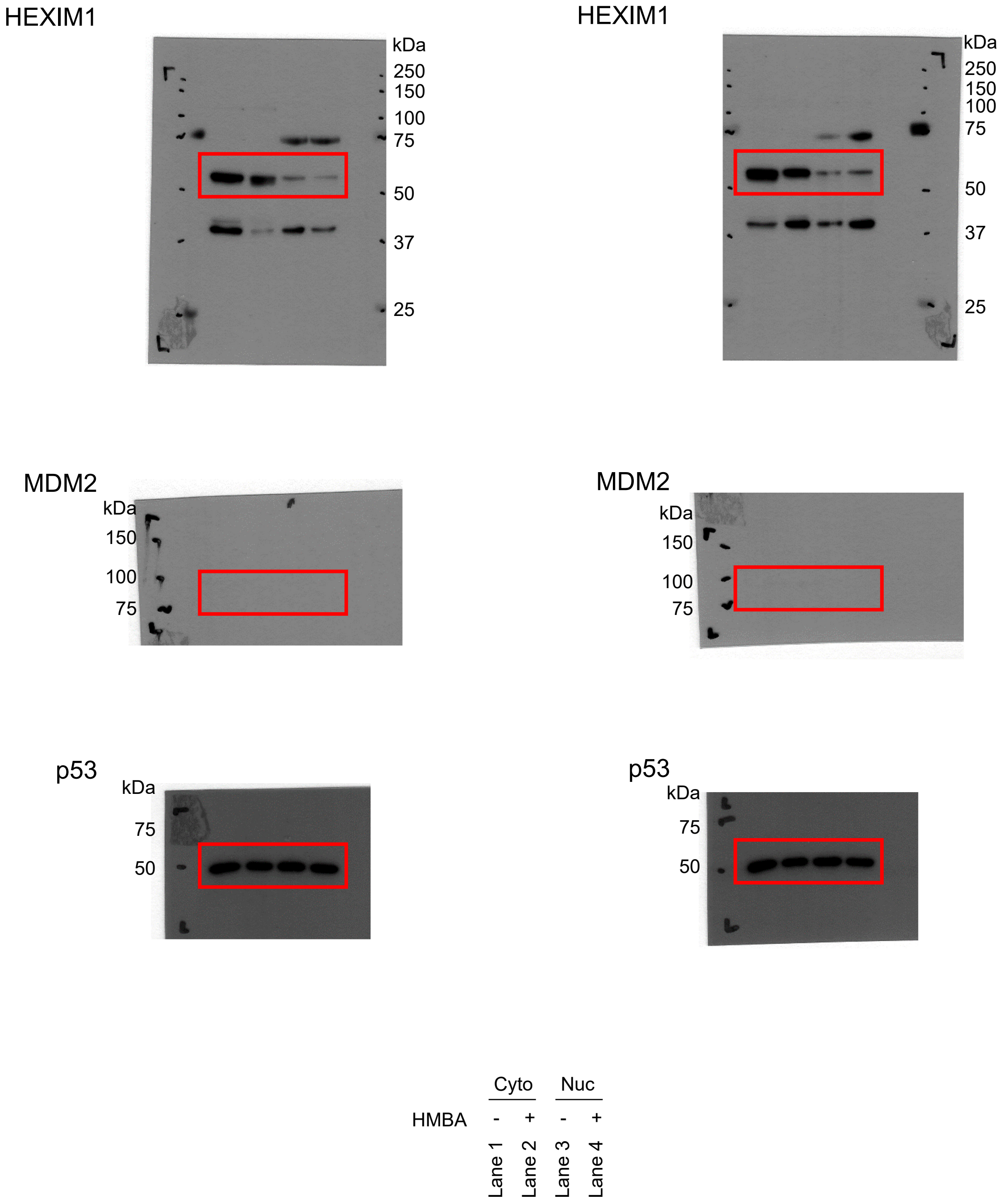

Uncropped blots for Fig 7D

Fig. 7D

mHypoE-N41

IP: p53

HEXIM1

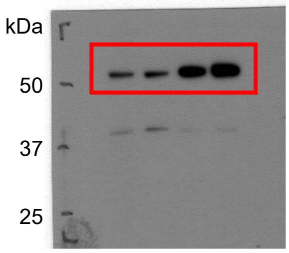

p53

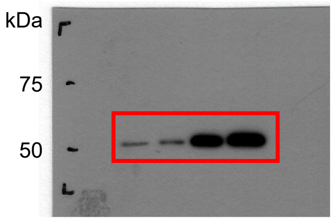

MDM2

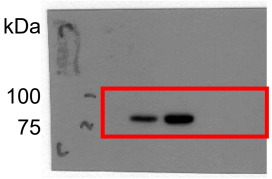

mHypoE-N43/5

IP: p53

HEXIM1

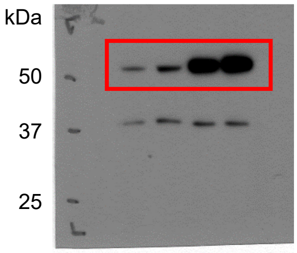

p53

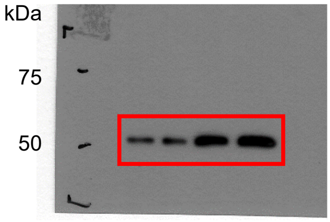

MDM2

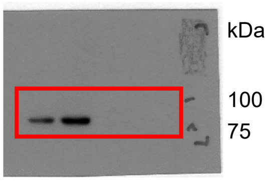

	Cyto		Nuc	
HMBA	-	+	-	+
	Lane 1	Lane 2	Lane 3	Lane 4
