# Supplementary material for: HMBA ameliorates obesity by MYH9‐ and ACTG1‐dependent regulation of hypothalamic neuropeptides
Source: EMBO Mol Med. 2023 Nov 20;15(12):e18024. doi: 10.15252/emmm.202318024 (PMC10701615; doi:10.15252/emmm.202318024)
Supplement: Supplementary file 11 [file EMMM-15-e18024-s007.pdf]

EMBO Molecular Medicine

HMBA ameliorates obesity by MYH9 and ACTG1-dependent regulation of hypothalamic neuropeptides

Seokjae Park, Sungjoon Oh, Nayoun Kim and Eun-Kyoung Kim
DOI: 10.15252/emmm.202318024

Corresponding author: Eun-Kyoung Kim (ekkim@dgist.ac.kr)

Review Timeline:

Submission Date:	17th May 23
Editorial Decision:	14th Jun 23
Appeal:	20th Jun 23
Editorial Decision:	20th Jun 23
Revision Received:	15th Sep 23
Editorial Decision:	9th Oct 23
Revision Received:	19th Oct 23
Accepted:	27th Oct 23

Editor: Lise Roth

Transaction Report:

(Note: With the exception of the correction of typographical or spelling errors that could be a source of ambiguity, letters and reports are not edited. Depending on transfer agreements, referee reports obtained elsewhere may or may not be included in this compilation. Referee reports are anonymous unless the Referee chooses to sign their reports.)

1st Editorial Decision 14th Jun 2023

13th Jun 2023

Decision on your manuscript EMM-2023-18024

Dear Dr. Kim,

Thank you for the submission of your manuscript to EMBO Molecular Medicine. We have now received feedback from the three referees who had agreed to review your manuscript.

As you will see from the reports below, the referees acknowledge the potential interest of the study, but also raise several major and partially overlapping concerns, such as unclear rationale, potential toxicity of HMBA, missing information, inadequate statistics, use of non-neuronal cells, etc.

Based on the referees' reports, I am afraid I see little choice but to return the manuscript to you at this point with the decision that we cannot offer to publish it.

While we cannot pursue this manuscript further, we encourage you to transfer your study to our not-for-profit open-access sister journal, Life Science Alliance (LSA). We shared your manuscript and the accompanying reviews with LSA Executive Editor, Eric Sawey, who is interested in these findings, and would like to invite further consideration of this manuscript at LSA pending the following revisions:
- Address Reviewer 1's comments regarding Pomc and Npy expression effects on body weight, and the minor issues listed.
- Address Reviewer 2's comments.
- Address Reviewer 3's comments.

We understand that such a revision might need to be re-reviewed, in which case, Dr. Sawey will walk the Reviewers through our transfer process. We encourage you to use the link below to transfer your manuscript to LSA. You do not need to revise the manuscript before transferring it to LSA. Once you transfer, Dr. Sawey will email you an invitation to revise and resubmit, listing the same revision requests as mentioned above. Please feel free to reach out at e.sawey@life-science-alliance.org if you have any questions about the LSA journal, the transfer process or the revisions requested.

I am very sorry to disappoint you on this occasion and I hope you will view the possibility of a transfer favorably. If this is the case, please use the link below to transfer the manuscript directly.

With kind regards,

Lise Roth

Lise Roth
Senior Editor
EMBO Molecular Medicine

Referee #1 (Remarks for Author):

In this study, the authors aimed to explore the potential of hexamethylene 3 bisacetamide (HMBA) as a treatment for obesity. Their experiments on diet-induced obese mice demonstrated positive effects on weight loss through both peripheral and central administration of HMBA. The authors discovered that HMBA interacts with MYH9 and ACTG1 proteins in hypothalamic cells, leading to an increase in HEXIM1 expression and its interaction with MDM2. This interaction results in the dissociation of the HEXIM1-p53 complex. Subsequently, the released HEXIM1 and p53 proteins translocate to the nucleus of hypothalamic cells in vitro. Within the nucleus, these proteins downregulate the transcription of the appetite-stimulating neuropeptide NPY while simultaneously upregulating the transcription of the appetite-suppressing neuropeptide POMC. This modulation of gene transcription contributes to the regulation of appetite and energy balance.

The study presents novel findings regarding the role of the HMBA-Hexim1 axis in energy metabolism and offers new insights into feeding regulation. It is important to note that a relatively high dose of HMBA (500-1000 mg) was used in the study. However, it remains uncertain whether the observed impact on food intake and body weight can be attributed to sickness

behavior induced by HMBA treatment. If sickness behavior is indeed responsible, it would diminish the therapeutic potential of HMBA.

Concerns arise when examining the data presented in Figure 2b, where intracerebroventricular (icv) injection of HMBA led to a near-complete cessation of feeding on multiple days. This substantial reduction in food intake cannot be adequately explained by a modest alteration in POMC or NPY mRNA levels. Furthermore, the manuscript suggests that HMBA treatment reduces body weight and food intake by increasing Pomc expression and reducing NPY expression in adult mice. However, the evidence supporting the hypothesis that adult increases in Pomc or adult reductions in NPY directly result in decreased body weight is neither cited nor supported by references. Additionally, considering that Hex1m1 is widely expressed in hypothalamic cells, it is doubtful that HMBA has a specific effect on Pomc or NPY neurons.

While the authors conducted various mechanistic studies in the manuscript, it is important to note that many of these studies were performed in "hypothalamic" cells that are not neurons. Therefore, it remains to be determined whether key components of the signaling cascade actually occur in hypothalamic neurons, such as Pomc or NPY neurons.

In terms of minor issues:
1. The manuscript does not provide validation for the expression of Myh9 and ACTG1, nor does it confirm their knockout by shRNA in Figure 5.
2. The remarkable increase in brown adipose tissue (BAT) temperature depicted in Figure 1g is noteworthy. However, the manuscript does not provide specific information regarding whether the mice developed a fever following HMBA treatment.

Referee #2 (Remarks for Author):

This manuscript describes the effects of HMBA on hypothalamic neuropeptides and defines some of the molecular events that are involved in this process. The authors indicate that HMBA causes anorexia and weight loss, although it is not completely clear how much weight is lost as they do not provide enough information on the weights of the mice. There are some concerns with some of the methodology and a few questions.

Comments:
1. The title is overstated considering the authors never demonstrate that the mice are obese - no weights are provided. So clearly HMBA does not "ameliorate obesity". The mouse weights should be provided and the changes in food intake provided. The graph used is somewhat deceiving as it is in percent and it is unclear exactly how much weight the mice lost overall.

2. Since HMBA has been shown to be toxic in humans (albeit at higher concentrations), the authors should provide evidence that the mice are not sick at this concentration thus causing the weight change.

3. The experiments lack any female mice. Do females have the same effect?

4. The body weights of the mice should be included for all animal experiments.

5. The number of replicates in the IP and IV injection experiments are confusing (the neuropeptide mRNA quantification is only n=3, the IP experiment overall is only n=5 overall, etc). Please clarify why replicates are not consistent.

6. The use of a less stringent t-test is not appropriate when a two-way ANOVA should have been used.

7. Is 4 weeks of HFD in C57/BL6 mice sufficient for an obese model?

8. The dose of HMBA used in the cell lines is a magnitude higher than what is used in the ICV. Why was this dose chosen?

9. In the GFP mice experiments, HMBA injection doesn't decrease food intake in the scrambled controls but any injection (HMBA or saline) in the GFP mice leads to increased food intake, which is causing the significant difference reported.

10. A potential explanation or commentary for why knockout of HEXIM1 completely abolishes Npy mRNA expression would be helpful.

11. Central administration of HMBA reduced appetite in chow-fed mice (Supplementary Fig. 1). However, the injected group only had significantly reduced food intake in 3 discontinuous days, where there were no significant changes the rest of the days during the 20-day measurements. The observed differences may be due to daily fluctuation or external stress (change cage/ top up food/ change water bottle). The authors claim that central administration of HMBA reduced appetite in chow-fed mice (Supplementary Fig. 1). The authors should compare the cumulated food intake to supplement their statement. If food intake does not change, an alternative mechanism may be responsible for the weight loss effect of HMBA, at least in the chow-fed group.

12. Why was a 500 mg/kg dose used for the first i.v. injection but 1000 mg/kg for all the other days and i.p. injections. Please provide a rationale.

13. Did the authors focus on the effect of HMBA in their cell line experiments because 80% of the metabolites detected in the cell lines was HMBA while only 16% was NADAH? However, since NADAH is one of the dominant metabolites they found in the hypothalamus of ICV injected mice, perhaps treatment with NADAH is appropriate to see whether it has similar metabolic effects to HMBA before focusing solely on HMBA.

14. There is a lack of consistency in using IP, IV, or ICV injected groups for experimental measurements. For instance, the level of HMBA in the plasma and hypothalamus and hypothalamic neuropeptide mRNA expression was measured in both IP and IV groups. However, all the metabolic phenotype measurements (e.g., thermogenesis, fat and lean mass, energy expenditure) were done only in the IV group but not theIP injected group. ITT, GTT, and plasma lipid profile were only measured in ICV injected groups. Consistent measurements should be provided for all experimental paradigms.

15. For the AAV experiments, they only have a sh Control group but no Cre negative control group. Why was this control not included?

16. Most of their neuropeptide qPCR experiments have extremely small error bars (figure 3). They mentioned that 3 independent experiments were conducted, but it is unclear if they are referring to independent biological replicates or technical replicates. The variance expected with lowly expressed genes (such as POMC) would likely be larger.

17. How do the authors explain the contradictory finding that HMBA increases Hexim1 expression and binding to the Npy promoter and decreases Npy, however at the same time, Hexim1 KD decreases Npy? Is Hexim1 an inhibitor of Npy with HMBA but an activator in basal conditions? How would this work mechanistically?

18. Comment on health of mice in Fig 2b (ICV mice) where food intake drops to nearly 0 g - are the mice sick?

19. Fig 5 l,m,n,o - sh control+DIO+saline group is missing. Can authors confirm that sh control+DIO+saline has the same weight gain as sh Myh9+sh Actg1 + DIO+saline (i.e. knocking down these factors in Npy/Pomc neurons doesn't automatically reduce weight/food intake without HMBA).

20. Overall this is an interesting story with some intriguing molecular findings, however the results as presented are somewhat confusing and need significant clarification to enhance confidence in the results.

Referee #3 (Comments on Novelty/Model System for Author):

Analysis of Physiologiocal data out of animal experiment are not clear and thus tt does not allow a full appreciation if the model is suitable or not

Referee #3 (Remarks for Author):

The authors highlight a central site of action for HMDA, a molecule used in oncology. They correctly demonstrate the cascade chain of HMDA action on gene expression of genes involved in energy homeostasis. This is a remarkable job of mechanistic description in order to validate the different intermediates of HMDA action (central and periphery injection). The authors identify MYH9 and ACTG1 as possible players in the hypothalamic regulation of energy balance.
Although the authors highlight a new role for this molecule, the choice of methods for analyzing physiological data is inappropriate and weakens the potential interest in this molecule. As it stands, some of the article's data need to be re-analyzed (proposed improvements presented below).
Finally, the choice to investigate HMDA alone, puzzled me. An effort of rationalization and explanation seems necessary to explain the choice of exploring the role of HMDA amongst other molecule, presented here by the simple homology (among other molecules) with an orexigenic peptide (Oleylethanolamide).
Overall, this is a complete and well-written article, and the methods are complete (notwithstanding a few modifications). It brings potential interest in opening up fields of exploration with application in human health. And so I'm in favor of it being considered for publication notwithstanding major revisions.

L19: "...often associated with dysfunction of the hypothalamus in the brain" too reductionist replace by "central nervous system" or It will be more appropriate to indicate an impairment of hypothalamic integration of peripheral signals and/or extrahypothalamic structures, as it is well established that defects in central energy homeostasis is the multiple and implicate internals and externals cues.
L56 "its role in maintaining energy balance is also emerging" The role P53 in metabolism is referenced as back as early 2000's, I suggest the author to cite a much earlier reference, but this reference is appropriate towards the role of P53 in regards of

hypothalamic neurons.
L112: missing line withdrawal

Major Concerns
"Our study points to a previously unappreciated efficacy of HMBA".
The authors highlight the underestimated role of HMBA in the regulation of energy balance (Abstract), however, the authors do not indicate any elements or citations indicating clues to this potential function. It is only in the Results section, using "The Connectivity Map" interface, that the authors establish their hypothesis of HMBA's role based on homology with Oleylethanolamide. I would suggest that the authors supplement the rationale for HMDA with existing observations of body weight change (publications of Saravanan N as a start which described mixed change in body weight in some model). Indeed, as also indicated by the authors (Supplementary Table 1) other molecules respond with similar homology, so why HMDA? It's a pity that the authors don't offer an explanation for the choice of this molecule.

Results and Methods
Overall, the choice and sequencing of methods and techniques is sound, well chosen, and supports the authors' questions. However, several details are missing and inappropriate methods analysis, impact the full appreciation of the results.
Address and country of suppliers are missing (ex:Alfa Aesar, Koatech, plastic One etc..) throughout the method section.
L426. Indicates the exact times of the light/dark schedule
L429: Mandatory, the authors should indicate the reference number of their approved agreement of use of animals and care committee.
L460 section: i.v., indicates which vein was chosen. Please, could the authors be more precise about the time of injection.
L477: ICV, indicates the volume injected, and the duration of injection. Please indicates the time of injection.
Throughout the methods, indicates when animals were grouped or individualized.
L499 Calorimetry
Mandatory: 1 day of acclimatization in Colombus cage is very short, plus the authors do not indicate at which day of the injection procedure the animals enter in the calorimetric system. Could the authors provide a more precise experimental design?
Please, could the authors provide in supplemental the full figures of calorimetric analysis (Days of adaptation, if available + day of measurement).
Major concern.
Mandatory: As the body weight change over time, I would recommend strongly the authors to perform Calorimetric data analysis using body weight as covariates (Virtue, S., Lelliott, C.J. & Vidal-Puig, A. What is the most appropriate covariate in ANCOVA when analyzing metabolic rate?. Nat Metab 3, 1585 (2021). The significant change in EE observed under ICV treatment may be consecutive to the change in body mass. See: Fernández-Verdejo, R., Ravussin, E., Speakman, J.R. et al. Progress and challenges in analyzing rodent energy expenditure. Nat Methods 16, 797-799 (2019).
L513 Brown adipose tissue
Please, indicates how many picture of the animals was taken.
In order to properly determine a change in the thermogenic activity of bat in vivo, I suggest that the authors measure the surface temperature of the lower back on the same photos, and then extract the ratio between both regions to normalize the temperature per animal and make appropriate comparison. (Meyer CW, Ootsuka Y, Romanovsky AA. Body Temperature Measurements for Metabolic Phenotyping in Mice. Front Physiol. 2017 Jul 31;8:520. doi: 10.3389/fphys.2017.00520.)
Fig 1 and 2: RER (VCO_2/VO_2)
L111: Central administration of HMBA
Calorimetric analysis on figures 2 was performed on the last day in contrary of IV injection. Could the authors explain the reason?
Please provides the food intake pattern if available with the calorimetric analysis.
Please indicates on the Fig 1b and 2b, when treatment occurs or stops.
Major concern.
Food intake (HFD) decrease dramatically at the end of the 7 days of treatment. Such a reduction in food intake jeopardize the animal's health. Although the authors indicate: (1) a change in the expression of hypothalamic peptides involved in the regulation of energy balance, following the knocking down MYH9 and ACTG1 (downstream of HMBA) and thus that changes could drive the change in energy balance, It cannot be ruled out that the dramatic change in intake may also be due to the deleterious nature of the molecule of interest.
L152, L201, Fig3, F4 .
Very nice parts, authors well demonstrate in vivo and in vitro the dependent relationship form HMBA exposure, change in Hexim1 expression, determinant role of Myh9 and Actg1, hence expression of NPY and POMC. However, the authors should indicate whether HMBA exposure compromises cell survival and/or presents a pro-inflammatory or apoptotic state. Those missing information would re enforce the finding and avoid implying that the molecule is potentially harmful.

L334. Fig 7g. Nice summary and nice graph.

Discussion:
Well written, but overestimated interpretation, as metabolic data must be revisited according to the above suggestions.

==========================

As a service to authors, EMBO provides authors with the possibility to transfer a manuscript that one journal cannot offer to publish to another EMBO publication. The full manuscript and if applicable, reviewers reports are automatically sent to the receiving journal to allow for fast handling and a prompt decision on your manuscript. For more details of this service, and to transfer your manuscript to another EMBO title please click on Link Not Available

Please do not share this URL as it will give anyone who clicks it access to your account.

Dear Editor Roth,

Thank you very much for processing our manuscript and allowing us to receive comments from the reviewers. We appreciate their valuable comments to help improve our manuscript and also feel strongly that we can fully address their concerns.

For several major concerns that you summarized, we can provide better rationale and information, and provide data to show the non-toxicity of HMBA at the doses used in our study.

In regard to the use of non-neuronal cells, there are no suitable in vitro models for each NPY or POMC-expressing hypothalamic neuron at present. Since the use of cell models for the mechanistic study is inevitable, we utilized NPY- or POMC-expressing mouse embryonic hypothalamic cells which have been available and appropriate cell models for understanding the regulation of their expression. As complementary evidence, we confirmed the efficacy of HMBA and the roles of HMBA-binding partner proteins using NPY or POMC-expressing neuron-specific Cre or GFP transgenic mice in vivo, and their results are consistent with those obtained from in vitro cell lines.

Many of the comments raised by reviewers are about editorial correction and reformatting of the current data without requests for massive experimental works. Some of the comments are repetitive and a few comments are from a misunderstanding of our manuscript. For example, presentation by changes in body weight (%) is a more common practice in the field of metabolism. The information regarding body weight (grams) will be added to the figures as pointed out by the reviewer. The information on the approved agreement of the use of animals and care committee was removed because of our anonymous submission. We appropriately performed statistical analyses using two-way ANOVA with multiple comparisons of Tukey or Bonferroni post hoc test for data from more than two groups, whereas a Student’s t-test was

used only for determining the significant difference between two groups. I would say that we are confident in addressing all of the other comments as well.

I hope I do not appear to be arguing against your decision or reviewers' opinions. Despite the need to improve our manuscript, I feel that our findings discover the new efficacy of HMBA and provide its mode of action as a potent anti-obesity compound, which might be sufficiently interesting to researchers in this field. We would greatly appreciate you considering our request for a second chance at handling our manuscript if we provide a point-by-point response to all of the comments.

Thank you very much for your time and consideration of my request in advance.

Sincerely yours,

Eun-Kyoung

Eun-Kyoung Kim, Ph.D

Professor and Director

Department of Brain Sciences

Neurometabolomics Research Center

DGIST

Republic of Korea

20th Jun 2023

Dear Dr. Kim,

Thank you for your e-mail asking us to reconsider our decision on your manuscript. I have discussed your points with my colleagues here, and we agreed that, should you provide a revised version of your manuscript addressing all the referees' concerns, we would consider it.

Please note that convincingly showing the absence of toxicity of HMBA will be essential and addressing the other reviewers' concerns in full will be necessary for further considering the manuscript in our journal. A revised manuscript will once again be subject to review. Based on the initial reports and on the amount of work needed to address the referees' concerns, we cannot guarantee at this stage that the eventual outcome will be favorable.

EMBO Molecular Medicine encourages a single round of revision only and therefore, acceptance or rejection of the manuscript will depend on the completeness of your responses included in the next, final version of the manuscript. For this reason, and to save you from any frustrations in the end, I would strongly advise against returning an incomplete revision. Should you find that the requested revisions are not feasible within the constraints outlined here and prefer, therefore, to submit your paper elsewhere, we would welcome a message to this effect.

Revised manuscripts should be submitted within three months of a request for revision; they will otherwise be treated as new submissions, except under exceptional circumstances in which a short extension is obtained from the editor.

When submitting your revised manuscript, please carefully review the instructions that follow below. We perform an initial quality control of all revised manuscripts before re-review; failure to include requested items will delay the evaluation of your revision.

We require:

1) A .docx formatted version of the manuscript text (including legends for main figures, EV figures and tables). Please make sure that the changes are highlighted to be clearly visible.

2) Individual production quality figure files as .eps, .tif, .jpg (one file per figure). For guidance, download the 'Figure Guide PDF' (https://www.embopress.org/page/journal/17574684/authorguide#figureformat).

3) At EMBO Press we ask authors to provide source data for the main figures. Our source data coordinator will contact you to discuss which figure panels we would need source data for and will also provide you with helpful tips on how to upload and organize the files.

4) A .docx formatted letter INCLUDING the reviewers' reports and your detailed point-by-point responses to their comments. As part of the EMBO Press transparent editorial process, the point-by-point response is part of the Review Process File (RPF), which will be published alongside your paper.

5) A complete author checklist, which you can download from our author guidelines (https://www.embopress.org/page/journal/17574684/authorguide#submissionofrevisions). Please insert information in the checklist that is also reflected in the manuscript. The completed author checklist will also be part of the RPF.

6) Please note that all corresponding authors are required to supply an ORCID ID for their name upon submission of a revised manuscript.

7) It is mandatory to include a 'Data Availability' section after the Materials and Methods. Before submitting your revision, primary datasets produced in this study need to be deposited in an appropriate public database, and the accession numbers and database listed under 'Data Availability'. Please remember to provide a reviewer password if the datasets are not yet public (see https://www.embopress.org/page/journal/17574684/authorguide#dataavailability).

In case you have no data that requires deposition in a public database, please state so in this section. Note that the Data Availability Section is restricted to new primary data that are part of this study.

8) For data quantification: please specify the name of the statistical test used to generate error bars and P values, the number (n) of independent experiments (specify technical or biological replicates) underlying each data point and the test used to calculate p-values in each figure legend. The figure legends should contain a basic description of n, P and the test applied. Graphs must include a description of the bars and the error bars (s.d., s.e.m.). Please provide exact p values.

9) Our journal encourages inclusion of *data citations in the reference list* to directly cite datasets that were re-used and obtained from public databases. Data citations in the article text are distinct from normal bibliographical citations and should directly link to the database records from which the data can be accessed. In the main text, data citations are formatted as follows: "Data ref: Smith et al, 2001" or "Data ref: NCBI Sequence Read Archive PRJNA342805, 2017". In the Reference list, data citations must be labeled with "[DATASET]". A data reference must provide the database name, accession number/identifiers and a resolvable link to the landing page from which the data can be accessed at the end of the reference. Further instructions are available at .

10) We replaced Supplementary Information with Expanded View (EV) Figures and Tables that are collapsible/expandable online. A maximum of 5 EV Figures can be typeset. EV Figures should be cited as 'Figure EV1, Figure EV2" etc... in the text and their respective legends should be included in the main text after the legends of regular figures.
- For the figures that you do NOT wish to display as Expanded View figures, they should be bundled together with their legends in a single PDF file called *Appendix*, which should start with a short Table of Content. Appendix figures should be referred to in the main text as: "Appendix Figure S1, Appendix Figure S2" etc.
- Additional Tables/Datasets should be labeled and referred to as Table EV1, Dataset EV1, etc. Legends have to be provided in a separate tab in case of .xls files. Alternatively, the legend can be supplied as a separate text file (README) and zipped together with the Table/Dataset file.
See detailed instructions here:

11) The paper explained: EMBO Molecular Medicine articles are accompanied by a summary of the articles to emphasize the major findings in the paper and their medical implications for the non-specialist reader. Please provide a draft summary of your article highlighting
- the medical issue you are addressing,
- the results obtained and
- their clinical impact.
This may be edited to ensure that readers understand the significance and context of the research. Please refer to any of our published articles for an example.

12) For more information: There is space at the end of each article to list relevant web links for further consultation by our readers. Could you identify some relevant ones and provide such information as well? Some examples are patient associations, relevant databases, OMIM/proteins/genes links, author's websites, etc...

13) Author contributions: CRediT has replaced the traditional author contributions section because it offers a systematic machine readable author contributions format that allows for more effective research assessment. Please remove the Authors Contributions from the manuscript and use the free text boxes beneath each contributing author's name in our system to add specific details on the author's contribution. More information is available in our guide to authors.

14) Disclosure statement and competing interests: We updated our journal's competing interests policy in January 2022 and request authors to consider both actual and perceived competing interests. Please review the policy https://www.embopress.org/competing-interests and update your competing interests if necessary.

15) Every published paper now includes a 'Synopsis' to further enhance discoverability. Synopses are displayed on the journal webpage and are freely accessible to all readers. They include a short stand first (maximum of 300 characters, including space) as well as 2-5 one-sentences bullet points that summarizes the paper. Please write the bullet points to summarize the key NEW findings. They should be designed to be complementary to the abstract - i.e. not repeat the same text. We encourage inclusion of key acronyms and quantitative information (maximum of 30 words / bullet point). Please use the passive voice. Please attach these in a separate file or send them by email, we will incorporate them accordingly.

Please also suggest a striking image or visual abstract to illustrate your article as a PNG file 550 px wide x 300-600 px high.

16) As part of the EMBO Publications transparent editorial process initiative (see our Editorial at http://embomolmed.embopress.org/content/2/9/329), EMBO Molecular Medicine will publish online a Review Process File (RPF) to accompany accepted manuscripts.
In the event of acceptance, this file will be published in conjunction with your paper and will include the anonymous referee reports, your point-by-point response and all pertinent correspondence relating to the manuscript. Let us know whether you agree with the publication of the RPF and as here, if you want to remove or not any figures from it prior to publication.
Please note that the Authors checklist will be published at the end of the RPF.

EMBO Molecular Medicine has a "scooping protection" policy, whereby similar findings that are published by others during review or revision are not a criterion for rejection. Should you decide to submit a revised version, I do ask that you get in touch after three months if you have not completed it, to update us on the status.

I look forward to receiving your revised manuscript.

Yours sincerely,

Lise Roth

Lise Roth, PhD
Senior Editor
EMBO Molecular Medicine

EMM-2023-18024-V3

Reviewer Comments:

Referee #1 (Remarks for Author):

In this study, the authors aimed to explore the potential of hexamethylene bisacetamide (HMBA) as a treatment for obesity. Their experiments on diet-induced obese mice demonstrated positive effects on weight loss through both peripheral and central administration of HMBA. The authors discovered that HMBA interacts with MYH9 and ACTG1 proteins in hypothalamic cells, leading to an increase in HEXIM1 expression and its interaction with MDM2. This interaction results in the dissociation of the HEXIM1-p53 complex. Subsequently, the released HEXIM1 and p53 proteins translocate to the nucleus of hypothalamic cells in vitro. Within the nucleus, these proteins downregulate the transcription of the appetite-stimulating neuropeptide NPY while simultaneously upregulating the transcription of the appetite-suppressing neuropeptide POMC. This modulation of gene transcription contributes to the regulation of appetite and energy balance.

The study presents novel findings regarding the role of the HMBA-Hexim1 axis in energy metabolism and offers new insights into feeding regulation. It is important to note that a relatively high dose of HMBA (500-1000 mg) was used in the study. However, it remains uncertain whether the observed impact on food intake and body weight can be attributed to sickness behavior induced by HMBA treatment. If sickness behavior is indeed responsible, it would diminish the therapeutic potential of HMBA.

Author Response:

We understand the concern raised by the reviewer. To address the reviewer's concern, we provide critical evidence that the dose we used in vivo did not cause any sickness behavior. First, we observed that none of the mice displayed any noticeable signs of sickness such as vomiting, diarrhea, inactivity, and hair loss during HMBA experiments. Our metabolic cage data also showed no negative effect of HMBA on locomotor activity compared with saline-injected mice. In order to demonstrate the health status of the mice more accurately, we employed the laboratory animal behavior observation registration and analysis system (LABORAS), a robust tool renowned for its capability in automated behavioral

scoring, after the last dosing of HMBA (i.v., i.p., or i.c.v.). This sophisticated system accurately measures a range of behaviors, including immobility, rearing, locomotion, climbing, grooming, eating, and drinking. Our LABORAS data demonstrated that eating was decreased by HMBA treatment, however, no significant differences in the other behaviors were observed compared to the saline-injected group (Expanded View Figure 1A, 1B, 1D, 1E, 1G, 1H). These results indicate that HMBA does not induce sickness behaviors in mice. We also performed a conditioned taste aversion (CTA) test to assess the effect of HMBA on a taste aversion associated with an illness over sucrose preference. The CTA test showed that HMBA did not induce a taste aversion, either (Expanded View Figure 1C, 1F, 1I). Collectively, our data demonstrate that HMBA did not exhibit sickness-associated or aversive appetite suppression and weight loss under our experimental conditions. The details are elaborated in the Results of the revised manuscript.

Concerns arise when examining the data presented in Figure 2b, where intracerebroventricular (icv) injection of HMBA led to a near-complete cessation of feeding on multiple days. This substantial reduction in food intake cannot be adequately explained by a modest alteration in POMC or NPY mRNA levels. Furthermore, the manuscript suggests that HMBA treatment reduces body weight and food intake by increasing Pomc expression and reducing NPY expression in adult mice. However, the evidence supporting the hypothesis that adult increases in Pomc or adult reductions in NPY directly result in decreased body weight is neither cited nor supported by references.

Author Response:

Pharmacological interventions with compounds or hormones enabling modulation of neuropeptide expression in ARC are effective in the control of food intake and body weight. I.p. injection of C75, a fatty acid synthase inhibitor, almost blocked food intake (<0.2 g in 24 h, ~95% drop) with a 50% reduction of *Npy* and *Agrp* but no changes in *Pomc* and *Cart* mRNA levels in obese adult mice (1). Also, i.c.v. injection of C75 drastically reduced food intake (<0.1 g in 24 h, ~90% drop) with a 60% reduction of *Npy*, 40% *Agrp* and a 40% elevation of *Pomc* mRNA levels in non-obese adult mice (2, 3). In another study with adult rats, i.c.v. injection of aminoprocalcitonin, a neuroendocrine peptide, suppressed food

intake (< 1 g in 1 h, ~90% drop) with a 60% reduction of *Npy*, 40% *Agrp* and a 25% elevation of *Pomc* mRNA levels (4, 5). These studies show that even a modest change in *Npy* or *Pomc* mRNA levels can lead to a substantial reduction in food intake and weight loss in adult animals.

Other numerous studies show that the levels of NPY and POMC are important for body weight in adults because they react to energy status and integrate signals from the periphery to regulate neuronal circuits responsible for feeding and energy expenditure directly or indirectly by coordinating activation and controlling the neuroendocrine and autonomic nervous system (6-11). We included and cited the relevant references in the Introduction.

Additionally, considering that Hexim1 is widely expressed in hypothalamic cells, it is doubtful that HMBA has a specific effect on Pomc or NPY neurons.

Author Response:

We are grateful to the reviewer for raising this critical issue. To clarify whether HMBA has a specific effect on NPY or POMC neurons, we investigated the expression of HEXIM1 in diverse cells such as neurons, microglia, and astrocytes in the hypothalamus of NPY- or POMC-GFP mice injected with HMBA. Our immunohistochemical analyses revealed that HEXIM1 is mainly expressed in neurons and hardly detected in astrocytes and microglia. Notably, HMBA increased the expression of HEXIM1 in NPY and POMC neurons but not in other neurons or glia in the ARC (Expanded View Figure 4). Although we do not exclude the possibility that the other hypothalamic areas could be also affected by HMBA, our results suggest that HMBA specifically regulates HEXIM1 expression in NPY and POMC neurons rather than other cells in the ARC.

While the authors conducted various mechanistic studies in the manuscript, it is important to note that many of these studies were performed in "hypothalamic" cells that are not neurons. Therefore, it remains to be determined whether key components of the signaling cascade actually occur in hypothalamic neurons, such as Pomc or NPY neurons.

Author Response:

In regard to the use of non-neuronal cells, there are no suitable in vitro models for each NPY- or POMC-expressing hypothalamic neuron at present. Since the use of cell models for the mechanistic study is inevitable, we utilized NPY or POMC-expressing mouse embryonic hypothalamic cells which have been available and appropriate cell models for understanding the regulation of their expressions. As complementary evidence in vivo, we confirmed the efficacy of HMBA and the roles of HMBA downstream proteins such as HEXIM1, MYH9 and ACTG1 in NPY- or POMC neuron-specific Cre or GFP transgenic mice, and the results were consistent with those obtained from in vitro cell lines.

In terms of minor issues:

1. The manuscript does not provide validation for the expression of Myh9 and ACTG1, nor does it confirm their knockout by shRNA in Figure 5.

Author Response:

To address this issue, we validated the knockdown of *Myh9* and *Actg1* in *Npy*-expressing AgRP or POMC neurons using immunohistochemistry after injecting the AAV-shRNA into the ARC of AgRP- or POMC-Cre DIO mice (Figure 5F, 5G). Our data confirmed the efficient knockdown of *Myh9* and *Actg1* as well as their expression in NPY and POMC neurons.

2. The remarkable increase in brown adipose tissue (BAT) temperature depicted in Figure 1g is noteworthy. However, the manuscript does not provide specific information regarding whether the mice developed a fever following HMBA treatment.

Author Response:

As demonstrated in behavioral experiments, HMBA-treated mice displayed no sickness behaviors, indicating that the BAT temperature increased by HMBA is not due to one of the symptoms of sickness. *Ucp1* is used as a reliable marker for BAT thermogenesis activity, and its expression remains unaltered

by the pyrogenic temperature elevation associated with fever (12, 13). Our data show that the mRNA level of *Ucp1* in the BAT was increased by HMBA, indicating that an increase in BAT thermogenesis was not due to fever. This information is elaborated in Figure 1L and Figure 2G and the Results of the revised manuscript.

References

1. Shimokawa T et al., *Proc Natl Acad Sci*, 2002;99(1)66-71
2. Loftus TM et al., *Science*, 2000;288(5475)2379-2381
3. Hu Z et al., *Proc Natl Acad Sci*, 2003;100, 12624-12629
4. Tavares E et al., *Am J Physiol Endocrinol Metab*, 2013;304(12)E1251-1262
5. Tavares E et al., *Endocrinology*, 2007;148(4)1891-1901
6. Stanley BG et al., *Proc Natl Acad Sci*, 1985;82, 3940-3943
7. Poggioli R et al., *Peptides*, 1986;7, 843-848
8. Garcia de Yebenes E et al., *Brain Res*, 1995;674, 112-116
9. Huszar D et al., *Cell*, 1997;88, 131-141
10. Brady LS et al., *Neuroendocrinology*, 1990;52, 441-447
11. Vohra MS et al., *Eur J Pharmacol*, 2022;915, 174611
12. Okamatsu-Ogura Y et al., *AM J Physiol Endocrinol Metab*, 2007;292(4)e1135-9.
13. Eskilsson A et al., *FASEB J*, 2020;34(4)5863-5876.

Referee #2 (Remarks for Author):

This manuscript describes the effects of HMBA on hypothalamic neuropeptides and defines some of the molecular events that are involved in this process. The authors indicate that HMBA causes anorexia and weight loss, although it is not completely clear how much weight is lost as they do not provide enough information on the weights of the mice. There are some concerns with some of the methodology and a few questions.

Comments:

1. The title is overstated considering the authors never demonstrate that the mice are obese - no weights are provided. So clearly HMBA does not "ameliorate obesity". The mouse weights should be provided and the changes in food intake provided. The graph used is somewhat deceiving as it is in percent and it is unclear exactly how much weight the mice lost overall.

Author Response:

As the presentation of changes in body weight (%) is a more common practice, the changes in body weight (%) were used to show the weight loss effect in our study. To address the reviewer's concerns, data on body weight and food intake (grams) were added to the figures for all animal experiments. We used high-fat diet-fed mice weighing over 32 g, which were more than 8 g heavier than those of age-matched mice fed a normal chow diet.

2. Since HMBA has been shown to be toxic in humans (albeit at higher concentrations), the authors should provide evidence that the mice are not sick at this concentration thus causing the weight change.

Author Response:

We understand the concern raised by the reviewer. To address the reviewer's concern, we provide critical evidence that the dose we used in vivo did not cause any sickness behavior. First, we observed that none of the mice displayed any noticeable signs of sickness such as vomiting, diarrhea, inactivity, and hair loss during HMBA experiments. Our metabolic cage data also showed no negative effect of

HMBA on locomotor activity compared with saline-injected mice. Secondly, in order to demonstrate the health status of the mice more accurately, we employed the laboratory animal behavior observation registration and analysis system (LABORAS), a robust tool renowned for its capability in automated behavioral scoring, after the last dosing of HMBA (i.v., i.p., or i.c.v.). This sophisticated system accurately measures a range of behaviors, including immobility, rearing, locomotion, climbing, grooming, eating, and drinking. Our LABORAS data demonstrated that eating was decreased by HMBA treatment, however, no significant differences in the other behaviors were observed compared to the saline-injected group (Expanded View Figure 1A, 1B, 1D, 1E, 1G, 1H). These results indicate that HMBA does not induce sickness behaviors in mice. Lastly, we performed a conditioned taste aversion (CTA) test to assess the effect of HMBA on a taste aversion associated with an illness over sucrose preference. The CTA test showed that HMBA did not induce a taste aversion, either (Expanded View Figure 1C, 1F, 1I). Collectively, our data demonstrate that HMBA did not exhibit sickness-associated or aversive appetite suppression and weight loss under our experimental conditions. The details are elaborated in the Results of the revised manuscript.

3. The experiments lack any female mice. Do females have the same effect?

Author Response:

As the reviewer suggested, we conducted i.v., i.p., and i.c.v. injection of HMBA in female DIO mice (Appendix Figure S1). Our data showed that each injection of HMBA had very similar effects on the reduction in body weight and food intake in females as seen in HMBA-injected DIO males. It is likely that HMBA has the same effects on food intake and body weight regardless of gender. Please note that DIO females fed 4-week HFD weigh 22-24g vs. NCD females (~18g) (1).

4. The body weights of the mice should be included for all animal experiments.

Author Response:

We hope that we appropriately addressed this issue in our response to reviewer's comment #1.

5. The number of replicates in the IP and IV injection experiments are confusing (the neuropeptide mRNA quantification is only n=3, the IP experiment overall is only n=5 overall, etc). Please clarify why replicates are not consistent.

Author Response:

We apologize for the confusion. Due to the limited number of experiments possible with a single set of animals, additional sets were used for different metabolic measurements. Although the number of replicates was not the same across all measurements, the number of replicates was kept within a range of 3-6.

6. The use of a less stringent t-test is not appropriate when a two-way ANOVA should have been used.

Author Response:

We performed statistical analyses using a two-way ANOVA with multiple comparisons of Tukey or Bonferroni post hoc tests for data having more than two groups, whereas a Student's t-test was used to determine the significant difference between the two groups. However, as suggested, the statistical analysis using a t-test was changed to a two-way ANOVA except for body weight and food intake between the two groups in the revised manuscript (Figure 2O, 2P, 5F, 5G, 6F, 6I). The t-test used for body weight and food intake data between the two groups was deemed to be appropriate as reported in many other references (2-6).

7. Is 4 weeks of HFD in C57/BL6 mice sufficient for an obese model?

Author Response:

Mice fed an HFD for 4 weeks are sufficient for an obese model as validated in numerous references. For instance, the obese phenotype such as body weight, hyperglycemia, and fat composition typically develop within 4 weeks of 60 kcal% HFD (7, 8). In addition, HFD given to young mice resulted in more

severe obesity compared to those given to adult mice (8, 9). Thus, we fed mice with HFD for 4 weeks starting at age 4 weeks, which resulted in a weight gain of over 8 g (>32g, more than a 33% increase) compared to those of NCD mice (<24 g). Notably, we used C57BL/6N mice which develop greater obesity and impaired glucose sensitivity compared to those of C57BL/6J mice (10).

8. The dose of HMBA used in the cell lines is a magnitude higher than what is used in the ICV. Why was this dose chosen?

Author Response:

The information about the pharmacokinetics of HMBA is largely lacking and the drug's efficacy can vary between in vitro and in vivo. According to the data on cell viability across the dynamic range of HMBA treatment (0.001-10 mM) (Appendix Figure S4), the concentration of HMBA (0.1 mM) used in cell lines was within the range that did not reduce cell viability. In addition, HMBA (0.1 mM) does not induce apoptosis as evidenced by no increases in cleaved forms of caspases 3 and 9 (Appendix Figure S8). More importantly, we demonstrated dose-dependent changes in *Npy* and *Pomc* expression in the cell lines treated with HMBA, and within the non-toxic range of dosages, 0.1 mM HMBA exerted the most significant changes on *Npy* and *Pomc* expression (Figure 3H, 3I). Thus, this dosage was chosen to elucidate the underlying mechanism in vitro.

9. In the GFP mice experiments, HMBA injection doesn't decrease food intake in the scrambled controls but any injection (HMBA or saline) in the GFP mice leads to increased food intake, which is causing the significant difference reported.

Author Response:

We did not measure food intake using GFP mice in our study. NPY- or POMC-GFP DIO mice were injected with HMBA or saline, and then a change in HEXIM1 expression in those neurons was analyzed.

10. A potential explanation or commentary for why knockout of HEXIM1 completely abolishes Npy mRNA expression would be helpful.

Author Response:

Our data showed that HMBA-induced HEXIM1 decreased *Npy* mRNA expression (Figure 3O). Surprisingly, the genetic knockout of HEXIM1 abolished *Npy* mRNA expression even in the absence of HMBA.

When we performed ChIP-qPCR analysis following HEXIM1 knockout, H3K9Ac binding to the *Npy* promoter was significantly reduced by HMBA and more significantly by HEXIM1 knockout regardless of the presence of HMBA (Response Figure 1), which is very similar to the change in *Npy* mRNA expression (Figure 3O). One explanation would be that HEXIM1 may be involved directly or indirectly in the promoter binding of H3K9Ac that positively regulates *Npy* expression. It is likely that a basal level of HEXIM1 is required for *Npy* promoter binding of H3K9Ac in basal conditions. However, when HMBA induces the expression of HEXIM1 over its basal level, HEXIM1 binds to the *Npy* promoter instead of H3K9Ac to negatively regulate *Npy* expression. It has been known that some transcription factors (e.g. C/EBPβ and Sp3) can play a dual role in transcriptional activation and repression (11, 12). These factors carry out the activating and repressing roles at the same locus in a promoter in a signal-dependent fashion. Like those factors, HEXIM1 might have a dual function in *Npy* gene expression; HEXIM1 might function as a transcription activator in basal conditions where HEXIM1 binds to the *Npy* promoter along with H3K9Ac (Figure 7E), whereas HEXIM1 bound to the promoter might function as a transcription repressor in HMBA-treated conditions through a not-yet-known mechanism.

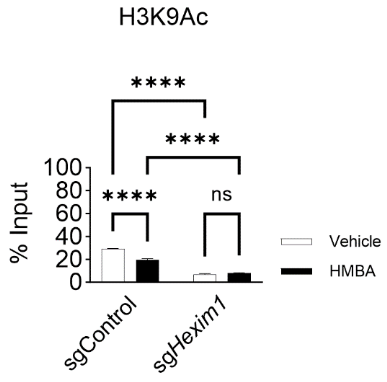

Response Figure 1

11. Central administration of HMBA reduced appetite in chow-fed mice (Supplementary Fig. 1). However, the injected group only had significantly reduced food intake in 3 discontinuous days, where there were no significant changes the rest of the days during the 20-day measurements. The observed differences may be due to daily fluctuation or external stress (change cage/ top up food/ change water bottle). The authors claim that central administration of HMBA reduced appetite in chow-fed mice (Supplementary Fig. 1). The authors should compare the cumulated food intake to supplement their statement. If food intake does not change, an alternative mechanism may be responsible for the weight loss effect of HMBA, at least in the chow-fed group.

Author Response:

As the reviewer commented, we compared cumulative food intake for the chow-fed group experiment. The cumulative food intake in HMBA-treated mice fed a chow diet was also decreased compared to saline-treated mice (Appendix Figure S2D). This result indicates that central administration of HMBA reduced appetite in chow-fed mice as well.

12. Why was a 500 mg/kg dose used for the first i.v. injection but 1000 mg/kg for all the other days and i.p. injections. Please provide a rationale.

Author Response:

I.v. injection is an effective and rapid method of delivering drugs into the body. Since multiple i.v. injections of HMBA to mice were planned, an i.v. injection with half of the working dose on the first day was chosen. This dosing is based on FDA guidelines (Dose-Response Information to Support Drug Registration, 1994) and has often been used in animal studies (13, 14) or phase I clinical trials (15) especially where compounds or drugs are repeatedly administered. Even when the dose for an i.v. injection on the first day was replaced by 1000 mg/kg, changes in body weight and food intake were similar to those in the group of 500 mg/kg dose (Response Figure 2).

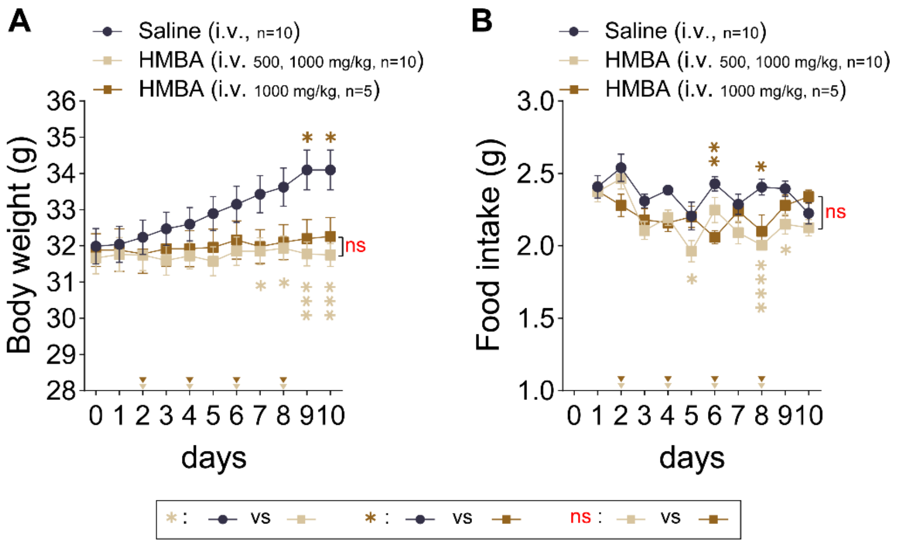

*Two-way ANOVA (Tukey's)

Response Figure 2

13. Did the authors focus on the effect of HMBA in their cell line experiments because 80% of the metabolites detected in the cell lines was HMBA while only 16% was NADAH? However, since NADAH is one of the dominant metabolites they found in the hypothalamus of ICV injected mice, perhaps treatment with NADAH is appropriate to see whether it has similar metabolic effects to HMBA before focusing solely on HMBA.

Author Response:

We appreciate the reviewer's comments. We did consider the potential effects of NADAH. Before we started in vivo work, we first investigated the change in protein levels of HEXIM1 and mRNA levels of *Npy* and *Pomc* in the cell lines treated with HMBA or NADAH (Response Figure 3–5). We observed that NADAH induced all of them less than HMBA does. Based on these preliminary data, we chose HMBA for in vivo study. However, it would be interesting to see whether NADAH has similar metabolic effects in vivo. The study with NADAH warrants further investigation.

Figures for reviewers removed.

14. There is a lack of consistency in using IP, IV, or ICV injected groups for experimental measurements. For instance, the level of HMBA in the plasma and hypothalamus and hypothalamic neuropeptide mRNA expression was measured in both IP and IV groups. However, all the metabolic phenotype measurements (e.g., thermogenesis, fat and lean mass, energy expenditure) were done only in the IV group but not theIP injected group. ITT, GTT, and plasma lipid profile were only measured in ICV injected groups. Consistent measurements should be provided for all experimental paradigms.

Author Response:

We first compared the effects of peripheral injection of HMBA between i.v. and i.p injected groups. The changes in body weight, food intake, and neuropeptide expression were more significant in i.v. groups than i.p. groups. Thus, we further validated the effects of HMBA on another metabolic phenotype such as calorimetry in the i.v. groups. Next, we observed greater effects of i.c.v. groups on body weight, food intake, and neuropeptide expression than i.v. groups. Therefore, the assessments on ITT, GTT, and plasma lipid profile were further added on top of calorimetric analyses in the i.c.v. group. Following this rationale, we focused on the HMBA injection method that led to more significant changes in metabolic parameters rather than conducting massive experimental measurements in all injection groups.

15. For the AAV experiments, they only have a sh Control group but no Cre negative control group. Why was this control not included?

Author Response:

Our objective was to examine whether the HMBA-induced changes in HEXIM1 expressions and metabolic phenotype are specific to AgRP/NPY or POMC neurons. We injected shControl and sh*Myh9*/*Actg1* AAV viruses carrying the flox gene into AgRP- or POMC-Cre mice to specifically silence *Myh9* and *Actg1* in AgRP or POMC neurons. Hence, we considered that the shControl group was more suitable than the no Cre negative control group.

16. Most of their neuropeptide qPCR experiments have extremely small error bars (figure 3). They mentioned that 3 independent experiments were conducted, but it is unclear if they are referring to independent biological replicates or technical replicates. The variance expected with lowly expressed genes (such as POMC) would likely be larger.

Author Response:

The datasets in our qPCR experiments in Figure 3 are comprised of three biological replicates and each biological replicate is an average of three technical replicates. If nine replicates were combined from all samples, it could give rise to larger error bars. Figure 3 presents the qPCR data obtained from cell lines, which may contribute to smaller error bars as well. We revised the sentence in the legends of all figures to clarify the replicates.

17. How do the authors explain the contradictory finding that HMBA increases Hexim1 expression and binding to the Npy promoter and decreases Npy, however at the same time, Hexim1 KD decreases Npy? Is Hexim1 an inhibitor of Npy with HMBA but an activator in basal conditions? How would this work mechanistically?

Author Response:

This issue is closely correlated with the reviewer's comment #10. Similar to the case of *Hexim1* knockout, HEXIM1 might function as a transcription activator in basal conditions where HEXIM1 binds to the *Npy* promoter along with H3K9Ac (Figure 7E), whereas HEXIM1 bound to the promoter might function as a transcription repressor in HMBA-treated conditions through a not-yet-known mechanism.

We hope that we appropriately explained this point in our response to the reviewer's comment #10.

18. Comment on health of mice in Fig 2b (ICV mice) where food intake drops to nearly 0 g - are the mice sick?

Author Response:

Through a series of behavioral and CTA tests, we demonstrated that the mice do not exhibit signs of illness (Expanded View Figure 1) as addressed in the reviewer's comment #2.

19. Fig 5 l,m,n,o - sh control+DIO+saline group is missing. Can authors confirm that sh control+DIO+saline has the same weight gain as sh Myh9+sh Actg1 + DIO+saline (i.e. knocking down these factors in Npy/Pomc neurons doesn't automatically reduce weight/food intake without HMBA).

Author Response:

We added the data on body weight and food intake of the shControl+DIO+Saline group to Figure 5 L–O and confirmed that there was no difference compared to the sh*Myh9*/*Actg1*+DIO+Saline group. The data also showed that the knockdown of *Myh9* and *Actg1* in *Npy*-expressing AgRP or POMC neurons had no effect on basal body weight and food intake in the absence of HMBA.

20. Overall this is an interesting story with some intriguing molecular findings, however the results as presented are somewhat confusing and need significant clarification to enhance confidence in the results.

Author Response:

We are thankful for the reviewer's positive assessment of our work. We earnestly hope that our responses successfully conveyed the required information for clarification.

References

1. Guerra-Cantera S et al., *Front Endocrinol*, 2021;12, 796661

2. Williams KW et al, *Cell Metab*, 2014;20(3)471-482.

3. Quinones M et al., *Nat Commun*, 2018;9, 3432

4. Jansson JO et al., *Proc Natl Acad Sci*, 2017;115(2)427-432

5. Liu C et al., *Diabetes*, 2023;72(4)467-482

6. Gonzalez-Garcia I et al., *Metab Clin Exp*, 2022;129, 155122

7. Wang CY et al., *Methods Mol Biol*, 2012;821:421-433.

8. Dias MM et al., *Diabetol Metab Syndr*, 2021;13:32.

9. Krishna KB et al., *Physiol Rep*, 2016;4(6):e12708

10. Odom MR et al., *Int J Impot Res*, 2022;34(3):308-316.

11. Mo X et al., *Mol Cell*, 2004;13(2):241-250

12. Valin A et al., *Biochem Soc Trans*, 2007;35(6):1393-1396

13. Kutchy NA et al., *Metabolites*, 2023;13(3):434

14. Leonardo M et al., *Sci Rep*, 2023;13:799

15. Shi A et al., *Adv Ther*, 2021;38:550-561

Referee #3 (Comments on Novelty/Model System for Author):

Analysis of Physiological data out of animal experiment are not clear and thus it does not allow a full appreciation if the model is suitable or not.

Author Response:

We reanalyzed the relevant physiological data and revised our manuscript as advised by the referee.

Referee #3 (Remarks for Author):

The authors highlight a central site of action for HMDA, a molecule used in oncology. They correctly demonstrate the cascade chain of HMDA action on gene expression of genes involved in energy homeostasis. This is a remarkable job of mechanistic description in order to validate the different intermediates of HMDA action (central and periphery injection). The authors identify MYH9 and ACTG1 as possible players in the hypothalamic regulation of energy balance.

Although the authors highlight a new role for this molecule, the choice of methods for analyzing physiological data is inappropriate and weakens the potential interest in this molecule. As it stands, some of the article's data need to be re-analyzed (proposed improvements presented below).

Finally, the choice to investigate HMDA alone, puzzled me. An effort of rationalization and explanation seems necessary to explain the choice of exploring the role of HMDA amongst other molecule, presented here by the simple homology (among other molecules) with an orexigenic peptide (Oleylethanolamide).

Overall, this is a complete and well-written article, and the methods are complete (notwithstanding a few modifications). It brings potential interest in opening up fields of exploration with application in human health. And so I'm in favor of it being considered for publication notwithstanding major revisions.

Author Response:

We greatly appreciate the reviewer's encouraging comments and suggestions that help us to improve the quality of our manuscript. We provide a point-by-point response to all of the comments as follows.

L19: "...often associated with dysfunction of the hypothalamus in the brain" too reductionist replace by "central nervous system" or It will be more appropriate to indicate an impairment of hypothalamic integration of peripheral signals and/or extrahypothalamic structures, as it is well established that defects in central energy homeostasis is the multiple and implicate internals and externals cues.

Author Response:

As the reviewer commented, we revised the sentence to "…often associated with impairments in hypothalamic integration of peripheral signals and/or extrahypothalamic structures".

L56 "its role in maintaining energy balance is also emerging" The role P53 in metabolism is referenced as back as early 2000's, I suggest the author to cite a much earlier reference, but this reference is appropriate towards the role of P53 in regards of hypothalamic neurons.

Author Response:

As the reviewer suggested, we added the reference with a much earlier publication (1) citing the role of p53 in the regulation of metabolism.

L112: missing line withdrawal

Author Response:

We removed the empty line in the revised manuscript.

Major Concerns

"Our study points to a previously unappreciated efficacy of HMBA". The authors highlight the underestimated role of HMBA in the regulation of energy balance (Abstract), however, the authors do not indicate any elements or citations indicating clues to this potential function. It is only in the Results

section, using "The Connectivity Map" interface, that the authors establish their hypothesis of HMBA's role based on homology with Oleylethanolamide. I would suggest that the authors supplement the rationale for HMDA with existing observations of body weight change (publications of Saravanan N as a start which described mixed change in body weight in some model). Indeed, as also indicated by the authors (Supplementary Table 1) other molecules respond with similar homology, so why HMDA? It's a pity that the authors don't offer an explanation for the choice of this molecule.

Author Response:

We are thankful for the reviewer's suggestion of a clear explanation as to why HMBA was chosen for this study. We explored compound candidates through a homology search with oleoylethanolamide to select a potential anti-obesity compound. In the compound list, three compounds including HMBA were not studied yet in the metabolic diseases (Appendix Table S1). Although the roles of otenzepad (a cardioselective muscarinic receptor antagonist) and valacyclovir (anti-herpes medication) on metabolism are unknown, these two drugs were excluded from the candidates due to their adverse effects on the central nervous system, nephrotoxicity, or tissue-specific response in previous studies (2–5). We chose HMBA as a suitable candidate since its effects on obesity are not known at all among the listed compounds. We revised sentences to clearly explain the rationale for the choice of HMBA in the manuscript.

We regret to inform the referee that the compound referred to as HMBA (2-hydroxy-4-methoxy benzoic acid) in Saravanan N's publication is not HMBA (hexamethylene bisacetamide) used in this study. These two compounds are completely different in structures and functions.

Results and Methods

Overall, the choice and sequencing of methods and techniques is sound, well chosen, and supports the authors' questions. However, several details are missing and inappropriate methods analysis, impact the full appreciation of the results.

Address and country of suppliers are missing (ex:Alfa Aesar, Koatech, plastic One etc..) throughout the

method section.

Author Response:

We added the addresses (cities) and countries of several suppliers in the Materials and Methods section.

L426. Indicates the exact times of the light/dark schedule.

Author Response:

We added the exact time (light: 7:00 am – 7:00 pm; dark: 7:00 pm – 7:00 am) of the light/dark schedule in the Animal studies of the Materials and Methods section.

L429: Mandatory, the authors should indicate the reference number of their approved agreement of use of animals and care committee.

Author Response:

Since we initially submitted the manuscript anonymously, we were unable to inform the reference number that includes the name of our institution. We provide the approval number in the revised manuscript.

L460 section: i.v., indicates which vein was chosen. Please, could the authors be more precise about the time of injection.

Author Response:

The time of HMBA administration into the tail vein of mice was already indicated in the calorimetric graphs in Figure 1 N–S. We added the information on injection time (pm 04:30) in the Methods section.

L477: ICV, indicates the volume injected, and the duration of injection. Please indicates the time of

injection. Throughout the methods, indicates when animals were grouped or individualized.

Author Response:

We provided the information on the volume and time of i.c.v. injection, and the housing in the Materials and Methods section.

L499 Calorimetry

Mandatory: 1 day of acclimatization in Colombus cage is very short, plus the authors do not indicate at which day of the injection procedure the animals enter in the calorimetric system. Could the authors provide a more precise experimental design?

Author Response:

Consistent with previous studies (6–8), mice were acclimated in the CLAMS cage for 1 day in this study. Seburn KL from Mouse Phenome Database at The Jackson Laboratory reported that there was no difference in data when mice were acclimatized for 3 days versus 1 day on the same CLAMS system that we used (9). In the i.v. HMBA injected group, we placed the mice in metabolic cages when the 10-day monitoring was terminated. After 1 day of acclimatization in metabolic cages, mice were given an additional injection of HMBA right before calorimetry analysis, and metabolic parameters were monitored for 24 h. We considered the additional injection to appropriately assess the metabolic changes following the injection because i.v. injected HMBA gets rapidly excreted from the body to urine due to a short biological half-life (10–12). In the case of i.c.v. injection of HMBA, we placed the mice in metabolic cages for 1 day of acclimatization after the 21-day monitoring and measured the metabolic parameters without any additional i.c.v. injection.

Please, could the authors provide in supplemental the full figures of calorimetric analysis (Days of adaptation, if available + day of measurement).

Author Response:

As the reviewer suggested, the figures of calorimetric analysis were changed to full figures including the acclimatization data in Figures 1 N–S and 2 I–N.

Major concern.

Mandatory: As the body weight change over time, I would recommend strongly the authors to perform Calorimetric data analysis using body weight as covariates (Virtue, S., Lelliott, C.J. & Vidal-Puig, A. What is the most appropriate covariate in ANCOVA when analyzing metabolic rate?. Nat Metab 3, 1585 (2021). The significant change in EE observed under ICV treatment may be consecutive to the change in body mass. See: Fernández-Verdejo, R., Ravussin, E., Speakman, J.R. et al. Progress and challenges in analyzing rodent energy expenditure. Nat Methods 16, 797-799 (2019).

Author Response:

We appreciate the reviewer's constructive suggestion of a more convincing way of presentation. To maximize the reliability and relevance of the data, we additionally performed the analysis of covariance (ANCOVA) using body mass as the covariate for a regression-based metabolic rate of mice. ANCOVA data demonstrated that i.v. and i.c.v. injection of HMBA led to marked induction in energy expenditure compared to the saline-injected mice, indicating that HMBA increases metabolic rates independently of the body mass. The calorimetric analyses of ANCOVA were added in Figure 1M and Figure 2H and revised in the figure legends and the Method sections.

L513 Brown adipose tissue

Please, indicates how many picture of the animals was taken. In order to properly determine a change in the thermogenic activity of bat in vivo, I suggest that the authors measure the surface temperature of the lower back on the same photos, and then extract the ratio between both regions to normalize the temperature per animal and make appropriate comparison. (Meyer CW, Ootsuka Y, Romanovsky AA. Body Temperature Measurements for Metabolic Phenotyping in Mice. Front Physiol. 2017 Jul 31;8:520. doi: 10.3389/fphys.2017.00520.)

Author Response:

The number of pictures was shown as the values in the temperature graph next to the representative picture (i.v.: n=10-11; i.c.v.: n=7). As the reviewer commented, we indicated the information on how many pictures were taken for the representative picture in the Figure legend. As the reviewer suggested, we measured the surface temperature of the lower back on the same photo to normalize the temperature per animal in i.v. and i.c.v. experiments (Response Figure 1, 2). Since there was no discernible difference in the lower back temperature between saline- and HMBA-treated groups, normalization was not applied to BAT thermogenesis analysis.

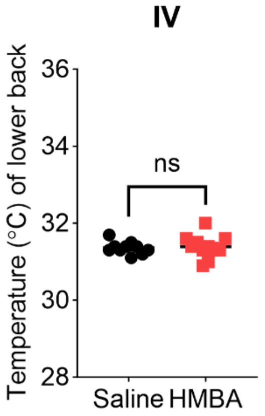

Response Figure 1

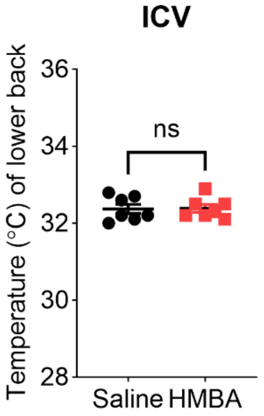

Response Figure 2

Fig 1 and 2: RER (VCO2/VO2)

L111: Central administration of HMBA

Calorimetric analysis on figures 2 was performed on the last day in contrary of IV injection. Could the authors explain the reason?

Author Response:

We hope that we addressed this point in the reviewer's aforementioned comment (L499 Calorimetry). Because the i.v. injected HMBA gets rapidly excreted from the body to urine due to a short biological half-life, we performed an additional injection after a 10-day monitoring. In the case of i.c.v. injection,

we did not conduct the additional injection.

Please provides the food intake pattern if available with the calorimetric analysis.

Author Response:

Unfortunately, we did not collect data on food intake patterns during the calorimetric analysis due to a technical problem with the food measurement component of the CLAMS system. As an alternative, the data of the Laboratory Animal Behavior Observation Registration and Analysis System (LABORAS) provide eating behavior that shows a reduction in eating time in HMBA-injected mice (Expanded View Figure 1A, 1D, 1G). We hope that analyzing dietary behavior using LABORAS could address the reviewer’s question.

Please indicates on the Fig 1b and 2b, when treatment occurs or stops.

Author Response:

As the reviewer commented, we indicated each injection in the graphs of Figures 1C–F and 2B, 2C.

Major concern.

Food intake (HFD) decrease dramatically at the end of the 7 days of treatment. Such a reduction in food intake jeopardize the animal's health. Although the authors indicate: (1) a change in the expression of hypothalamic peptides involved in the regulation of energy balance, following the knocking down MYH9 and ACTG1 (downstream of HMBA) and thus that changes could drive the change in energy balance, It cannot be ruled out that the dramatic change in intake may also be due to the deleterious nature of the molecule of interest.

Author Response:

We understand the concern raised by the reviewer. To address the reviewer’s concern, we provide

critical evidence that the dose we used in vivo did not cause any sickness behavior. First, we observed that none of the mice displayed any noticeable signs of sickness such as vomiting, diarrhea, inactivity, and hair loss during HMBA experiments. Our metabolic cage data also showed no negative effect of HMBA on locomotor activity compared with saline-injected mice. Secondly, in order to demonstrate the health status of the mice more accurately, we employed the laboratory animal behavior observation registration and analysis system (LABORAS), a robust tool renowned for its capability in automated behavioral scoring, after the last dosing of HMBA (i.v., i.p., or i.c.v.). This sophisticated system accurately measures a range of behaviors, including immobility, rearing, locomotion, climbing, grooming, eating, and drinking. Our LABORAS data demonstrated that eating was decreased by HMBA treatment, however, no significant differences in the other behaviors were observed compared to the saline-injected group (Expanded View Figure 1A, 1B, 1D, 1E, 1G, 1H). These results indicate that HMBA does not induce sickness behaviors in mice. Lastly, we performed a conditioned taste aversion (CTA) test to assess the effect of HMBA on a taste aversion associated with an illness over sucrose preference. The CTA test showed that HMBA did not induce a taste aversion, either (Expanded View Figure 1C, 1F, 1I). Collectively, our data demonstrate that HMBA did not exhibit sickness-associated or aversive appetite suppression and weight loss under our experimental conditions. The details are elaborated in the Results of the revised manuscript.

L152, L201, Fig3, F4.

Very nice parts, authors well demonstrate in vivo and in vitro the dependent relationship form HMBA exposure, change in Hexim1 expression, determinant role of Myh9 and Actg1, hence expression of NPY and POMC. However, the authors should indicate whether HMBA exposure compromises cell survival and/or presents a pro-inflammatory or apoptotic state. Those missing information would reenforce the finding and avoid implying that the molecule is potentially harmful.

Author Response:

We fully agree with the reviewer's opinion. We have already demonstrated that HMBA (0.1 mM) does not induce apoptosis as evidenced by no increases in cleaved forms of caspases 3 and 9 (Appendix

Figure S8). As the reviewer suggested, we added data on cell viability across the dynamic range of HMBA treatment (0.001-10 mM) to ensure its non-toxicity (Appendix Figure S4). According to the data, the concentration of HMBA (0.1 mM) used in this study was within the range that did not induce apoptosis or reduce cell survival. In addition, in vivo behavioral assessments in mice after i.v., i.p., and i.c.v. injections of HMBA showed no sickness in the animal. Collectively, the concentrations of HMBA used in our in vitro and in vivo studies have no harmful effect.

L334. Fig 7g. Nice summary and nice graph.

Author Response:

We greatly appreciate the reviewer's nice comments.

Discussion: Well written, but overestimated interpretation, as metabolic data must be revisited according to the above suggestions.

Author Response:

We are thankful for the reviewer's appreciation of our work. We earnestly hope that our responses successfully conveyed the information as requested by the reviewer.

References

1. Yahagi N et al., *J Biol Chem*, 2003;278(28):25395-25400

2. Broadley KJ et al., *Molecules*, 2001;6(3):142-193

3. Brandariz-Nunez D et al., *J Clin Pharm Ther*, 2021;46(4):918-926

4. Zhang Y et al., *Exp Ther Med*, 2016;12(6):4025-4028

5. Mickala P et al., *Nucl Med Biol*, 1996;23(2):173-179

6. Shu L et al., *Nat Comm*, 2017;8:14147

7. Luk CT et al., *Nat Comm*, 2017;8:14360

8. Xia T et al., *Diabetes*, 2012;61(10):2461-2471

9. https://phenome.jax.org/projects/Seburn1/protocol?method=intake+monitoring

10. Egorin MJ et al., *Cancer Res*, 1987;47:6142-6146

11. Egorin MJ et al., *Cancer Res*, 1987;47:617-623

12. Litterst CL et al., *Invest New Drugs*, 1985;3:263-272

9th Oct 2023

Dear Dr. Kim,

Thank you for the submission of your manuscript to EMBO Molecular Medicine. We have now received the feedback from the 3 referees on your revised manuscript. As you will see below, referee #1 still raises some concerns about potential HMBA toxicity. Based on the other referees' reports and after discussion within the team, we would like to invite you to address experimentally or by an adequate discussion this referee's concerns in a minor round of revision. Moreover, please address the following editorial points:

1/ Referee's comments:
Please address the remaining concerns from referees #1 and #3, experimentally when possible, or in writing.

2/ Manuscript text:
- Please remove the blue text, and only keep in track changes mode any new modification.
- Please suggest up to 5 keywords.
- Please remove "data not shown" (p.21): As per our guidelines on "Unpublished Data", the journal does not permit citation of "Data not shown". All data referred to in the paper should be displayed in the main or Expanded View figures.
- Material and methods:
Cell cultures: please indicate whether the cells were tested for mycoplasma contamination and adjust the checklist accordingly.
- Data Availability section: If you have no data that requires deposition in a public database, please state so in this section ('This study includes no data deposited in external repositories'). Note that the Data Availability Section is restricted to new primary data that are part of this study. Please remove "All the data are available in the article and supporting information or are available from the corresponding author upon reasonable request. Source data are provided with this paper.
- Please reformat the references to have 10 authors listed before et al.

3/ Figures and Appendix:
- Main and EV figures need to be uploaded as separate, high resolution figure files.
- The EV figure legends should be in the manuscript, after the main figure legends.
- Please provide exact p values (including for ns, non-significant) in the figures or their legends.
- Carefully check that all figures/figure panels are referenced in the manuscript text (callout for suppl. Figure 8 should be Appendix Figure S8, callout for Appendix Fig. S5 is missing).
- Please note that the box plots need to be defined in terms of minima, maxima, centre, bounds of box and whiskers, and percentile in the legend of figures 1g, h, j, k, l; 2d-g; 5l, n.

4/ At EMBO Press we ask authors to provide source data for the main figures. Our source data coordinator will contact you to discuss which figure panels we would need source data for and will also provide you with helpful tips on how to upload and organize the files.

5/ Checklist:
- Please check the category "Plants and Microbes"
- Please provide information for "Cell Materials", mycoplasma and authentication
- Please provide information in "Statistics", inclusion/exclusion criteria.

6/ Thank you for providing The Paper Explained, please add it to the main manuscript file.

7/ Synopsis: please resize your synopsis as a png/tiff/jpeg file 550 pixels wide x 300-600 pixels high. Please make sure that the text remains legible.

8/ As part of the EMBO Publications transparent editorial process initiative (see our Editorial at http://embomolmed.embopress.org/content/2/9/329), EMBO Molecular Medicine will publish online a Review Process File (RPF) to accompany accepted manuscripts.
In the event of acceptance, this file will be published in conjunction with your paper and will include the anonymous referee reports, your point-by-point response and all pertinent correspondence relating to the manuscript. Let us know whether you agree with the publication of the RPF and as here, if you want to remove or not any figures from it prior to publication.
Please note that the Authors checklist will be published at the end of the RPF.

I look forward to receiving your revised manuscript.

Yours sincerely,

Lise Roth

Lise Roth, PhD
Senior Editor
EMBO Molecular Medicine

***** Reviewer's comments *****

Referee #1 (Comments on Novelty/Model System for Author):

The authors have conducted a substantial number of new experiments to provide additional support for their original claim. These new data address some of my previous concerns, although I remain somewhat skeptical.

The inclusion of new LABORAS data is intriguing, indicating that HMBA may not induce acute negative effects in mice. However, the dose remains notably high, raising questions about its potential therapeutic application in humans. It would be beneficial to explore the effects of lower doses of the drug or conduct a dose-dependent study to determine if similar beneficial effects can be achieved.

The issue of feeding cessation after HMBA administration remains a significant concern, especially when compared to the absence of this effect with current blockbuster GLP1-agonists. Similarly, the findings from the C75 study conducted by others do little to address this concern. Although a reduction in NPY and AgRP was observed in that study, it only establishes an association and not a direct causation, similar to many other studies in the field.

Referee #1 (Remarks for Author):

A great mouse study, but therapeutic potential is limited.

Referee #2 (Comments on Novelty/Model System for Author):

No Major concerns after the revisions

Referee #2 (Remarks for Author):

The authors have adequately addressed my concerns.

Referee #3 (Remarks for Author):

The authors made a real effort to answer all referee points and it allows to strengthen their finding.
Minor correction to do: Fig 1N and Fig 2I. Please change to RER(VCO2/VO2).
The i) the laboras study and ii) the authors explanation about the choice of HMBA amongst other molecules is well appreciated.

In the present forms, including the above correction, I consider the article suitable for publication.

best regards

Reviewer's Comments:

Referee #1 (Comments on Novelty/Model System for Author):

The authors have conducted a substantial number of new experiments to provide additional support for their original claim. These new data address some of my previous concerns, although I remain somewhat skeptical.

The inclusion of new LABORAS data is intriguing, indicating that HMBA may not induce acute negative effects in mice. However, the dose remains notably high, raising questions about its potential therapeutic application in humans. It would be beneficial to explore the effects of lower doses of the drug or conduct a dose-dependent study to determine if similar beneficial effects can be achieved.

The issue of feeding cessation after HMBA administration remains a significant concern, especially when compared to the absence of this effect with current blockbuster GLP1-agonists. Similarly, the findings from the C75 study conducted by others do little to address this concern. Although a reduction in NPY and AgRP was observed in that study, it only establishes an association and not a direct causation, similar to many other studies in the field.

Referee #1 (Remarks for Author):

A great mouse study, but therapeutic potential is limited.

Author Response:

We greatly appreciate the referee's encouraging comments and constructive suggestions that help us to improve the quality of our manuscript. We understand the concern raised by the referee. We included the data on food intake and body weight using lower doses of HMBA in Appendix Fig S12 of the revised manuscript. We found that i.v. or i.p. injections at lower doses (200 or 500 mg/kg) than 1000 mg/kg of HMBA did not have any noticeable effects on appetite and body weight in DIO mice. In contrast, i.c.v. injection of 80 nmoles HMBA, a lower dosage than 400 nmoles, induced a less but still significant reduction in both body weight and food intake. As shown in Response Tables 1 and 2, the amount of HMBA accumulated in the hypothalamus rather than plasma after i.v., i.p., or i.c.v. injections might be important for metabolic benefits. Our results suggest that at least 0.1 μg/g of HMBA accumulated in the hypothalamus might be required to reduce appetite and weight gain. Hence, for potential therapeutic

applications in humans, optimization of HMBA maximizing its metabolic benefits at lower doses by extended half-life and efficient delivery targeted to the hypothalamus will be necessary.

In regard to feeding cessation, it only occurred one day during i.c.v. injection of 400 nmoles of HMBA and the feeding was recovered after the next day. Liraglutide, a GLP-1R agonist, has been also reported to induce a feeding cessation for the initial four days when administered daily via 400 μg/kg subcutaneous injection in DIO mice (*J Biol Chem* 298(12):102682, 2022). However, a lower dose of 80 nmoles HMBA i.c.v. injection, or even a high dose of 1000 mg/kg i.v. or i.p. injection did not cause feeding cessation.

We explored the molecular underpinnings of the anorexigenic response by assessing the transcriptional levels of relevant hypothalamic neuropeptides (*Npy, Agrp, Pomc, Cart*) after HMBA treatment. Although their expression levels per se do not necessarily reflect the direct causation of HMBA effects, we were able to provide evidence that HMBA regulates their expressions which are important for HMBA's roles in controlling feeding and body weight. In our study, MYH9 and ACTG1 were suggested as direct binding target molecules of HMBA to regulate *Npy* and *Pomc* expression in NPY and POMC neurons, respectively. Although we cannot rule out a possibility of another binding target(s) of HMBA in those neurons, it would be worth a precise evaluation of the interaction between HMBA and its binding targets, and any other downstream of HMBA-MYH9/ACTG1 beyond the expression of the neuropeptide in further in-depth studies, which may help understanding the direct causation of HMBA effects. We earnestly hope that our responses can address the referee's concerns.

Response Table 1. HMBA concentrations in the hypothalamus after injections

	Fig 1G			Fig 3C	
**(Mean values)**	**HMBA Conc.**	**i.v. (μg/g)**	**i.p. (μg/g)**	**HMBA Conc.**	**i.c.v. (μg/g)**
**Concentration**	1000 mg/kg	0.21	0.11	400 nmole	159.95

Response Table 2. HMBA concentrations in plasma after injections

	Fig 1G			Appendix Fig S3	
**(Mean values)**	**HMBA Conc.**	**i.v. (μg/ml)**	**i.p. (μg/ml)**	**HMBA Conc.**	**i.c.v. (μg/ml)**
**Concentration**	1000 mg/kg	1.68	1.04	400 nmole	0.13

Referee #2 (Comments on Novelty/Model System for Author):

No Major concerns after the revisions.

Referee #2 (Remarks for Author):

The authors have adequately addressed my concerns.

Author Response:

We thank the referee for the insightful suggestions that greatly improved our manuscript.

Referee #3 (Remarks for Author):

The authors made a real effort to answer all referee points and it allows to strengthen their finding.

Minor correction to do: Fig 1N and Fig 2I. Please change to RER(VCO2/VO2).

The i) the laboras study and ii) the authors explanation about the choice of HMBA amongst other molecules is well appreciated.

In the present forms, including the above correction, I consider the article suitable for publication.

best regards

Author Response:

We apologize for the mistake; corrected in Fig 1N and Fig 2I of the revised manuscript. We thank the referee for the valuable remarks, which significantly improved the paper.

27th Oct 2023

Dear Prof. Kim,

I am pleased to inform you that your manuscript is accepted for publication and is now being sent to our publisher to be included in the next available issue of EMBO Molecular Medicine.

Please note that, according to your cover letter and as mentioned in a previous correspondence, I have added the following section after your Materials and Methods:

"Data Availability:
This study includes no data deposited in external repositories."

Let us know immediately if you disagree.

Please read below for additional IMPORTANT information regarding your article, its publication and the production process.

Congratulations on your interesting work!

With kind regards,

Lise Roth

Lise Roth, Ph.D
Senior Editor
EMBO Molecular Medicine

Follow us on Twitter @EmboMolMed
Sign up for eTOCs at embopress.org/alertsfeeds

*** *** *** IMPORTANT INFORMATION *** *** ***

SPEED OF PUBLICATION
The journal aims for rapid publication of papers, using using the advance online publication "Early View" to expedite the process: A properly copy-edited and formatted version will be published as "Early View" after the proofs have been corrected. Please help the Editors and publisher avoid delays by providing e-mail address(es), telephone and fax numbers at which author(s) can be contacted.

Should you be planning a Press Release on your article, please get in contact with embomolmed@wiley.com as early as possible, in order to coordinate publication and release dates.

LICENSE AND PAYMENT:

All articles published in EMBO Molecular Medicine are fully open access: immediately and freely available to read, download and share.

EMBO Molecular Medicine charges an article processing charge (APC) to cover the publication costs. You, as the corresponding author for this manuscript, should have already received a quote with the article processing fee separately. Please let us know in case this quote has not been received.

Once your article is at Wiley for editorial production you will receive an email from Wiley's Author Services system, which will ask you to log in and will present you with the publication license form for completion. Within the same system the publication fee can be paid by credit card, an invoice, pro forma invoice or purchase order can be requested.

Payment of the publication charge and the signed Open Access Agreement form must be received before the article can be

published online.

PROOFS

You will receive the proofs by e-mail approximately 2 weeks after all relevant files have been sent o our Production Office. Please return them within 48 hours and if there should be any problems, please contact the production office at embopressproduction@wiley.com.

Please inform us if there is likely to be any difficulty in reaching you at the above address at that time. Failure to meet our deadlines may result in a delay of publication.

All further communications concerning your paper proofs should quote reference number EMM-2023-18024-V4 and be directed to the production office at embopressproduction@wiley.com.

EMBO Press Author Checklist

Corresponding Author Name: Eun-Kyoung Kim	
Journal Submitted to: 20th October 2023	
Manuscript Number: EMM-2023-18024-4	

USEFUL LINKS FOR COMPLETING THIS FORM
The EMBO Journal - Author Guidelines
EMBO Reports - Author Guidelines
Molecular Systems Biology - Author Guidelines
EMBO Molecular Medicine - Author Guidelines

Reporting Checklist for Life Science Articles (updated January
This checklist is adapted from Materials Design Analysis Reporting (MDAR) Checklist for Authors. MDAR establishes a minimum set of requirements in transparent reporting in the life sciences (see Statement of Task: 10.31222/osf.io/9sm4x). Please follow the journal's guidelines in preparing your
Please note that a copy of this checklist will be published alongside your article.

Abridged guidelines for figures
1. Data
The data shown in figures should satisfy the following conditions:
- the data were obtained and processed according to the field's best practice and are presented to reflect the results of the experiments in an accurate and unbiased manner.
- ideally, figure panels should include only measurements that are directly comparable to each other and obtained with the same assay.
- plots include clearly labeled error bars for independent experiments and sample sizes. Unless justified, error bars should not be shown for technical
- if n<5, the individual data points from each experiment should be plotted. Any statistical test employed should be justified.
- Source Data should be included to report the data underlying figures according to the guidelines set out in the authorship guidelines on Data

2. Captions
Each figure caption should contain the following information, for each panel where they are relevant:
- a specification of the experimental system investigated (eg cell line, species name).
- the assay(s) and method(s) used to carry out the reported observations and measurements.
- an explicit mention of the biological and chemical entity(ies) that are being measured.
- an explicit mention of the biological and chemical entity(ies) that are altered/varied/perturbed in a controlled manner.
- the exact sample size (n) for each experimental group/condition, given as a number, not a range;
- a description of the sample collection allowing the reader to understand whether the samples represent technical or biological replicates (including how many animals, litters, cultures, etc.).
- a statement of how many times the experiment shown was independently replicated in the laboratory.
- definitions of statistical methods and measures:
  - common tests, such as t-test (please specify whether paired vs. unpaired), simple χ2 tests, Wilcoxon and Mann-Whitney tests, can be unambiguously identified by name only, but more complex techniques should be described in the methods section;
  - are tests one-sided or two-sided?
  - are there adjustments for multiple comparisons?
  - exact statistical test results, e.g., P values = x but not P values < x;
  - definition of 'center values' as median or average;
  - definition of error bars as s.d. or s.e.m.

Please complete ALL of the questions below.
Select "Not Applicable" only when the requested information is not relevant for your study.

Materials

Newly Created Materials	Information included in the manuscript?	In which section is the information available? (Reagents and Tools Table, Materials and Methods, Figures, Data Availability Section)
New materials and reagents need to be available; do any restrictions apply?	Not Applicable	

Antibodies	Information included in the manuscript?	In which section is the information available? (Reagents and Tools Table, Materials and Methods, Figures, Data Availability Section)
For **antibodies** provide the following information:<br>- Commercial antibodies: RRID (if possible) or supplier name, catalogue number and or/clone number<br>- Non-commercial: RRID or citation	Yes	Supporting Information

DNA and RNA sequences	Information included in the manuscript?	In which section is the information available? (Reagents and Tools Table, Materials and Methods, Figures, Data Availability Section)
**Short novel DNA or RNA including primers, probes**: provide the sequences.	Yes	Materials and Methods, and Supporting Information

Cell materials	Information included in the manuscript?	In which section is the information available? (Reagents and Tools Table, Materials and Methods, Figures, Data Availability Section)
**Cell lines:** Provide species information, strain. Provide accession number in repository **OR** supplier name, catalog number, clone number, and/**OR** RRID.	Yes	Materials and Methods
**Primary cultures:** Provide species, strain, sex of origin, genetic modification status.	Not Applicable	
Report if the cell lines were recently **authenticated** (e.g., by STR profiling) and tested for mycoplasma contamination.	Yes	Materials and Methods

Experimental animals	Information included in the manuscript?	In which section is the information available? (Reagents and Tools Table, Materials and Methods, Figures, Data Availability Section)
**Laboratory animals or Model organisms:** Provide species, strain, sex, age, genetic modification status. Provide accession number in repository **OR** supplier name, catalog number, clone number, **OR** RRID.	Yes	Materials and Methods
**Animal observed in or captured from the field:** Provide species, sex, and age where possible**.**	Not Applicable	
Please detail **housing and husbandry conditions**.	Yes	Materials and Methods

Plants and microbes	Information included in the manuscript?	In which section is the information available? (Reagents and Tools Table, Materials and Methods, Figures, Data Availability Section)
**Plants:** provide species and strain, ecotype and cultivar where relevant, unique accession number if available, and source (including location for collected wild specimens).	Not Applicable	
**Microbes:** provide species and strain, unique accession number if available, and source.	Not Applicable	

Human research participants	Information included in the manuscript?	In which section is the information available? (Reagents and Tools Table, Materials and Methods, Figures, Data Availability Section)
If collected and within the bounds of privacy constraints report on age, sex and gender or ethnicity for all study participants.	Not Applicable	

Core facilities	Information included in the manuscript?	In which section is the information available? (Reagents and Tools Table, Materials and Methods, Figures, Data Availability Section)
If your work benefited from core facilities, was their service mentioned in the acknowledgments section?	Not Applicable	

Design

**Study protocol**	**Information included in the manuscript?**	**In which section is the information available?** (Reagents and Tools Table, Materials and Methods, Figures, Data Availability Section)
If study protocol has been **pre-registered, provide DOI in the manuscript**. For clinical trials, provide the trial registration number **OR** cite DOI.	Not Applicable	
Report the **clinical trial registration number** (at ClinicalTrials.gov or equivalent), where applicable.	Not Applicable	

**Laboratory protocol**	**Information included in the manuscript?**	**In which section is the information available?** (Reagents and Tools Table, Materials and Methods, Figures, Data Availability Section)
Provide DOI OR other citation details if **external detailed step-by-step protocols** are available.	Not Applicable	

**Experimental study design and statistics**	**Information included in the manuscript?**	**In which section is the information available?** (Reagents and Tools Table, Materials and Methods, Figures, Data Availability Section)
Include a statement about **sample size** estimate even if no statistical methods were used.	Yes	Materials and Methods
Were any steps taken to minimize the effects of subjective bias when allocating animals/samples to treatment (e.g. **randomization procedure**)? If yes, have they been described?	Yes	Materials and Methods
Include a statement about **blinding** even if no blinding was done.	Yes	Materials and Methods
Describe **inclusion/exclusion criteria** if samples or animals were excluded from the analysis. Were the criteria pre-established?<br><br>If sample or data points were omitted from analysis, report if this was due to attrition or intentional exclusion and provide justification.	Yes	Materials and Methods, no data points were excluded
For every figure, are **statistical tests** justified as appropriate? Do the data meet the assumptions of the tests (e.g., normal distribution)? Describe any methods used to assess it. Is there an estimate of variation within each group of data? Is the variance similar between the groups that are being statistically compared?	Yes	Figures, and Materials and Methods

**Sample definition and in-laboratory replication**	**Information included in the manuscript?**	**In which section is the information available?** (Reagents and Tools Table, Materials and Methods, Figures, Data Availability Section)
In the figure legends: state number of times the experiment was **replicated** in laboratory.	Yes	Figures
In the figure legends: define whether data describe **technical or biological replicates**.	Yes	Figures

Ethics

**Ethics**	**Information included in the manuscript?**	**In which section is the information available?** (Reagents and Tools Table, Materials and Methods, Figures, Data Availability Section)
Studies involving **human participants**: State details of **authority granting ethics approval** (IRB or equivalent committee(s), provide reference number for approval.	Not Applicable	
Studies involving **human participants**: Include a statement confirming that **informed consent** was obtained from all subjects and that the experiments conformed to the principles set out in the WMA Declaration of Helsinki and the Department of Health and Human Services Belmont Report.	Not Applicable	
Studies involving **human participants:** For publication of **patient photos**, include a statement confirming that consent to publish was obtained.	Not Applicable	
Studies involving experimental **animals**: State details of **authority granting ethics approval** (IRB or equivalent committee(s), provide reference number for approval. Include a statement of compliance with ethical regulations.	Yes	Materials and Methods
Studies involving **specimen and field samples:** State if relevant **permits** obtained, provide details of authority approving study; if none were required, explain why.	Not Applicable	

**Dual Use Research of Concern (DURC)**	**Information included in the manuscript?**	**In which section is the information available?** (Reagents and Tools Table, Materials and Methods, Figures, Data Availability Section)
Could your study fall under dual use research restrictions? Please check biosecurity documents and list of **select agents and toxins** (CDC): https://www.selectagents.gov/sat/list.htm	Not Applicable	
If you used a select agent, is the security level of the lab appropriate and reported in the manuscript?	Not Applicable	
If a study is subject to dual use research of concern regulations, is the name of the **authority granting approval and reference number** for the regulatory approval provided in the manuscript?	Not Applicable	

Reporting

The MDAR framework recommends adoption of discipline-specific guidelines, established and endorsed through community initiatives. Journals have their own policy about requiring specific guidelines and recommendations to complement MDAR.

**Adherence to community standards**	**Information included in the manuscript?**	**In which section is the information available?** (Reagents and Tools Table, Materials and Methods, Figures, Data Availability Section)
State if relevant guidelines or checklists (e.g., **ICMJE, MIBBI, ARRIVE, PRISMA**) have been followed or provided.	Not Applicable	
For **tumor marker prognostic studies,** we recommend that you follow the **REMARK** reporting guidelines (see link list at top right). See author guidelines, under 'Reporting Guidelines'. Please confirm you have followed these guidelines.	Not Applicable	
For **phase II and III randomized controlled trials**, please refer to the **CONSORT** flow diagram (see link list at top right) and submit the CONSORT checklist (see link list at top right) with your submission. See author guidelines, under 'Reporting Guidelines'. Please confirm you have submitted this list.	Not Applicable	

Data Availability

**Data availability**	**Information included in the manuscript?**	**In which section is the information available?** (Reagents and Tools Table, Materials and Methods, Figures, Data Availability Section)
Have **primary datasets** been deposited according to the journal's guidelines (see 'Data Deposition' section) and the respective accession numbers provided in the Data Availability Section?	Not Applicable	
Were **human clinical and genomic datasets** deposited in a public access-controlled repository in accordance to ethical obligations to the patients and to the applicable consent agreement?	Not Applicable	
Are **computational models** that are central and integral to a study available without restrictions in a machine-readable form? Were the relevant accession numbers or links provided?	Not Applicable	
If publicly available data were reused, provide the respective **data citations in the reference list**.	Not Applicable	
